# Discovery of Cell-Permeable Allosteric Inhibitors of Liver Pyruvate Kinase: Design and Synthesis of Sulfone-Based Urolithins

**DOI:** 10.3390/ijms25147986

**Published:** 2024-07-22

**Authors:** Shazia Iqbal, Md. Zahidul Islam, Sajda Ashraf, Woonghee Kim, Amal A. AL-Sharabi, Mehmet Ozcan, Essam Hanashalshahaby, Cheng Zhang, Mathias Uhlén, Jan Boren, Hasan Turkez, Adil Mardinoglu

**Affiliations:** 1 Trustlife Labs Drug Research & Development Center, 34774 Istanbul, Türkiye; shazia.iqbal@trustlifelabs.com (S.I.); sajda.ashraf@trustlifelabs.com (S.A.); amal.sharabi@trustlifelabs.com (A.A.A.-S.); essam.hanash@trustlifelabs.com (E.H.); 2 Science for Life Laboratory, KTH-Royal Institute of Technology, SE-17121 Stockholm, Sweden; woonghee.kim@scilifelab.se (W.K.); cheng.zhang@scilifelab.se (C.Z.); mathias.uhlen@scilifelab.se (M.U.); 3 Department of Pharmaceutical Chemistry, Faculty of Pharmacy, Anadolu University, 26470 Eskişehir, Türkiye; 4 Department of Medical Biochemistry, Faculty of Medicine, Zonguldak Bulent Ecevit University, 67100 Zonguldak, Türkiye; m.ozcan@beun.edu.tr; 5 Department of Molecular and Clinical Medicine, University of Gothenburg, Sahlgrenska University Hospital, 413 45 Gothenburg, Sweden; jan.boren.wlab@gmail.com; 6 Department of Medical Biology, Faculty of Medicine, Atatürk University, 25240 Erzurum, Türkiye; hasanturkez@gmail.com; 7 Centre for Host-Microbiome Interactions, Faculty of Dentistry, Oral & Craniofacial Sciences, King’s College London, London SE1 9RT, UK

**Keywords:** MAFLD, allosteric PKL inhibition, TAG content, sulfone-based urolithin analogues, pyruvate kinase liver, cell permeability

## Abstract

Metabolic dysfunction-associated fatty liver disease (MAFLD) presents a significant global health challenge, characterized by the accumulation of liver fat and impacting a considerable portion of the worldwide population. Despite its widespread occurrence, effective treatments for MAFLD are limited. The liver-specific isoform of pyruvate kinase (PKL) has been identified as a promising target for developing MAFLD therapies. Urolithin C, an allosteric inhibitor of PKL, has shown potential in preliminary studies. Expanding upon this groundwork, our study delved into delineating the structure-activity relationship of urolithin C via the synthesis of sulfone-based urolithin analogs. Our results highlight that incorporating a sulfone moiety leads to substantial PKL inhibition, with additional catechol moieties further enhancing this effect. Despite modest improvements in liver cell lines, there was a significant increase in inhibition observed in HepG2 cell lysates. Specifically, compounds **15d**, **9d**, **15e**, **18a**, **12d**, and **15a** displayed promising IC_50_ values ranging from 4.3 µM to 18.7 µM. Notably, compound **15e** not only demonstrated a decrease in PKL activity and triacylglycerol (TAG) content but also showed efficient cellular uptake. These findings position compound **15e** as a promising candidate for pharmacological MAFLD treatment, warranting further research and studies.

## 1. Introduction

**Figure 1:**
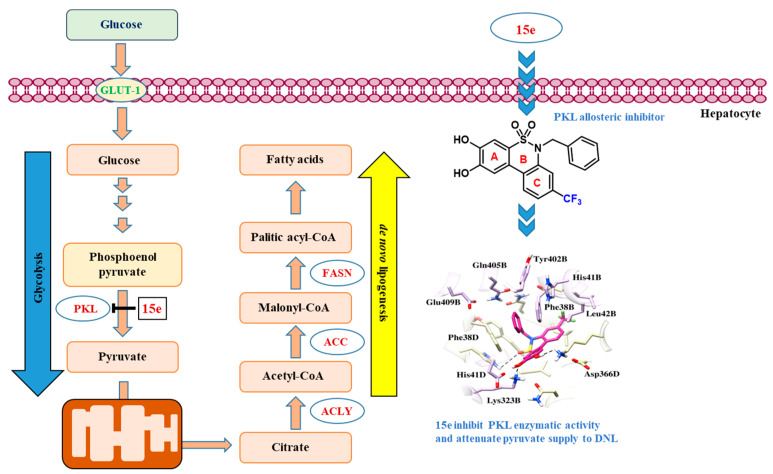
Metabolic Pathway and Key Players Involved in MAFLD.

Pyruvate kinase (PK) is a critical enzyme in glycolysis, the metabolic pathway that breaks down glucose to produce energy [1,2]. PK catalyzes the non-reversible, final step in the cytoplasmic metabolic pathway, converting phosphoenolpyruvate to pyruvate and adenosine triphosphate (ATP), using adenosine diphosphate (ADP) as a substrate (Figure 1) [3]. PK exists in four distinct isoforms in various vital organs of the human body, named PKL, PKR, PKM1, and PKM2 [4]. Although multiple PK isoforms might be present within a single tissue, specific cells in different organs predominantly express one PK isoform [5]. Recent studies have reported that the PKM2 isoform is exclusively present in most adult tissues, whereas the PKL and PKLR isoforms are primarily found in the liver and intestines, pancreatic β-cells, and renal proximal tubules, depending on the cell type [6]. Notably, the PKL isoform is significantly expressed in the liver, suggesting that cell-permeable allosteric inhibitors of these enzymes could be effectively used to prevent or treat MAFLD [7].

The accumulation of fat in the liver is now known to be a mechanism that contributes to hepatic injury in the context of metabolic diseases, such as Metabolic Dysfunction-Associated Fatty Liver Disease (MAFLD). This condition leads to inflammation and a progressive decline in liver health, marked by stages such as steatosis, fibrosis, ballooning degeneration, and ultimately cirrhosis, a signaling end-stage liver disease [8]. Recently, MAFLD has emerged as a significant global health concern. Historically, it was more common in developing countries due to poor dietary habits and a lack of health awareness, but now it is prevalent in developed countries.

For the time being, it is estimated that 25% of the global population is affected by MAFLD, a figure that is projected to rise in the foreseeable future [9,10]. Despite the severe risks associated with MAFLD, including its potential to shorten life expectancy by an estimated five years and its progression to liver cancer, a definitive treatment has yet to be identified. Presently, the recommendations for managing this condition include vitamin E supplements and regular physical activity [11]. Furthermore, glucagon-like peptide-1 (GLP-1) receptor agonists have also emerged as a potential treatment option for patients with MAFLD [12]. However, there is a critical need to identify and develop new pharmaceuticals and biomarkers for diagnosing and effectively treating MAFLD and other liver diseases, including liver cancer. 

To date, the literature has not reported sufficient experimental results demonstrating the efficacy of a cell-permeable allosteric inhibitor of PKL, highlighting a significant gap in the therapeutic landscape for these liver conditions. Moreover, current studies have not conclusively determined whether polyphenolic compounds can penetrate human cells, a crucial aspect of their effectiveness in treating cellular-level disorders [13,14,15,16,17,18,19,20,21,22,23,24]. Therefore, recent studies have been conducted to develop PKLR inhibitors, with some showing active bioavailability in cell lysates of hepatic cancer lines [20]. Nonetheless, the ability of these inhibitors to penetrate human cells and achieve optimal pharmacological efficacy remains a question of significant importance, requiring thorough and serious procedures to simplify the concept. The bioactivity of a drug is intricately linked to its physicochemical properties, cell permeability, and ability to interact with specific residues of the target substrate. The development of effective and efficient bioactive compounds involves medicinal chemists meticulously designing molecules with these factors in mind. Therefore, developing drugs that are both efficacious and capable of cell penetration requires a delicate balance of hydrophilic, hydrophobic, lipophilic, acidic, and basic molecular properties, a challenging task for medicinal chemists [25]. The fluorine atom, as the most electronegative element in the periodic table, plays a pivotal role in the structural composition of many commercially available drugs [26,27,28]. Incorporating fluorine into a compound can endow the molecule with acidic and mildly lipophilic characteristics, making it a key element in the design of bioactive drugs. Furthermore, fluorination can enhance interactions with crucial substrate residues and provide metabolic stability against cytochrome P-450 liver enzymes, thereby improving the drug’s efficacy and longevity [25]. 

## 2. Rationale and Drug Design Strategies

In the literature, ellagic acid (1) and its natural metabolites, urolithin C (2) and urolithin D (3), have been identified as allosteric inhibitors of PKL. These compounds exhibit inhibition rates of 21%, 33%, and 37% at a concentration of 10 μM in liver cell lysate [20]. Various synthetic modifications of urolithin C have resulted in the discovery of potent allosteric PKL modulators. While these modifications showed enhanced inhibition in HepG2 cell lysate, their efficacy in liver cell lines was less pronounced [11].

**Figure 2:**
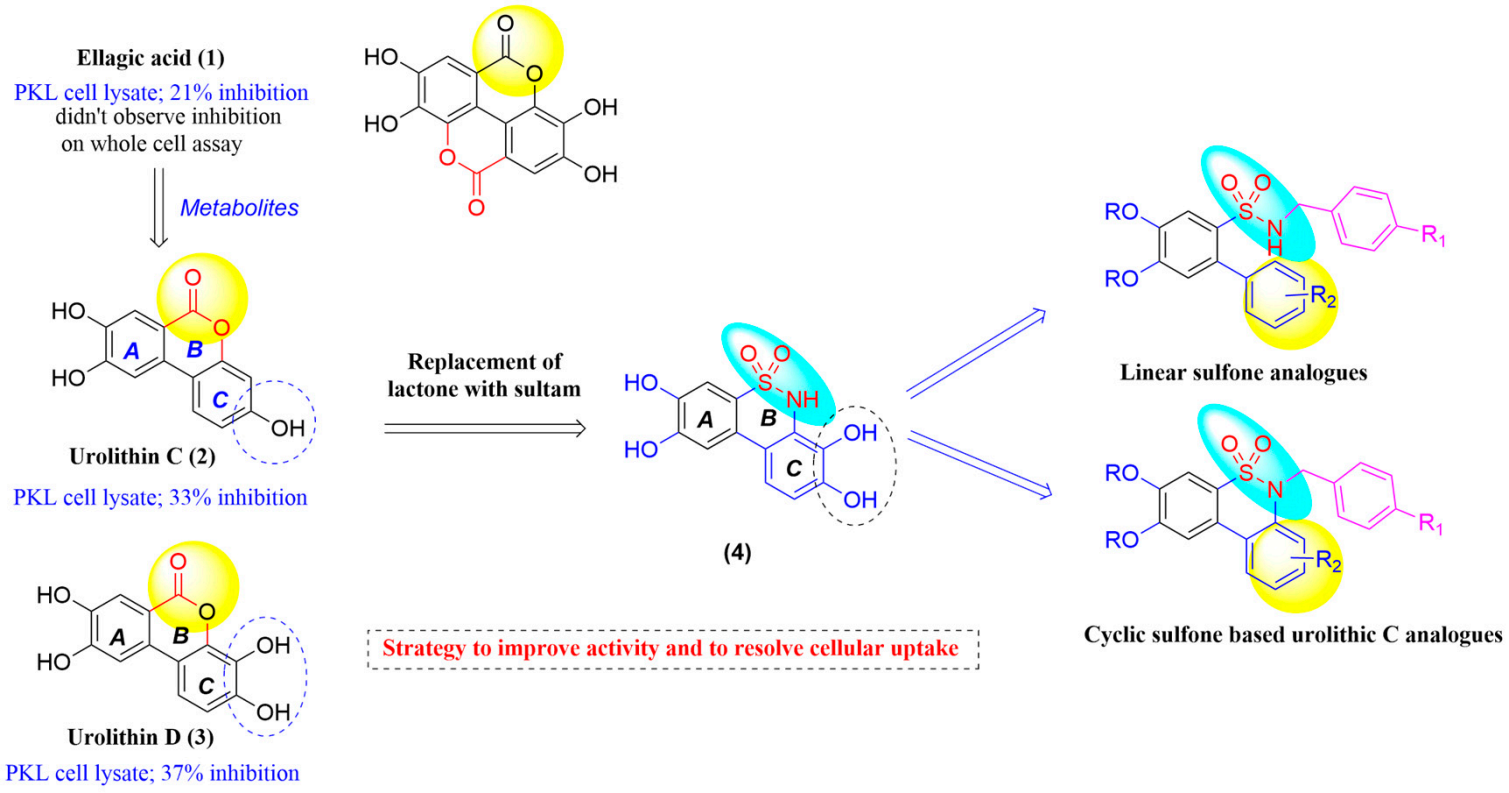
Structural basis of ellagic acid (1), urolithin C (2), urolithin D (3), and their synthetic derivative (4), along with the proposed design of sulfonamides and sultam derivatives featuring a substituted benzylamine ‘handle’ for the development of PKL inhibitors.

The effectiveness of these compounds, including their bioactivity, cell permeability, and interaction with specific residues of the target substrate, is largely influenced by the balance of their acidic, basic, hydrophilic, hydrophobic, and lipophilic properties [25]. Fine-tuning of polarity and hydrophilicity play crucial roles in ensuring good inhibitory activity against PKL. Based on reported SAR studies and PKL inhibition data, we focused on enhancing the inhibition in liver cell lysate while identifying critical features for further exploration (Figure 2).

**Figure 3:**
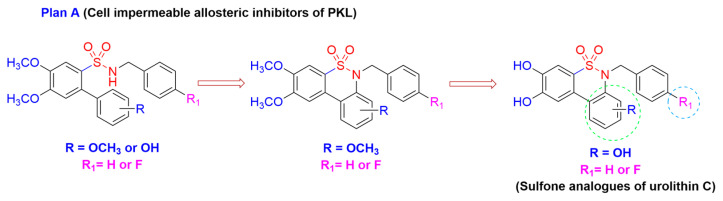
Synthesis of sulfone-based urolithin C compounds.

Our research has initially demonstrated promising results, indicating the bioactivity of these compounds in cell lysate and suggesting potential therapeutic effects. However, a significant challenge remains ineffective cellular penetration. This highlights the complexity of drug design and the necessity for innovative approaches to improve the cellular uptake of sulfone-based urolithin analogs (Plan A) (Figure 3).

**Figure 4:**
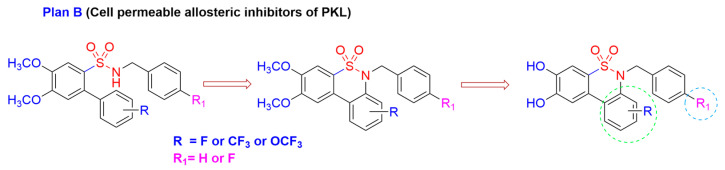
Synthesis of sulfone-based urolithin C analogs.

A significant breakthrough was achieved through the development of cell-permeable and bioactively potent allosteric PKL inhibitors. This was accomplished by incorporating highly electronegative fluorine atoms into sulfone-based urolithin C structures (referred to as Plan B), as shown in Figure 4. This approach effectively utilized the unique properties of fluorine to improve the pharmacological profile of the compounds, aiding in their entry into cells and interaction with the PKL enzyme to support their inhibitory action. 

According to reported data, the presence of a trifluoromethyl group enhances drug potency by lowering the pKa of the cyclic carbamate, forming a crucial hydrogen bonding interaction with the protein [29]. The electron-withdrawing nature, hydrophobic domain, and inert carbon-fluorine linkages of the -CF_3_ group contribute to improved pharmacodynamic and kinetic profiles of drug candidates. Our main goal is to enhance cellular uptake by functionalizing the -OCH_3_ and -OH groups on ring C with -CF_3_ or other substituents to improve cell permeability and refine drug-like properties.

## 3. Results and Discussion

### 3.1. Synthesis of Inhibitors

#### 3.1.1. Biaryl Sulfonamides

**Scheme 1:**
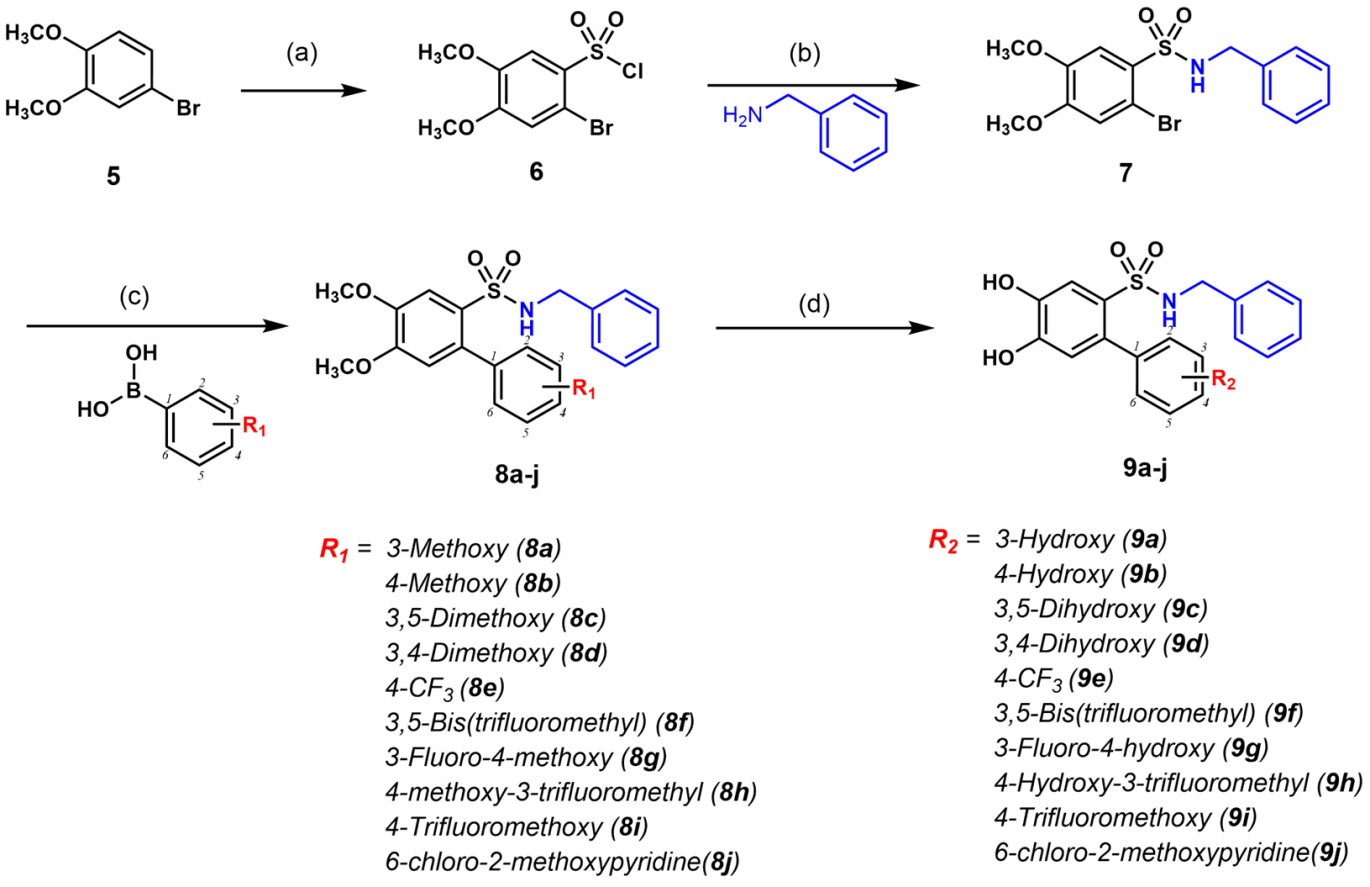
Synthesis of biaryl sulfonamides (with a benzyl group); reagents and conditions: (a) HSO_3_Cl, 0 °C, 15 min, 82%; (b) DIPEA, CH_2_Cl_2_, r.t., 1 h, 90%; (c) Pd (PPh_3_)_4_, toluene:EtOH:water/5:2:1, 120 °C, MW, 1 h, 72–92%; (d) BBr_3_, DCM, r.t., o/n, 52–75%.

Scheme 1 illustrates the synthesis of biaryl sulfonamides with a benzyl group. The process begins with the electrophilic aromatic substitution of commercially available 4-bromoveratrole **5** and chlorosulfonic acid, resulting in the formation of sulfonyl chloride **6** [21]. Using 2-bromo-4,5-dimethoxybenzene-1-sulfonyl chloride 6 as a starting material, nucleophilic substitution with the appropriate benzylamine takes place, yielding sulfonamide **7** with a high yield of 90% after crystallization from methanol. Compound **7** serves as a common starting material already equipped with a benzylamine functional group. The subsequent step involves Suzuki cross-coupling of **7** with suitable boronic acids, leading to the synthesis of biphenyl derivatives of benzyl-sulfonamides (**8a**–**j**) with yields ranging from 72% to 92%. Deprotection of the compounds is achieved using BBr_3_, resulting in the demethylation of the compounds (**9a**–**j**) with yields ranging from 52% to 75%. Finally, the compounds are purified using reverse-phase flash chromatography.

**Scheme 2:**
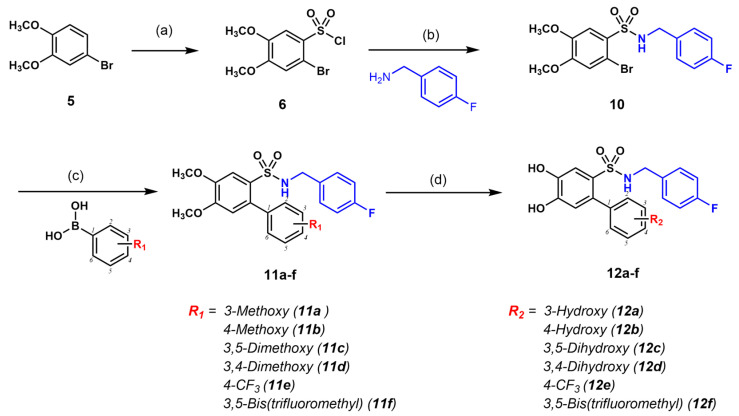
Synthesis of biaryl sulfonamides (with a fluorobenzyl group); reagents and conditions: (a) HSO_3_Cl, 0 °C, 15 min, 82%; (b) DIPEA, CH_2_Cl_2_, r.t., 1 h, 82%; (c) Pd (PPh_3_)_4_, toluene:EtOH:water/5:2:1, 120 °C, MW, 1 h, 67–88%; (d) BBr_3_, DCM, r.t., o/n, 52–72%.

Scheme 2 illustrates the synthesis of biaryl sulfonamides with a fluorobenzyl group. The process began with 2-bromo-4,5-dimethoxybenzene-1-sulfonyl chloride **6**, which underwent nucleophilic substitution with fluorobenzylamine. This reaction resulted in the formation of sulfonamide **10**, with a yield of 82% after crystallization from methanol and diethyl ether. Compound **10** served as a starting material already equipped with a fluorobenzylamine group.

The next step involved Suzuki cross-coupling **10** with appropriate boronic acids. This reaction led to the formation of biphenyl-derivatives of fluorobenzyl-sulfonamides, specifically **11a**–**f**, with yields ranging from 67% to 88%. To remove the protecting groups, BBr_3_ was used, resulting in the demethylation of the compounds. This step yielded demethylated compounds, namely **12a**–**f**, with yields ranging from 52% to 72%.

To purify the synthesized compounds, reverse-phase flash chromatography was employed. This purification method ensured the isolation of the desired biaryl sulfonamides with a fluorobenzyl group.

#### 3.1.2. Sultam Derivatives of Urolithin C

**Scheme 3:**
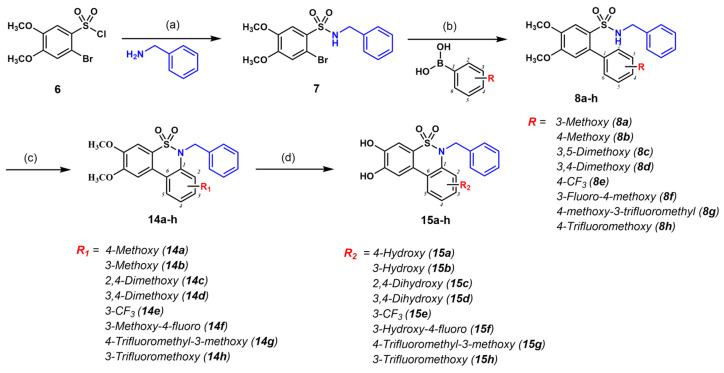
Synthesis of sultam derivatives of urolithin C (with benzyl group); Reagents and conditions: (a) DIPEA, CH_2_Cl_2_, r.t., 1 h, 90%; (b) Pd (PPh_3_)_4_, toluene:EtOH:water/5:2:1, 120 °C, MW, 1 h, 72–92%; (c) PIDA, I_2_, K_2_CO_3_, CH_2_Cl_2_, 35 °C, 30 min–3 h, 45–79%; (d) BBr_3_, DCM, r.t., o/n, 52–71%.

Scheme 3 illustrates the synthesis of sultam derivatives of urolithin C with a benzyl group. The synthesis process involved Suzuki cross-coupling of **7** with appropriate boronic acids. This reaction led to the formation of biphenyl derivatives of benzyl-sulfonamides, namely **8a**–**h**, with yields ranging from 72% to 92%. These compounds were then prepared for cyclization in the subsequent reaction step.

For the cyclization process, a diacetoxyiodo benzene (PIDA)-mediated intramolecular sp^2^ C–H bond amination method was employed. This method was chosen due to its favorable reaction conditions, including a mild temperature of 35 °C and short reaction times ranging from 30 min to 3 h. As a result, the desired sultams, namely **14a**–**h**, were synthesized with moderate to excellent yields ranging from 45% to 79%. The synthesized compounds were subsequently purified using flash chromatography. For the deprotection step, BBr_3_ was utilized, resulting in the formation of benzylated compounds (**15a**–**h**) as the major products. These compounds were obtained with yields ranging from 52% to 71%.

**Scheme 4:**
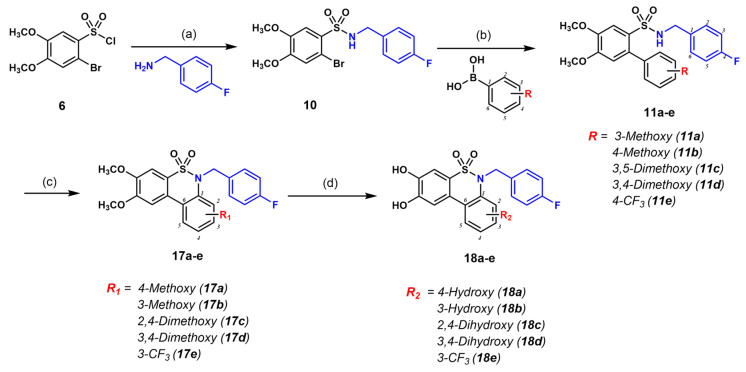
Synthesis of biaryl sulfonamides (with a fluorobenzyl group); reagents and conditions: (a) DIPEA, CH_2_Cl_2_, r.t., 1 h, 85%; (b) Pd (PPh_3_)_4_, toluene:EtOH:water/5:2:1, 120 °C, MW, 1 h, 67–88%; (c) PIDA, I_2_, K_2_CO_3_, CH_2_Cl_2_, 35 °C, 30 min–3 h, 55–70%; (d) BBr_3_, DCM, r.t., o/n, 55–68%.

Scheme 4 illustrates the synthesis of sultam derivatives of urolithin C with a fluorobenzyl group. The process started with sulfonyl chloride **6** reacting with fluorobenzylamine to produce sulfonamide **10**. Compound **10**, containing a fluorobenzylamine “handl”, underwent Suzuki cross-coupling with boronic acids to form biphenyl derivatives of benzyl-sulfonamides **11a**–**e**. These compounds were cyclized using PIDA to achieve sultams **17a**–**e** in moderate to excellent yields. Deprotection was achieved using BBr_3_, resulting in the formation of benzylated compounds (**18a**–**e**) as the major products. These compounds were obtained with yields ranging from 55% to 68%.

**Scheme 5:**
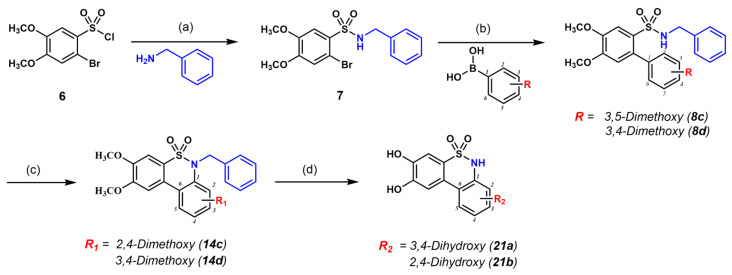
Synthesis of sultam derivatives of urolithin C (without benzyl group); reagents and conditions: (a) DIPEA, CH_2_Cl_2_, r.t., 1 h, 90%; (b) Pd(PPh_3_)_4_, toluene:EtOH:water/5:2:1, 120 °C, MW, 1 h; (c) PIDA, I_2_, K_2_CO_3_, CH_2_Cl_2_, 35 °C, 30 min–3 h; (d) BBr_3_, DCM, r.t., o/n, and MsOH, r.t., 1 h.

The synthesis of sultam derivatives of urolithin C (without a benzyl group) followed Scheme 5. Starting with sulfonyl chloride **6**, it reacted with benzylamine to yield sulfonamide **7**. Suzuki cross-coupling with boronic acids produced biphenyl derivatives **8c** and **8d** (82% and 92% yields). Cyclization using PIDA led to sultams **14d** and **14c** (69% and 55% yields), purified via flash chromatography. To achieve complete deprotection of the compounds, methanesulfonic acid was added to the BBr_3_-reaction mixture. This step yielded deprotected compounds, namely **21a** and **21b**, with yields of 50% and 95%, respectively. 

The structures of all compounds were elucidated using IR, ^1^H-, and ^13^C-NMR spectroscopy, as detailed in the experimental section.

The formation of biaryl sulfonamide and sultam analogs of urolithin C with benzyl and fluorobenzyl groups was confirmed using different spectroscopic techniques, taking sultam **14d** as a representative compound. ^1^H-NMR confirmed sultam formation by the disappearance of a biaryl sulfonamide **8d**-NH and an aromatic proton of ring C after the Suzuki cross-coupling reaction and the appearance of a singlet at δ 4.82 for the methylene proton, in addition to other methoxy protons and aromatic protons. Additionally, sultam **14d** was subjected to deprotection to generate **15d**, as confirmed by the disappearance of up-field singlets at δ 4.01, 3.95, and 3.67 -OCH_3_ protons and the appearance of down-field singlets of -OH protons at δ 9.98, 9.90, 9.46, 9.26, and other aromatic protons.

### 3.2. Screening Cell Permeable PKL Inhibitor and Validation of Therapeutic Effects on MAFLD

#### 3.2.1. Screening out Cell Permeable PKL Inhibitor **15e**

**Figure 5:**
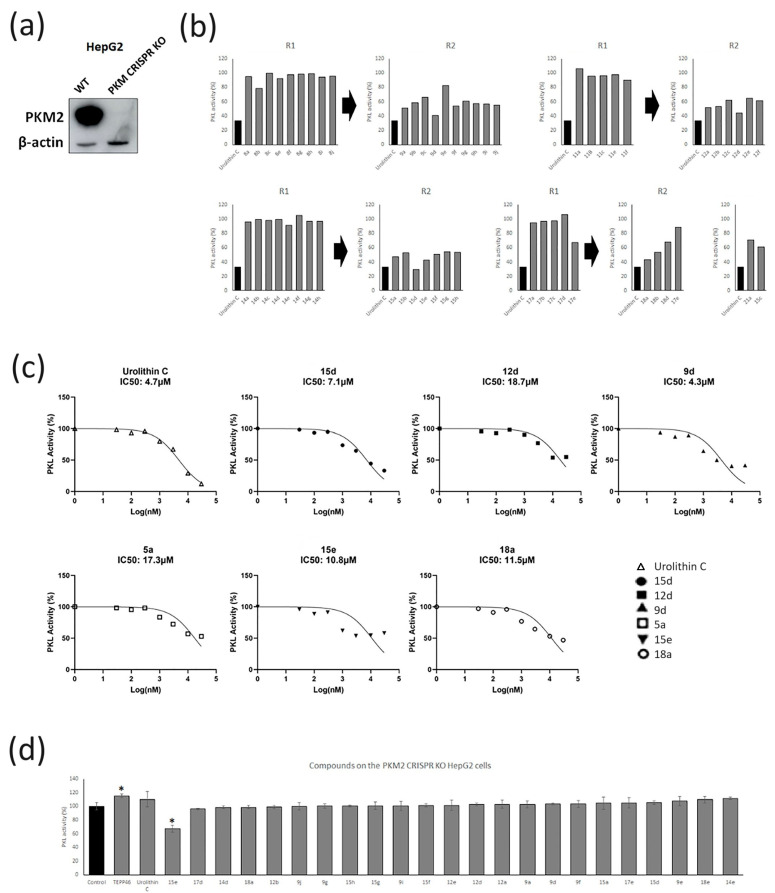
(**a**) PKM CRISPR knock-out HepG2 WT cells were designed. HepG2 PKM CRISPR KO cells have PKL only. (**b**) Pyruvate kinase activity assay on HepG2 KO cells (PKL only) protein lysate. Generated pyruvate (O.D. value at 570 nm) was calculated as a percentile (%). (**c**) IC_50_ value of PKL enzymatic activity inhibition for most active compounds. 30 µM, 10 µM, 3 µM, 1 µM, 300 nM, 30 nM, and untreated groups were tested on HepG2 KO cell lysate. (**d**) Pyruvate kinase activity assay on HepG2 KO cells after 20 µM compounds for 4 hr treatment. Generated pyruvate (O.D. value at 570 nm) was calculated as a percentile (%). Data are represented as mean ± SD, * *p* < 0.05, Student’s *t*-test.

PKL is specifically expressed in the liver. However, HepG2 wild-type (WT) cells, a hepatocellular carcinoma cell line, and the cell model used in this study express both PKL and PKM. To investigate PKL inhibitors, we developed PKM CRISPR knockout HepG2 (HepG2 KO) cells, which exclusively express PKL, as illustrated in Figure 5a. Western blot analysis confirmed the complete removal of PKM expression in the HepG2 cells. Treatment with urolithin C and its derivatives on HepG2 KO cell lysates at a concentration of 10 µM demonstrated their inhibitory effect on PKL, as assessed through a PK activity kinetic assay (Figure 5b and Supplementary Figure S1a). Urolithin C led to a 33.2% inhibition of PKL activity in the cell lysate. The six most active compounds were identified as **15d** (29.8% inhibition), **9d** (40.9%), **15e** (42.9%), **18a** (50.8%), **12d** (44.1%), and **15a** (47.6%). The enzymatic IC_50_ values of these compounds indicated a dose-dependent decrease in PKL activity within the protein lysate. The IC_50_ values were recorded as follows: urolithin C at 4.7 µM, **15d** at 7.1 µM, **12d** at 18.7 µM, **9d** at 4.3 µM, **15a** at 17.3 µM, **15e** at 10.8 µM, and **18a** at 11.5 µM, respectively. After identifying the active compounds through protein lysate screening, we tested their ability to inhibit PKL activity in a cell-permeable manner, as depicted in Figure 5c and Supplementary Figure S1b. Compounds were administered at 20 µM into the cell culture media for 4 h. The cells were then washed with PBS, directly lysed, and analyzed for PKL activity using a PK activity kinetic assay. TEPP46, a known activator of PK activity, increased PKL activity to 115.3% ± 2.5. Unlike urolithin C, which did not inhibit PKL activity in cells, Compound **15e** significantly reduced PKL activity in HepG2 KO cells by showing 67.1% ± 4.8 activity, showcasing its potential as a potent PKL inhibitor with cell permeability.

#### 3.2.2. Validation of Cell-Permeable PKL Inhibitor **15e**

**Figure 6:**
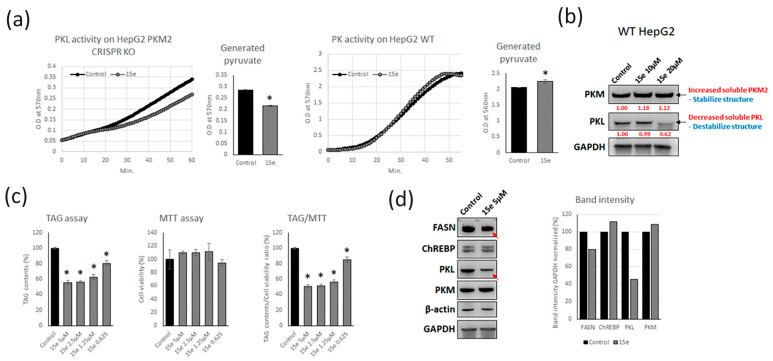
(**a**) Pyruvate kinase activity on cells. PK activities were tested on 20 µM **15e** for 4 h treated HepG2 CRISPR PKM KO cells (**left**) and HepG2 WT (**right**). The generated pyruvate during the assay was visualized in the histogram. (**b**) CETSA assay for PKM2 and PKL. **15e** 2 h treated HepG2 WT cells had heat shock at 60 °C for 5 min, and soluble proteins were analyzed using western blot. (**c**) Cell permeable PKL inhibitor **15e** was used to treat the HepG2 WT DNL steatosis model for one week at 5 µM, 2.5 µM, 1.25 µM, and 0.625 µM. After one week, TAG contents and cell viability were measured. (**d**) Western blot analysis for **15e** treated one-week HepG2 WT steatosis model. Compound **15e** was used to treat the HepG2 DNL steatosis model at a 5 µM concentration. The band intensity of DNL-involved steatosis proteins was measured. Arrows indicate FASN and PKL, which decreased protein expression. Data are represented as mean ± SD, * *p* < 0.05, Student’s *t*-test.

We focused on evaluating the efficacy of a promising compound, designated as **15e**, for its ability to inhibit the PKL isoform in a cell-permeable manner, specifically targeting HepG2 WT cells. HepG2 WT cells express both PKM and PKL isoforms. We assessed the PK activity in both HepG2 KO and WT cells under treatment of 20 µM for 4 h, as depicted in Figure 6a. The PK activity in HepG2 KO cells, which lack PKM expression, was significantly lower compared to the combined PKM and PKL activity in HepG2 WT cells. Untreated HepG2 KO cells exhibited a PK activity value of 0.35 at an optical density (O.D.) of 570 nm, whereas HepG2 WT cells showed a plateau curve at 2.5 O.D. 570 nm, indicating that PKM activity predominantly occurs in HepG2 WT cells. Interestingly, compound **15e** induced a 9% activation of PK activity in HepG2 WT cells (containing both PKM and PKL) but inhibited PK activity by 25% in HepG2 KO cells (containing only PKL). To further explore these differential effects on PKM and PKL, we conducted a Cellular Thermal Shift Assay (CETSA), as illustrated in Figure 6b. HepG2 WT cells treated with compound **15e** for 2 h were subjected to a 60 °C heat shock for 5 min. Western blot analysis revealed a 12% increase in PKM and a 38% decrease in PKL levels. These results suggest that **15e**’s differential effects on PKM and PKL could be attributed to its ability to stabilize the enzyme structure of PKM while destabilizing that of PKL. 

Additionally, we examined the therapeutic potential of **15e** for MAFLD by applying it to a HepG2 de novo lipogenic (DNL) steatosis model. This approach aimed to elucidate the compound’s effect on liver disease models, particularly in terms of lipid accumulation and metabolic regulation, thereby offering insights into its potential as a treatment for MAFLD [30]. 10 µM of compound **15e** showed high toxicity on a one-week DNL steatosis procedure (Supplementary Figure S1c). So, we tested **15e** in a dose-dependent manner against the DNL steatosis model at 5 µM, 2.5 µM, 1.25 µM, and 0.625 µM (Figure 6c,d). **15e** did not show cytotoxicity at 5 µM to 0.625 µM. However, compound **15e** decreased TAG contents markedly at 5 µM (55.6% ± 2.4), 2.5 µM (56.3% ± 1.9), 1.25 µM (62.7% ± 2.9), and 0.625 µM (80.4% ± 3.2). TAG contents that were normalized by cell viability (MTT assay) showed 5 µM (50.5% ± 2.2), 2.5 µM (51.2% ± 1.7), 1.25 µM (56.3% ± 2.6), and 0.625 µM (84.9% ± 3.3). We measured the protein levels of the enzymes in the DNL pathway, including fatty acid synthase (FASN), carbohydrate response element binding protein (ChREBP), PKL, and PKM2, using western blot analysis [31] (Figure 6d). Compound **15e** demonstrated a significant reduction in the expression levels of FASN by up to 79% and PKL by 45%. However, the protein levels of ChREBP and PKM remained unaffected. This specificity suggests that **15e** selectively targets the pathways associated with FASN and PKL without influencing the regulatory mechanisms or activity of ChREBP and PKM. Previous studies showed that inhibition of PKL activity reduces the protein level of FASN [32]. Hence, we observed that **15e** exhibits therapeutic potential for MAFLD by reducing the protein expression levels of FASN through the structural destabilization of PKL and subsequent decrease in its expression. This mechanism highlights **15e**’s ability to target key enzymatic pathways involved in lipid synthesis and accumulation, addressing the critical factors in the progression of MAFLD.

### 3.3. Computational Studies

To validate our rational design, we exposed compounds **9d**, **12d**, **15a**, **15e**, **15d**, and **18a**, demonstrating the most promising biological activity, and docked them against the allosteric site of PKL. Utilizing the crystal structure of the PKL complex with PDB code 7FS5 as our molecular modeling target, we performed molecular docking investigations [20]. The steadiness of the docking approach was confirmed through the re-docking of the native ligand using MOE, revealing consistent results with root mean square deviation (RMSD) values ≤ 1 Å. The docking simulations produced relative binding free energies ranging from −7.92 to −11.33 kcal mol^−1^, suggesting a wide range of expected binding affinities. Examination of the reference ligand indicated that catechol rings were positioned between the side chains of Phe38 residues, with molecules occupying both symmetrical slots across the dimer interface in the binding site. Notably, the docked molecules retained critical connections with Lys323, Tyr402, Glu409, and other significant contacts, including π-π interactions between the two Phe38 side chains. Notwithstanding these discoveries, the docking inquiries revealed numerous probable binding locations for these compounds on PKL.

**Figure 7:**
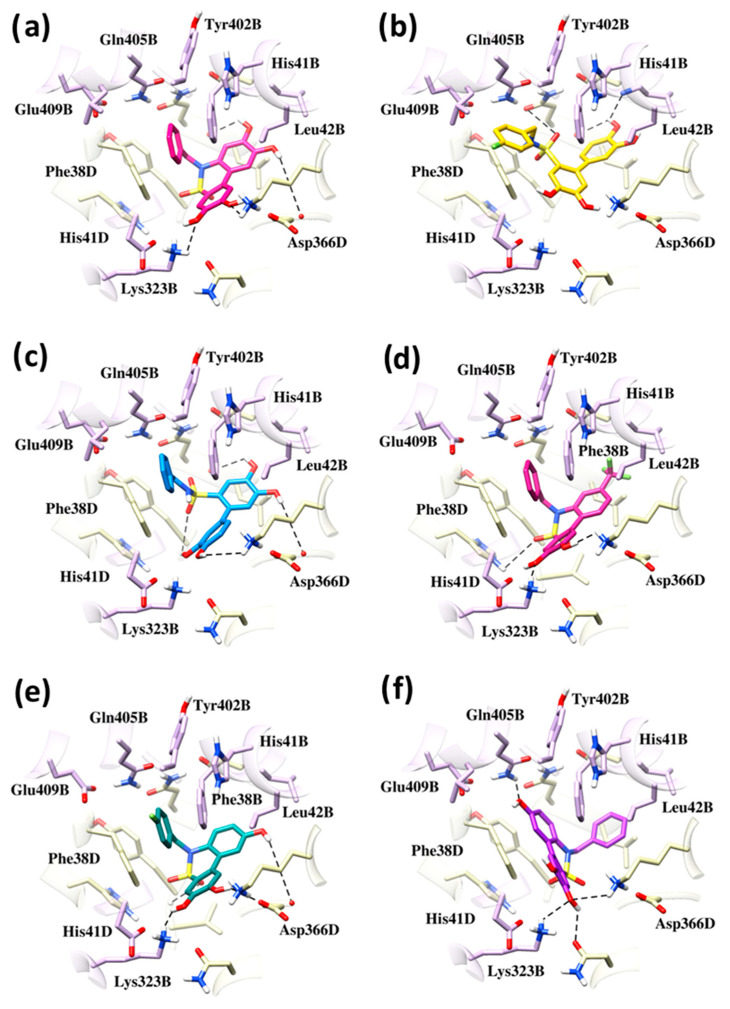

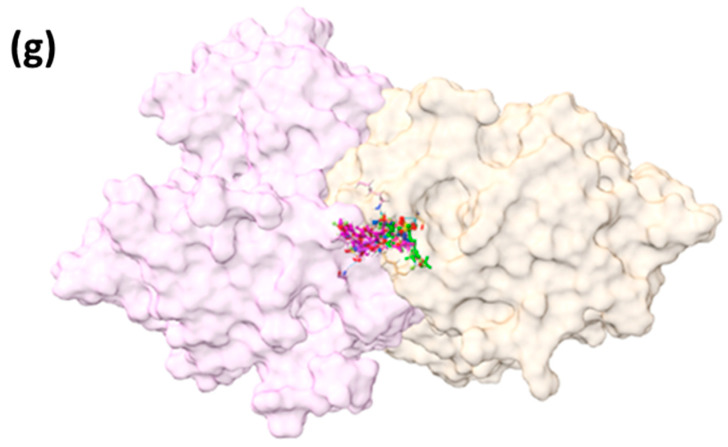
Protein-ligand interaction diagram of compounds (**a**) **15d**, (**b**) **12d**, (**c**) **9d**, (**d**) **15e**, (**e**) **18a**, and (**f**) **15a** bound to the allosteric interface of PKLR; (**g**) presenting the docking pose of the above-mentioned six urolithin D derivatives.

As shown in Figure 7a, the catechol ring of compound **15d** bounds to the interface binding pocket by forming multiple H-bond interactions with the main chains of Lys323, Asp366, Tyr402, and Gln405 of chain D and Ly323 of chain B at a distance of 3.4, 2.8, 3.0, 2.3, and 3.3 Å, respectively. The benzene ring of the ligand interacts with Phe38 of chains B and D through face-to-face π-stacking interactions. Additionally, compound **15d** also mediated hydrophobic interaction with Ph38 and Leu42 of chain B and Phe38 and Leu365 of chain D. Figure 7b depicts the binding mode of compound **12d**. The compound also exhibits hydrophobic contacts with Phe38, Leu42 of chain B, and Phe38, Leu365, and Leu406 of chain D. An essential feature of the ligand is its water-mediated interaction with Asp366. The benzene ring of the ligand is involved in face-to-face π-stacking interactions with Phe38 of chains B and D. The polar atoms of the ligand-mediated hydrogen bond with the side chains of Lys323 and Gln405 of chain D and Ph38 of chain B at a distance of 3.5, 3.0, and 2.9 Å, respectively. The plausible binding mode of compound **9d** is depicted in Figure 7c. Compound **9d** mediates hydrophobic contacts with Phe38, Leu365, and Leu406 of chain D. The benzene ring of the compound is sandwiched between Phe38 of chain B and chain D. The sulfonyl oxygen of the ligand-mediated hydrogen bond interaction with Tyr402 of chain D is at a distance of 3.2 Å, respectively. while the catechol ring interacts with Lys323, Asp366 and Gln405 of chain D at a distance of 2.9, 2.3, and 3.5 Å, respectively. The compound **15e** resides comfortably in the binding site, extending hydrophobic interactions with Phe38 and Leu42 of chain B, while Phe38, Leu306, and Leu402 of chain D (Figure 7d). The aromatic ring of Phe38 from both chains and Tyr402 from chain D mediates face-to-face and edge-to-face stacking interactions against the benzene ring of the ligand. The polar atoms of the ligand-mediated hydrogen bond with the side chains Lys323 of chain B and His41, Lys323, and Tyr402 of chain D at a distance of 1.8, 3.5, 3.4, and 2.5 Å, respectively. In the case of compound **18a**, the catechol ring was found to form numerous H-bond interactions with Lys323, Asp366 of chain D, and Lys323 of chain B at a distance of 2.6, 2.7, and 2.0 Å, respectively (Figure 7e). While the oxygen of hydroxyl and sulfonyl groups mediate H-bond interaction with Asp366 and Tyr402 of chain D at a distance of 3.0 and 2.3 Å, respectively. The docked poses for all the urolithin D derivatives are presented in Figure 7g.

The analysis of the top-ranked binding mode of compound **15a** highlights hydrophobic contacts as the most dominant interaction between the ligand and protein. As evident from Figure 7f, the ligand presents hydrophobic contacts with Leu42 and Gln405 of chain D, while Phe38 is parallel to the benzene ring of the ligand, reinforcing a face-to-face π-stacking interaction. The polar atoms of the ligand-mediated hydrogen bond with the side chains of Lys323 and Glu405 of chain B and Asn362 and Lys323 of chain D at a distance of 2.7, 3.4, 2.7, and 3.5 Å, respectively. Moreover, a water-mediated bridge connects the ligand with Asp366 and Tyr402 of chain B.

### 3.4. In Silico ADME Properties

The QikProp program was used to compute the following ten pharmacokinetic properties: aqua solubility, polar surface area, number of rotatable bonds, number of hydrogen bond donors or acceptors, caco-2 cell permeability, human serum albumin binding, blood/brain partition coefficient, and human oral absorption of the five active compounds. These properties were in addition to the in silico ADME properties (adsorption, distribution, metabolism, and excretion). Table 1 provides a summary of the computations’ outcomes. QPlogPo/w is used to estimate the octanol-water partition coefficient or lipophilicity; a greater QPlogPo/w value was associated with poor absorption. Table 1 clearly shows that the most effective chemicals, **15d**, **12d**, **9d**, **15e**, and **15a**, exhibit good pharmacokinetic profiles for all descriptors. Another important factor related to medication bioavailability is the polar surface area (PSA), which should not be greater than 140 Å. The target molecules’ PSA values fall between 85.144 and 132.578 Å. All compounds showed significant results in the aqua solubility parameter (QPlog S) and the Caco-2 cell permeability parameter (QPPcaco). The blood-brain barrier-crossing capacity of a medication, measured by the blood/brain partition co-efficient (QPlogBB) value, is within an acceptable range for all active molecules. Moreover, QPlogKhsa, or the human serum albumin binding coefficient, is another important component, and the compounds are expected to have values between −0.062 and 0.291. There may be a greater oral bioavailability of the active substances based on the estimated values of human oral absorption, which fall between 57.7 and 86.6%. It is important to note that the majority of the active compounds in the library of this study have good oral bioavailability, QPPcaco permeability, and QPlog S aqua solubility. To sum up, the silico ADME prediction of the active compounds may assist in the creation of new therapeutic candidates with advantageous pharmacokinetic properties.

### 3.5. Structure-Activity Relationship (SAR) Study

This study investigated the optimization of polyphenolic compound bioactivity and cell permeability. Initial attempts to combine polyphenol groups with sulfonamide for targeting PKL resulted in potent bioactivity in cell lysates but poor cell penetration. This was attributed to the sulfonamide group’s basic nature and the polyphenol’s hydrophilicity, hindering the lipophilicity and acidity necessary for cell permeability. Introducing fluorine atoms adjusted the ligand’s basicity towards a slightly acidic character, enhancing lipophilicity and cell penetration. This demonstrates the crucial role of molecular design in developing effective therapeutics that balance bioactivity with efficient cellular interaction.

Based on in vitro results, it has been found that the presence of the catechol moiety (ring **A**) is crucial for inhibiting the PKL enzyme. Computational studies have shown that it forms hydrogen bonds in the allosteric pocket of PKL. Additionally, the benzylated part (ring **D**) has been identified as an important area for enhancing activity through hydrophobic interactions. Replacement of lactone with a sultam structure has also been observed to improve activity by providing two oxygen atoms that increase interactions and appropriate conformational structures in the allosteric pocket. Finally, by making chemical modifications and incorporating electron-donating and electron-withdrawing groups in the aromatic ring (ring **C**), significant changes have been observed in PKL cell lysates and cell-based assays.

In this study, we evaluated the inhibitory activities of linear analogs (**9a**–**j** and **12a**–**f**) of biaryl sulfonamide and cyclic analogs (**15a**–**h** and **18a**–**e**) of sulfone containing urolithin C against the liver pyruvate kinase (PKL) enzyme in vitro.

**Figure 8:**
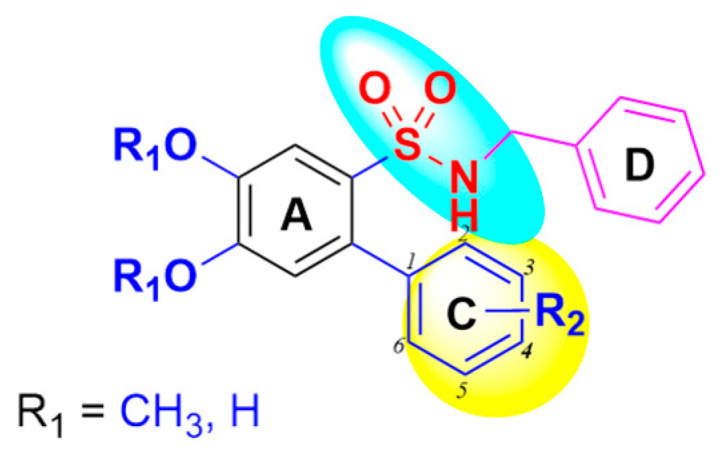
Structural representation of biaryl sulfonamide (linear) analogs for SAR.

Methylated biaryl sulfonamides (ring **A**, R1=CH_3_, and ring **D** = Benzyl group) showed no activity in the bioassay. However, when these compounds were demethylated and contained a benzyl group, their activity dramatically increased. The percentage of inhibition ranged between 19.0% and 60.0% for compounds **9a**–**j**. The presence of substituents at ring **C** (R_2_) also played a role in inhibitory activity, with promising results observed for different substituents. Among them, compound **9d** with 3,4-diOH exhibited the best inhibitory activity, with an IC_50_ value of 4.3 µM. This was even better than the reference compound urolithin C, which had an IC_50_ value of 4.7 µM (Figure 8).

Similarly, methylated biaryl sulfonamides (ring **A**, R1=CH_3_, and ring **D** = fluorobenzyl group) showed no activity in the bioassay. However, when these compounds were demethylated and contained a fluorobenzyl group, their activity significantly increased. The percentage of inhibition ranged between 35.0% and 55.0% for compounds **12a**–**f**. The presence of substituents at ring **C** (R_2_) also influenced inhibitory activity, with promising results observed for different substituents. Among them, compound **12d** with 3,4-diOH exhibited the best inhibitory activity, with an IC_50_ value of 18.7 µM (Figure 8).

**Figure 9:**
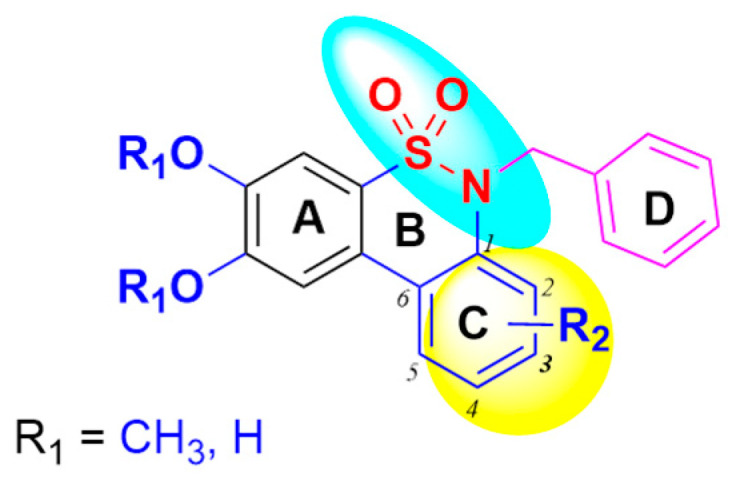
Structural representation of Sultam (cyclic) analogs of urolithic C for SAR.

Benzylated sultam analogs (Figure 9), with a methyl group (R1=CH_3_) on ring **A** and different functionalities on ring **C**, did not exhibit inhibition. However, when the benzylated sultam analogs (Figure 9) were demethylated and had a hydrogen atom (R1=H) on ring **A**, along with various functionalities on ring C, they showed improved inhibitory activity. After evaluating the inhibition rates of seven compounds (**15d**, **15e**, **15a**, **15f**, **15b**, **15h**, and **15g**) based on substituents at ring **C** (R_2_ = 3,4-OH > 3-CF_3_ > 4-OH > 3-OH, 4-F > 3-OH > 3-OCF_3_ > 3-OCH_3_,4-CF_3_), we observed increased activity with inhibition rates ranging from 46.0% to 70.0% (Figure 9). Among them, compounds **15d**, **15e**, and **15a** exhibited good inhibitory activity.

Fluorobenzylated sultam analogs (Figure 9) with a methyl group (R1=CH_3_) on ring A and different functionalities on ring **C** did not show inhibition. However, when the benzylated sultam analogs (Figure 9) were demethylated and had a hydrogen atom (R1=H) on ring **A**, along with various functionalities on ring **C**, they displayed better inhibitory activity. After assessing the inhibition rates of compounds **18a**, **18b**, **18d**, and **18e** based on substituents at ring **C** (R_2_ = 4-OH > 3-OH > 3,4-OH > 3-CF_3_), we identified increased activity with inhibition rates ranging from 11.0% to 57.0% (Figure 9). Among them, compound **18a** exhibited good inhibitory activity.

**Figure 10:**
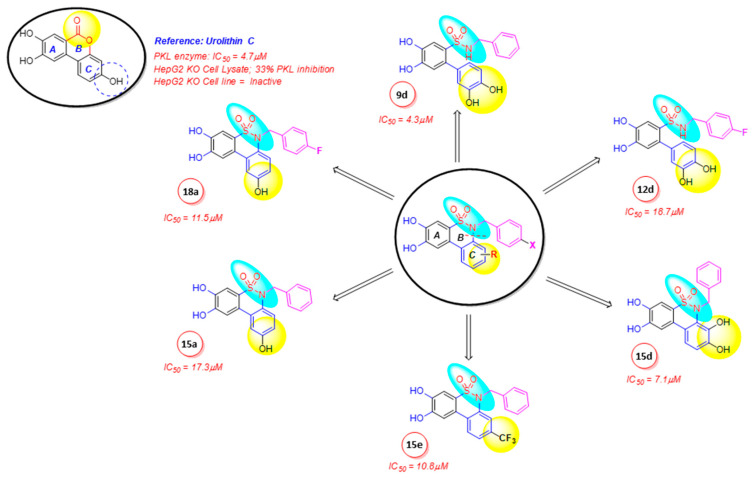
Shows the SAR study, identifying the pharmacophoric features of urolithin C and newly designed molecules, along with their IC_50_ values.

Based on the inhibition data, the following active compounds were selected for enzymatic IC_50_ value determination: **9d**, **12d**, **15d**, **15d**, **15e**, **18a**, and **15a** (Figure 10). Compound **12d**, a biaryl sulfonamide with 3,4-diOH, exhibited activity with an IC_50_ value of 18.7 µM. Compound **15a** with 4-OH displayed an IC_50_ value of 17.3 µM, while compound 18a with 3-OH showed an IC_50_ value of 11.5 µM. Sultam derivative **15e** demonstrated an IC_50_ value of 10.8 µM, and sultam **15d** with 3,4-diOH exhibited good inhibitory activity with an IC_50_ value of 7.1 µM. Compound **9d** with 3,4-diOH displayed the best inhibitory activity with an IC_50_ value of 4.3 µM, outperforming the reference compound urolithin C with an IC_50_ value of 4.7 µM (Figure 10). Notably, sultams with ring C substitution of -OH groups with hydrophilic character were most crucial for PKL enzyme inhibition. Additionally, the -CF_3_ group at the 3-position on ring **C** in compound **15e** displayed good activity due to the unique chemical property of the trifluoromethyl group. It enhances drug potency by lowering the pKa of the cyclic carbamate and forming a crucial hydrogen bonding interaction with the protein.

**Figure 11:**
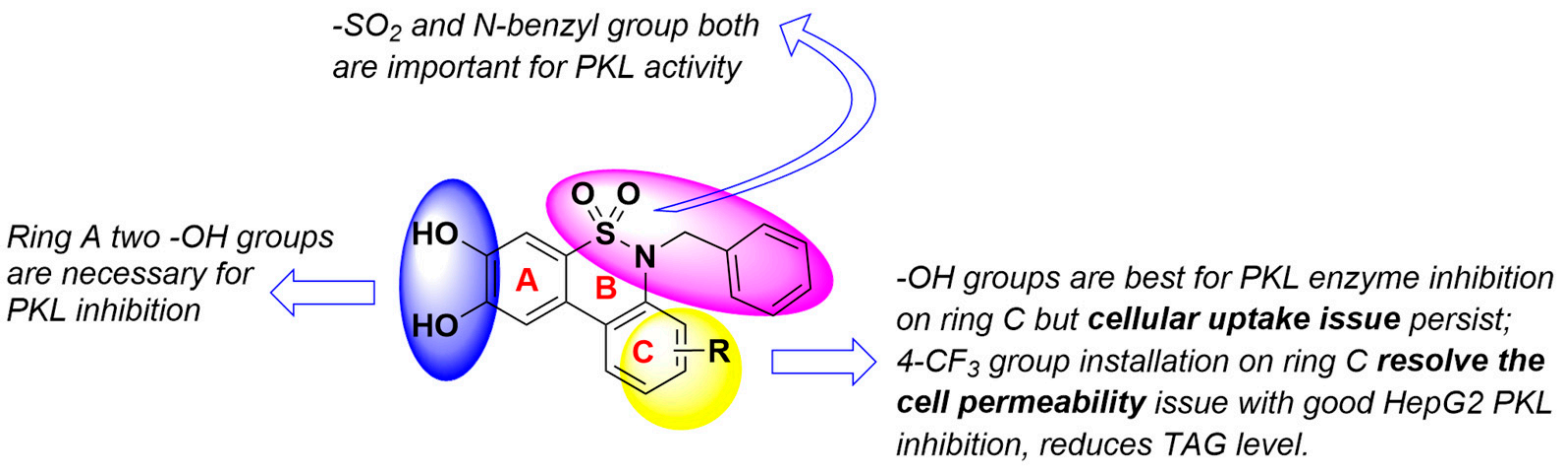
Presents a concise summary of the observed structure-activity relationship (SAR) of the PKL inhibitors investigated in this study.

After evaluating the inhibitory effects of various compounds on protein lysate, we selected **23** sultam compounds to determine their ability to inhibit PKL in a whole-cell assay, taking into consideration their cell permeability. Regrettably, the majority of benzylated sultam compounds did not exhibit PKL inhibition in the whole-cell assay. However, it is worth noting that compound **15e** demonstrated significant PKL inhibition in the whole-cell assay, effectively resolving the issue of cell permeability (Figure 5d). The significance of polarity and hydrophilicity in achieving effective inhibitory activity against PKL has been acknowledged. Extensive studies on the modification sites and PKL inhibition data have revealed that enhancing the inhibitory activity, cell permeability, and drug-like properties can be achieved by functionalizing -OH groups on ring C with -CF_3_, as shown in Figure 11. 

### 3.6. In Vitro Evaluation of Cell-Permeable Allosteric PKL Inhibitors for the Treatment of MAFLD

MAFLD is a prevalent health concern characterized by the accumulation of liver fat and impacting a significant portion of the global population. The frequency of MAFLD is rapidly rising, becoming a prominent global health issue due to its connection with obesity, metabolic syndrome, and diabetes [33]. Hence, there is a need for more targeted and effective drugs for MAFLD treatment. PK plays a pivotal role as an enzyme in glycolysis, which is the metabolic pathway responsible for the final step of converting glucose into pyruvate [34]. Therefore, dysregulation of PK activity has been implicated in several diseases, making it an attractive target for drug discovery efforts [35]. Significantly, the PKL isoform exhibits substantial expression in the liver, indicating that cell-permeable allosteric inhibitors targeting these enzymes could be effectively utilized for the prevention or treatment of MAFLD [7]. 

Ellagic and its natural metabolites, urolithin C and urolithin D, have been identified in the literature as allosteric inhibitors of PKL [21]. In liver cell lysate, they display inhibition rates of 21%, 33%, and 37% at a concentration of 10 μM, respectively [20]. Although ellagic acid and its metabolites have shown promising allosteric inhibition of PKL, their limited permeability across biological barriers poses challenges to their therapeutic development. Various studies have investigated the ability of ellagic acid and its metabolites to penetrate cellular membranes and biological barriers, including the intestinal epithelium and blood-brain barrier (BBB), to elucidate its pharmacokinetic profile and potential therapeutic applications such as anticancer effects, support for microbiota health, and neuroprotection [36,37,38,39]. To the best of our knowledge, no study has reported the efficacy of allosteric inhibitors of PKL containing ellagic acid and its derivatives in intact cells. This present study is the first to validate the efficacy of sulfone-based urolithins as cell-permeable allosteric inhibitors of PKL. In this study, **15e** displayed promising outcomes by significantly reducing PKL expression levels, lowering TAG content, and exhibiting effective cellular uptake. Furthermore, it was revealed that the SAR analysis identified the sulfone moiety and -OH groups as crucial for PKL inhibition, with the addition of -CF_3_ contributing to cell penetration. The discovery of cell-permeable allosteric inhibitors of PKL such as **15e** opens new avenues for therapeutic intervention in conditions characterized by dysregulated glycolysis. Sulfone-based urolithins might present a promising starting point with their ability to selectively target PKL and modulate its activity. This holds particular significance in the context of metabolic disorders, such as MAFLD. 

### 3.7. Metabolic Pathways and Molecular Mechanisms Implicated in MAFLD and PKM2-Related Diseases

MAFLD includes nonalcoholic fatty liver (NAFL) and nonalcoholic steatohepatitis (NASH), which can develop cirrhosis and hepatocellular carcinoma (HCC) [40]. An imbalance in the hepatic fat metabolism (uptake from circulating lipids or de novo lipogenesis) causes MAFLD. The liver absorbs excess fatty acid from blood generated and released by the adipose tissue and synthesizes fatty acids from glucose via the DNL pathway [41]. These two pathways are simultaneously implicated in the human body. Fatty acids synthesized through de novo lipogenesis are significantly elevated in MAFLD patients, and DNL is considered the major cause of MAFLD [42]. Kim et al. introduced the HepG2 DNL steatosis model using insulin and the LXR agonist T0901317 [30]. Insulin and T0901317 increased SREBP-1c (hyperinsulinemia-induced transcription factor), ChREBP (hyperglycemia-induced transcription factor), FASN, and ACACA. In a recent study, JNK-IN-5A, which systems biology predicted compounds that decrease PKL expression, showed reduced TAG and the entire DNL pathway protein, including SREPB-1c and ChREBP [30]. **15e**, which was designed for PKL inhibitors, also decreased PKL protein expression levels and FASN but did not decrease one of the main DNL pathway transcription factors, ChREBP. It can be translated that **15e** specifically binds and inhibits PKL and leads to DNL inhibition, including FASN protein expression, as previously studied [30]. This is why the ChREBP transcription factor was not affected. However, we strongly believe that specific PKLs targeting compound **15e** may have a higher potential to discover new drugs.

PKM2 is one of the most well-known pyruvate kinases that control cell energy metabolism. PKM2 can exist in monomer, dimer, and tetramer forms [43]. The monomer and dimer forms of PKM2 show lower pyruvate kinase activity than the tetramer form. The mono-dimer form of PKM2 can be localized to the nucleus, works as a transcription factor, and leads the destination of pyruvate to lactate in glycolysis [44]. In contrast, the tetramer form of PKM2 has higher kinase activity and leads pyruvate to oxidative phosphorylation [45,46]. The Warburg effect is well known for manipulating cellular metabolism [47]. Cancer cells are addicted to glycolysis for energy generation rather than oxidative phosphorylation, even after recovering from hypoxia. Many studies showed that the PKM2 monomer and dimer form have a major role in the Warburg effect in cancer and accelerate tumorigenesis [46,48]. This increased lactate generation was also reported in NAFLD [49]. Our hit compound **15e** showed increased PK activity in HepG2 WT cells and decreased PK activity in HepG2 PKM2 CRISPR KO cells. **15e** also showed structural stabilization of PKM2 but destabilization of PKL in the CETSA assay. These results can be translated as **15e** activating PKM2 via intact tetramerization and inhibiting structural PKL destabilization. This effect may also show therapeutic effects on many other diseases related to PKM2 tetramerization, including kidney fibrosis [3], inflammation [50], and dilated cardiomyopathy [51].

## 4. Material and Methods

### 4.1. Chemistry General Methods 

#### General Information

All reactions were performed with oven-dried glassware and under an inert atmosphere (nitrogen), unless otherwise stated. All reagents were obtained from a commercial supplier, Sigma-Aldrich, and used without further purification. Dried solvents were obtained from a commercial supplier, Sigma Aldrich. Organic solutions were concentrated under reduced pressure on a Heidolph rotary evaporator. Reactions were monitored by LC-MS (Thermo Fisher TSQ Series, Athena C_18_-WP,100 Å, 2.1 × 50 mm, 3 μm, Thermo Fisher Scientitic, Waltham, MA USA 0245); water: MeOH (0.01 formic acid) or by thin-layer chromatography (TLC), and TLC analysis was performed using silica gel pre-coated aluminum plates (Kieselgel 60, 254, E. Merck, Darmstadt, 64293, Germany). The chromatograms were visualized using ultraviolet light (254 and 366 nm) and stained with vanillin dips or an aqueous potassium permanganate solution. ^1^H NMR spectra were recorded on an Avance Bruker 400 and 500 MHz spectrometer in CDCl_3_, CD_3_OD, and DMSO-d_6_. ^13^C NMR spectra were recorded in deuterated solvents on a Bruker spectrometer at 126 MHz, with the central peak of the deuterated solvent as the internal standard. The ^1^H NMR spectra are reported as δ/ppm downfield from tetramethyl silane (multiplicity, number of protons, coupling constant J/Hz). The ^13^C NMR spectra are reported as δ/ppm. All chemical shifts are reported in parts per million (ppm) relative to the residual solvent peak. The following abbreviations are used to denote signal patterns: (s) singlet, (d) doublet, (t) triplet, (q) quartet, (m) multiplet, and (br) broad, unless otherwise noted. Flash-column chromatography was performed on the PuriFlash XS 520 Plus Flash chromatography system (Interchim) with a built-in UV detector, ELSD detector, and fraction collector with Interchim silica gel columns.

General procedure A: sulfonamide preparation. 2-bromo-4,5-dimethoxybenzene-1-sulfonyl chloride **2** (1.0 eq.) was added to the stirred solution of corresponding amine (1.2 eq.) and DIPEA in dry DCM (20 mL), cooled to 0 °C. The reaction was allowed to warm to r.t. and stirred until complete consumption of the starting material was determined by TLC (Hexane:EtOAc/3:2), 1–2 h. The reaction is quenched with water (20 mL) and the crude product is extracted with DCM (3 × 20 mL). The combined organic phases were washed with 1 M HCl (3 × 60 mL) and brine (3 × 60 mL), and then dried over anhydrous Na_2_SO_4_. The solvent was removed under reduced pressure to afford crude sulfonamides, which were purified by trituration with methanol or purified by flash chromatography using hexane or EtOAc (gradient) to afford sulfonamides.

General procedure B: cross-coupling reaction. Biaryl sulfonamides were prepared according to the slightly modified procedure described. Sulfonamides (1.0 eq.), appropriate boronic acid (1.5 eq.), potassium carbonate (4.0 eq.), and Pd (PPh_3_)_4_ (0.03 eq.) were suspended in the mixture of toluene, ethanol, and water (5:2:1) (13 mL). A sequential application of N_2_ flow and vacuum was used to degas the mixture before heating in a MW reactor at 120 °C for 1 h. The crude product was extracted with ethyl acetate (3 × 30 mL). The organic phase was dried over sodium sulfate. The mixture was then filtered through the bed of celite, and the solvent was removed under reduced pressure. The residue was purified by flash chromatography using Hexane/EtOAc (gradient) to afford the biaryl sulfonamides.

General procedure C: cyclization of secondary sulfonamide. Cyclization was achieved according to the procedure described in the literature [52]. Appropriate sulfonamide (1 eq.), PIDA (1.10 eq.), I2 (1.10 eq.), and K_2_CO_3_ (1.50 eq.) were suspended in DCM (20 mL). The dark red solution was stirred at 35 °C for 3 h, until almost complete conversion was determined by TLC (3% EtOAc in DCM). The reaction is quenched with sat. aq. Na_2_S_2_O_5_, and the mixture is stirred at the r.t. until the discoloration of the solution occurs. The crude product was extracted with DCM, purified via flash chromatography (DCM/EtOAc, gradient, 0–1% EtOAc), and triturated with MeOH to afford sultams.

General procedure D: Deprotection of Methoxy groups. To a solution of appropriately protected compound (1 eq.) cooled to 0 °C in DCM (5–10 mL) was added BBr_3_ (1 M in DCM, 5 eq. per methoxy group). The reaction was allowed to warm up to room temperature and stirred for 3–12 h. LC/MS analysis revealed complete consumption of the starting material and the formation of N-benzyl or N-fluorobenzyl sultam as the main product, along with a small to medium amount of corresponding debenzylated sultam. The reaction is cooled to 0 °C and quenched with water and a mixture extracted with diethyl ether (3 × 50 mL). The combined organic phases were dried over Na_2_SO_4_, evaporated, and crude was purified via a reverse phase column to afford a compound.

General procedure E: Deprotection of Benzyl groups. To achieve complete cleavage of the benzyl group, 1 mL of methanesulfonic acid was added to the mixture, and the reaction was stirred for one hour at room temperature. The reaction is quenched with water (50 mL) and the crude product is extracted with EtOAc (3 × 50 mL). The combined organic phases were dried over Na_2_SO_4_ and evaporated. Crude was purified via a reverse-phase column to afford the compound.

General Procedure for the Synthesis of 2-bromo-4,5-dimethoxybenzenesulfonyl chloride **(2)**:

4-Bromoveratrole **1** (4.0 mL, 27.8 mmol) was added dropwise over 20 min. to a well-stirred flask containing chlorosulfonic acid (8 mL) cooled to 0 °C. The black mixture was then stirred for a further 30 min before cautious addition to ice (150 mL). After melting, DCM (100 mL) was added, and the phases were separated. The aqueous phase was extracted with DCM (100 mL), and the combination of organic phases was dried over MgSO_4_. Filtration and solvent removal under reduced pressure gave sulfonyl chloride **2** (7.17 g, 82%), which was further purified by recrystallization from diethyl ether and obtained white crystals (7.17 g, 82%). M.P: 102–104 °C; ^1^H-NMR (400 MHz, CDCl_3_): 7.60 (s, 1H, Ar), 7.22 (s, 1H, Ar), 3.98 (s, 3H, CH_3_), 3.94 (s, 3H, CH_3_); ^13^C-NMR (100 MHz, CDCl_3_): 154.3, 148.1, 134.9, 117.9, 113.3, 112.8, 56.9, 56.7.

*Synthesis of N-benzyl-2-bromo-4,5-dimethoxybenzenesulfonamide* **(7)**

**6** (10.0 g, 31.69 mmol) was added to a solution of benzylamine (4.15 mL, 38.03 mmol) and DIPEA (16.45 mL, 95.07 mmol) in DCM (30 mL) and cooled to 0 °C. The reaction was allowed to warm to room temperature and stirred for 6 h until complete conversion of the starting material had been confirmed by TLC (3% EtOAc in DCM). The reaction is quenched with water (30 mL). The crude product was extracted with DCM (3 × 30 mL). The combined organic phases were washed with 1 M HCl (3 × 50 mL), dried over Na_2_SO_4,_ and evaporated to obtain the white solid **7** (10.7 g, 90%). M.P: 108–109 °C, ^1^H NMR (400 MHz, DMSO-*d*_6_) δ 8.24 (s, 1H, NH), 7.38 (s, 1H, Ar), 7.26 (s, 1H, Ar), 7.25–7.15 (m, 5H, Ar), 4.08 (s, 2H, CH_2_), 3.84 (s, 3H, CH_3_), 3.76 (s, 3H, CH_3_); ^13^C NMR (101 MHz, DMSO-*d*_6_) δ 151.7, 147.3, 137.6, 131.5, 128.0, 127.6, 127.0, 117.4, 113.3, 110.6, 56.3(OCH_3_), 55.8(OCH_3_), 46.1(CH_2_); IR (cm^−1^) v = 3345, 3036, 1509, 1139, 839, 706; LCMS *m*/*z*: [M + 2]^+^, found 388.19. C_15_H_16_BrNO_4_S requires 386.26.

General procedure for the synthesis of biaryl sulfonamides **(cross-coupling)**:

Biaryl sulfonamides were prepared according to the slightly modified described procedure. Compound **7** (1.0 eq.), appropriate boronic acid (1.5 eq.), potassium carbonate (4.0 eq.), and Pd (PPh_3_)_4_ (0.03 eq.) were suspended in the mixture of toluene, ethanol and water (5:2:1) (13 mL). A sequential application of N_2_ flow and vacuum was used to degas the mixture before heating in a MW reactor at 120 °C for 1 h. The crude product was extracted with ethyl acetate (3 × 30 mL). The organic phase was dried over sodium sulfate. The mixture was then filtered through the bed of celite, and the solvent was removed under reduced pressure. The residue was purified by flash chromatography using Hexane/EtOAc (gradient) to afford the biaryl sulfonamides.

*Synthesis of N-benzyl-3′,4,5-trimethoxy-[1,1′-biphenyl]-2-sulfonamide* **(8a)**:

**7** (400 mg, 1.03 mmol), 3-Methoxybenzeneboronic acid (334 mg, 1.54 mmol), K_2_CO_3_ (560 g, 4.12 mmol), and Pd (PPh_3_)_4_ (34 mg, 0.03 mmol) were used according to the general procedure **B** to afford compound **8a** as a white solid (80%). M.P: 119–120 °C, ^1^H NMR (500 MHz, DMSO-*d*_6_) δ 7.65 (t, *J* = 6.3 Hz, 1H), 7.44 (s, 1H), 7.27 (m, 3H), 7.20 (m, 3H), 6.94 (m, 3H), 6.82 (s, 1H), 5.75 (s, 1H), 3.87 (d, *J* = 6.2 Hz, 2H), 3.82 (d, *J* = 7.5 Hz, 6H), 3.75 (s, 3H); ^13^C NMR (126 MHz, DMSO-*d*_6_) δ 158.80, 151.05, 147.79, 141.46, 138.40, 134.50, 131.13, 129.00, 128.64, 128.07, 127.56, 122.37, 115.79, 115.55, 113.27, 111.76, 56.36, 56.33, 55.43, 46.51; IR (cm^−1^) v = 3399, 3096, 1507, 1039, 833; LCMS *m*/*z*: [M + H]^+^, found 412.41. C_22_H_21_NO_5_S requires 411.47.

*Synthesis of N-benzyl-4,4′,5-trimethoxy-[1,1′-biphenyl]-2-sulfonamide* **(8b)**:

**7** (300 mg, 0.58 mmol), 4-Methoxybenzeneboronic acid (170 mg, 1.16 mmol), K_2_CO_3_ (400 mg, 2.9 mmol), and Pd (PPh_3_)_4_ (34 mg, 0.02 mmol) were used according to the general procedure **B** to afford compound **8b** as a white solid (74%). M.P: 163–164 °C, ^1^H NMR (500 MHz, DMSO-*d*_6_) δ 7.57 (t, *J* = 6.1 Hz, 1H), 7.43 (s, 1H), 7.30 (m, 2H), 7.26 (dd, *J* = 1.2, 7.1 Hz, 2H), 7.23 (m, 1H), 7.18 (m, 2H), 6.93 (m, 2H), 6.78 (s, 1H), 3.85 (d, *J* = 5.8 Hz, 2H), 3.82 (s, 3H), 3.80 (s, 3H), 3.79 (s, 3H); ^13^C NMR (126 MHz, DMSO-*d*_6_) δ 159.04, 151.06, 147.54, 138.44, 134.54, 132.36, 131.22, 131.13, 128.62, 128.07, 127.54, 116.15, 115.86, 115.05, 113.41, 111.83, 56.31, 55.56, 46.47; IR (cm^−1^) v = 3370, 3013, 2984, 1084, 874; LCMS *m*/*z*: [M + H]^+^, found 414.46. C_22_H_23_NO_5_S requires 413.49.

*Synthesis of N-benzyl-3′,4,5,5′-tetramethoxy-[1,1′-biphenyl]-2-sulfonamide* **(8c)**:

**7** (500 g, 1.29 mmol), 3,5-Dimethoxybenzeneboronic acid (460 mg, 2.5 mmol), K_2_CO_3_ (890 mg, 6.45 mmol), and Pd (PPh_3_)_4_ (50 mg, 0.05 mmol) were used according to the general procedure **B** to afford compound **8c** as a white solid (82%). M.P: 167–168 °C, ^1^H NMR (500 MHz, DMSO-*d*_6_) δ 7.63 (t, *J* = 6.3 Hz, 1H), 7.44 (s, 1H), 7.27 (m, 2H), 7.20 (m, 3H), 6.84 (s, 1H), 6.55 (d, *J* = 2.3 Hz, 2H), 6.49 (t, *J* = 2.3 Hz, 1H), 3.89 (d, *J* = 6.3 Hz, 2H), 3.83 (s, 3H), 3.81 (s, 3H), 3.74 (s, 6H); ^13^C NMR (126 MHz, DMSO-*d*_6_) δ 161.60, 159.96, 159.60, 151.05, 147.80, 141.93, 138.37, 134.48, 131.01, 128.65, 128.08, 127.58, 115.42, 111.78, 108.41, 99.69, 94.39, 91.93, 56.38, 56.34, 55.59, 55.38, 46.55; IR (cm^−1^) v = 3335, 3002, 2986, 1071, 745; LCMS *m*/*z*: [M + H]^+^, found 444.21. C_23_H_25_NO_6_S requires 443.51.

*Synthesis of N-benzyl-3′,4,4′,5-tetramethoxy-[1,1′-biphenyl]-2-sulfonamide* **(8d)**:

**7** (500 mg, 1.29 mmol), 3,4-Dimethoxybenzeneboronic acid (350 mg, 1.93 mmol), K_2_CO_3_ (710 mg, 5.16 mmol), and Pd (PPh_3_)_4_ (43 mg, 0.03 mmol) were used according to the general procedure **B** to afford compound **8d** as a white viscous liquid (92%). ^1^H NMR (400 MHz, DMSO-*d*_6_) δ 7.50 (t, *J* = 6.3 Hz, 1H, NH), 7.46 (s, 1H, Ar), 7.30–7.15 (m, 5H, Ar), 7.01 (d, *J* = 2.0 Hz, 1H, Ar), 6.96 (d, *J* = 8.3 Hz, 1H, Ar), 6.92 (dd, *J* = 8.2, 2.0 Hz, 1H, Ar), 6.84 (s, 1H, Ar), 3.86 (d, *J* = 6.2 Hz, 2H, CH_2_), 3.83 (s, 3H, CH_3_), 3.81 (s, 3H, CH_3_), 3.78 (s, 3H, CH_3_), 3.73 (s, 3H, CH_3_); ^13^C NMR (101 MHz, DMSO-*d*_6_) δ 150.6, 148.2, 147.6, 147.1, 137.9, 134.2, 132.0, 130.6, 128.2, 127.6, 127.1, 121.8, 115.4, 113.7, 111.5, 111.0, 55.9(OCH_3_), 55.9(OCH_3_), 55.5(OCH_3_), 55.4(OCH_3_), 46.0(CH_2_); IR (cm^−1^) v = 3387, 3016, 2979, 1114, 867; LCMS *m*/*z*: [M + H]^+^, found 444.11. C_23_H_25_NO_6_S requires 443.14.

*Synthesis of N-benzyl-4,5-dimethoxy-4′-(trifluoromethyl)-[1,1′-biphenyl]-2-sulfonamide* **(8e)**:

**7** (500 mg, 1.29 mmol), 4-Trifluoromethylbenzeneboronic acid (293 mg, 1.93 mmol), K_2_CO_3_ (713 mg, 5.16 mmol), and Pd (PPh_3_)_4_ (57 mg, 0.5 mmol) were used according to the general procedure **B** to afford compound **8e** as a white solid (72%). M.P: 147–148 °C, ^1^H NMR (500 MHz, DMSO-*d*_6_) δ 7.93 (t, *J* = 6.3 Hz, 1H), 7.73 (d, *J* = 8.1 Hz, 2H), 7.57 (d, *J* = 8.0 Hz, 2H), 7.45 (s, 1H), 7.23 (m, 5H), 6.86 (s, 1H), 3.92 (d, *J* = 6.2 Hz, 2H), 3.82 (d, *J* = 1.9 Hz, 6H); ^13^C NMR (126 MHz, DMSO-*d*_6_) δ 151.17, 148.24, 144.50, 138.27, 133.10, 131.20, 130.79, 128.63, 128.10, 128.07, 127.59, 124.79, 124.76, 124.73, 115.37, 111.75, 56.45, 56.34, 46.46; IR (cm^−1^) v = 3370, 3006, 2990, 1241, 1147, 842; LCMS *m*/*z*: [M + H]^+^, found 452.41. C_22_H_20_F_3_NO_4_S requires 451.46.

*Synthesis N-benzyl-4,5-dimethoxy-3′,5′-bis(trifluoromethyl)-[1,1′-biphenyl]-2-sulfonamide* **(8f)**:

**7** (600 g, 1.55 mmol), 3,5-Ditrifluoromethylbenzeneboronic acid (700 mg, 3.10 mmol), K_2_CO_3_ (850 mg, 6.2 mmol), and Pd (PPh_3_)_4_ (80 mg, 0.07 mmol) were used according to the general procedure **B** to afford compound **8f** as a white solid (85%). M.P: 158–159 °C, ^1^H NMR (500 MHz, Chloroform-*d*) δ 7.86 (s, 1H), 7.73 (d, *J* = 1.6 Hz, 2H), 7.64 (s, 1H), 7.24 (dd, *J* = 2.1, 5.1 Hz, 3H), 7.01–6.94 (m, 2H), 6.66 (s, 1H), 3.99 (s, 3H), 3.95 (d, *J* = 5.9 Hz, 2H), 3.93 (s, 3H).^13^C NMR (126 MHz, CDCl_3_) δ 151.79, 148.57, 140.88, 135.63, 131.27, 131.23, 130.96, 130.44, 130.09, 130.06, 128.82 (2CH), 128.21, 127.99 (2CH), 124.19, 122.01, 121.97, 114.45, 112.70, 56.52, 56.48, 47.25; IR (cm^−1^) v = 3370, 3018, 2995, 1220, 1069, 793, 686; LCMS *m*/*z*: [M + H]^+^, found 520.41. C_23_H_19_F_6_NO_4_S requires 519.46.

*Synthesis N-benzyl-3′-fluoro-4,4′,5-trimethoxy-[1,1′-biphenyl]-2-sulfonamide* **(8g)**:

**7** (600 g, 1.55 mmol), (3-fluoro-4-methoxyphenyl) boronic acid (520 mg, 3.1 mmol), K_2_CO_3_ (850 mg, 6.2 mmol), and Pd (PPh_3_)_4_ (80 mg, 0.07 mmol) were used according to the general procedure **B** to afford compound **8g** as a white solid (78%). M.P: 164–165 °C, ^1^H NMR (500 MHz, Chloroform-*d*) δ 7.59 (s, 1H), 7.22–7.16 (m, 3H), 7.13–7.08 (m, 1H), 6.99 (dd, *J* = 2.9, 6.6 Hz, 3H), 6.96 (dd, *J* = 2.2, 11.8 Hz, 1H), 6.87 (t, *J* = 8.6 Hz, 1H), 6.66 (s, 1H), 3.93 (s, 3H), 3.87 (s, 3H), 3.82 (s, 3H), 3.80 (d, *J* = 6.0 Hz, 2H), 3.76 (s, 1H). ^13^C NMR (126 MHz, CDCl_3_) δ 152.55, 151.61, 150.58, 148.06, 147.88, 136.00, 132.54, 132.25, 131.15, 130.11, 128.76, 128.68, 128.13, 125.92, 117.53, 114.76, 112.88, 112.39, 56.52, 56.41, 56.34, 47.42; IR (cm^−1^) v = 3373, 3017, 2949, 1132, 821, 712; LCMS *m*/*z*: [M + H]^+^, found 432.39. C_22_H_22_FNO_5_S requires 431.48.

*Synthesis N-benzyl-4,4′,5-trimethoxy-3′-(trifluoromethyl)-[1,1′-biphenyl]-2-sulfonamide* **(8h)**:

**7** (600 g, 1.24 mmol), (4-methoxy-3-(trifluoromethyl) phenyl) boronic acid (540 mg, 2.48 mmol), K_2_CO_3_ (685 mg, 4.96 mmol), and Pd (PPh_3_)_4_ (71 mg, 0.06 mmol) were used according to the general procedure **B** to afford compound **8h** as a white solid (82%). M.P: 124–125 °C, ^1^H NMR (500 MHz, Chloroform-*d*) δ 7.68–7.54 (m, 2H), 7.40 (d, *J* = 2.2 Hz, 1H), 7.21 (dd, *J* = 2.5, 3.9 Hz, 3H), 7.08–6.93 (m, 3H), 6.67 (s, 1H), 3.96 (s, 3H), 3.90 (s, 3H), 3.88 (s, 3H), 3.82 (d, *J* = 6.1 Hz, 2H), 3.71 (t, *J* = 6.2 Hz, 1H).^13^C NMR (126 MHz, CDCl_3_) δ 157.53, 151.74, 148.14, 135.73, 132.54, 130.25, 130.19, 128.82, 128.19, 128.17, 127.83, 127.75, 124.47, 122.30, 118.55, 114.90, 112.59, 111.65, 77.16, 56.55, 56.47, 56.17, 47.48; IR (cm^−1^) v = 3375, 3012, 2907, 1218, 1041, 811; LCMS *m*/*z*: [M + H]^+^, found 482.38. C_23_H_22_F_3_NO_5_S requires 481.49.

*Synthesis N-benzyl-4,5-dimethoxy-4′-(trifluoromethoxy)-[1,1′-biphenyl]-2-sulfonamide* **(8i)**:

**7** (600 g, 1.55 mmol), 4-Trifluoromethoxybenzeneboronic acid (640 mg, 3.1 mmol), K_2_CO_3_ (820 mg, 6.0 mmol), and Pd (PPh_3_)_4_ (80 mg, 0.07 mmol) were used according to the general procedure **B** to afford compound **8i** as a white crystal solid (79%). M.P.: 88–89 °C, ^1^H NMR (500 MHz, Chloroform-*d*) δ 7.64 (s, 1H), 7.38–7.34 (m, 2H), 7.25–7.21 (m, 3H), 7.19–7.15 (m, 2H), 7.00 (dd, *J* = 3.2, 6.4 Hz, 2H), 6.70 (s, 1H), 3.98 (s, 3H), 3.91 (s, 3H), 3.84 (d, *J* = 6.2 Hz, 2H), 3.71 (t, *J* = 6.2 Hz, 1H). ^13^C NMR (126 MHz, CDCl_3_) δ 151.57, 149.16, 148.11, 137.26, 135.83, 132.51, 131.15 (2CH), 130.03, 128.68 (2CH), 128.06, 127.87 (2CH), 120.43, 119.37, 119.26, 114.57, 112.37, 56.44, 56.34, 47.20; IR (cm^−1^) v = 3376, 3017, 2940, 1220, 1165, 843, 664; LCMS *m*/*z*: [M + H]^+^, found 468.39. C_22_H_20_F_3_NO_5_S requires 467.46.

*Synthesis N-benzyl-2-(6-chloro-2-methoxypyridin-3-yl)-4,5-dimethoxybenzenesulfonamide* **(8j)**:

**7** (400 g, 1.03 mmol), (6-chloro-2-methoxypyridin-3-yl) boronic acid (380 mg, 2.06 mmol), K_2_CO_3_ (560 mg, 4.12 mmol), and Pd (PPh_3_)_4_ (58 mg, 0.05 mmol) were used according to the general procedure **B** to afford compound **8j** as a light-yellow viscous liquid (75%). ^1^H NMR (500 MHz, Chloroform-*d*) δ 7.59 (d, *J* = 4.9 Hz, 1H), 7.48 (d, *J* = 7.7 Hz, 1H), 7.32–7.27 (m, 3H), 7.17 (dd, *J* = 1.7, 7.8 Hz, 2H), 6.98 (d, *J* = 7.7 Hz, 1H), 6.70 (s, 1H), 3.98 (s, 3H, CH_3_), 3.95–3.86 (m, 5H, CH_3_, CH_2_), 3.78 (s, 3H, CH_3_). ^13^C NMR (126 MHz, CDCl_3_) δ 159.17, 150.67, 147.36, 141.54, 137.60, 135.13, 129.16, 127.72 (2CH), 127.01, 126.74 (2CH), 123.59, 119.18, 113.68, 111.42, 105.47, 55.35, 55.28, 53.33, 46.30; IR (cm^−1^) v = 3368, 3032, 2972, 1121, 821, 703; LCMS *m*/*z*: [M + H]^+^, found 449.69. C_21_H_21_ClN_2_O_5_S requires 448.92.

General procedure for the deprotection of methoxy groups **(D)**:

To a solution of methoxy-containing compounds (1 eq.) cooled to 0 °C in DCM (5–10 mL), BBr_3_ (1M in DCM, 4 eq. per methoxy group) was added dropwise. The reaction was allowed to warm up to room temperature and stirred overnight. LC/MS analysis revealed complete consumption of the starting material and the formation of the desired product. The reaction is cooled to 0 °C, quenched with water (30 mL), and the mixture is extracted with diethyl ether (3 × 50 mL). Combined org. phases were dried over Na_2_SO_4_, evaporated, and purified via reverse phase flash chromatography (Hexane/EtOAc, gradient, 0–90% EtOAc).

*Synthesis of N-benzyl-3′,4,5-trihydroxy-[1,1′-biphenyl]-2-sulfonamide* **(9a)**:

**8a** (120 mg, 0.29 mmol) and BBr_3_ (0.82 mL, 8.7 mmol) were used according to the general procedure **H** for the deprotection of methoxy groups. Compound **9a** was obtained as a brown viscous liquid (75%). ^1^H NMR (500 MHz, DMSO-*d*_6_) δ 9.95 (s, 1H), 9.60 (s, 1H), 9.39 (s, 1H), 7.95 (s, 1H), 7.38 (s, 1H), 7.27 (dd, *J* = 6.6, 8.1 Hz, 2H), 7.22 (m, 1H), 7.17 (m, 2H), 7.12 (td, *J* = 2.9, 7.4, 8.1 Hz, 2H), 6.73 (ddd, *J* = 1.2, 3.4, 5.8 Hz, 2H), 6.60 (s, 1H), 3.78 (d, *J* = 6.1 Hz, 2H); ^13^C NMR (126 MHz, DMSO-*d*_6_) δ 162.80, 156.84, 148.61, 144.51, 141.56, 138.71, 133.25, 129.64, 128.87, 128.63, 127.91, 127.87, 127.50, 120.76, 119.48, 117.05, 116.31, 114.52, 46.41; IR (cm^−1^) v = 3381, 3660, 3022, 2972, 835; LCMS *m*/*z*: [M + H]^+^, found 372.38. C_19_H_17_NO_5_S requires 371.41.

*Synthesis of N-benzyl-4,4′,5-trihydroxy-[1,1′-biphenyl]-2-sulfonamide* **(9b)**:

**8b** (135 mg, 0.31 mmol) and BBr_3_ (1.34 mL, 14.1 mmol) were used according to the general procedure **H** for the deprotection of methoxy groups. Compound **9b** was obtained as a brown viscous liquid (72%). ^1^H NMR (500 MHz, DMSO-*d*_6_) δ 9.89 (s, 1H), 9.55 (s, 1H), 9.43 (s, 1H), 7.95 (s, 2H), 7.39 (s, 1H), 7.27 (m, 2H), 7.22 (m, 1H), 7.15 (m, 2H), 7.12 (m, 2H), 7.00 (t, *J* = 6.4 Hz, 1H), 6.72 (m, 2H), 6.60 (s, 1H), 3.76 (d, *J* = 6.3 Hz, 2H); ^13^C NMR (126 MHz, DMSO-*d_6_*) δ 162.80, 156.98, 148.67, 144.20, 138.69, 133.39, 131.01, 130.78, 129.73, 128.60, 127.92, 127.49, 119.96, 116.46, 114.75, 46.36; IR (cm^−1^) v = 3389, 3681, 3017, 2992, 839; LCMS *m*/*z*: [M + H]^+^, found 372.32. C_19_H_17_NO_5_S requires 371.41.

*Synthesis of N-benzyl-3′,4,5,5′-tetrahydroxy-[1,1′-biphenyl]-2-sulfonamide* **(9c)**:

**8c** (100 mg, 0.22 mmol) and BBr_3_ (1.28 mL, 13.53 mmol) were used according to the general procedure **H** for the deprotection of methoxy groups. Compound **9c** was obtained as a brown liquid (52%). ^1^H NMR (500 MHz, DMSO-*d*_6_) δ 9.94 (s, 1H), 9.56 (s, 1H), 9.23 (s, 2H), 7.63 (t, *J* = 6.3 Hz, 1H), 7.44 (s, 1H), 7.27 (m, 2H), 7.20 (m, 3H), 6.84 (s, 1H), 6.55 (d, *J* = 2.3 Hz, 2H), 6.49 (t, *J* = 2.3 Hz, 1H), 3.89 (d, *J* = 6.3 Hz, 2H). ^13^C NMR (126 MHz, DMSO-*d*_6_) δ 157.87, 148.60, 144.41, 141.90, 134.88, 133.46, 129.95, 129.89, 129.33, 119.21, 116.23, 115.44, 115.27, 108.41, 101.92, 45.77; IR (cm^−1^) v = 3394, 3342, 3008, 2985, 841; LCMS *m*/*z*: [M + H]^+^, found 388.47. C_19_H_17_NO_6_S requires 387.41.

*Synthesis of N-benzyl-3′,4,4′,5-tetrahydroxy-[1,1′-biphenyl]-2-sulfonamide* **(9d)**:

**8d** (100 mg, 0.225 mmol) and BBr_3_ (0.46 mL, 2.7 mmol) were used according to the general procedure for the deprotection of methoxy groups. Compound **9d** was obtained as a white solid (65%). M.P: 255–256 °C, ^1^H NMR (500 MHz, DMSO-*d*_6_) δ 9.65 (bs, 2H), 8.91 (bs, 2H), 7.38 (d, *J* = 11.8 Hz, 1H), 7.26 (dd, *J* = 1.8, 7.7 Hz, 2H), 7.22 (dd, *J* = 1.5, 7.1 Hz, 1H), 7.17 (m, 2H), 6.80 (m, 1H), 6.74 (m, 1H), 6.68 (m, 1H), 6.60 (s, 1H), 3.75 (d, *J* = 6.3 Hz, 2H); ^13^C NMR (126 MHz, DMSO-*d*_6_) δ 157.87, 148.65, 145.07, 144.63, 144.15, 134.90, 134.88, 133.51, 131.20, 129.96, 129.89, 129.44, 120.96, 119.83, 117.63, 116.36, 115.42, 115.25, 45.68; IR (cm^−1^) v = 3398, 3674, 3020, 2984, 845; LCMS *m*/*z*: [M + H]^+^, found 388.38. C_19_H_17_NO_6_S requires 387.41.

*Synthesis of N-benzyl-4,5-dihydroxy-4′-(trifluoromethyl)-[1,1′-biphenyl]-2-sulfonamide* **(9e)**:

**8e** (70 mg, 0.12 mmol) and BBr_3_ (0.34 mL, 3.6 mmol) were used according to the general procedure **H** for the deprotection of methoxy groups. Compound **9e** was obtained as a white solid (70%). M.P: 161–162 °C, ^1^H NMR (500 MHz, DMSO-*d*_6_) δ 10.06 (s, 1H), 9.76 (s, 1H), 7.67 (m, 3H), 7.50 (d, *J* = 8.0 Hz, 2H), 7.41 (s, 1H), 7.27 (dd, *J* = 6.4, 8.0 Hz, 2H), 7.22 (m, 1H), 7.17 (m, 2H), 6.63 (s, 1H), 3.85 (d, *J* = 6.3 Hz, 2H); ^13^C NMR (126 MHz, DMSO-*d*_6_) δ 148.82, 145.15, 144.69, 138.59, 131.63, 130.71, 129.75, 128.61, 128.06, 127.91, 127.81, 127.51, 126.01, 124.73, 124.70, 123.84, 119.39, 116.51, 46.28; IR (cm^−1^) v = 3381, 3380, 3021, 2992, 1251, 851; LCMS *m*/*z*: [M + H]^+^, found 424.32. C_20_H_16_F_3_NO_4_S requires 423.41.

*Synthesis of N-benzyl-4,5-dihydroxy-3′,5′-bis(trifluoromethyl)-[1,1′-biphenyl]-2-sulfonamide* **(9f)**:

**7** (100 mg, 0.19 mmol) and BBr_3_ (0.72 mL, 7.6 mmol) were used according to the general procedure **H** for the deprotection of methoxy groups. Compound **9f** was obtained as a brown liquid (65%). ^1^H NMR (500 MHz, Methanol-*d*_4_) δ 7.89 (s, 1H), 7.75 (d, *J* = 1.7 Hz, 2H), 7.55 (s, 1H), 7.21 (dd, *J* = 1.7, 5.7 Hz, 3H), 7.08–7.00 (m, 2H), 6.63 (s, 1H), 3.91 (s, 2H). ^13^C NMR (126 MHz, Methanol-*d*_4_) δ 173.02, 164.85, 150.23, 146.48, 143.37, 138.58, 131.78, 131.60, 130.94, 129.38 (2CH), 129.02 (2CH), 128.44, 125.95, 123.79, 122.09, 120.01, 117.90, 61.54, 47.49; IR (cm^−1^) v = 3379, 3640, 3012, 2989, 1275, 849; LCMS *m*/*z*: [M + H]^+^, found 492.38. C_21_H_15_F_6_NO_4_S requires 491.40.

*Synthesis of N-benzyl-3′-fluoro-4,4′,5-trihydroxy-[1,1′-biphenyl]-2-sulfonamide* **(9g)**:

**8g** (50 mg, 0.11 mmol) and BBr_3_ (0.55 mL, 5.8 mmol) were used according to the general procedure **H** for the deprotection of methoxy groups. Compound **9g** was obtained as a light-yellow viscous liquid (65%). ^1^H NMR (500 MHz, *Methanol-d*_4_) δ 7.49 (s, 1H), 7.29–7.16 (m, 3H), 7.09 (dd, *J* = 1.8, 7.7 Hz, 2H), 7.00 (dd, *J* = 2.1, 12.1 Hz, 1H), 6.94–6.80 (m, 2H), 6.65 (s, 1H), 3.84 (s, 2H). ^13^C NMR (126 MHz, Methanol-*d_4_*) δ 152.64, 150.72, 149.80, 145.61, 145.28, 138.46, 133.71, 132.21, 130.16, 129.20, 128.85, 128.28, 126.87, 120.23, 118.59, 117.71, 117.68, 117.38, 47.52; IR (cm^−1^) v = 3398, 3374, 3020, 2984, 845, 687; LCMS *m*/*z*: [M + H]^+^, found 391.38. C_19_H_16_FNO_5_S requires 389.40.

*Synthesis of N-benzyl-4,4′,5-trihydroxy-3′-(trifluoromethyl)-[1,1′-biphenyl]-2-sulfonamide* **(9h)**:

**8h** (100 mg, 0.20 mmol) and BBr_3_ (0.85 mL, 9.0 mmol) were used according to the general procedure **H** for the deprotection of methoxy groups. Compound **9h** was obtained as a light brown viscous liquid (63%). ^1^H NMR (500 MHz, *Methanol-d*_4_) δ 7.51 (s, 1H), 7.48–7.43 (m, 2H), 7.21 (td, *J* = 2.7, 5.8 Hz, 3H), 7.13–7.03 (m, 3H), 6.63 (s, 1H), 3.86 (s, 2H). ^13^C NMR (126 MHz, Methanol-*d*_4_) δ 158.31, 150.07, 145.67, 138.63, 136.21, 133.50, 132.73, 130.65, 129.37 (2CH), 128.99 (2CH), 128.44, 126.18, 120.41, 118.73, 118.49, 117.72, 112.52, 47.61; IR (cm^−1^) v = 3365, 3339, 3017, 2991, 1275, 859; LCMS *m*/*z*: [M + H]^+^, found 340.37. C_20_H_16_F_3_NO_5_S requires 439.41.

*Synthesis of N-benzyl-4,5-dihydroxy-4′-(trifluoromethoxy)-[1,1′-biphenyl]-2-sulfonamide* **(9i)**:

**8i** (100 mg, 0.21 mmol) and BBr_3_ (0.59 mL, 6.3 mmol) were used according to the general procedure **H** for the deprotection of methoxy groups. Compound **9i** was obtained as a brown liquid (72%). ^1^H NMR (500 MHz, Methanol-*d*_4_) δ 7.50 (s, 1H), 7.38–7.32 (m, 2H), 7.27–7.16 (m, 5H), 7.12–7.08 (m, 2H), 6.65 (s, 1H), 3.87 (s, 2H). ^13^C NMR (126 MHz, Methanol-*d*_4_) δ 150.01, 149.85, 145.76, 140.19, 138.80, 133.63, 132.61 (2CH), 130.56, 129.37 (2CH), 128.95 (2CH), 128.44, 122.96, 121.05, 120.22, 117.62, 61.56, 47.55; IR (cm^−1^) v = 3369, 3339, 3007, 2989, 1279, 851; LCMS *m*/*z*: [M + H]^+^, found 440.39. C_20_H_16_F_3_NO_5_S requires 439.41.

*Synthesis of N-benzyl-2-(6-chloro-2-methoxypyridin-3-yl)-4,5-dihydroxybenzenesulfonamide* **(9j)**:

**8j** (25 mg, 0.05 mmol) and BBr_3_ (0.27 mL, 2.8 mmol) were used according to the general procedure **H** for the deprotection of methoxy groups. Compound **9j** was obtained as a brown liquid (65%). ^1^H NMR (500 MHz, Methanol-*d*_4_) δ 7.98 (s, 2H, 2OH), 7.44 (s, 1H), 7.38 (d, *J* = 7.6 Hz, 1H), 7.31–7.20 (m, 3H), 7.20–7.13 (m, 2H), 6.95 (d, *J* = 7.6 Hz, 1H), 6.60 (s, 1H), 3.92 (d, *J* = 9.3 Hz, 2H, CH_2_), 3.79 (s, 3H, OCH_3_). ^13^C NMR (126 MHz, Methanol-*d*_4_) δ 162.18, 150.02, 148.32, 145.98, 142.95, 138.79, 130.66, 129.24 (2CH), 128.72 (2CH), 128.32, 128.25, 122.77, 119.73, 117.39, 116.53, 54.34, 47.38; IR (cm^−1^) v = 3355, 3645, 3034, 2987, 1103, 861, 705; LCMS *m*/*z*: [M + H]^+^, found 421.78. C_19_H_17_ClN_2_O_5_S requires 420.86.

*Synthesis of 2-bromo-N-(4-fluorobenzyl)-4,5-dimethoxybenzenesulfonamide* **(10)**:

**6** (10.0 g, 31.69 mmol) was added to a solution of fluorobenzyl amine (4.15 mL, 38.03 mmol) and DIPEA (16.45 mL, 95.07 mmol) in DCM (30 mL) and cooled to 0 °C. The reaction was allowed to warm to room temperature and stirred for 6 h, until complete conversion of the starting material was confirmed by TLC (3% EtOAc in DCM). The reaction is quenched with water (30 mL). The crude product was extracted with DCM (3 × 30 mL). Combined org. phases were washed with 1M HCl (3 × 50 mL), dried over Na_2_SO_4_, and evaporated to obtain the yellow oil, which was recrystallized from hot MeOH to provide compound **10** as a white crystalline solid (10.41 g, 82%). M.P.: 165–166 °C, ^1^H NMR (400 MHz, DMSO-*d*_6_) δ 8.24 (s, 1H, NH), 7.38 (s, 1H, Ar), 7.26 (s, 1H, Ar), 7.25–7.15 (m, 5H, Ar), 4.08 (s, 2H, CH_2_), 3.84 (s, 3H, CH_3_), 3.76 (s, 3H, CH_3_); ^13^C NMR (101 MHz, DMSO-*d*_6_) δ 151.7, 147.3, 137.6, 131.5, 128.0, 127.6, 127.0, 117.4, 113.3, 110.6, 56.3, 55.8, 46.1; IR (cm^−1^) v = 3363, 3012, 2982, 1129, 833, 706; LCMS *m*/*z*: [M + 2H]^+^, found 406.17. C_15_H_15_BrFNO_4_S requires 404.25.

General procedure for the synthesis of biaryl sulfonamides **(cross-coupling)**:

Biaryl sulfonamides were prepared according to the slightly modified procedure described. Compound **10** (1.0 eq.), appropriate boronic acid (1.5 eq.), potassium carbonate (4.0 eq.) and Pd (PPh_3_)_4_ (0.03 eq.) were suspended in the mixture of toluene, ethanol, and water (5:2:1) (13 mL). A sequential application of N_2_ flow and vacuum was used to degas the mixture before heating in a MW reactor at 120 °C for 1h. The crude product was extracted with ethyl acetate (3 × 30 mL). The organic phase was dried over sodium sulfate. The mixture was then filtered through the bed of celite, and the solvent was removed under reduced pressure. The residue was purified by flash chromatography using Hexane/EtOAc (gradient) to afford the biaryl sulfonamides.

*Synthesis of N-(4-fluorobenzyl)-3′,4,5-trimethoxy-[1,1′-biphenyl]-2-sulfonamide* **(11a)**:

**10** (400 mg, 0.98 mmol), 3-Methoxybenzeneboronic acid (224 mg, 1.48 mmol), K_2_CO_3_ (540 mg, 3.92 mmol), and Pd (PPh_3_)_4_ (23 mg, 0.02 mmol) were used according to general procedure **B** to afford compound **11a** as a white viscous liquid (67%). ^1^H NMR (500 MHz, DMSO-*d*_6_) δ 7.65 (t, *J* = 6.3 Hz, 1H), 7.44 (s, 1H), 7.27 (m, 3H), 7.21 (dd, *J* = 5.7, 8.6 Hz, 2H), 7.09 (t, *J* = 8.9 Hz, 2H), 6.82 (s, 1H), 5.75 (s, 1H), 3.87 (d, *J* = 6.2 Hz, 2H), 3.82 (d, *J* = 7.5 Hz, 6H), 3.75 (s, 3H); ^13^C NMR (126 MHz, DMSO-*d*_6_) δ 158.80, 151.05, 147.79, 141.46, 138.40, 134.50, 131.13, 129.00, 128.64, 128.07, 127.56, 122.37, 115.79, 115.55, 113.27, 111.76, 56.36, 56.33, 55.43, 46.51; IR (cm^−1^) v = 3362, 3011, 2985, 1126, 849, 729; LCMS *m*/*z*: [M + H]^+^, found 432.37. C_22_H_22_FNO_5_S requires 431.48.

*Synthesis of N-(4-fluorobenzyl)-4,4′,5-trimethoxy-[1,1′-biphenyl]-2-sulfonamide* **(11b)**:

**10** (300 mg, 0.58 mmol), 4-Methoxybenzeneboronic acid (170 mg, 1.16 mmol), K_2_CO_3_ (400 mg, 2.9 mmol), and Pd (PPh_3_)_4_ (34 mg, 0.02 mmol) were used according to general procedure **B** to afford compound **11b** as a white solid (83%). M.P: 176–177 °C, ^1^H NMR (500 MHz, DMSO-*d*_6_) δ 7.60 (t, *J* = 6.3 Hz, 1H), 7.41 (s, 1H), 7.30 (m, 2H), 7.21 (dd, *J* = 5.7, 8.6 Hz, 2H), 7.09 (t, *J* = 8.9 Hz, 2H), 6.93 (d, *J* = 8.7 Hz, 2H), 6.78 (s, 1H), 3.84 (d, *J* = 6.4 Hz, 2H), 3.82 (d, *J* = 3.7 Hz, 6H), 3.79 (s, 3H); ^13^C NMR (126 MHz, DMSO-*d*_6_) δ 162.72, 160.79, 159.05, 151.09, 147.54, 134.56, 132.31, 131.12, 130.09, 130.03, 115.87, 115.42, 115.25, 113.41, 111.84, 56.31, 55.56, 45.70; IR (cm^−1^) v = 3377, 3018, 2985, 1105, 851, 721; LCMS *m*/*z*: [M + H]^+^, found 432.29. C_22_H_22_FNO_5_S requires 431.48.

*Synthesis of N-(4-fluorobenzyl)-3′,4,5,5′-tetramethoxy-[1,1′-biphenyl]-2-sulfonamide* **(11c)**:

**10** (500 g, 0.96 mmol), 3,5-Dimethoxybenzeneboronic acid (340 mg, 0.93 mmol), K_2_CO_3_ (660 mg, 4.8 mmol), and Pd (PPh_3_)_4_ (40 mg, 0.03 mmol) were used according to the general procedure **B** to afford compound **11c** as a white solid (67%). M.P: 162–163 °C, ^1^H NMR (500 MHz, DMSO-*d_6_*) δ 7.65 (t, *J* = 6.3 Hz, 1H), 7.42 (s, 1H), 7.22 (dd, *J* = 5.7, 8.6 Hz, 2H), 7.09 (t, *J* = 8.9 Hz, 2H), 6.83 (s, 1H), 6.53 (d, *J* = 2.3 Hz, 2H), 6.48 (t, *J* = 2.3 Hz, 1H), 3.87 (d, *J* = 6.0 Hz, 2H), 3.82 (d, *J* = 6.3 Hz, 6H), 3.74 (s, 6H); ^13^C NMR (126 MHz, DMSO-*d*_6_) δ 161.66, 160.51, 159.73, 158.88, 158.52, 150.00, 146.72, 140.81, 133.55, 133.41, 129.86, 129.02, 128.95, 114.35, 114.19, 110.69, 107.32, 98.59, 93.31, 90.85, 78.57, 55.30, 55.26, 54.51, 54.29, 44.69; IR (cm^−1^) v = 3370, 2982, 1089, 762, 726; LCMS *m*/*z*: [M + H]^+^, found 462.59. C_23_H_24_FNO_6_S requires 461.50.

*Synthesis of N-(4-fluorobenzyl)-3′,4,4′,5-tetramethoxy-[1,1′-biphenyl]-2-sulfonamide* **(11d)**:

**10** (500 mg, 1.23 mmol), 3,4-Dimethoxybenzeneboronic acid (330 mg, 1.84 mmol), K_2_CO_3_ (670 mg, 4.92 mmol), and Pd (PPh_3_)_4_ (30 mg, 0.03 mmol) were used according to the general procedure **B** to afford compound **11d** as a white viscous liquid (82%). ^1^H NMR (500 MHz, DMSO-*d*_6_) δ 7.50 (t, *J* = 6.3 Hz, 1H, NH), 7.46 (s, 1H, Ar), 7.21 (dd, *J* = 5.7, 8.6 Hz, 2H), 7.14 (t, *J* = 8.9 Hz, 2H), 7.01 (d, *J* = 2.0 Hz, 1H, Ar), 6.96 (d, *J* = 8.3 Hz, 1H, Ar), 6.92 (dd, *J* = 8.2, 2.0 Hz, 1H, Ar), 6.84 (s, 1H, Ar), 3.86 (d, *J* = 6.2 Hz, 2H, CH_2_), 3.83 (s, 3H, CH_3_), 3.81 (s, 3H, CH_3_), 3.78 (s, 3H, CH_3_), 3.73 (s, 3H, CH_3_); ^13^C NMR (101 MHz, DMSO-*d*_6_) δ 160.09, 150.6, 148.2, 147.6, 147.1, 137.9, 134.2, 132.0, 130.6, 128.2, 127.6, 127.1, 121.8, 115.4, 113.7, 111.5, 111.0, 55.9, 55.9, 55.5, 55.4, 46.0; IR (cm^−1^) v = 3385, 3002, 2971, 1087, 855, 723; LCMS *m*/*z*: [M + H]^+^, found 462.17. C_23_H_24_FNO_6_S requires 461.13.

*Synthesis of N-(4-fluorobenzyl)-4,5-dimethoxy-4′-(trifluoromethyl)-[1,1′-biphenyl]-2-sulfonamide* **(11e)**:

**10** (500 mg, 0.96 mmol), 4-Trifluoromethylbenzeneboronic acid (270 mg, 1.44 mmol), K_2_CO_3_ (530 mg, 3.84 mmol), and Pd (PPh_3_)_4_ (340 mg, 0.03 mmol) were used according to the general procedure **B** to afford compound **11e** as a white solid (75%). M.P: 177–118 °C, ^1^H NMR (500 MHz, DMSO-*d*_6_) δ 7.93 (t, *J* = 6.3 Hz, 1H), 7.73 (d, *J* = 8.1 Hz, 2H), 7.57 (d, *J* = 8.0 Hz, 2H), 7.45 (s, 1H), 7.21 (dd, *J* = 5.7, 8.6 Hz, 2H), 7.09 (t, *J* = 8.9 Hz, 2H), 6.86 (s, 1H), 3.92 (d, *J* = 6.2 Hz, 2H), 3.82 (d, *J* = 1.9 Hz, 6H); ^13^C NMR (126 MHz, DMSO-*d*_6_) δ 160.09, 153.05, 151.09, 147.54, 134.56, 132.31, 131.12, 130.09, 130.03, 115.87, 115.42, 115.25, 113.41, 111.84, 56.31, 55.56, 46.20; IR (cm^−1^) v = 3377, 3018, 2985, 1105, 851, 721; LCMS *m*/*z*: [M + H]^+^, found 470.47. C_22_H_19_F_4_NO_4_S requires 469.45.

*Synthesis of N-(4-fluorobenzyl)-4,5-dimethoxy-3′,5′-bis(trifluoromethyl)-[1,1′-biphenyl]-2-sulfon-amide* **(11f)**:

**10** (700 g, 1.72 mmol), 3,5-Ditrifluoromethylbenzeneboronic acid (880 mg, 3.44 mmol), K_2_CO_3_ (950 mg, 6.8 mmol), and Pd (PPh_3_)_4_ (92 mg, 0.08 mmol) were used according to the general procedure **B** to afford compound **11f** as a white solid (88%). M.P: 143–144 °C, ^1^H NMR (500 MHz, Chloroform-*d*) δ 7.87 (s, 1H), 7.75 (d, *J* = 1.6 Hz, 2H), 7.63 (s, 1H), 6.99 (dd, *J* = 5.4, 8.5 Hz, 2H), 6.92 (t, *J* = 8.6 Hz, 2H), 6.68 (s, 1H), 3.99 (s, 3H), 3.93 (s, 3H), 3.89 (d, *J* = 3.5 Hz, 2H); ^13^C NMR (126 MHz, CDCl_3_) δ 163.86, 161.90, 152.26, 149.01, 141.24, 131.93, 131.68, 131.61, 131.41, 130.69, 130.41, 130.16, 130.09, 124.55, 122.43, 116.19, 116.02, 114.80, 113.03, 77.16, 56.91, 56.88, 46.83; IR (cm^−1^) v = 3374, 2908, 1232, 1097, 889, 706; LCMS *m*/*z*: [M + H]^+^, found 538.39. C_23_H_18_F_7_NO_4_S requires 537.45.

General procedure for the deprotection of methoxy groups **(D)**:

To a solution of methoxy-containing compounds (1 eq.) cooled to 0 °C in DCM (5–10 mL), BBr_3_ (1M in DCM, 4 eq. per methoxy group) was added dropwise. The reaction was allowed to warm up to room temperature and stirred overnight. LC/MS analysis revealed complete consumption of the starting material and the formation of the desired product. The reaction is cooled to 0 °C, quenched with water (30 mL), and the mixture is extracted with diethyl ether (3 × 50 mL). Combined org. phases were dried over Na_2_SO_4_, evaporated, and purified via reverse phase flash chromatography (Hexane/EtOAc, gradient, 0–>90% EtOAc).

*Synthesis of N-(4-fluorobenzyl)-3′,4,5-trihydroxy-[1,1′-biphenyl]-2-sulfonamide* **(12a)**:

**11a** (100 mg, 0.23 mmol) and BBr_3_ (0.66 mL, 6.95 mmol) were used according to the general procedure **H** for the deprotection of methoxy groups. Compound **12a** was obtained as a brown viscous liquid (52%). ^1^H NMR (500 MHz, DMSO-*d*_6_) δ 9.96 (s, 1H), 9.60 (s, 1H), 9.39 (s, 1H), 7.95 (s, 1H), 7.37 (s, 1H), 7.23–7.16 (m, 3H), 7.15–7.07 (m, 3H), 6.75–6.71 (m, 2H), 6.61 (s, 1H), 3.78 (d, *J* = 6.1 Hz, 2H); ^13^C NMR (126 MHz, DMSO-*d*_6_) δ 162.80, 162.70, 160.77, 156.83, 148.64, 144.50, 141.54, 134.96, 134.93, 133.27, 129.92, 129.86, 129.56, 128.87, 120.75, 119.49, 117.05, 116.34, 115.42, 115.25, 114.53, 45.65; IR (cm^−1^) v = 3367, 3333, 3012, 2991, 1320, 846, 711; LCMS *m*/*z*: [M + H]^+^, found 390.51. C_19_H_16_FNO_5_S requires 389.40.

*Synthesis of N-(4-fluorobenzyl)-4,4′,5-trihydroxy-[1,1′-biphenyl]-2-sulfonamide* **(12b)**:

**11b** (60 mg, 0.14 mmol) and BBr_3_ (0.36 mL, 2.08 mmol) were used according to the general procedure **H** for the deprotection of methoxy groups. Compound **12b** was obtained as a brown viscous liquid (65%). ^1^H NMR (500 MHz, DMSO-*d*_6_) 9.88 (s, 1H), 9.53 (s, 1H), 9.41 (s, 1H), 7.95 (s, 1H), 7.36 (s, 1H), 7.18 (m, 2H), 7.10 (m, 4H), 6.71 (d, *J* = 8.5 Hz, 2H), 6.59 (s, 1H), 3.75 (d, *J* = 6.3 Hz, 2H); ^13^C NMR (126 MHz, DMSO-*d*_6_) δ 156.97, 148.68, 144.19, 134.96, 134.94, 133.40, 130.99, 130.76, 129.93, 129.87, 129.65, 119.97, 116.48, 115.39, 115.22, 114.75, 45.58; IR (cm^−1^) v = 3359, 3347, 3017, 2988, 1281, 855; LCMS *m*/*z*: [M + H]^+^, found 390.48. C_19_H_16_FNO_5_S requires 389.40.

*Synthesis of N-(4-fluorobenzyl)-3′,4,5,5′-tetrahydroxy-[1,1′-biphenyl]-2-sulfonamide* **(12c)**:

**11c** (100 mg, 0.21 mmol) and BBr_3_ (1.23 mL, 13.0 mmol) were used according to the general procedure **H** for the deprotection of methoxy groups. Compound **12c** was obtained as a brown liquid (61%). ^1^H NMR (500 MHz, DMSO-*d*_6_) δ 9.94 (s, 1H), 9.56 (s, 1H), 9.23 (s, 2H), 7.34 (s, 1H), 7.22 (dd, *J* = 5.7, 8.6 Hz, 2H), 7.10 (m, 2H), 6.97 (t, *J* = 6.4 Hz, 1H), 6.60 (s, 1H), 6.20 (d, *J* = 2.2 Hz, 2H), 6.16 (t, *J* = 2.2 Hz, 1H), 3.78 (d, *J* = 6.4 Hz, 2H); ^13^C NMR (126 MHz, DMSO-*d*_6_) δ 160.78, 157.87, 148.60, 144.41, 141.90, 134.88, 133.46, 129.95, 129.89, 129.33, 119.21, 116.23, 115.44, 115.27, 108.41, 101.92, 45.77; IR (cm^−1^) v = 3323, 3371, 3011, 2900, 1234, 886, 719; LCMS *m*/*z*: [M + H]^+^, found 406.41. C_19_H_16_FNO_6_S requires 405.40.

*Synthesis of N-(4-fluorobenzyl)-3′,4,4′,5-tetrahydroxy-[1,1′-biphenyl]-2-sulfonamide* **(12d)**:

**11d** (215 mg, 0.45 mmol) and BBr_3_ (0.86 mL, 9.0 mmol) were used according to the general procedure for the deprotection of methoxy groups. Compound **12d** was obtained as a brown liquid (68%). ^1^H NMR (500 MHz, DMSO-*d*_6_) δ 9.65 (s, 2H), 8.91 (s, 2H), 7.35 (s, 1H), 7.19 (dd, *J* = 5.7, 8.6 Hz, 2H), 7.09 (t, *J* = 8.9 Hz, 2H), 6.73 (d, *J* = 2.1 Hz, 1H), 6.68 (d, *J* = 8.1 Hz, 1H), 6.59 (m, 2H), 3.74 (s, 2H); ^13^C NMR (126 MHz, DMSO-*d*_6_) δ 162.69, 160.76, 148.65, 145.07, 144.63, 144.15, 134.90, 134.88, 133.51, 131.20, 129.96, 129.89, 129.44, 120.96, 119.83, 117.63, 116.36, 115.42, 115.25, 115.21, 45.68; IR (cm^−1^) v = 3349, 3333, 3009, 2987, 1233, 841, 714; LCMS *m*/*z*: [M + H]^+^, found 406.37. C_19_H_16_FNO_6_S requires 405.40.

*Synthesis of N-(4-fluorobenzyl)-4,5-dihydroxy-4′-(trifluoromethyl)-[1,1′-biphenyl]-2-sulfonamide* **(12e)**:

**11e** (190 mg, 0.40 mmol) and BBr_3_ (0.76 mL, 8.1 mmol) were used according to the general procedure **H** for the deprotection of methoxy groups. Compound **12e** was obtained as a white solid (72%). ^1^H NMR (500 MHz, DMSO-*d*_6_) δ 10.07 (s, 1H), 9.76 (s, 1H), 7.68 (d, *J* = 8.2 Hz, 2H), 7.50 (d, *J* = 8.0 Hz, 2H), 7.39 (s, 1H), 7.20 (dd, *J* = 5.7, 8.6 Hz, 2H), 7.09 (m, 2H), 6.63 (s, 1H), 3.84 (d, *J* = 6.1 Hz, 2H); ^13^C NMR (126 MHz, DMSO-*d*_6_) δ 160.78, 148.86, 145.15, 144.65, 134.82, 131.64, 130.69, 129.93, 129.86, 129.65, 128.08, 127.83, 126.00, 124.77, 124.74, 124.71, 124.68, 123.84, 119.39, 116.52, 115.40, 115.23, 45.52; IR (cm^−1^) v = 3391, 3369, 3006, 2900, 1232, 889, 721; LCMS *m*/*z*: [M + H]^+^, found 600.59. C_20_H_15_F_4_NO_4_S requires 599.67.

*Synthesis of N-(4-fluorobenzyl)-4,5-dihydroxy-3′,5′-bis(trifluoromethyl)-[1,1′-biphenyl]-2-sulfon-amide* **(12f)**:

**11f** (45 mg, 0.08 mmol) and BBr_3_ (0.30 mL, 3.2 mmol) were used according to the general procedure **H** for the deprotection of methoxy groups. Compound **12f** was obtained as a brown liquid (70%). ^1^H NMR (500 MHz, Methanol-*d*_4_) δ 7.98 (s, 1H), 7.90 (d, *J* = 1.7 Hz, 1H), 7.78 (d, *J* = 1.6 Hz, 1H), 7.52 (s, 1H), 7.07 (dd, *J* = 5.4, 8.5 Hz, 2H), 6.95 (t, *J* = 8.8 Hz, 2H), 6.65 (s, 1H), 3.89 (s, 2H);IR (cm^−1^) v = 3393, 3318, 2988, 1232, 891, 711; LCMS *m*/*z*: [M + H]^+^, found 510.37. C_21_H_14_F_7_NO_4_S requires 509.39.

General procedure for the synthesis of biaryl sulfonamides **(cross-coupling)**:

Biaryl sulfonamides were prepared according to the slightly modified procedure described. Compound **7** (1.0 eq.), appropriate boronic acid (1.5 eq.), potassium carbonate (4.0 eq.) and Pd (PPh_3_)_4_ (0.03 eq.) were suspended in the mixture of toluene, ethanol, and water(5:2:1) (13 mL). A sequential application of N_2_ flow and vacuum was used to degas the mixture before heating in a MW reactor at 120 °C for 1h. The crude product was extracted with ethyl acetate (3 × 30 mL). The organic phase was dried over sodium sulfate. The mixture was then filtered through the bed of celite, and the solvent was removed under reduced pressure. The residue was purified by flash chromatography using Hexane/EtOAc (gradient) to afford the biaryl sulfonamides.

General procedure for the synthesis of sultams via cyclization of secondary sulfonamides **(C)**:

Cyclization was achieved according to the procedure described in the literature: appropriate sulfonamide (1 eq.), PIDA (1.10 eq.), I_2_ (1.10 eq.), and K_2_CO_3_ (1.50 eq.) were suspended in DCM (20 mL) [52]. The dark red solution was stirred at 35 °C for 1–3 h, until almost complete conversion was determined by TLC (3% EtOAc in DCM). The reaction is quenched with sat. aq. Na_2_S_2_O_5_, and the mixture is stirred at the room temperature until the discoloration of the solution occurs. The crude product was extracted with DCM and purified via flash chromatography (DCM/EtOAc, gradient, 0->1% EtOAc), and triturated with MeOH to afford sultams.

*Synthesis of 6-benzyl-2,3,9-trimethoxy-6H-dibenzo[c,e][1,2]thiazine 5,5-dioxide* **(14a)**:

**13a** (180 mg, 0.43 mmol), PIDA (150 mg, 0.47 mmol), I_2_ (110 mg, 0.47 mmol), and K_2_CO_3_ (89 mg, 0.65 mmol) were used according to the general procedure **C** to afford compound **14a** as a white solid, purified by flash chromatography using (DCM/EtOAc, gradient, 0->1% EtOAc) to afford (45% **14a**). M.P: 191–192 °C, ^1^H NMR (500 MHz, DMSO-*d*_6_) δ 7.62 (d, *J* = 2.9 Hz, 1H), 7.37 (m, 2H), 7.29 (d, *J* = 8.9 Hz, 1H), 7.26 (s, 1H), 7.20 (s, 1H), 7.06 (m, 1H), 6.99 (td, *J* = 2.1, 8.7 Hz, 2H), 6.69 (dt, *J* = 1.3, 7.0 Hz, 1H), 4.96 (s, 2H), 3.95 (d, *J* = 4.3 Hz, 6H), 3.89 (s, 3H); ^13^C NMR (126 MHz, DMSO-*d*_6_) δ 156.14, 153.29, 148.57, 148.46, 135.21, 130.06, 127.85, 127.29, 126.86, 126.68, 125.32, 124.64, 116.50, 111.30, 109.85, 108.26, 108.18, 104.23, 55.67, 55.53, 55.02, 52.70; IR (cm^−1^) v = 3373, 3010, 2974, 1114, 879; LCMS *m*/*z*: [M + H]^+^, found 412.36. C_22_H_21_NO_5_S requires 411.47.

*Synthesis of 6-benzyl-2,3,8-trimethoxy-6H-dibenzo[c,e][1,2]thiazine 5,5-dioxide* **(14b)**:

**13b** (180 mg, 0.43 mmol), PIDA (150 mg, 0.47 mmol), I_2_ (110 mg, 0.47 mmol), and K_2_CO_3_ (89 mg, 0.65 mmol) were used according to the general procedure **C** to afford compound **14b** as a white viscous liquid (75%). ^1^H NMR (500 MHz, DMSO-*d*_6_) δ 8.11 (d, *J* = 9.6 Hz, 1H), 7.49 (s, 1H), 7.37 (s, 1H), 7.18 (m, 5H), 6.90 (m, 2H), 5.16 (s, 2H), 3.95 (s, 6H), 3.75 (s, 3H); ^13^C NMR (126 MHz, DMSO-*d*_6_) δ 159.30, 151.66, 147.73, 137.78, 135.56, 127.83, 126.86, 126.67, 126.39, 124.99, 124.92, 116.62, 110.43, 107.22, 105.30, 103.23, 55.55, 55.42, 54.88, 48.77; IR (cm^−1^) v = 3377, 3016, 2974, 1124, 877; LCMS *m*/*z*: [M + H]^+^, found 412.46. C_15_H_16_BrNO_4_S requires 411.47.

*Synthesis of 6-benzyl-2,3,7,9-tetramethoxy-6H-dibenzo[c,e][1,2]thiazine 5,5-dioxide* **(14c)**:

**13c** (150 mg, 0.33 mmol), PIDA (115 mg, 0.36 mmol), I_2_ (91 mg, 0.36 mmol), and K_2_CO_3_ (67 mg, 0.49 mmol) were used according to the general procedure **C** to afford compound **14c** as a white viscous liquid (69%). ^1^H NMR (500 MHz, DMSO-*d*_6_) δ 7.21 (s, 1H), 7.18 (s, 1H), 7.03–6.92 (m, 5H, Ar), 6.75 (s, 1H), 6.52 (s, 1H), 4.64 (s, 2H), 3.94 (s, 3H), 3.89 (s, 3H), 3.88 (s, 3H), 3.86 (s, 3H); ^13^C NMR (126 MHz, DMSO-*d*_6_) δ 152.20, 149.81, 148.72, 147.37, 135.52, 132.03, 131.95, 128.44, 128.32, 127.73, 126.56, 126.32, 118.73, 107.40, 107.19, 106.99, 105.32, 77.16, 56.37, 56.34, 56.25, 55.74, 54.43; IR (cm^−1^) v = 3347, 3016, 2985, 1108, 864; LCMS *m*/*z*: [M + H]^+^, found 442.37. C_23_H_23_NO_6_S requires 441.50.

*Synthesis of 6-benzyl-2,3,8,9-tetramethoxy-6H-dibenzo[c,e][1,2]thiazine 5,5-dioxide* **(14d)**:

**13d** (213 mg, 0.48 mmol), PIDA (170.16 mg, 0.52 mmol), I_2_ (134 mg, 0.53 mmol), and K_2_CO_3_ (99.5 mg, 0.72 mmol) were used according to the general procedure **C** to afford compound **14d** as a white viscous liquid (55%). ^1^H NMR (500 MHz, Chloroform-*d*) δ 7.40 (s, 1H, Ar), 7.22–7.16 (m, 6H, Ar), 7.08 (s, 1H, Ar), 6.54 (s, 1H, Ar), 4.79 (s, 2H, CH_2_), 4.01 (d, *J* = 8.3 Hz, 6H, CH_3_), 3.95 (s, 3H, CH_3_), 3.67 (s, 3H, CH_3_); ^13^C NMR (126 MHz, CDCl_3_) δ 152.20, 149.81, 148.72, 147.37, 135.52, 132.03, 131.95, 128.44, 128.32, 127.73, 126.56, 126.32, 118.73, 107.40, 107.19, 106.99, 105.32, 77.16, 56.37(OCH_3_), 56.34(OCH_3_), 56.25(OCH_3_), 55.74(OCH_3_), 54.43(CH_2_); IR (cm^−1^) v = 3377, 3012, 2975, 1118, 868; LCMS *m*/*z*: [M + H]^+^, found 442.57. C_23_H_23_NO_6_S requires 441.50.

*Synthesis of 6-benzyl-2,3-dimethoxy-8-(trifluoromethyl)-6H-dibenzo[c,e][1,2]thiazine 5,5-dioxide* **(14e)**:

**13e** (100 mg, 0.22 mmol), PIDA (77 mg, 0.24 mmol), I_2_ (60 mg, 0.24 mmol), and K_2_CO_3_ (46 mg, 0.33 mmol) were used according to the general procedure **C** to afford compound **14e** as a white solid (72%). M.P.: 198–199 °C, ^1^H NMR (500 MHz, DMSO-*d*_6_) δ 8.44 (d, *J* = 8.3 Hz, 1H), 7.77 (d, *J* = 1.7 Hz, 1H), 7.65 (s, 1H), 7.47 (s, 1H), 7.19 (m, 4H), 7.10 (m, 2H), 5.26 (s, 2H), 3.97 (d, *J* = 7.0 Hz, 6H); ^13^C NMR (126 MHz, DMSO-*d*_6_) δ 152.76, 150.49, 137.82, 137.59, 135.96, 131.15, 130.79, 128.97, 128.63, 128.53, 128.18, 128.16, 128.12, 128.09, 127.80, 127.40, 124.31, 121.62, 121.58, 118.73, 109.59, 104.48, 56.81, 56.71, 50.36, 19.03; IR (cm^−1^) v = 3337, 3011, 2995, 1232, 1128, 874; LCMS *m*/*z*: [M + H]^+^, found 450.39. C_22_H_18_F_3_NO_4_S requires 449.44.

*Synthesis of 6-benzyl-9-fluoro-2,3,8-trimethoxy-6H-dibenzo[c,e][1,2]thiazine 5,5-dioxide* **(14f)**:

**13f** (150 mg, 0.31 mmol), PIDA (109 mg, 0.34 mmol), I_2_ (86 mg, 0.34 mmol), and K_2_CO_3_ (63 mg, 0.46 mmol) were used according to the general procedure **C** to afford compound **14f** as a white solid (52%). M.P: 238–239 °C, ^1^H NMR (500 MHz, Chloroform-*d*) δ 7.47 (d, *J* = 12.0 Hz, 1H, Ar), 7.37 (s, 1H, Ar), 7.20 (p, *J* = 2.3, 2.8 Hz, 3H, Ar), 7.16 (dd, *J* = 2.6, 7.2 Hz, 2H, Ar), 7.02 (s, 1H, Ar), 6.60 (d, *J* = 7.8 Hz, 1H, Ar), 4.83 (s, 2H, CH_2_), 3.98 (d, *J* = 2.1 Hz, 6H, 2OCH_3_), 3.66 (s, 3H, OCH_3_). ^13^C NMR (126 MHz, CDCl_3_) δ 152.85, 151.66, 149.54, 148.50, 135.91, 132.53, 128.98, 128.57, 128.35, 126.97, 125.88, 119.28, 119.23, 112.49, 108.95, 108.93, 107.41, 105.57, 56.88, 56.74, 56.53, 54.48; IR (cm^−1^) v = 3376, 3022, 2957, 1116, 1141, 811, 702; LCMS *m*/*z*: [M + H]^+^, found 430.39. C_22_H_20_FNO_5_S requires 429.46.

*Synthesis of 6-benzyl-2,3,8-trimethoxy-9-(trifluoromethyl)-6H-dibenzo[c,e][1,2]thiazine 5,5-dioxide* **(14g)**:

**13g** (150 mg, 0.31 mmol), PIDA (100 mg, 0.34 mmol), I_2_ (86 mg, 0.34 mmol), and K_2_CO_3_ (63 mg, 0.46 mmol) were used according to the general procedure **C** to afford compound **14g** as a white solid (62%). M.P.: 231–232 °C, ^1^H NMR (500 MHz, Chloroform-*d*) δ 7.96 (s, 1H, Ar), 7.41 (s, 1H, Ar), 7.29–7.22 (m, 5H, Ar), 7.15 (s, 1H, Ar), 6.66 (s, 1H, Ar), 5.07 (s, 2H, CH_2_), 4.01 (d, *J* = 13.9 Hz, 6H, 2OCH_3_), 3.66 (s, 3H, OCH_3_). ^13^C NMR (126 MHz, CDCl_3_) δ 158.09, 153.03, 149.57, 136.02, 129.30, 128.41, 127.77 (2CH), 126.67, 125.68, 124.37, 124.33, 122.51, 117.05, 116.10, 115.85, 107.30, 104.97, 60.79, 56.88, 56.84, 56.33, 51.82; IR (cm^−1^) v = 3366, 3029, 2972, 1243, 1121, 821; LCMS *m*/*z*: [M + H]^+^, found 480.51. C_23_H_20_F_3_NO_5_S requires 479.47.

*Synthesis of 6-benzyl-2,3-dimethoxy-8-(trifluoromethoxy)-6H-dibenzo[c,e][1,2]thiazine 5,5-dioxide* **(14h)**:

**13h** (210 mg, 0.44 mmol), PIDA (150 mg, 0.48 mmol), I_2_ (121 mg, 0.48 mmol), and K_2_CO_3_ (91 mg, 0.66 mmol) were used according to the general procedure **C** to afford compound **14h** as a white viscous liquid (70%). ^1^H NMR (500 MHz, Chloroform-*d*) δ 7.85 (d, *J* = 8.8 Hz, 1H, Ar), 7.45 (s, 1H, Ar), 7.24–7.17 (m, 6H, Ar), 7.10 (ddd, *J* = 1.1, 2.4, 8.7 Hz, 1H, Ar), 7.03 (dd, *J* = 1.1, 2.3 Hz, 1H, Ar), 5.05 (s, 2H, CH_2_), 4.01 (d, *J* = 4.1 Hz, 6H, 2OCH_3_). ^13^C NMR (126 MHz, CDCl_3_) δ 152.61, 149.79, 149.33, 139.25, 135.25, 128.85, 128.06, 127.64, 127.59, 126.43, 125.19, 123.69, 121.43, 119.37, 117.40, 114.10, 107.60, 104.79, 56.66, 56.49, 52.00; IR (cm^−1^) v = 3376, 3037, 2943, 1225, 1135, 823, 661; LCMS *m*/*z*: [M + H]^+^, found 466.41. C_22_H_18_F_3_NO_5_S requires 465.44.

General procedure for the deprotection of methoxy groups **(D)**:

To a solution of appropriately protected compound (1 eq.) cooled to 0 °C in DCM (5–10 mL) was added BBr_3_ (1M in DCM, 5 eq. per methoxy group). The reaction was allowed to warm up to room temperature and stirred for 3–12 h. LC/MS analysis revealed complete consumption of the starting material and the formation of Demethylated N-benzyl sultam as a major product alongside a small amount of corresponding debenzylated sultam. The reaction is cooled to 0 °C and quenched with water and a mixture extracted with diethyl ether (3 × 50 mL). The combined organic phases were dried over Na_2_SO_4_, evaporated, and the residue was purified via a reverse phase column to afford a compound.

*Synthesis of 6-benzyl-2,3,9-trihydroxy-6H-dibenzo[c,e][1,2]thiazine 5,5-dioxide* **(15a)**:

**14a** (50 mg, 0.12 mmol) and BBr_3_ (0.51 mL, 5.4 mmol) were used according to the general procedure **I** for the deprotection of methoxy groups. Compound **15a** was obtained as a brown viscous liquid (52%). ^1^H NMR (500 MHz, DMSO-*d*_6_) δ 10.06 (bs, 2H), 9.68 (s, 1H), 7.62 (d, *J* = 2.9 Hz, 1H), 7.37 (m, 2H), 7.29 (d, *J* = 8.9 Hz, 1H), 7.26 (s, 1H), 7.20 (s, 1H), 7.06 (m, 1H), 6.99 (td, *J* = 2.1, 8.7 Hz, 2H), 6.69 (dt, *J* = 1.3, 7.0 Hz, 1H), 4.96 (s, 2H); ^13^C NMR (126 MHz, DMSO-*d_6_*) δ 156.14, 153.29, 148.57, 148.46, 135.21, 130.06, 127.85, 127.29, 126.86, 126.68, 125.32, 124.64, 116.50, 111.30, 109.85, 108.26, 108.18, 104.23, 52.70; IR (cm^−1^) v = 3393, 3371, 3025, 2981, 849; LCMS *m*/*z*: [M + H]^+^, found 370.37. C_19_H_15_NO_5_S requires 369.39.

*Synthesis of 6-benzyl-2,3,8-trihydroxy-6H-dibenzo[c,e][1,2]thiazine 5,5-dioxide* **(15b)**:

**14b** (50 mg, 0.12 mmol) and BBr_3_ (0.51 mL, 5.4 mmol) were used according to the general procedure **I** for the deprotection of methoxy groups. Compound **15b** was obtained as a brown viscous liquid (61%). ^1^H NMR (500 MHz, DMSO-d_6_) δ 10.03 (s, 1H), 9.98 (s, 1H), 9.93 (s, 1H), 7.69 (d, *J* = 8.8 Hz, 1H), 7.24 (m, 8H), 6.70 (dd, *J* = 2.4, 8.6 Hz, 1H), 6.65 (d, *J* = 2.4 Hz, 1H), 5.03 (s, 2H); ^13^C NMR (126 MHz, DMSO-d_6_) δ 158.54, 150.39, 145.57, 139.03, 137.11, 128.95, 128.92, 127.80, 127.38, 126.50, 125.20, 124.76, 116.24, 113.03, 111.62, 108.16, 107.24, 50.12; IR (cm^−1^) v = 3394, 3379, 3021, 2980, 829; LCMS *m*/*z*: [M + H]^+^, found 370.51. C_19_H_15_NO_5_S requires 369.39.

*Synthesis of 6-benzyl-2,3,7,9-tetrahydroxy-6H-dibenzo[c,e][1,2]thiazine 5,5-dioxide* **(15c)**:

**14c** (50 mg, 0.11 mmol) and BBr_3_ (0.6 mL, 4.2 mmol) were used according to the general procedure **D** for the deprotection of methoxy groups. Compound **15c** was obtained as a viscous liquid (50%). ^1^H NMR (500 MHz, DMSO-*d*_6_) 9.98 (s, 1H, OH), 9.90 (s, 1H, OH), 9.46 (s, 1H, OH), 9.26 (s, 1H, OH), δ 7.21 (s, 1H), 7.18 (s, 1H), 7.03–6.92 (m, 5H, Ar), 6.75 (s, 1H), 6.52 (s, 1H), 4.64 (s, 2H); ^13^ C NMR (101 MHz, DMSO-*d*_6_) δ 149.2, 145.9, 144.8, 140.1, 134.5, 128.5, 127.3, 127.3, 125.7, 125.6, 125.6, 120.4, 114.7, 114.0, 111.4, 108.5, 52.5; IR (cm^−1^) v = 3385, 3360, 3012, 2992, 848; LCMS *m*/*z*: [M + H]^+^, found 386.43, C_19_H_15_NO_6_S requires 385.39.

*Synthesis of 6-benzyl-2,3,8,9-tetrahydroxy-6H-dibenzo[c,e][1,2]thiazine 5,5-dioxide* **(15d)**:

**14d** (50 mg, 0.11 mmol) and BBr_3_ (0.6 mL, 4.2 mmol) were used according to the general procedure **D** for the deprotection of methoxy groups. Compound **15d** was obtained as a gray viscous liquid (66%). ^1^H NMR (500 MHz, DMSO-*d*_6_) δ 9.98 (s, 1H, OH), 9.90 (s, 1H, OH), 9.46 (s, 1H, OH), 9.26 (s, 1H, OH), 7.21 (m, 3H, Ar), 7.16 (s, 1H, Ar), 7.12 (m, 3H, Ar), 7.06 (s, 1H, Ar), 6.63 (s, 1H, Ar), 4.82 (s, 2H, CH_2_); ^13^ C NMR (101 MHz, DMSO-*d_6_*) δ 149.2, 145.9, 144.8, 140.1, 134.5, 128.5, 127.3, 127.3, 125.7, 125.6, 125.6, 120.4, 114.7, 114.0, 111.4, 108.5, 52.5(CH_2_); IR (cm^−1^) v = 3385, 3360, 3012, 2992, 848; LCMS *m*/*z*: [M + H]^+^, found 386.43, C_19_H_15_NO_6_S requires 385.39.

*Synthesis of 6-benzyl-2,3-dihydroxy-8-(trifluoromethyl)-6H-dibenzo[c,e][1,2]thiazine 5,5-dioxide* **(15e)**:

**14e** (200 mg, 0.44 mmol) and BBr_3_ (1.26 mL, 13.3 mmol) were used according to the general procedure **I** for the deprotection of methoxy groups. Compound **14e** was obtained as a white solid (68%). M.P: 183–184 °C, ^1^H NMR (500 MHz, DMSO-*d*_6_) δ 10.43 (bs, 2H, OH), 7.71 (d, *J* = 1.8 Hz, 1H, Ar), 7.60 (dd, *J* = 1.7, 8.5 Hz, 1H, Ar), 7.43 (s, 1H, Ar), 7.32 (s, 1H, Ar), 7.18 (m, 4H, Ar), 7.08 (dd, *J* = 2.0, 6.4 Hz, 2H, Ar), 5.20 (s, 2H, CH_2_). ^13^C NMR (126 MHz, DMSO-*d*_6_) δ 148.82, 145.15, 144.69, 138.59, 131.63, 130.71, 129.75, 128.61, 128.06, 127.91, 127.81, 127.51, 126.01, 124.73, 124.70, 123.84, 119.39, 116.51, 46.91; IR (cm^−1^) v = 3379, 3340, 3012, 2989, 1275, 849; LCMS *m*/*z*: [M + H]^+^, found 422.41, C_20_H_14_F_3_NO_4_S requires 421.39.

*Synthesis of 6-benzyl-9-fluoro-2,3,8-trihydroxy-6H-dibenzo[c,e][1,2]thiazine 5,5-dioxide* **(15f)**:

**14f** (70 mg, 0.16 mmol) and BBr_3_ (0.76 mL, 8.1 mmol) were used according to the general procedure **D** for the deprotection of methoxy groups. Compound **15f** was obtained as a light-yellow viscous liquid (71%). ^1^H NMR (500 MHz, *Methanol-d*_4_) δ 7.97 (brs, 3H, OH), 7.51 (d, *J* = 12.2 Hz, 1H, Ar), 7.24 (s, 1H, Ar), 7.19–7.14 (m, 3H, Ar), 7.13–7.07 (m, 3H, Ar), 6.79 (d, *J* = 8.0 Hz, 1H, Ar), 4.89 (s, 2H, CH_2_). ^13^ C NMR (126 MHz, *Methanol-d_4_*) δ 151.90, 151.16, 149.99, 147.00, 146.88, 137.07, 135.98, 129.34, 128.91, 128.69, 127.05, 126.25, 119.73, 119.68, 112.92, 112.90, 112.69, 109.55, 54.20; IR (cm^−1^) v = 3359, 3347, 3016, 2972, 1270, 861, 706; LCMS *m*/*z*: [M + H]^+^, found 488.43, C_19_H_14_FNO_5_S requires 387.38.

*Synthesis of 6-benzyl-2,3-dihydroxy-8-methoxy-9-(trifluoromethyl)-6H-dibenzo[c,e][1,2]thiazine 5,5-dioxide* **(15g)**:

**14g** (82 mg, 0.17 mmol) and BBr_3_ (0.73 mL, 7.69 mmol) were used according to the general procedure **D** for the deprotection of methoxy groups. Compound **15g** was obtained as a yellow viscous liquid (68%). ^1^H NMR (500 MHz, *Methanol-d*_4_) δ 7.99 (br, 2H, OH), 7.98 (s, 1H, Ar), 7.32 (s, 1H, Ar), 7.29–7.13 (m, 6H, Ar), 6.95 (s, 1H, Ar), 5.17 (s, 2H, CH_2_), 3.77 (s, 3H, OCH_3_). ^13^ C NMR (126 MHz, *Methanol-d*_4_) δ 173.01, 164.87, 158.76, 151.49, 147.38, 143.31, 137.15, 129.66, 128.89, 128.77, 127.00, 125.54, 124.53, 124.48, 118.78, 112.15, 109.27, 106.47, 61.54, 56.72, 51.77; IR (cm^−1^) v = 3355, 3645, 3034, 2987, 1275, 861; LCMS *m*/*z*: [M + H]^+^, found 452.47, C_21_H_16_F_3_NO_5_S requires 451.42.

*Synthesis of 6-benzyl-2,3-dihydroxy-8-(trifluoromethoxy)-6H-dibenzo[c,e][1,2]thiazine 5,5-dioxide* **(15h)**:

**14h** (100 mg, 0.21 mmol) and BBr_3_ (0.6 mL, 6.45 mmol) were used according to the general procedure **D** for the deprotection of methoxy groups. Compound **15h** was obtained as a light-yellow viscous liquid (69%). ^1^H NMR (500 MHz, *Methanol-d*_4_) δ 7.97 (br, 2H, OH), 7.92 (d, *J* = 9.5 Hz, 1H, Ar), 7.33 (s, 1H, Ar), 7.28 (s, 1H, Ar), 7.20–7.14 (m, 5H, Ar), 7.13–7.08 (m, 2H, Ar), 5.05 (s, 2H, CH_2_). ^13^C NMR (126 MHz, *Methanol-d*_4_) δ 164.68, 151.17, 149.74, 147.73, 140.01, 136.54, 129.31, 128.66, 127.77, 127.34, 126.01, 125.17, 122.62, 120.58, 118.49, 115.68, 112.88, 109.20, 52.55, 36.78; IR (cm^−1^) v = 3369, 3339, 3007, 2989, 1251, 846; LCMS *m*/*z*: [M + H]^+^, found 438.56, C_20_H_14_F_3_NO_5_S requires 437.39.

General procedure for the synthesis of sultams via cyclization of secondary sulfonamides (**C**):

Cyclization was achieved according to the procedure described in the literature: appropriate sulfonamide (1 eq.), PIDA (1.10 eq.), I_2_ (1.10 eq.), and K_2_CO_3_ (1.50 eq.) were suspended in DCM (20 mL) [52]. The dark red solution was stirred at 35 °C for 1–3 h, until almost complete conversion was determined by TLC (3% EtOAc in DCM). The reaction is quenched with sat. aq. Na_2_S_2_O_5_, and the mixture is stirred at the room temperature until the discoloration of the solution occurs. The crude product was extracted with DCM and purified via flash chromatography (DCM/EtOAc, gradient, 0->1% EtOAc), and triturated with MeOH to afford sultams.

*Synthesis of 6-(4-fluorobenzyl)-2,3,9-trimethoxy-6H-dibenzo[c,e][1,2]thiazine 5,5-dioxide* **(17a)**:

**16a** (200 mg, 0.46 mmol), PIDA (160 mg, 0.50 mmol), I_2_ (126 mg, 0.50 mmol), and K_2_CO_3_ (95.9 mg, 0.69 mmol) were used according to the general procedure **C** to afford compound **17a** as a white solid, purified by flash chromatography using (DCM/EtOAc, gradient, 0->1% EtOAc) to afford 55%. ^1^H NMR (500 MHz, DMSO-*d*_6_) δ 7.61 (d, J = 2.8 Hz, 1H, Ar), 7.58 (dd, *J* = 1.2, 8.2 Hz, 1H, Ar), 7.48 (s, 1H, Ar), 7.39 (t, *J* = 8.1 Hz, 1H, Ar), 7.34 (s, 1H, Ar), 7.33 (d, *J* = 9.0 Hz, 1H, Ar), 7.23 (m, 1H, Ar), 7.19 (dd, *J* = 1.1, 8.4 Hz, 1H, Ar), 6.98 (m, 1H, Ar), 4.93 (s, 2H), 3.95 (d, *J* = 2.3 Hz, 6H), 3.92 (s, 3H); ^13^C NMR (126 MHz, DMSO-*d*_6_) δ 161.81, 156.33, 153.30, 148.60, 131.09, 129.93, 127.21, 126.68, 125.28, 124.67, 116.51, 114.62, 113.55, 111.37, 109.85, 108.31, 104.35, 55.68, 55.57, 55.03, 50.83; IR (cm^−1^) v = 3371, 3014, 2995, 1115, 856, 731; LCMS *m*/*z*: [M + H]^+^, found 430.47, C_22_H_20_FNO_5_S requires 429.46.

*Synthesis of 6-(4-fluorobenzyl)-2,3,8-trimethoxy-6H-dibenzo[c,e][1,2]thiazine 5,5-dioxide* **(17b)**:

**16b** (100 mg, 0.23 mmol), PIDA (80 mg, 0.25 mmol), I_2_ (63 mg, 0.25 mmol), and K_2_CO_3_ (98 mg, 0.34 mmol) were used according to the general procedure **C** to afford compound **17b** as a white solid (70%). M.P.: 198–199 °C. ^1^H NMR (500 MHz, DMSO-*d*_6_) δ 8.10 (d, *J* = 8.8 Hz, 1H), 7.47 (s, 1H), 7.36 (s, 1H), 7.17 (m, 2H), 7.04 (m, 2H), 6.94 (d, *J* = 2.5 Hz, 1H), 6.91 (dd, *J* = 2.6, 8.7 Hz, 1H), 5.14 (s, 2H), 3.95 (s, 3H), 3.91 (s, 3H), 3.77 (s, 3H); ^13^C NMR (126 MHz, DMSO-*d*_6_) δ 162.86, 160.92, 160.44, 152.77, 148.83, 138.73, 132.61, 129.97, 129.91, 127.49, 126.08, 126.02, 117.94, 115.76, 115.59, 111.80, 108.33, 106.64, 104.38, 56.64, 56.51, 56.01, 49.43; IR (cm^−1^) v = 3379, 3011, 2981, 1110, 857, 728; LCMS *m*/*z*: [M + H]^+^, found 430.17, C_22_H_20_FNO_5_S requires 429.10.

*Synthesis of 6-(4-fluorobenzyl)-2,3,7,9-tetramethoxy-6H-dibenzo[c,e][1,2]thiazine 5,5-dioxide* **(17c)**:

**16c** (170 mg, 0.36 mmol), PIDA (128 mg, 0.40 mmol), I_2_ (101 mg, 0.40 mmol), and K_2_CO_3_ (74 mg, 0.54 mmol) were used according to the general procedure **C** to afford compound **17c** as a white solid (68%). M.P: 196–197 °C, ^1^H NMR (500 MHz, DMSO-*d*_6_) δ 7.21 (s, 1H), 7.18 (s, 1H), 7.03 (d, *J* = 2.5 Hz, 1H), 6.75 (d, *J* = 2.5 Hz, 1H), 6.72 (dd, *J* = 2.9, 7.4 Hz, 4H), 4.64 (s, 2H), 3.94 (s, 3H), 3.89 (s, 3H), 3.88 (s, 3H), 3.86 (s, 3H); ^13^C NMR (126 MHz, DMSO-*d*_6_) δ 161.77, 159.83, 158.22, 154.52, 151.21, 148.60, 130.14, 130.07, 129.29, 129.05, 127.44, 125.38, 118.37, 113.49, 113.32, 108.56, 104.49, 100.69, 98.98, 55.66, 55.48, 55.10, 52.27; IR (cm^−1^) v = 3369, 3014, 2911, 1230, 1103, 891, 720; LCMS *m*/*z*: [M + H]^+^, found 460.53, C_19_H_16_FNO_6_S requires 459.49.

*Synthesis of 6-(4-fluorobenzyl)-2,3,8,9-tetramethoxy-6H-dibenzo[c,e][1,2]thiazine 5,5-dioxide* **(17d)**:

**16d** (200 mg, 0.43 mmol), PIDA (150 mg, 0.47 mmol), I_2_ (110 mg, 0.47 mmol), and K_2_CO_3_ (80 mg, 0.64 mmol) were used according to the general procedure **C** to afford compound **17d** as a white solid (55%). M.P: 196–197 °C, ^1^H NMR (500 MHz, Chloroform-*d*) δ 7.37 (s, 1H), 7.16 (s, 1H), 7.08 (dd, *J* = 5.4, 8.6 Hz, 2H), 7.03 (s, 1H), 6.85 (t, *J* = 8.6 Hz, 2H), 6.58 (s, 1H), 4.76 (s, 2H), 4.00 (d, *J* = 5.8 Hz, 6H), 3.95 (s, 3H), 3.74 (s, 3H); ^13^C NMR (126 MHz, CDCl_3_) δ 163.34, 161.38, 152.38, 150.03, 148.91, 147.71, 131.91, 131.16, 131.14, 130.21, 130.14, 126.75, 126.39, 119.23, 115.30, 115.13, 107.53, 107.37, 107.12, 105.41, 56.48, 56.40, 55.97, 54.14; IR (cm^−1^) v = 3395, 3012, 2978, 1089, 858, 750; LCMS *m*/*z*: [M + H]^+^, found 460.57, C_23_H_22_FNO_6_S requires 459.49.

*Synthesis of 6-(4-fluorobenzyl)-2,3-dimethoxy-8-(trifluoromethyl)-6H-dibenzo[c,e][1,2]thiazine 5,5-dioxide* **(17e)**:

**16e** (200 mg, 0.46 mmol), PIDA (160 mg, 0.50 mmol), I_2_ (126 mg, 0.50 mmol), and K_2_CO_3_ (95.9 mg, 0.69 mmol) were used according to the general procedure **C** to afford compound **17e** as a white solid (66%). M.P: 227–228 °C, ^1^H NMR (500 MHz, DMSO-*d*_6_) δ 8.43 (d, *J* = 8.3 Hz, 1H), 7.81 (d, *J* = 1.7 Hz, 1H), 7.67 (dd, *J* = 1.7, 8.4 Hz, 1H), 7.63 (s, 1H), 7.45 (s, 1H), 7.11 (m, 2H), 7.03 (m, 2H), 5.24 (s, 2H), 3.97 (d, *J* = 4.4 Hz, 6H); ^13^C NMR (126 MHz, DMSO-*d*_6_) δ 162.94, 161.00, 152.78, 150.50, 137.69, 131.99, 130.06, 130.00, 128.79, 128.12, 127.42, 124.30, 121.83, 119.12, 115.84, 115.67, 109.60, 104.56, 56.82, 56.71, 49.91; IR (cm^−1^) v = 3376, 3012, 2907, 1220, 1103, 891, 719; LCMS *m*/*z*: [M + H]^+^, found 468.53, C_22_H_17_F_4_NO_4_S requires 467.43.

General procedure for the deprotection of methoxy groups **(D)**:

To a solution of appropriately protected compound (1 eq.) cooled to 0 °C in DCM (5–10 mL) was added BBr_3_ (1M in DCM, 5 eq. per methoxy group). The reaction was allowed to warm up to room temperature and stirred for 3–12 h. LC/MS analysis revealed complete consumption of the starting material and the formation of Demethylated N-benzyl sultam as a major product alongside a small amount of corresponding debenzylated sultam. The reaction is cooled to 0 °C and quenched with water and a mixture extracted with diethyl ether (3 × 50 mL). The combined org. phases were dried over Na_2_SO_4_, evaporated, and the residue was purified via a reverse phase column to afford a compound.

*Synthesis of 6-(4-fluorobenzyl)-2,3,9-trihydroxy-6H-dibenzo[c,e][1,2]thiazine 5,5-dioxide* **(18a)**:

**17a** (40 mg, 0.09 mmol) and BBr_3_ (0.38 mL, 4.1 mmol) were used according to the general procedure **D** for the deprotection of methoxy groups. Compound **18a** was obtained as a light brown viscous liquid (55%). ^1^H NMR (500 MHz, DMSO-*d*_6_) δ 10.05 (s, 2H, OH), 9.69 (s, 1H, OH), 7.19 (s, 1H, Ar), 7.14 (d, J = 8.8 Hz, 1H, Ar), 7.12 (s, 1H, Ar), 7.09 (d, *J* = 2.7 Hz, 1H, Ar), 7.03 (dd, *J* = 2.7, 5.8 Hz, 2H, Ar), 6.98 (m, 2H, Ar), 6.76 (dd, *J* = 2.7, 8.7 Hz, 1H, Ar), 4.80 (s, 2H, CH_2_); IR (cm^−1^) v = 3361, 3335, 3011, 2991, 1249, 851, 716; LCMS *m*/*z*: [M + H]^+^, found 388.32, C_19_H_14_FNO_5_S requires 387.38.

*Synthesis of 6-(4-fluorobenzyl)-2,3,8-trihydroxy-6H-dibenzo[c,e][1,2]thiazine 5,5-dioxidedioxide* **(18b)**:

**17b** (55 mg, 0.13 mmol) and BBr_3_ (0.45 mL, 5.4 mmol) were used according to the general procedure **I** for the deprotection of methoxy groups. Compound **18b** was obtained as a gray solid (61%). M.P: 193–194 °C, ^1^H NMR (500 MHz, DMSO-*d*_6_) δ 9.89 (s, 2H, OH), 9.69 (s, 1H, OH), 7.68 (d, *J* = 8.7 Hz, 1H, Ar), 7.24 (s, 1H, Ar), 7.19 (m, 3H, Ar), 7.09 (m, 2H, Ar), 6.71 (dd, *J* = 2.4, 8.6 Hz, 1H, Ar), 6.65 (d, *J* = 2.4 Hz, 1H, Ar), 4.99 (s, 2H, CH_2_); ^13^C NMR (126 MHz, DMSO-*d*_6_) δ 160.78, 148.86, 145.15, 144.65, 134.82, 131.64, 130.69, 129.93, 129.86, 129.65, 128.08, 127.83, 126.00, 124.77, 124.74, 124.71, 124.68, 123.84, 119.39, 116.52, 115.40, 115.23, 45.52; IR (cm^−1^) v = 3362, 3333, 3013, 2981, 1240, 849; LCMS *m*/*z*: [M + H]^+^, found 388.43, C_19_H_14_FNO_5_S requires 387.38.

*Synthesis of 6-(4-fluorobenzyl)-2,3,7,9-tetrahydroxy-6H-dibenzo[c,e][1,2]thiazine 5,5-dioxide* **(18c)**:

**17c** (260 mg, 0.56 mmol) and BBr_3_ (2.13 mL, 22.1 mmol) were used according to the general procedure **D** for the deprotection of methoxy groups. Compound **18c** was obtained as a light-yellow viscous liquid (65%). ^1^H NMR (500 MHz, DMSO-*d_6_*) δ 9.99 (s, 1H, OH), 9.91 (s, 1H, OH), 9.49 (s, 1H, OH), 9.30 (s, 1H, OH), 7.21 (s, 1H), 7.18 (s, 1H), 7.03 (d, *J* = 2.5 Hz, 1H), 6.75 (d, *J* = 2.5 Hz, 1H), 6.72 (dd, *J* = 2.9, 7.4 Hz, 4H), 4.64 (s, 2H); ^13^C NMR (126 MHz, DMSO-*d_6_*) δ 160.76, 148.65, 145.07, 144.63, 144.15, 134.90, 134.88, 133.51, 131.20, 129.96, 129.89, 129.44, 120.96, 119.83, 117.63, 116.36, 115.42, 115.25, 115.21, 45.68. IR (cm^−1^) v = 3321, 3361, 3013, 2911, 1231, 879, 711; LCMS *m*/*z*: [M + H]^+^, found 404.49. C_19_H_14_FNO_6_S requires 403.38.

*Synthesis of 6-(4-fluorobenzyl)-2,3,8,9-tetrahydroxy-6H-dibenzo[c,e][1,2]thiazine 5,5-dioxide* **(18d)**:

**17d** (260 mg, 0.56 mmol) and BBr_3_ (2.13 mL, 22.1 mmol) were used according to the general procedure **D** for the deprotection of methoxy groups. Compound **18d** was obtained as a light-yellow viscous liquid (62%). ^1^H NMR (400 MHz, DMSO-*d*_6_) δ 9.99 (s, 1H, OH), 9.91 (s, 1H, OH), 9.49 (s, 1H, OH), 9.30 (s, 1H, OH), 7.14 (s, 1H, Ar), 7.09 (d, *J* = 2.0 Hz, 2H, Ar), 7.03 (m, 4H, Ar), 6.63 (s, 1H, Ar), 4.77 (s, 2H, CH_2_); ^13^C NMR (126 MHz, DMSO-*d*_6_) δ 160.76, 148.65, 145.07, 144.63, 144.15, 134.90, 134.88, 133.51, 131.20, 129.96, 129.89, 129.44, 120.96, 119.83, 117.63, 116.36, 115.42, 115.25, 115.21, 45.68. IR (cm^−1^) v = 3321, 3361, 3013, 2911, 1231, 879, 711; LCMS *m*/*z*: [M + H]^+^, found 404.49. C_19_H_14_FNO_6_S requires 403.38.

*Synthesis of 6-(4-fluorobenzyl)-2,3-dihydroxy-8-(trifluoromethyl)-6H-dibenzo[c,e][1,2]thiazine 5,5-dioxide* **(18e)**:

**17e** (30 mg, 0.06 mmol) and BBr_3_ (0.12 mL, 0.64 mmol) were used according to the general procedure **D** for the deprotection of methoxy groups. Compound **18e** was obtained as a light brown solid (68%). M.P: 189–190 °C, ^1^H NMR (500 MHz, DMSO-*d*_6_) δ 10.38 (s, 2H, OH), 8.10 (d, *J* = 8.4 Hz, 1H, Ar), 7.74 (d, *J* = 1.7 Hz, 1H, Ar), 7.61 (dd, *J* = 1.8, 8.5 Hz, 1H, Ar), 7.41 (s, 1H, Ar), 7.30 (s, 1H, Ar), 7.10 (dd, J = 5.6, 8.7 Hz, 2H, Ar), 7.02 (m, 2H, Ar), 5.18 (s, 2H, CH_2_); ^13^C NMR (126 MHz, DMSO-*d*_6_) δ 160.78, 148.86, 145.15, 144.65, 134.82, 131.64, 130.69, 129.93, 129.86, 129.65, 128.08, 127.83, 126.00, 124.77, 124.74, 124.71, 124.68, 123.84, 119.39, 116.52, 115.40, 115.23, 45.52; IR (cm^−1^) v = 3390, 3674, 2906, 1232, 889, 711; LCMS *m*/*z*: [M + H]^+^, found 440.41, C_20_H_13_F_4_NO_4_S requires 439.38.

*Synthesis of 2,3,8,9-tetrahydroxy-6H-dibenzo[c,e][1,2]thiazine 5,5-dioxide* **(21a)**:

According to the general procedure **E** for the deprotection of benzyl groups. Compound **21a** was obtained as a beige viscous liquid (50%). ^1^H NMR (500 MHz, DMSO-*d*_6_) δ ^1^H NMR (500 MHz, DMSO-d_6_) δ 10.34 (s, 1H, NH), 9.93 (s, 1H, OH), 9.85 (s, 1H, OH), 9.53 (s, 1H, OH), 9.00 (s, 1H, OH), 7.13 (m, 3H, Ar), 6.54 (s, 1H, Ar). ^13^C NMR (126 MHz, DMSO-*d*_6_) δ 149.87, 147.06, 145.26, 142.93, 128.97, 125.37, 114.09, 110.79, 110.73, 108.02, 107.63; IR (cm^−1^) v = 3381, 3326, 3026, 2970, 840; LCMS *m*/*z*: [M + H]^+^, found 396.33, C_12_H_9_NO_6_S requires 295.27.

*Synthesis of 2,3,7,9-tetrahydroxy-6H-dibenzo[c,e][1,2]thiazine 5,5-dioxide* **(21b)**:

According to the general procedure **E** for the deprotection of methoxy and benzyl groups. Compound **21b** was obtained as a beige viscous liquid (95%). ^1^H NMR (500 MHz, *Methanol-d_4_*) δ 7.97 (s, NH), 7.30 (s, 1H, Ar), 7.26 (s, 1H, Ar), 6.78 (d, *J* = 2.5 Hz, 1H, Ar), 6.41 (d, *J* = 2.5 Hz, 1H, Ar); ^13^C NMR (126 MHz, *Methanol-d*_4_) δ 155.62, 151.06, 150.36, 146.80, 128.19, 126.82, 126.32, 117.54, 112.23, 108.68, 103.48, 101.89; IR (cm^−1^) v = 3377, 3319, 3011, 2978, 849; LCMS *m*/*z*: [M + H]^+^, found 296.37, C_12_H_9_NO_6_S requires 295.27.

### 4.2. Molecular Docking

The MOE v. 2019.01 was utilized to conduct computational docking studies [53]. The protein structure with the PDB ID code 7FS5 was prepared using the protein preparation module in MOE [20]. This involved adding missing atoms and residues, correcting bond order and formal charge, and adjusting the tautomer. The system underwent protonation using the protonate 3D technique, and the electrostatics functioned using the generalized Born volume integral (GB/VI) approach, with a dielectric constant value of 80. The values chosen for the electrostatics and van der Waals cut-offs were 10 and 15 Å, respectively. The protein was electrically charged and reduced in size using the AMBER10:EHT force field applied in the MOE program.

The compounds were constructed using ChemBioDraw Ultra 14.0 and then subjected to charge and minimization using the MMFF94x force field [54]. Additionally, the hydrogens and lone pairs were adjusted. The dataset underwent energy minimization with a preset RMS gradient of 0.1 kcal/mol/Å^2^. After undergoing protonation and minimization, the compounds were stored in the MOE database format. 

The allosteric site, located between chains B and D, was selected for docking to encompass all atoms within a 5 Å radius of the cognate ligand. Before conducting the docking process, a thorough evaluation of various combinations of scoring and placement procedures in MOE Dock was carried out to determine the most appropriate combination of algorithm and scoring functions for the target protein. The induced fit docking process, using the Triangle Matcher algorithm for placement and LondonDG as the initial scoring method, together with GBVI/WSA dG as the re-scoring method, yielded the most reliable results. For every compound, a total of 10 separate docking runs were performed, resulting in the generation of 100 conformations. Subsequently, the highest-ranked solution for each drug was evaluated for binding mode analysis.

The QikProp module implemented in Schrödinger was used to calculate the ADME properties of the selected active compounds. The compounds were loaded into the Maestro workspace in mol2 format, and quick calculations using QikProp were performed on them. For additional processing, the findings were exported in CSV format.

### 4.3. Biological Experiment Method

#### 4.3.1. Pyruvate Kinase Activity

HepG2 KO cells were lysed and protein lysated with CelLytic M (C2978, Sigma-Aldrich, St. Louis, MO, USA) lysis buffer. The effect of 10 µM compounds on pyruvate kinase activity on protein lysate was measured with the Pyruvate Kinase Assay Kit (ab83432, Abcam, Cambridge, UK), followed by the manufacturer’s instructions. 2 × 10^4^ HepG2 WT cells and 1 × 10^5^ HepG2 KO cells were seeded into 96-well plates for a pyruvate kinase activity assay on cells. Drugs were treated at 20 µM concentration on the cell for 4 h and washed with 250 µL of PBS. Cells were lysed with 50 µL PK assay buffer (ab83432, Abcam) and started reaction and kinetic O.D measurement with 50 µL PK assay buffer that contained the assay component (ab83432, Abcam). O.D. values were measured with a microplate reader (Hidex Sense Beta Plus, Turku, Finland) at 570 nm wavelength.

#### 4.3.2. Cell Culture Experiments and In Vitro Steatosis Induction

HepG2 wild-type cells and HepG2 PKM CRISPR KO cells were purchased from the genome engineering company Synthego (Menlo Park, CA, USA). Cells were maintained with growth media RPMI 1640 (R2405, Sigma-Aldrich) and supplemented with 10% fetal bovine serum (FBS, F7524, Sigma-Aldrich) and 1% P/S (P4333, Sigma-Aldrich). Steatosis induction media was prepared as DMEM high glucose (D0819, Sigma-Aldrich) with 10% FBS, 1% P/S media supplemented with 10 µg/mL insulin (I9278, Sigma-Aldrich), and 10 µM T0901317 (T2320, Sigma-Aldrich) for one week. HepG2 cells were seeded 6 × 10^4^ cells per well into a 96-well plate for TAG and MTT assays after steatosis and 1 × 10^6^ cells per well into a 6-well plate for western blot analysis after steatosis. HepG2 cells were incubated with steatosis-inducing media for one week by 3 days + 2 days + 2 days with a total of three media exchanges. 

#### 4.3.3. TAG Measurement and MTT Assay

HepG2 cells were seeded at 6 × 10^4^ cells per well into 96-well plates and induced steatosis for one week with a designated concentration of small molecules. After steatosis induction, cells were washed with 200 µL of PBS, and triglyceride contents were measured by the Triglyceride Assay Kit-Quantification (ab65336, Abcam) following the manufacturer’s instructions. O.D. values were detected with a microplate reader at 570 nm (Hidex Sense Beta Plus). A 5 mg/mL MTT (M2128, Sigma-Aldrich) solution in PBS (10 µL) was added to each well for the MTT assay. After 1 h, the MTT solution and all media were removed from the wells. 100 μL of DMSO was added and mixed to dissolve the formazan crystals. Cell viability was analyzed by measuring the absorbance of the dissolved formazan in a microplate reader at a wavelength of 570 nm with a microplate reader (Hidex Sense Beta Plus). 

#### 4.3.4. Western Blot

After steatosis induction with a designated concentration of compounds, whole cell lysate was prepared with CelLytic M (C2978, Sigma-Aldrich) buffer. SDS PAGE was performed using Mini-PROTEAN^®^ TGX™ Precast Gels (Bio-Rad, Hercules, CA, USA) and transferred using the Trans-Blot^®^ Turbo™ Transfer System (Bio-Rad). FASN (ab22759, Abcam), ChREBP (92809, Abcam), PKL (06653, Sigma), PKM (4053S, Cell Signaling Technology, Danvers, MA, USA), β-actin (ab8227, Abcam), and GAPDH (sc-47724, Santa Cruz Biotechnology, Inc., Dallas, TX, USA) were blotted as primary antibodies overnight. Secondary antibodies, Goat Anti-Rabbit HRP (ab205718, Abcam) and goat anti-mouse IgG-HRP (sc2005, Santa Cruz Biotechnology, Inc.), were blotted for one hour. The protein band was detected with ImageQuantTMLAS 500 (29-0050-63, GE Healthcare Bio-Sciences AB, Uppsala, Sweden).

#### 4.3.5. Cellular Thermal Shift Assay (Cetsa)

HepG2 WT cells were trypsinized and transferred into a 1.5 mL tube at 500,000 cells per mL concentration. Compound **15e** was treated with 10 µM and 20 µM in the 1.5 mL tube and incubated for 2 h in a 37 °C CO_2_ incubator. Tubes sustained heat shock at 60 °C for 5 min. with a pre-heated heat block. After centrifugation for heated cells at 13,000 rpm for 1 min, cell pellets were lysed with 50 µL of Native lysis Buffer (ab156035, Abcam). Centrifugation was again used to purify soluble proteins in a lysed solution, and the same volume (9 µL) of each group was analyzed using Western blot.

## 5. Conclusions

This study investigated PKL inhibitors, focusing on sulfone derivatives of the urolithin C structure, known to be an allosteric inhibitor of PKL. Building upon previous research, we designed and synthesized two new series of compounds by modifying the C and B rings with linear and cyclic sulfonamide groups, integrating electron-donating and -withdrawing groups to assess their impact on cell permeability. The synthesized compounds were evaluated for their efficacy in inhibiting PKL activity, reducing triacylglycerol (TAG) levels, and their effects on cell viability, with urolithin C serving as the benchmark compound. While modest improvements were observed in liver cell lines, a significant increase in inhibition was observed in HepG2 cell lysates. Specifically, compounds **15d**, **9d**, **15e**, **18a**, **12d**, and **15a** displayed promising IC_50_ values ranging from 4.3 µM to 18.7 µM. Our findings emphasize the importance of polarity and hydrophilicity in achieving effective PKL inhibition. Extensive studies on modification sites and PKL inhibition data revealed that functionalizing -OH groups on ring C with -CF_3_ enhanced inhibitory activity, cell permeability, and drug-like properties. 

Among the compounds, **15e** stood out, demonstrating encouraging results by significantly decreasing PKL expression levels, reducing TAG content, and showing effective cellular uptake. These outcomes highlight **15e** as a promising candidate for the pharmacological management of MAFLD. The promising results warrant further research and development of this compound to explore its potential as a new therapeutic option for MAFLD.

## Figures and Tables

**Table 1:** In-silico ADME predictions of the active compound’s series.

Comp	Mol_MW	^a^ Don-HB	^b^ Acp-HB	^c^ N-rot	^d^ QPlogPo/w	^e^ QPlogS	^f^ PSA	^g^ QPPCaco	^h^ QPlogBB	^i^ QPlogKhsa	^j^ OA%
		(<5)	(<10)	(0-15)	(<5)	(<0.5)	(<140)	(<25 poor; >500 high)	(−3.0–1.2)	(−1.5–1.5)	(<25% is poor)
15d	385.391	4	7.5	2	0.951	−3.412	129.392	25.664	−2.283	−0.198	57.735
9d	387.406	5	7.5	5	0.992	−2.676	132.578	41.289	−2.172	−0.341	61.673
15e	421.39	2	6	3	3.132	−4.998	85.144	204.507	−1.061	0.291	86.644
12d	405.397	5	7.5	5	1.205	−2.908	132.35	43.149	−2.042	−0.308	63.266
15a	369.391	3	6.75	2	1.571	−3.726	107.747	62.105	−1.867	−0.062	68.24

^a^ Number of hydrogen bond donors (Don-HB). ^b^ Number of hydrogen bond acceptors (Acp-HB). ^c^ Number of rotatable bonds (N-rot). ^d^ Logarithm of partition coefficient between n-octanol and water (QPlog P). ^e^ Aqua solubility parameter (QPlog S). ^f^ Polar Surface Area (PSA). ^g^ Caco-2 Cell permeability in nm/s. ^h^ Blood/Brain Partition Coefficient (QPlogBB). ^i^ Human serum albumin binding coefficient (QPlogKhsa). ^j^ Percentage of human oral absorption (%OA).

## Data Availability

The original contributions presented in the study are included in the article/Supplementary Material, further inquiries can be directed to the corresponding author.

## Supplementary Materials

The following supporting information can be downloaded at: https://www.mdpi.com/article/10.3390/ijms25147986/s1.

## References

[1] Moreira-Pais A., Ferreira R., Oliveira P.A., Neuparth M.J., Appell H.J., Duarte J.A., Ferreira R., Oliveira P.F., Nogueira-Ferreira R. (2023). Glycolysis and skeletal muscle plasticity: Lactate as a key signaling molecule. Glycolysis Tissue-Specific Metabolic Regulation in Physio-Pathological, Conditions.

[2] Fothergill-Gilmore L.A., Michels P.A.M. (1993). Evolution of glycolysis. Prog. Biophys. Mol. Biol..

[3] Liu H., Takagaki Y., Kumagai A., Kanasaki K., Koya D. (2021). The PKM2 activator TEPP-46 suppresses kidney fibrosis via inhibition of the EMT program and aberrant glycolysis associated with suppression of HIF-1α accumulation. J. Diabetes Investig..

[4] Muirhead H. (1990). Isoenzymes of pyruvate kinase. Biochem. Soc. Trans..

[5] Christofk H.R., Heiden M.G.V., Harris M.H., Ramanathan A., Gerszten R.E., Wei R., Fleming M.D., Schreiber S.L., Cantley L.C. (2008). The M2 splice isoform of pyruvate kinase is important for cancer metabolism and tumour growth. Nature.

[6] Goldberg M.S., Sharp P.A. (2012). Pyruvate kinase M2-specific siRNA induces apoptosis and tumor regression. J. Exp. Med..

[7] Yamada K., Noguchi T. (1999). Nutrient and hormonal regulation of pyruvate kinase gene expression. Biochem. J..

[8] Marjot T., Moolla A., Cobbold J.F., Hodson L., Tomlinson J.W., Tomlinson J. (2019). Nonalcoholic fatty liver disease in adults: Current concepts in etiology, outcomes, and management. Endocr. Rev..

[9] Younossi Z.M., Koenig A.B., Abdelatif D., Fazel Y., Henry L., Wymer M. (2016). Global epidemiology of nonalcoholic fatty liver disease—Meta-analytic assessment of prevalence, incidence, and outcomes. Hepatology.

[10] Huang D.Q., El-Serag H.B., Loomba R. (2021). Global epidemiology of NAFLD-related HCC: Trends, predictions, risk factors and prevention. Nat. Rev. Gastroenterol. Hepatol..

[11] Battisti U.M., Monjas L., Akladios F., Matic J., Andresen E., Nagel C.H., Hagkvist M., Håversen L., Kim W., Uhlen M. (2023). Exploration of Novel Urolithin C Derivatives as Non-Competitive Inhibitors of Liver Pyruvate Kinase. Pharmaceuticals.

[12] Flint A., Andersen G., Hockings P., Johansson L., Morsing A., Palle M.S., Vogl T., Loomba R., Plum-Mörschel L. (2021). Randomised clinical trial: Semaglutide versus placebo reduced liver steatosis but not liver stiffness in subjects with non-alcoholic fatty liver disease assessed by magnetic resonance imaging. Aliment. Pharmacol. Ther..

[13] Aslan E., Guler C., Adem S. (2016). In vitro effects of some flavonoids and phenolic acids on human pyruvate kinase isoenzyme M2. J. Enzyme Inhib. Med. Chem..

[14] Baer-Dubowska W., Szaefer H., Majchrzak-Celińska A., Krajka-Kuźniak V. (2020). Tannic Acid: Specific Form of Tannins in Cancer Chemoprevention and Therapy-Old and New Applications. Curr. Pharmacol. Rep..

[15] Adem S., Aslan A., Ahmed I., Krohn K., Guler C., Comakli V., Demirdag R., Kuzu M. (2016). Inhibitory and Activating Effects of Some Flavonoid Derivatives on Human Pyruvate Kinase Isoenzyme M2. Arch. Pharm..

[16] Rodriguez-Ramiro I., Vauzour D., Minihane A.M. (2016). Polyphenols and non-alcoholic fatty liver disease: Impact and mechanisms. Proc. Nutr. Soc..

[17] Abenavoli L., Milic N., Luzza F., Boccuto L., De Lorenzo A. (2017). Polyphenols Treatment in Patients with Nonalcoholic Fatty Liver Disease. J. Transl. Intern. Med..

[18] Li S., Tan H.Y., Wang N., Cheung F., Hong M., Feng Y. (2018). The Potential and Action Mechanism of Polyphenols in the Treatment of Liver Diseases. Oxid. Med. Cell. Longev..

[19] Sarno S., Salvi M., Battistutta R., Zanotti G., Pinna L.A. (2005). Features and potentials of ATP-site directed CK2 inhibitors. Biochim. Biophys. Acta—Proteins Proteom..

[20] Nain-Perez A., Nilsson O., Lulla A., Håversen L., Brear P., Liljenberg S., Hyvönen M., Borén J., Grøtli M. (2023). Tuning liver pyruvate kinase activity up or down with a new class of allosteric modulators. Eur. J. Med. Chem..

[21] Battisti U.M., Gao C., Akladios F., Kim W., Yang H., Bayram C., Bolat I., Kiliclioglu M., Yuksel N., Tozlu O.O. (2023). Ellagic Acid and Its Metabolites as Potent and Selective Allosteric Inhibitors of Liver Pyruvate Kinase. Nutrients.

[22] Van De Wier B., Koek G.H., Bast A., Haenen G.R.M.M. (2017). The potential of flavonoids in the treatment of non-alcoholic fatty liver disease. Crit. Rev. Food Sci. Nutr..

[23] Agognon A.L., Casertano M., Vito A., Orso S., Cabaro S., Mormone F., Morelli C., Perruolo G., Formisano P., Menna M. (2024). Marine-Derived Phosphoeleganin and Its Semisynthetic Derivative Decrease IL6 Levels and Improve Insulin Signaling in Human Hepatocellular Carcinoma Cells. Int. J. Mol. Sci..

[24] Casertano M., Genovese M., Piazza L., Balestri F., Del Corso A., Vito A., Paoli P., Santi A., Imperatore C., Menna M. (2022). Identifying human PTP1B enzyme inhibitors from marine natural products: Perspectives for developing of novel insulin-mimetic drugs. Pharmaceuticals.

[25] Böhm H.J., Banner D., Bendels S., Kansy M., Kuhn B., Müller K., Obst-Sander U., Stahl M. (2004). Fluorine in medicinal chemistry. ChemBioChem.

[26] Park B.K., Kitteringham N.R., O’Neill P.M. (2001). Metabolism of fluorine-containing drugs. Annu. Rev. Pharmacol. Toxicol..

[27] O’Hagan D., Rzepa H.S. (1997). Some influences of fluorine in bioorganic chemistry. Chem. Commun..

[28] Smart B.E. (2001). Fluorine substituent effects (on bioactivity). J. Fluor. Chem..

[29] Nair A.S., Singh A.K., Kumar A., Kumar S., Sukumaran S., Koyiparambath V.P., Pappachen L.K., Rangarajan T.M., Kim H., Mathew B. (2022). FDA-Approved Trifluoromethyl Group-Containing Drugs: A Review of 20 Years. Processes.

[30] Kim W., Li M., Jin H., Yang H., Türkez H., Uhlén M., Zhang C., Mardinoglu A. (2023). Characterization of an in vitro steatosis model simulating activated de novo lipogenesis in MAFLD patients. IScience.

[31] Heeren J., Scheja L. (2021). Metabolic-associated fatty liver disease and lipoprotein metabolism. Mol. Metab..

[32] Zhang C., Shi M., Kim W., Arif M., Klevstig M., Li X., Yang H., Bayram C., Bolat I., Tozlu Ö.Ö. (2022). Discovery of therapeutic agents targeting PKLR for NAFLD using drug repositioning. EBioMedicine.

[33] Godoy-Matos A.F., Júnior W.S.S., Valerio C.M. (2020). NAFLD as a continuum: From obesity to metabolic syndrome and diabetes. Diabetol. Metab. Syndr..

[34] Weernink P.A.O., Staal G.E.J., Rijksen G. (1997). Pyruvate Kinases. Encycl. Cancer.

[35] Gray L.R., Tompkins S.C., Taylor E.B. (2014). Regulation of pyruvate metabolism and human disease. Cell. Mol. Life Sci..

[36] Adams L.S., Zhang Y., Seeram N.P., Heber D., Chen S. (2010). Pomegranate ellagitannin-derived compounds exhibit antiproferative and antiaromatase activity in breast cancer cells In vitro. Cancer Prev. Res..

[37] Goudarzi M., Mombeini M.A., Fatemi I., Aminzadeh A., Kalantari H., Nesari A., Najafzadehvarzi H., Mehrzadi S. (2019). Neuroprotective effects of Ellagic acid against acrylamide-induced neurotoxicity in rats. Neurol. Res..

[38] Tomás-Barberán F.A., González-Sarrías A., García-Villalba R., Núñez-Sánchez M.A., Selma M.V., García-Conesa M.T., Espín J.C. (2017). Urolithins, the rescue of “old” metabolites to understand a “new” concept: Metabotypes as a nexus among phenolic metabolism, microbiota dysbiosis, and host health status. Mol. Nutr. Food Res..

[39] Cerdá B., Tomás-Barberán F.A., Espín J.C. (2005). Metabolism of antioxidant and chemopreventive ellagitannins from strawberries, raspberries, walnuts, and oak-aged wine in humans: Identification of biomarkers and individual variability. J. Agric. Food Chem..

[40] Friedman S.L., Neuschwander-Tetri B.A., Rinella M., Sanyal A.J. (2018). Mechanisms of NAFLD development and therapeutic strategies. Nat. Med..

[41] Browning J.D., Horton J.D. (2004). Molecular mediators of hepatic steatosis and liver injury. J. Clin. Investig..

[42] Lambert J.E., Ramos–Roman M.A., Browning J.D., Parks E.J. (2014). Increased de novo lipogenesis is a distinct characteristic of individuals with nonalcoholic fatty liver disease. Gastroenterology.

[43] Liu Z., Zhang C., Lee S., Kim W., Klevstig M., Harzandi A.M., Sikanic N., Arif M., Ståhlman M., Nielsen J. (2019). Pyruvate kinase L/R is a regulator of lipid metabolism and mitochondrial function. Metab. Eng..

[44] Yang W. (2015). Structural basis of PKM2 regulation. Protein Cell.

[45] Yang W., Lu Z. (2013). Nuclear PKM2 regulates the Warburg effect. Cell Cycle.

[46] Zahra K., Dey T., Ashish, Mishra S.P., Pandey U. (2020). Pyruvate kinase M2 and cancer: The role of PKM2 in promoting tumorigenesis. Front. Oncol..

[47] Liberti M.V., Locasale J.W. (2016). The Warburg effect: How does it benefit cancer cells?. Trends Biochem. Sci..

[48] Puckett D.L., Alquraishi M., Chowanadisai W., Bettaieb A. (2021). The role of PKM2 in metabolic reprogramming: Insights into the regulatory roles of non-coding RNAs. Int. J. Mol. Sci..

[49] Lu Q., Tian X., Wu H., Huang J., Li M., Mei Z., Zhou L., Xie H., Zheng S. (2021). Metabolic changes of hepatocytes in NAFLD. Front. Physiol..

[50] Palsson-McDermott E.M., Curtis A.M., Goel G., Lauterbach M.A., Sheedy F.J., Gleeson L.E., van den Bosch M.W., Quinn S.R., Domingo-Fernandez R., Johnston D.G. (2015). Pyruvate kinase M2 regulates Hif-1α activity and IL-1β induction and is a critical determinant of the warburg effect in LPS-activated macrophages. Cell Metab..

[51] Tang Y., Feng M., Su Y., Ma T., Zhang H., Wu H., Wang X., Shi S., Zhang Y., Xu Y. (2023). Jmjd4 facilitates Pkm2 degradation in cardiomyocytes and is protective against dilated cardiomyopathy. Circulation.

[52] Majumdar K.C., Mondal S. (2011). Recent developments in the synthesis of fused sultams. Chem. Rev..

[53] (2019). Molecular Operating Environment (MOE).

[54] Halgren T.A. (1996). Merck molecular force field. I. Basis, form, scope, parameterization, and performance of MMFF94. J. Comput. Chem..

