# Supplementary material for: Discovery of Cell-Permeable Allosteric Inhibitors of Liver Pyruvate Kinase: Design and Synthesis of Sulfone-Based Urolithins
Source: Int J Mol Sci. 2024 Jul 22;25(14):7986. doi: 10.3390/ijms25147986 (PMC11277446; doi:10.3390/ijms25147986)

SUPPORTING INFORMATION

Discovery of Cell-Permeable Allosteric Inhibitors of Liver Pyruvate Kinase: Design and Synthesis of Sulfone-Based Urolithins

Shazia Iqbal [1,†], Md. Zahidul Islam [1,†], Sajda Ashraf [1], Woonghee Kim [2], Amal A. AL-Sharabi [1,3], Mehmet Ozcan [4], Essam Hanashalshahaby [1], Cheng Zhang [2], Mathias Uhlén [2], Jan Boren [5], Hasan Turkez [6] and Adil Mardinoglu [2,7,*]

1 Trustlife Labs Drug Research & Development Center, 34774 Istanbul, Türkiye; shazia.iqbal@trustlifelabs.com (S.I.); zahidul.islam@trustlifelabs.com (M.Z.I.); sajda.ashraf@trustlifelabs.com (S.A.); amal.sharabi@trustlifelabs.com (A.A.A.-S.); essam.hanash@trustlifelabs.com (E.H.)
2 Science for Life Laboratory, KTH-Royal Institute of Technology, SE-17121 Stockholm, Sweden; woonghee.kim@scilifelab.se (W.K.); cheng.zhang@scilifelab.se (C.Z.); mathias.uhlen@scilifelab.se (M.U.)
3 Department of Pharmaceutical Chemistry, Faculty of Pharmacy, Anadolu University, 26470 Eskişehir, Türkiye
4 Department of Medical Biochemistry, Faculty of Medicine, Zonguldak Bulent Ecevit University, Zonguldak 67100, Türkiye; m.ozcan@beun.edu.tr
5 Department of Molecular and Clinical Medicine, University of Gothenburg, Sahlgrenska University Hospital, 413 45 Gothenburg, Sweden; jan.boren.wlab@gmail.com
6 Department of Medical Biology, Faculty of Medicine, Atatürk University, 25240 Erzurum, Türkiye; hasanturkez@gmail.com
7 Centre for Host-Microbiome Interactions, Faculty of Dentistry, Oral & Craniofacial Sciences, King's College London, London SE1 9RT, UK
* Correspondence: adilm@scilifelab.se
† These authors contributed equally to this work.

Content

Biological Data……………………….……………….. ………….……………...……………………S2

NMR Spectra of the precursors and final compounds.…………….. ………………...…………S3-S64

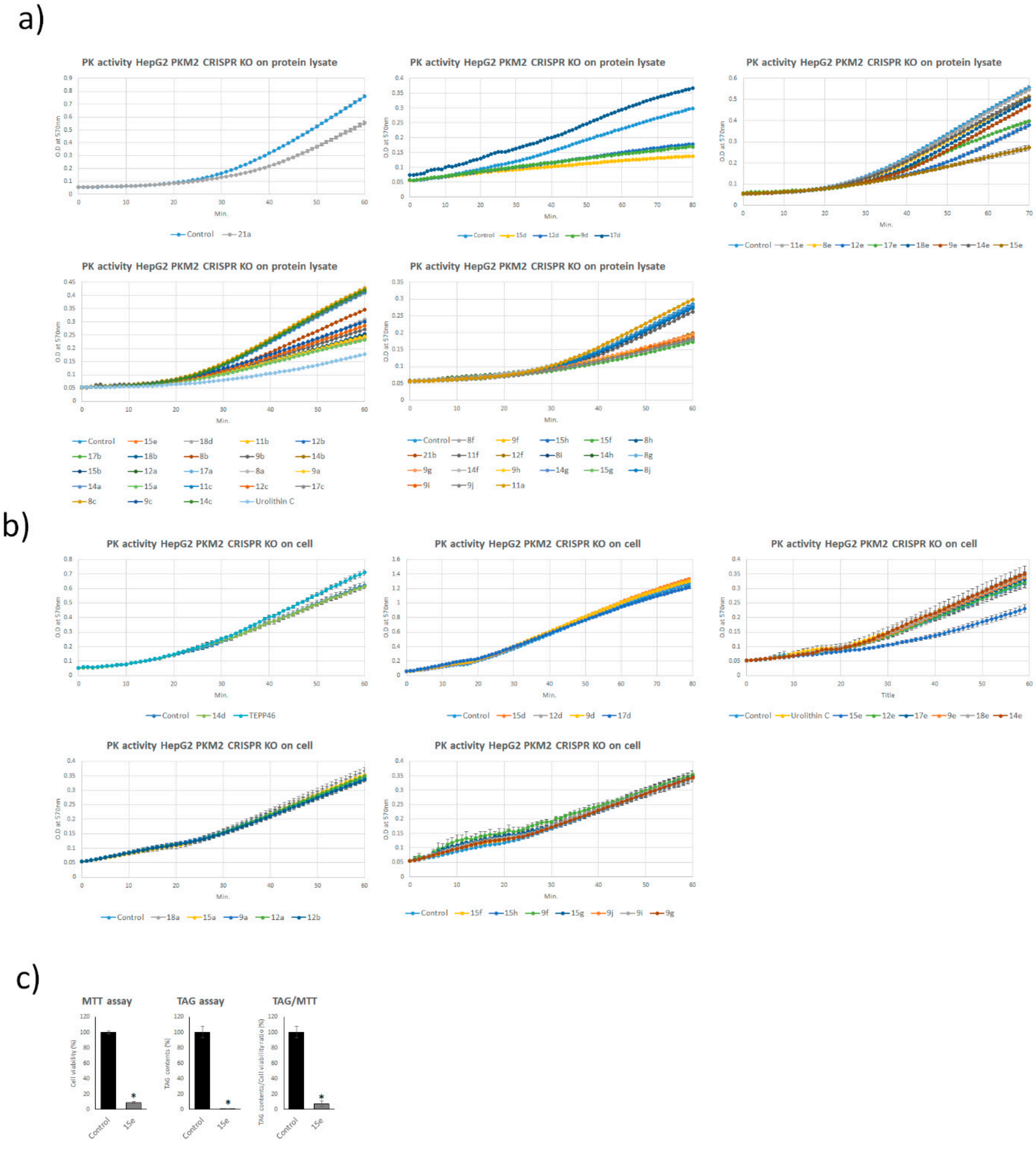

Supplementary Figure S1. a) Pyruvate kinase activity assay for Urolithin C and derivatives on HepG2 KO cells protein lysate at 10μM. b) Pyruvate kinase activity assay for Urolithin C and derivatives on HepG2 KO cells at 20μM. TEPP46 was treated as a positive control. C) TAG assay

and MTT assay for 10μM 15e treated HepG2 DNL steatosis model for 1week. Data are represented as mean ± SD, *p < 0.05, Student's t test.

2-bromo-4,5-dimethoxybenzenesulfonyl chloride ***(2)****:*

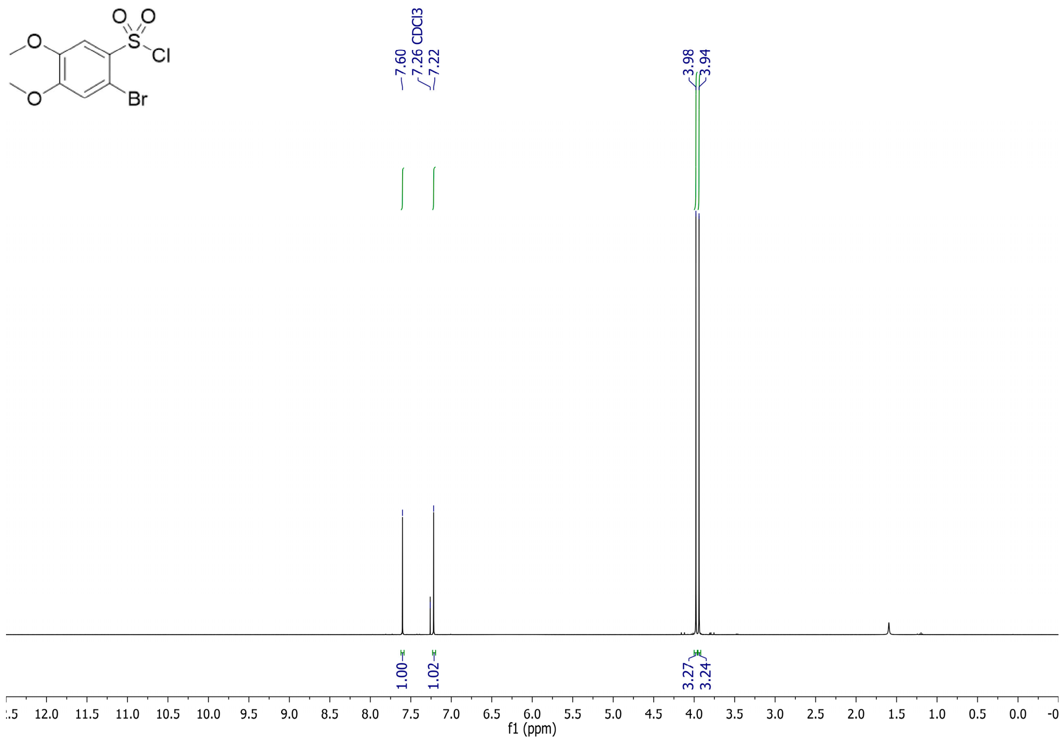

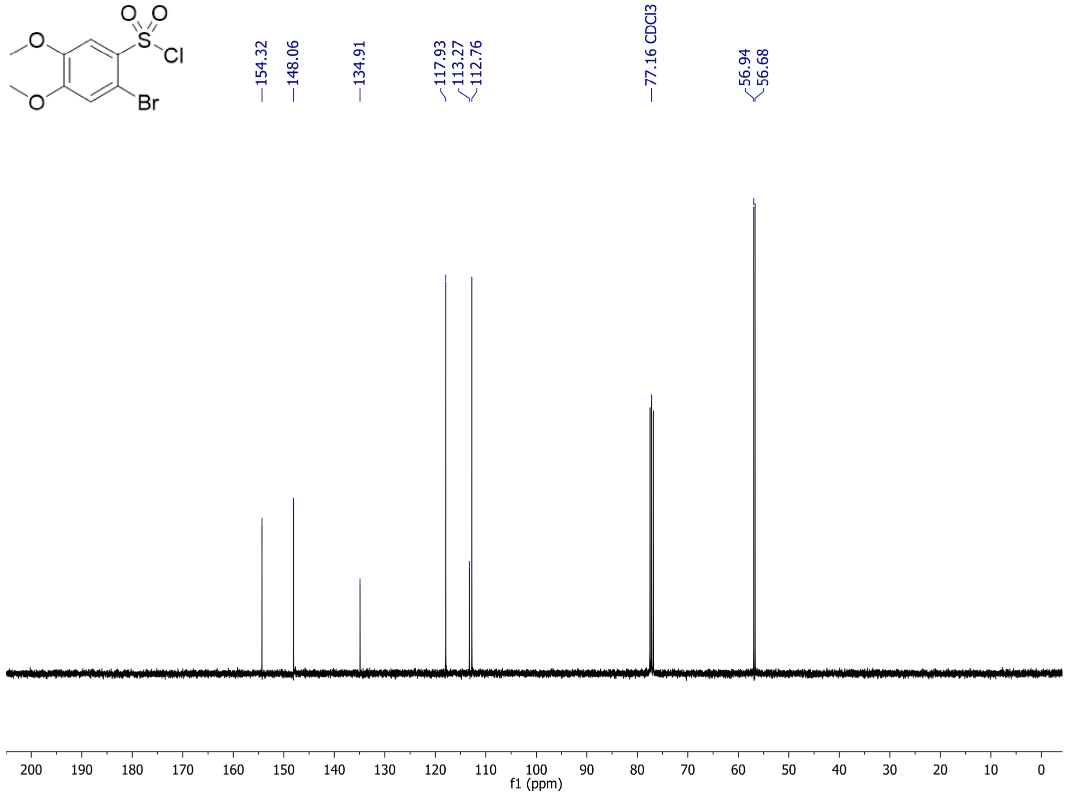

N-benzyl-2-bromo-4,5-dimethoxybenzenesulfonamide ***(7)***

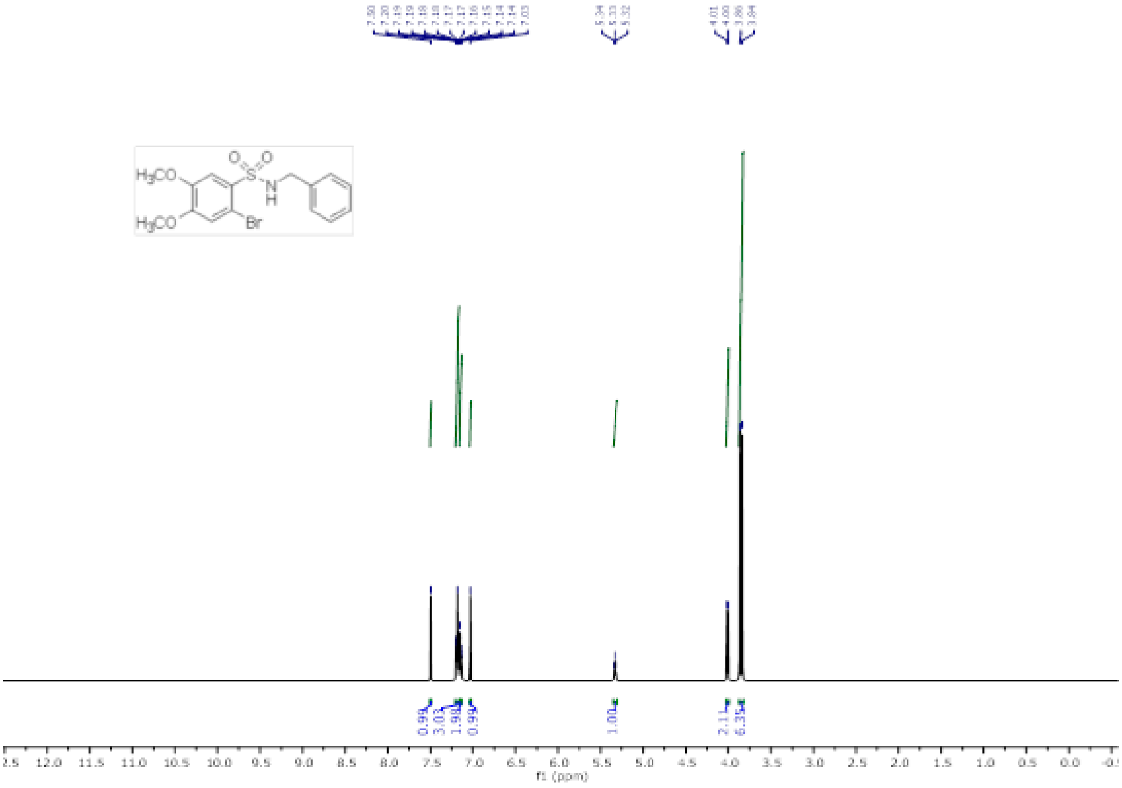

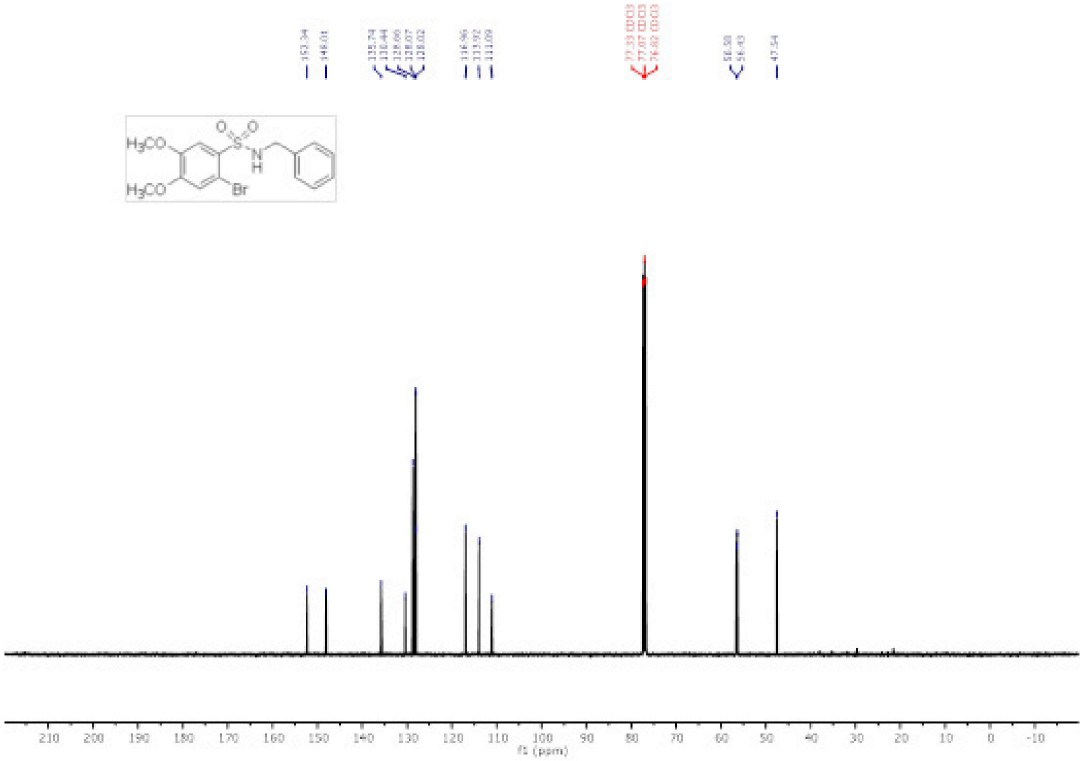

N-benzyl-3',4,5-trimethoxy-[1,1'-biphenyl]-2-sulfonamide ***(8a)****:*

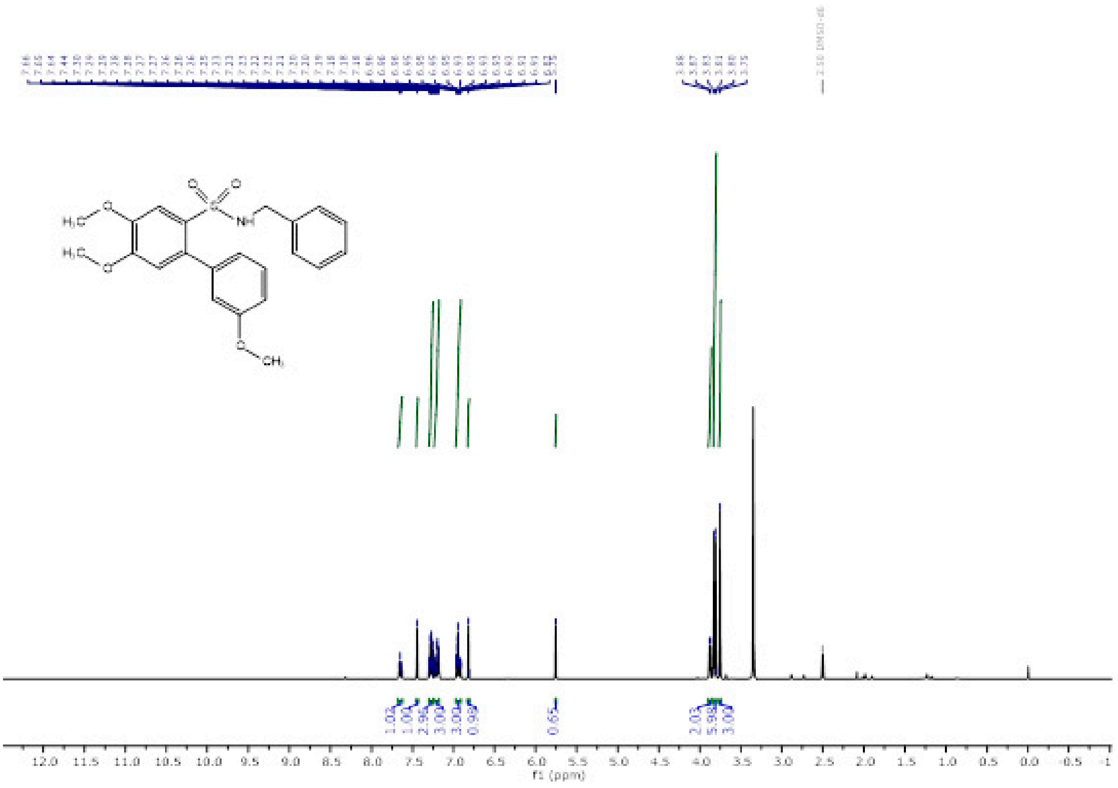

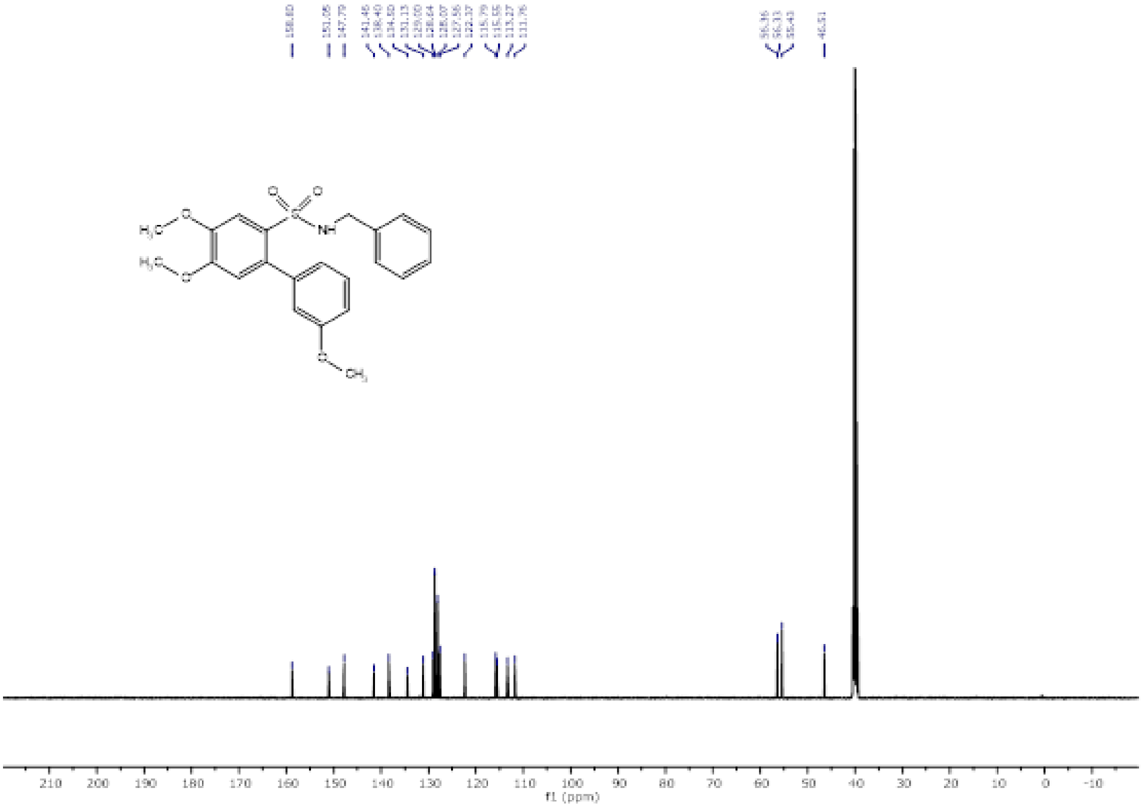

*N-benzyl-4,4',5-trimethoxy-[1,1'-biphenyl]-2-sulfonamide **(8b)**:*

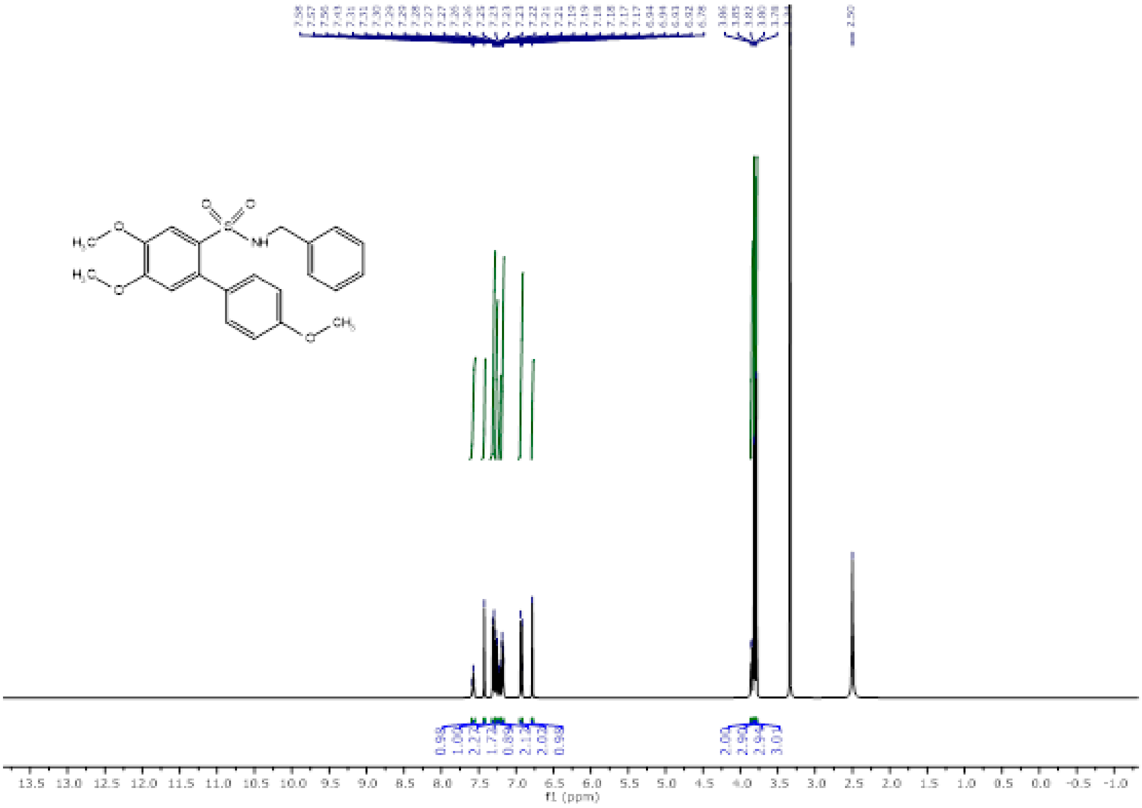

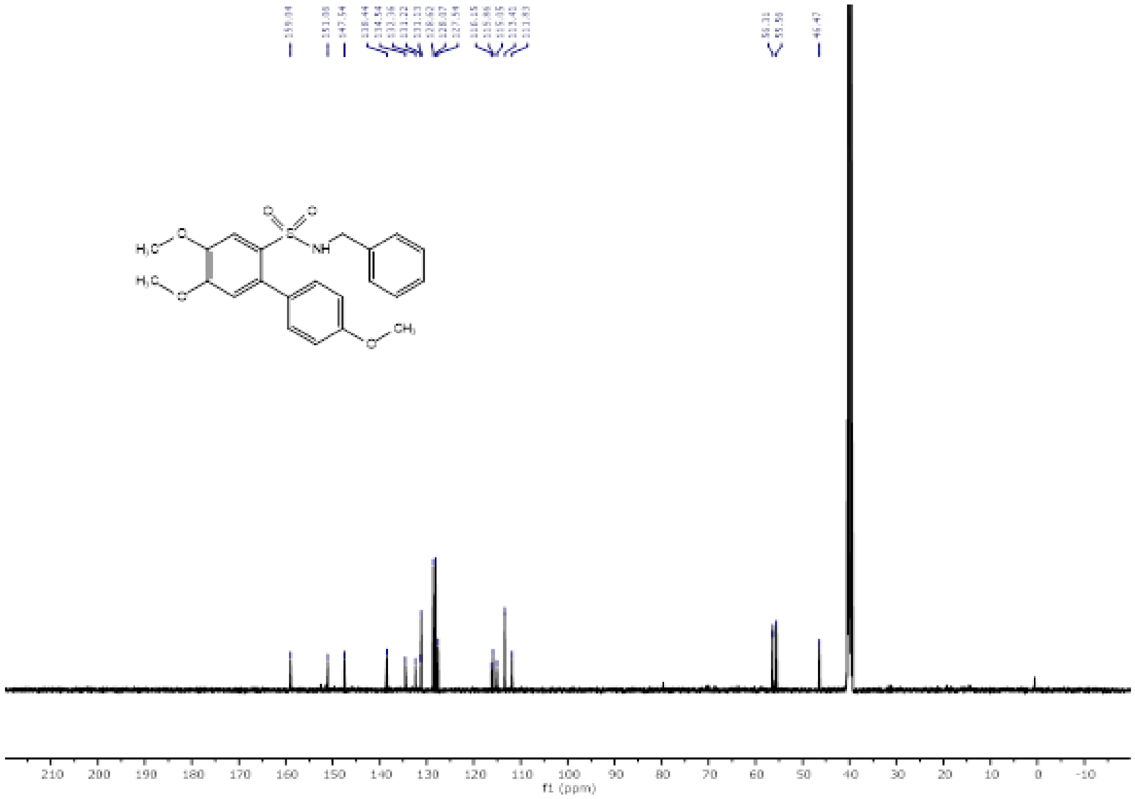

N-benzyl-3',4,5,5'-tetramethoxy-[1,1'-biphenyl]-2-sulfonamide ***(8c)****:*

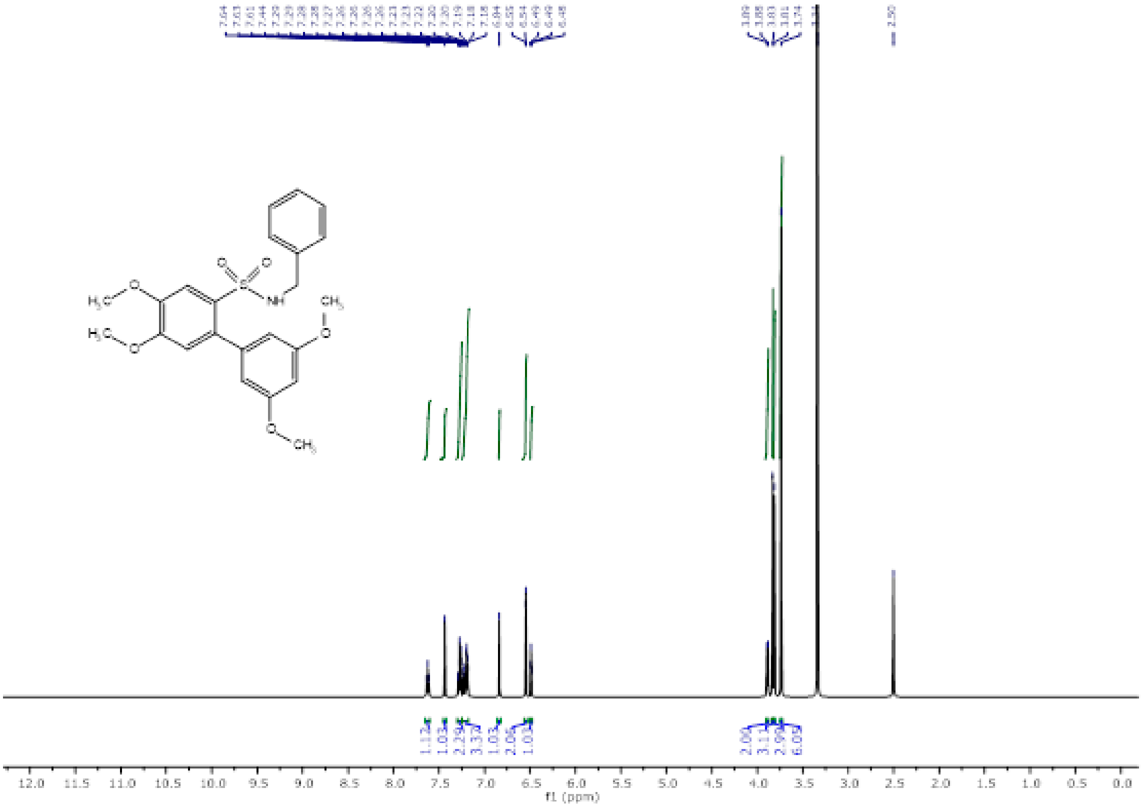

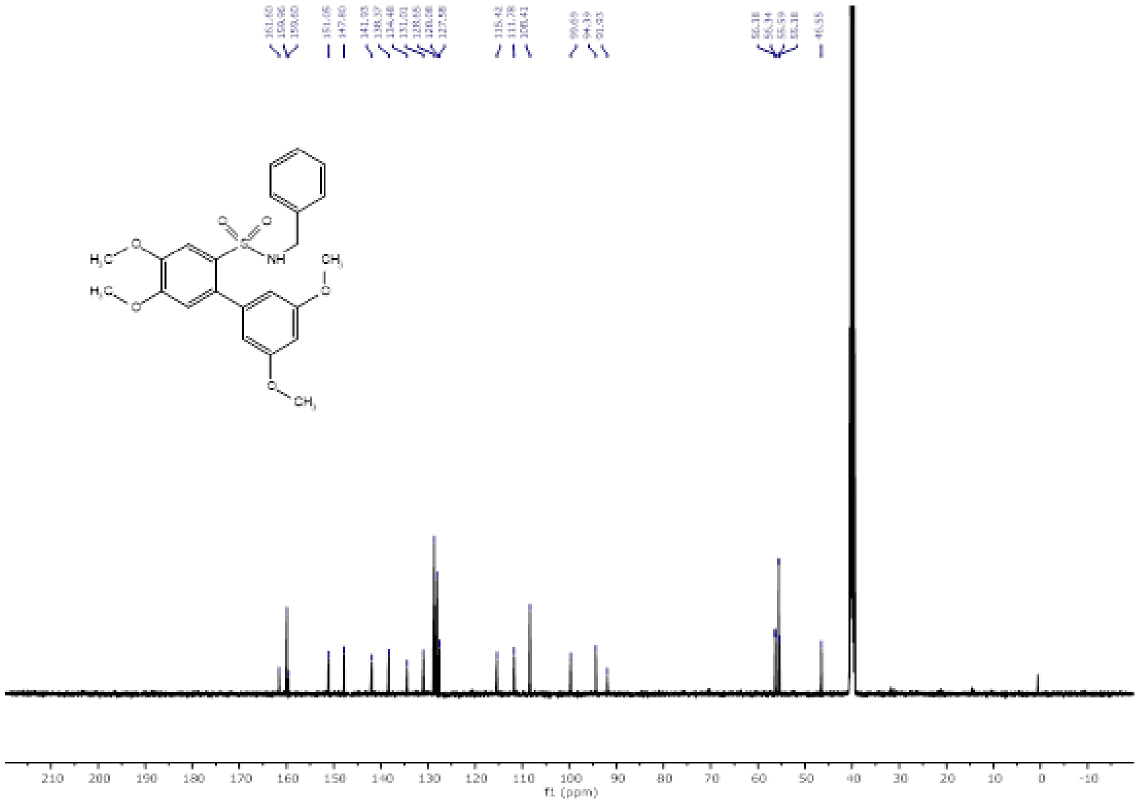

N-benzyl-3',4,4',5-tetramethoxy-[1,1'-biphenyl]-2-sulfonamide ***(8d)***

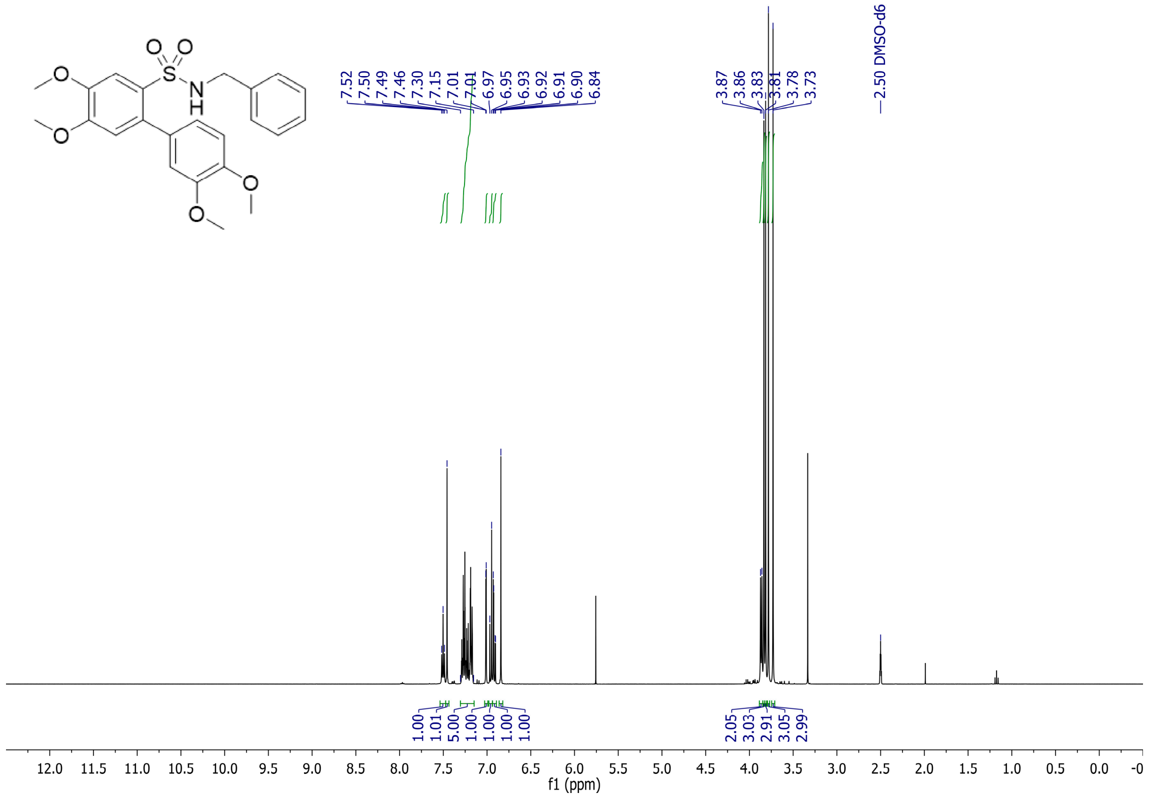

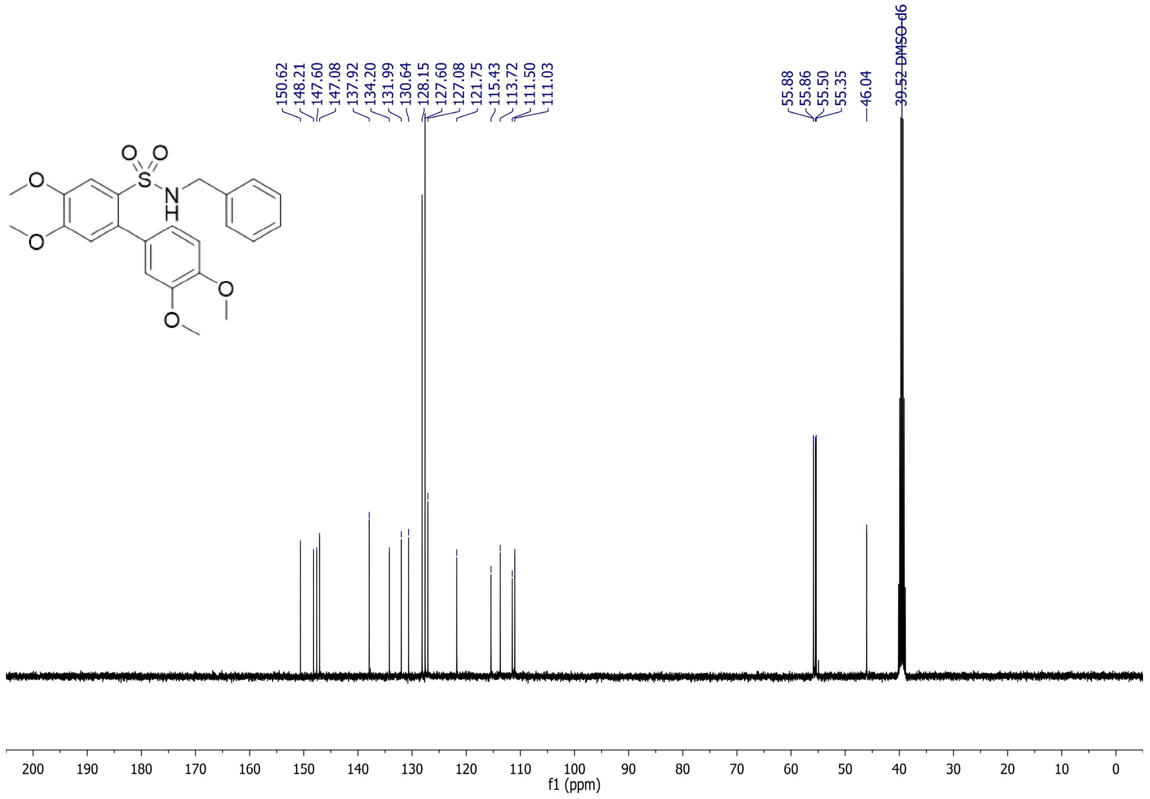

N-benzyl-4,5-dimethoxy-4'-(trifluoromethyl)-[1,1'-biphenyl]-2-sulfonamide ***(8e)***:

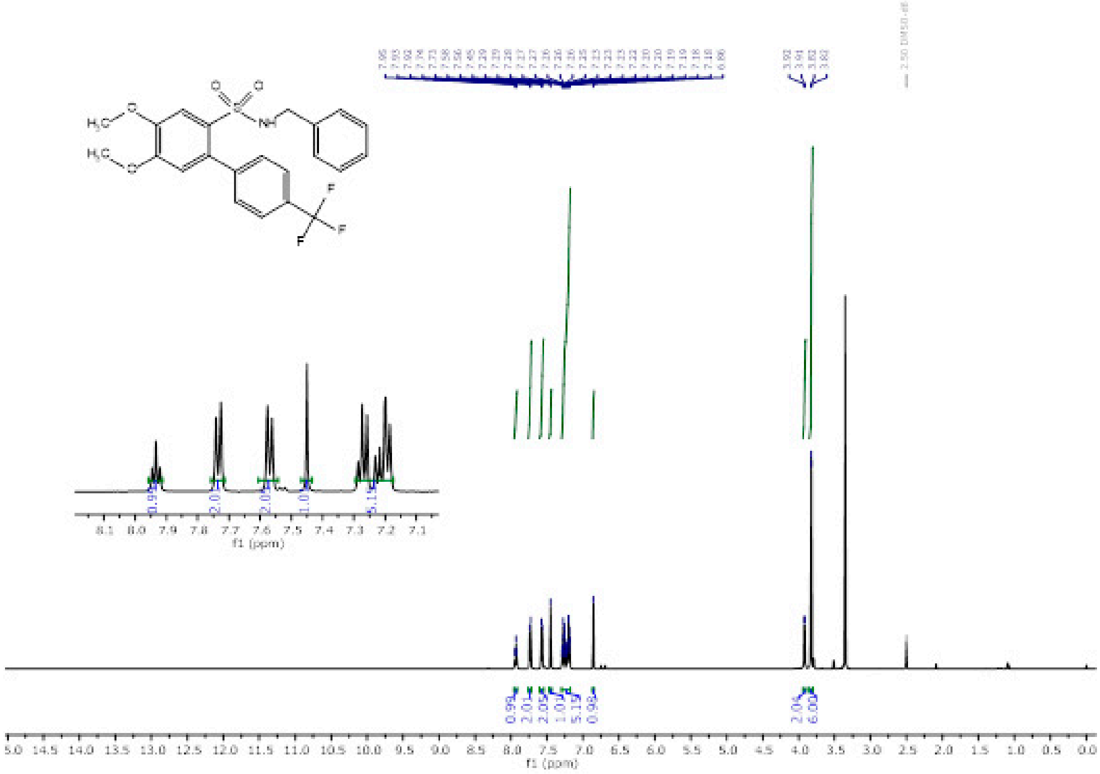

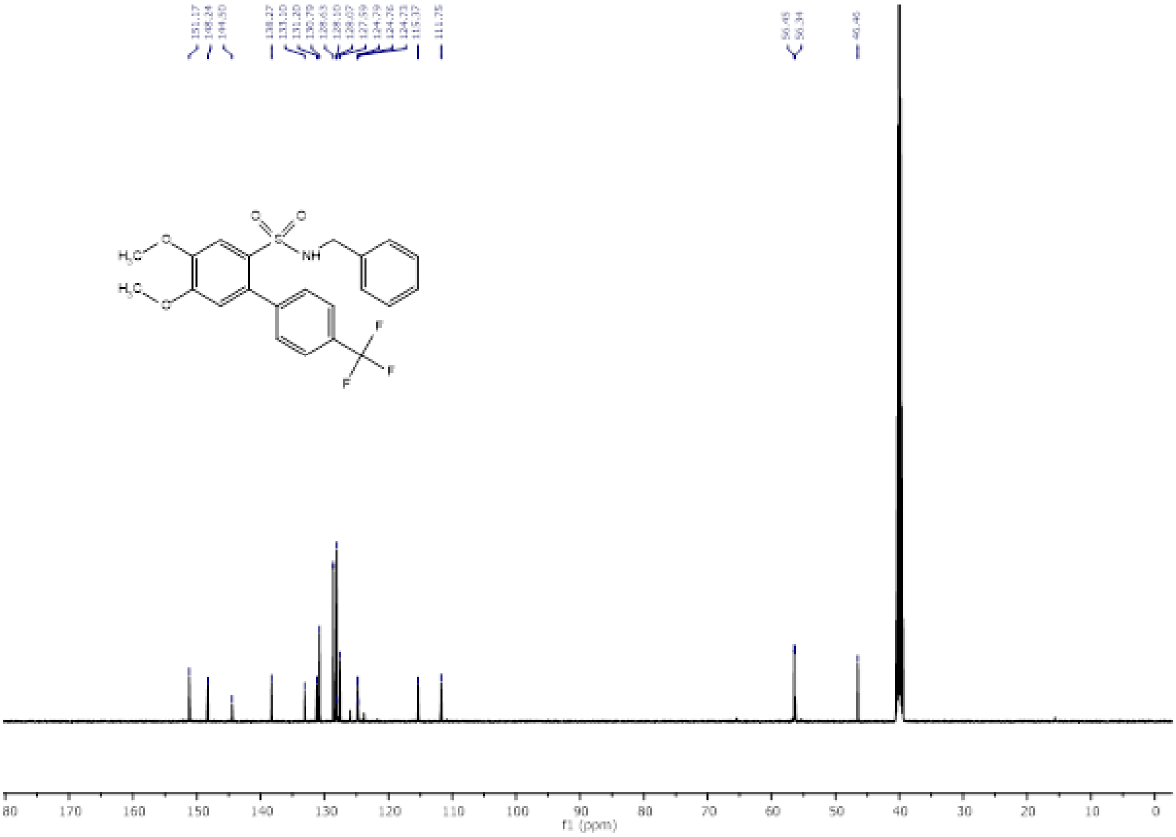

N-benzyl-4,5-dimethoxy-3',5'-bis(trifluoromethyl)-[1,1'-biphenyl]-2-sulfonamide ***(8f)***:

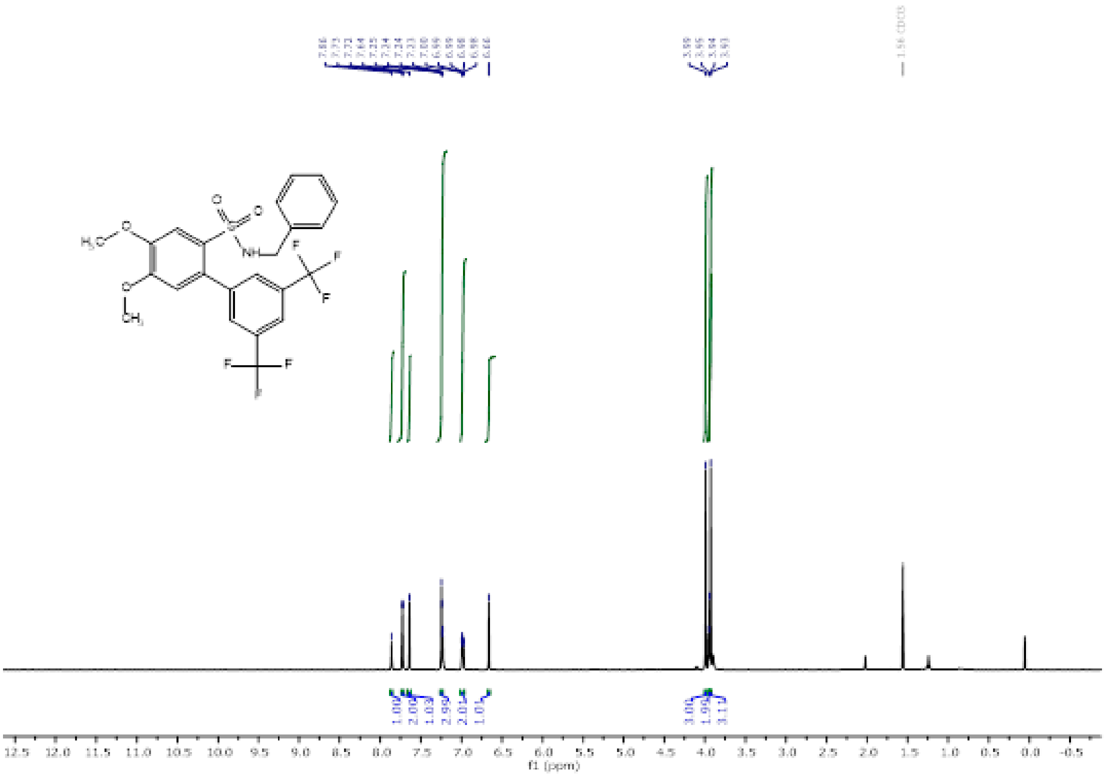

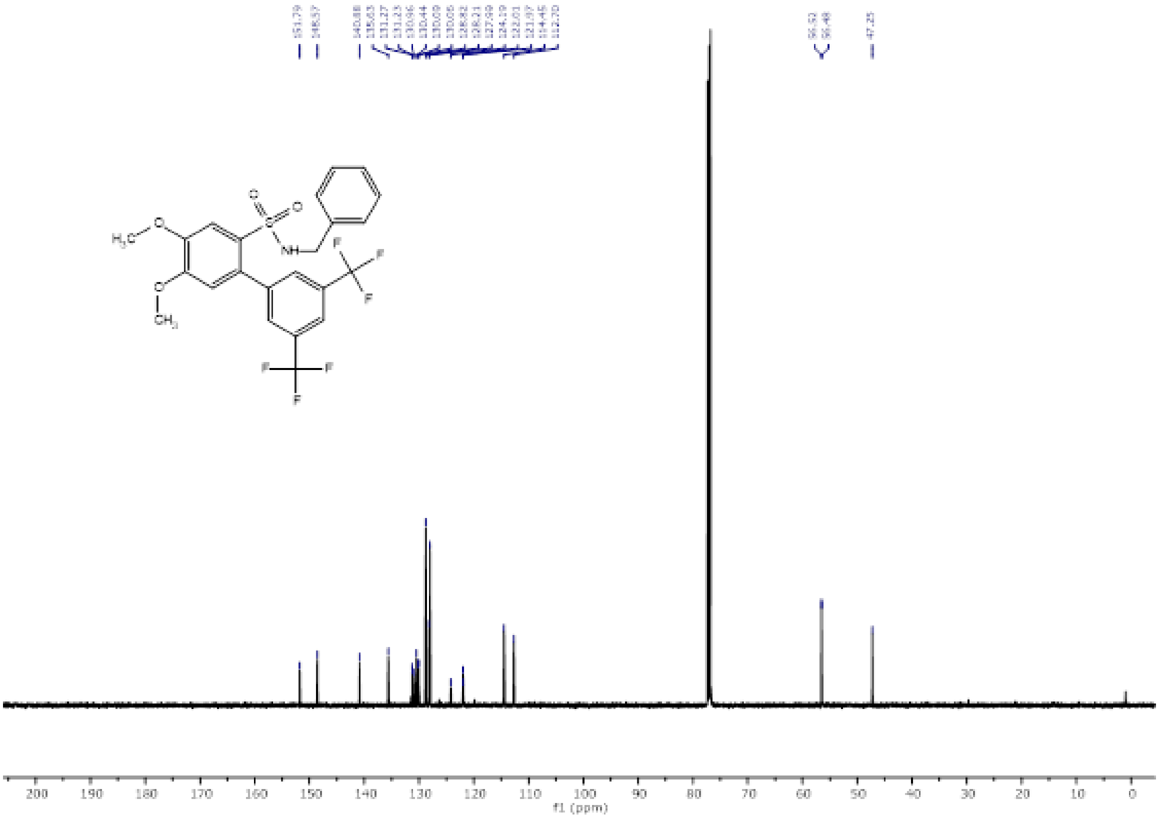

N-benzyl-3'-fluoro-4,4',5-trimethoxy-[1,1'-biphenyl]-2-sulfonamide ***(8g)***:

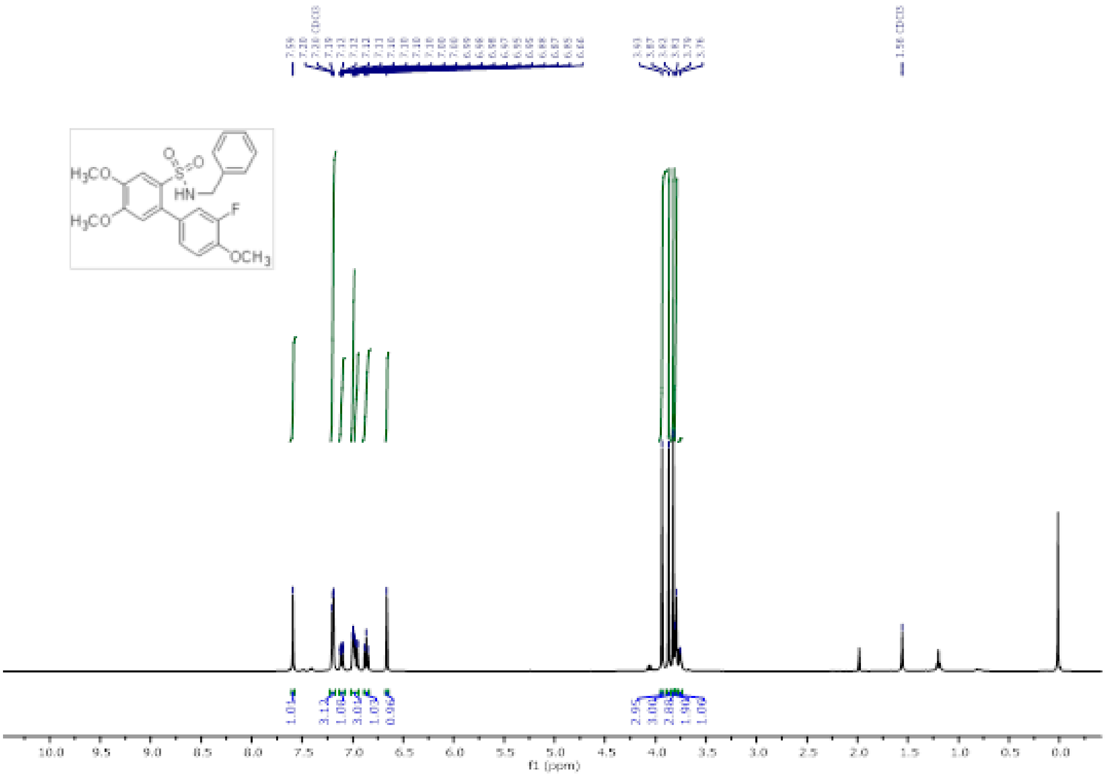

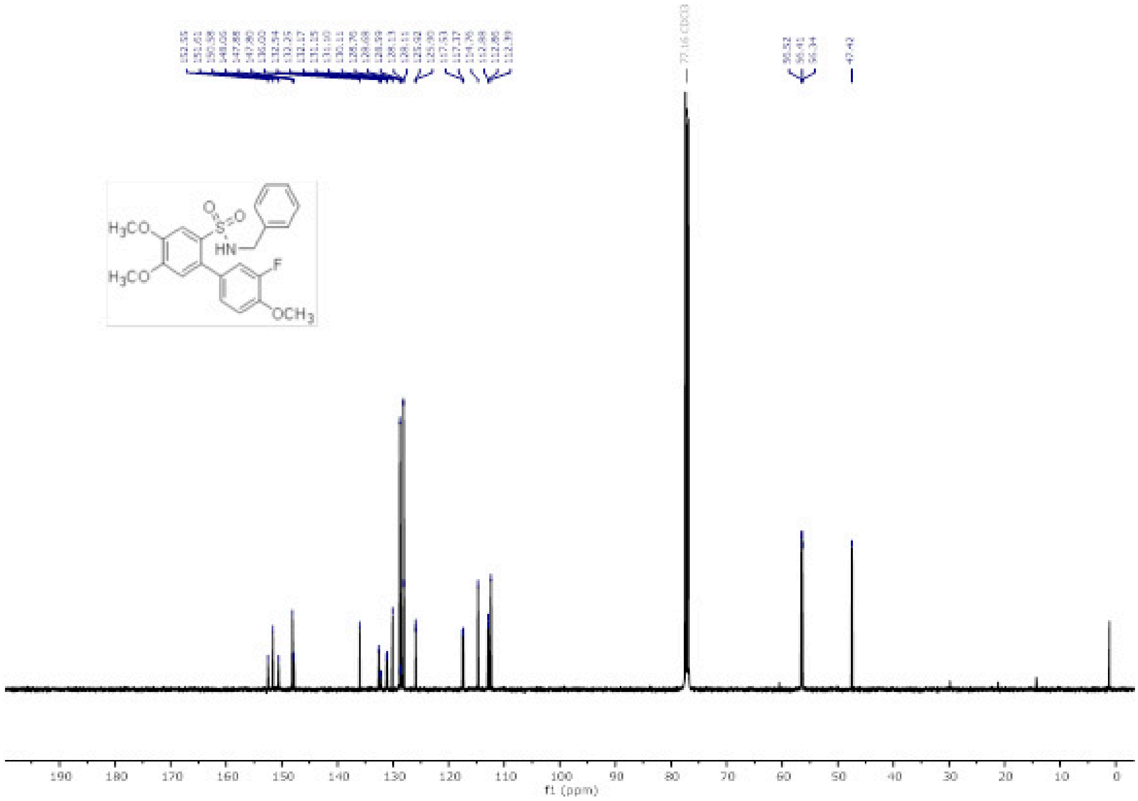

N-benzyl-4,4',5-trimethoxy-3'-(trifluoromethyl)-[1,1'-biphenyl]-2-sulfonamide ***(8h)****:*

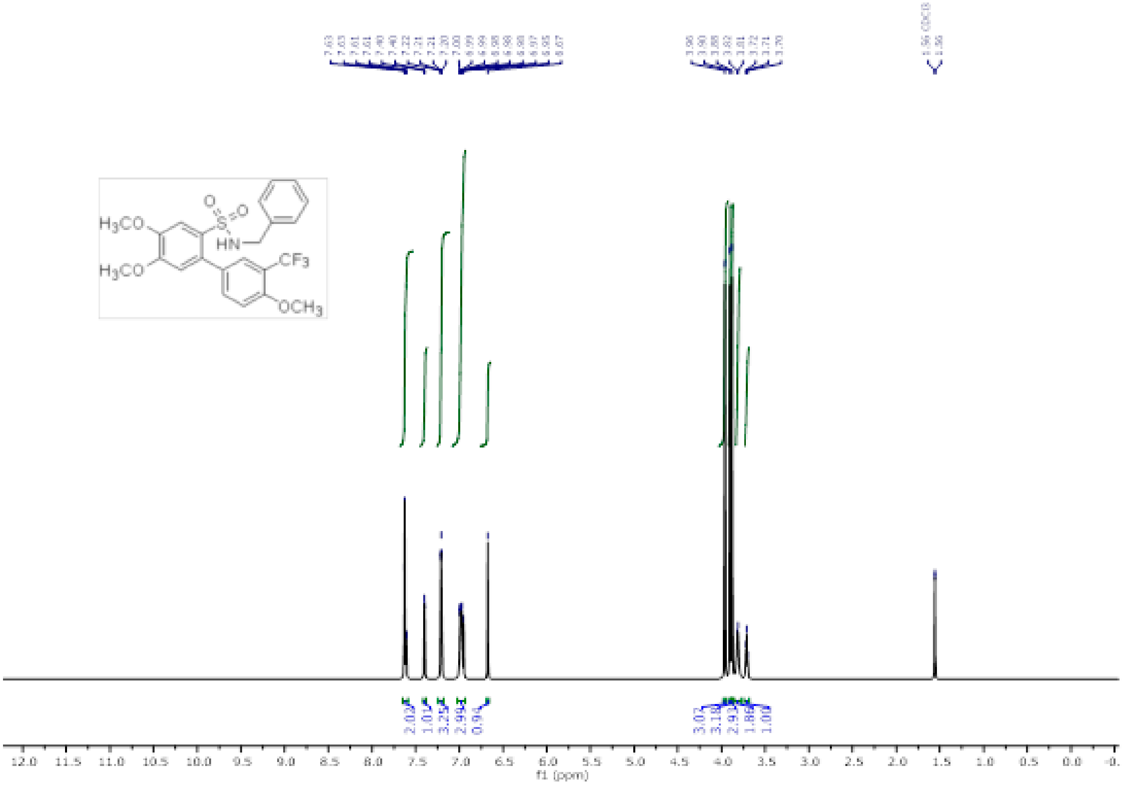

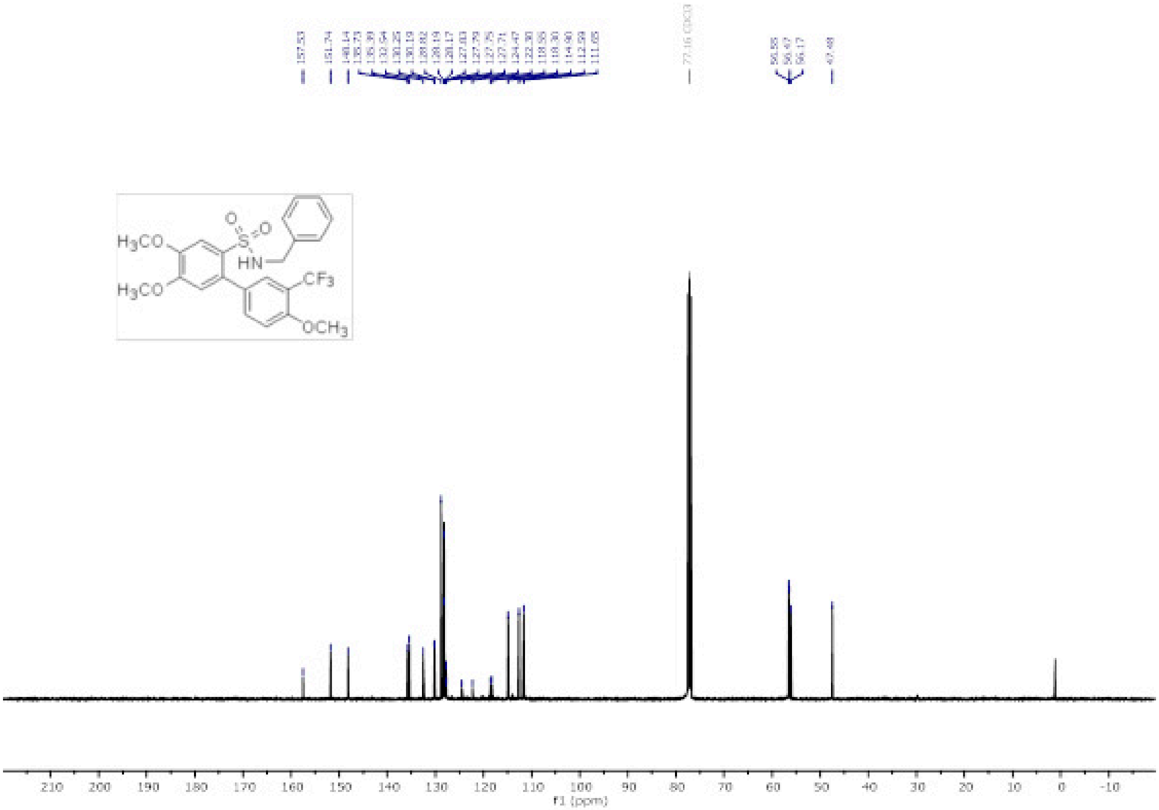

N-benzyl-4,5-dimethoxy-4'-(trifluoromethoxy)-[1,1'-biphenyl]-2-sulfonamide ***(8i)****:*

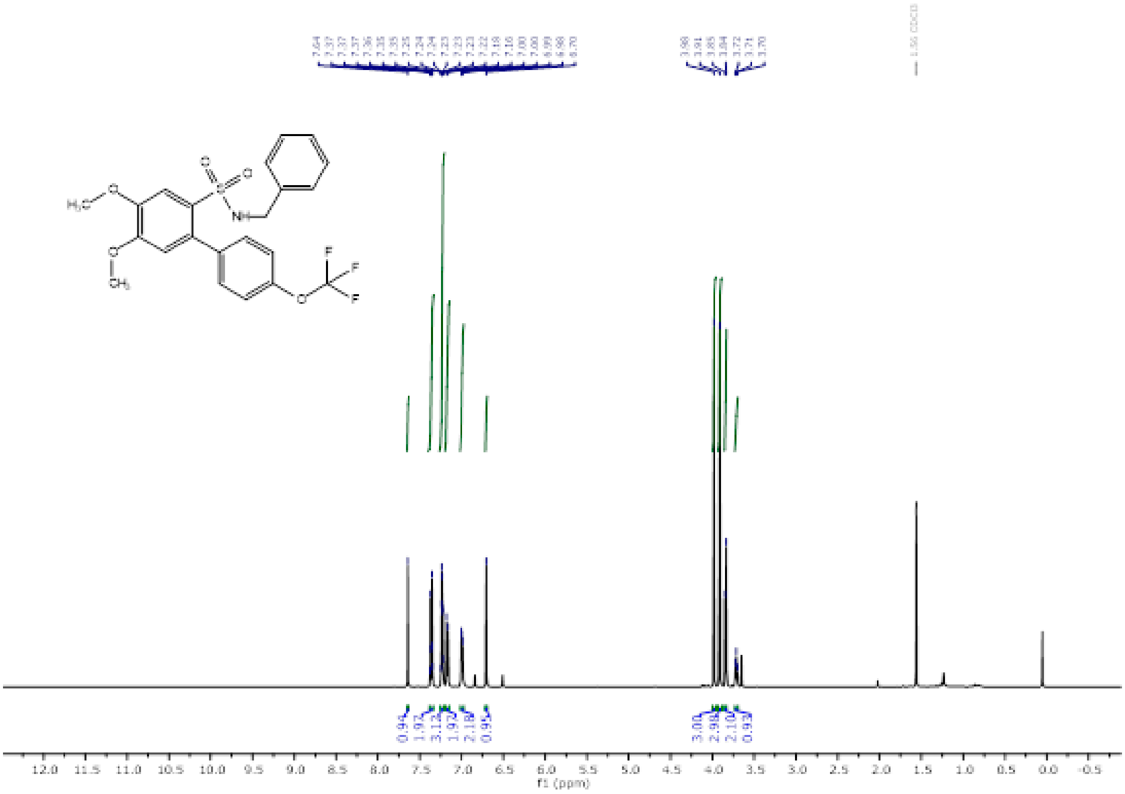

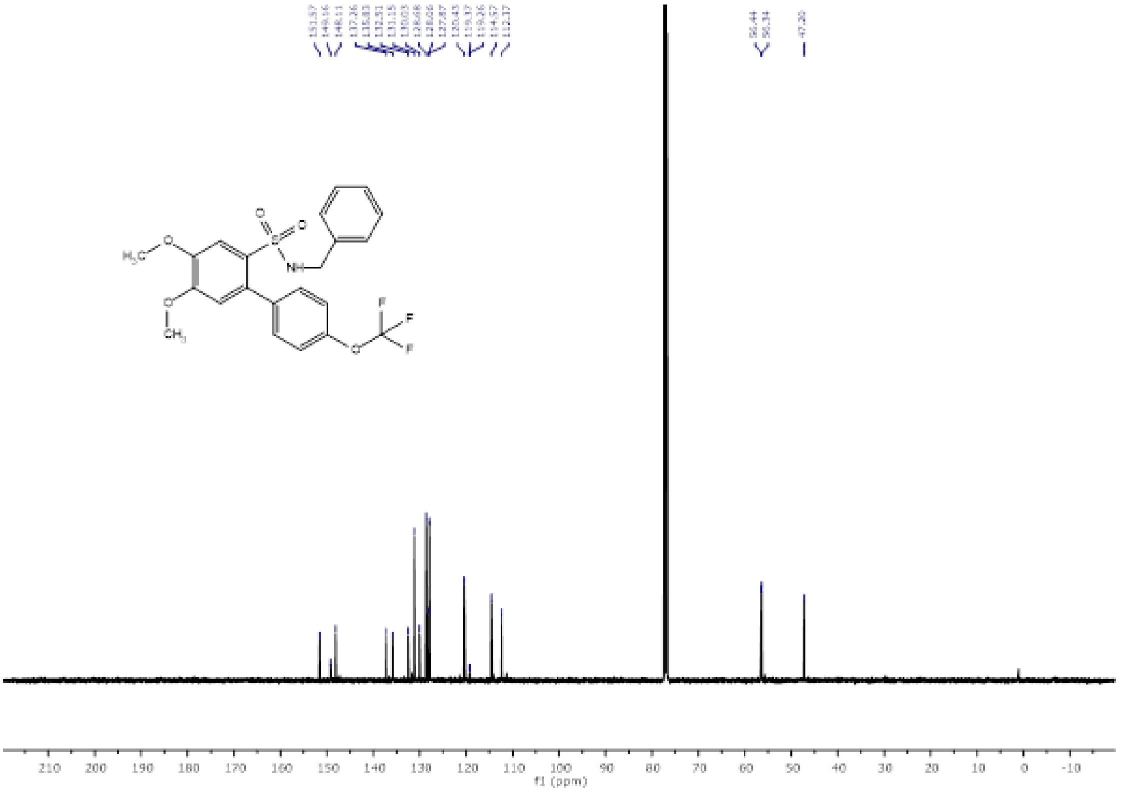

N-benzyl-2-(6-chloro-2-methoxypyridin-3-yl)-4,5-dimethoxybenzenesulfonamide ***(8j)***:

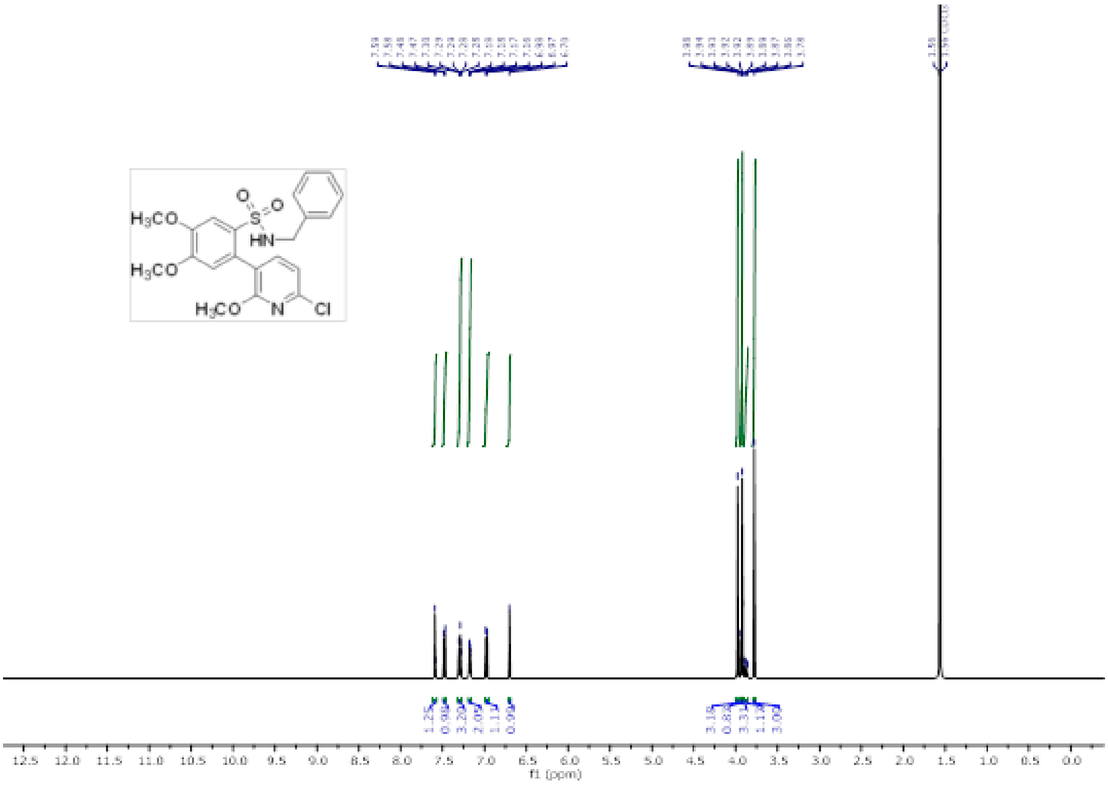

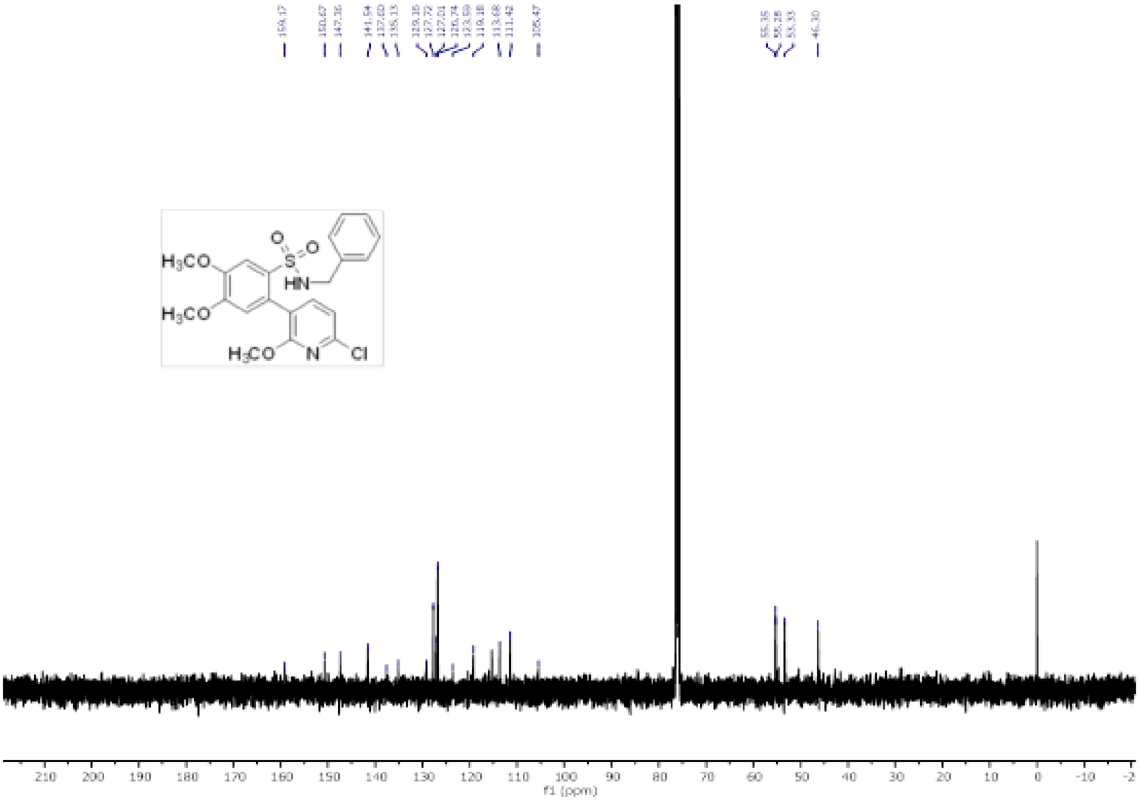

N-benzyl-3',4,5-trihydroxy-[1,1'-biphenyl]-2-sulfonamide ***(9a)***:

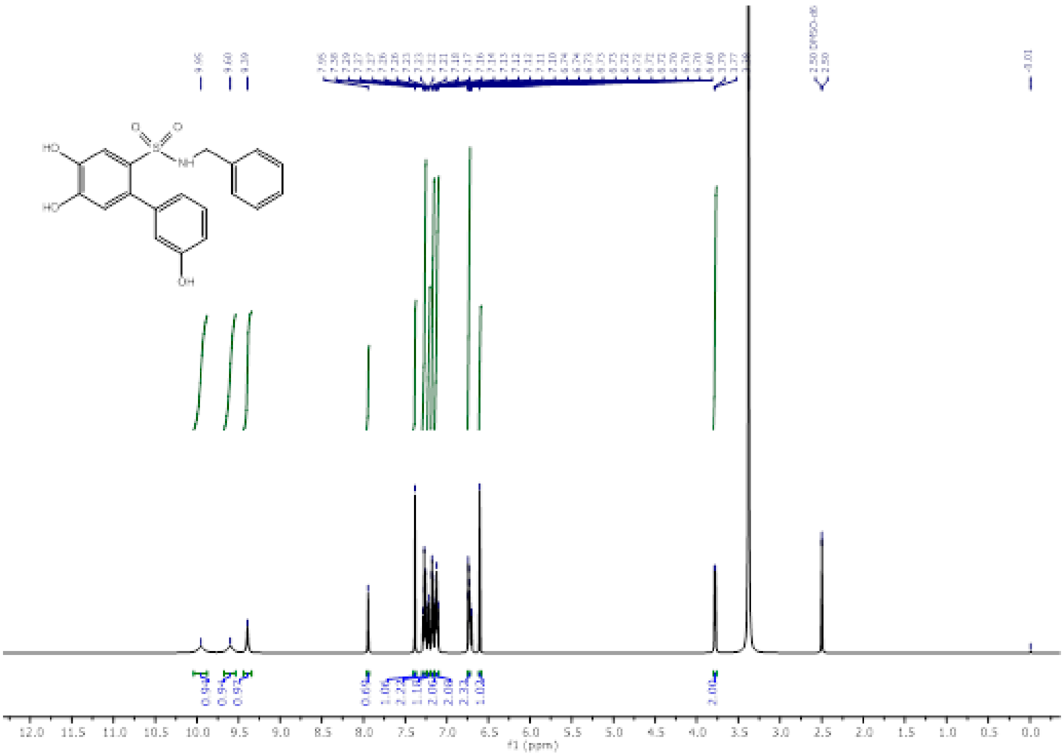

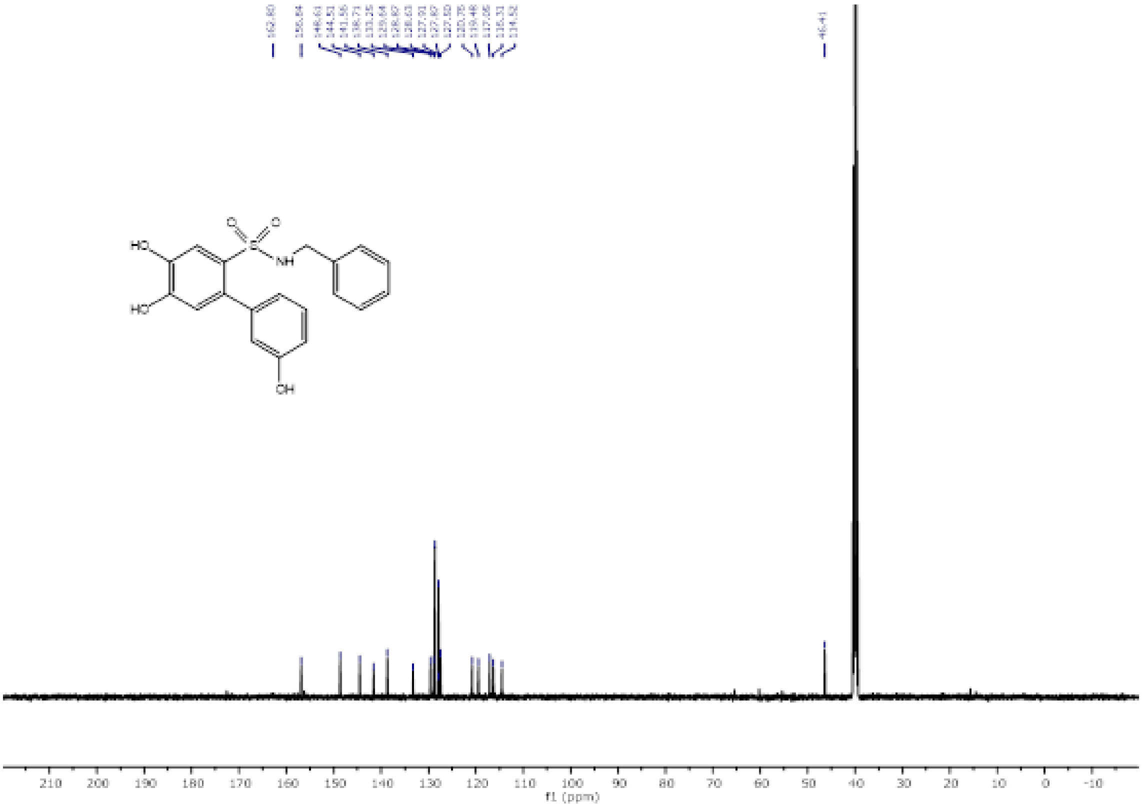

N-benzyl-4,4',5-trihydroxy-[1,1'-biphenyl]-2-sulfonamide ***(9b)***:

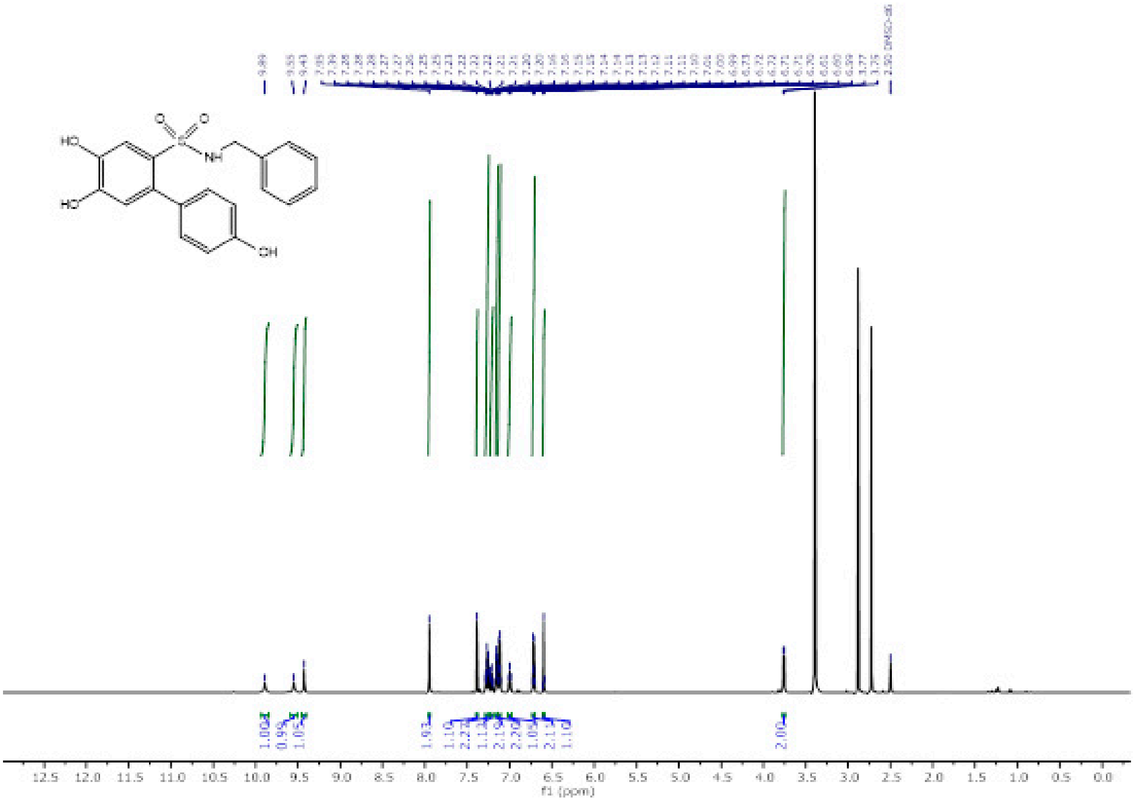

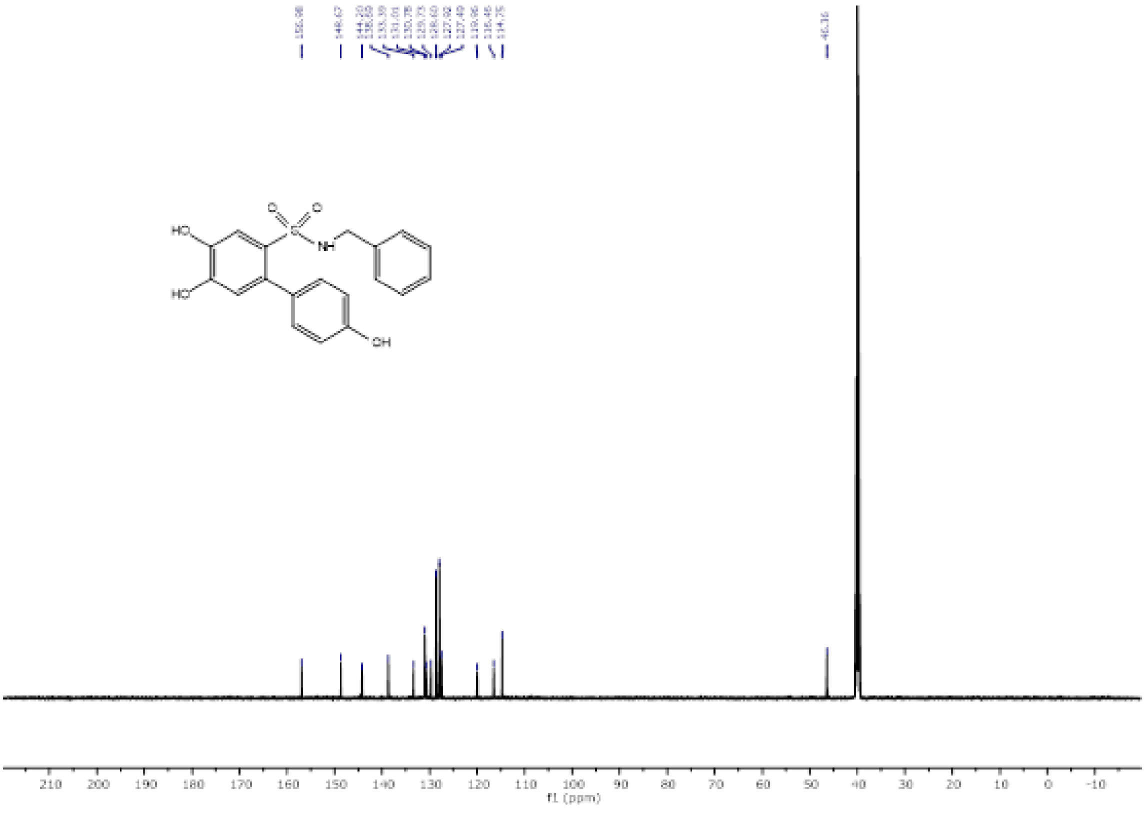

N-benzyl-3',4,5,5'-tetrahydroxy-[1,1'-biphenyl]-2-sulfonamide ***(9c)****:*

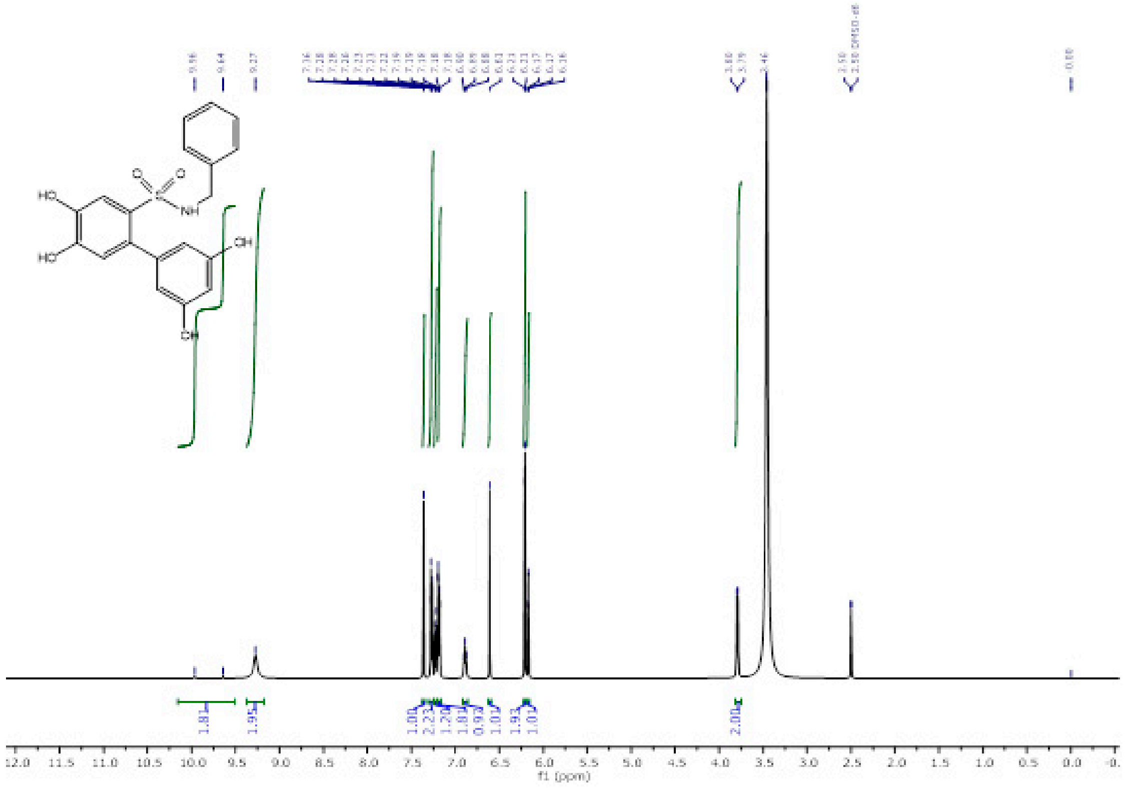

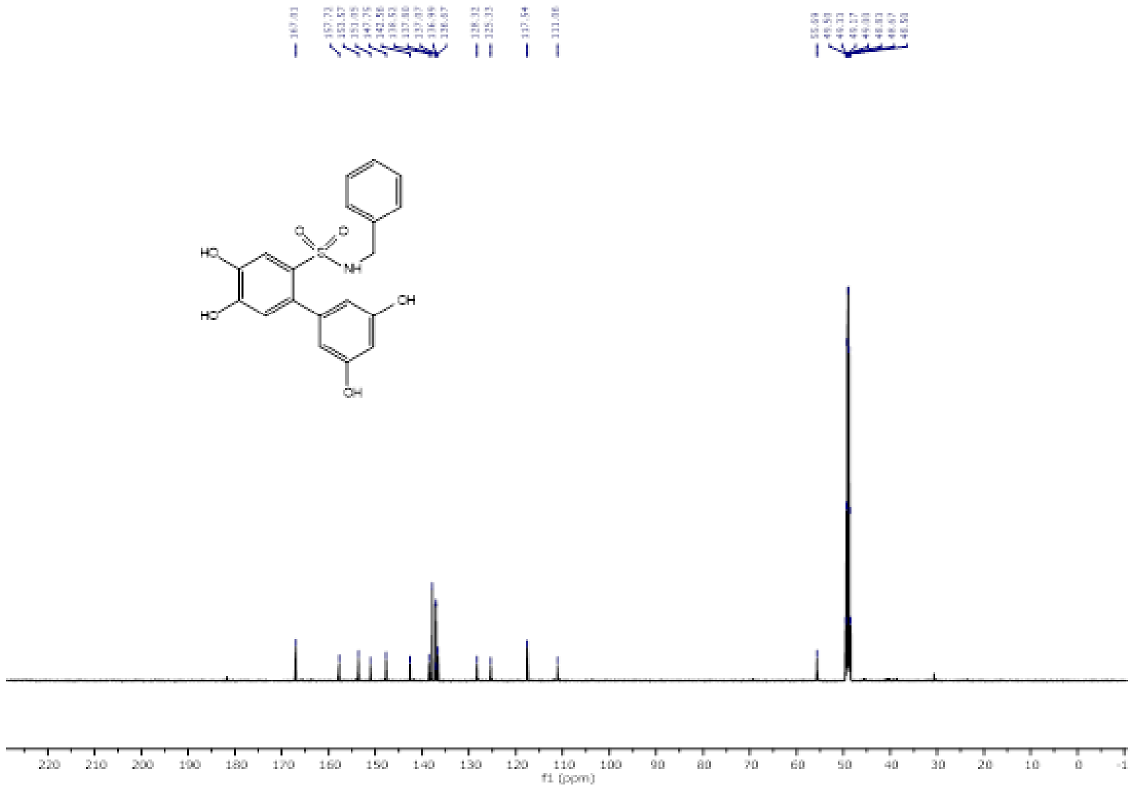

N-benzyl-3',4,4',5-tetrahydroxy-[1,1'-biphenyl]-2-sulfonamide ***(9d)***:

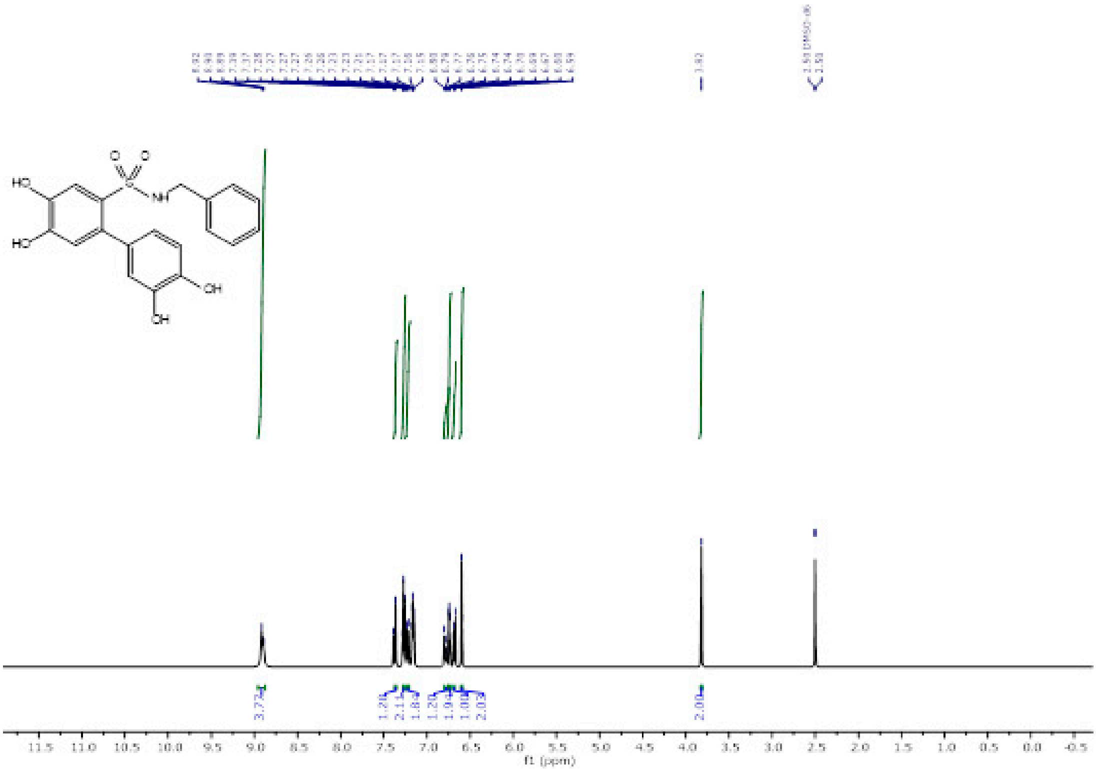

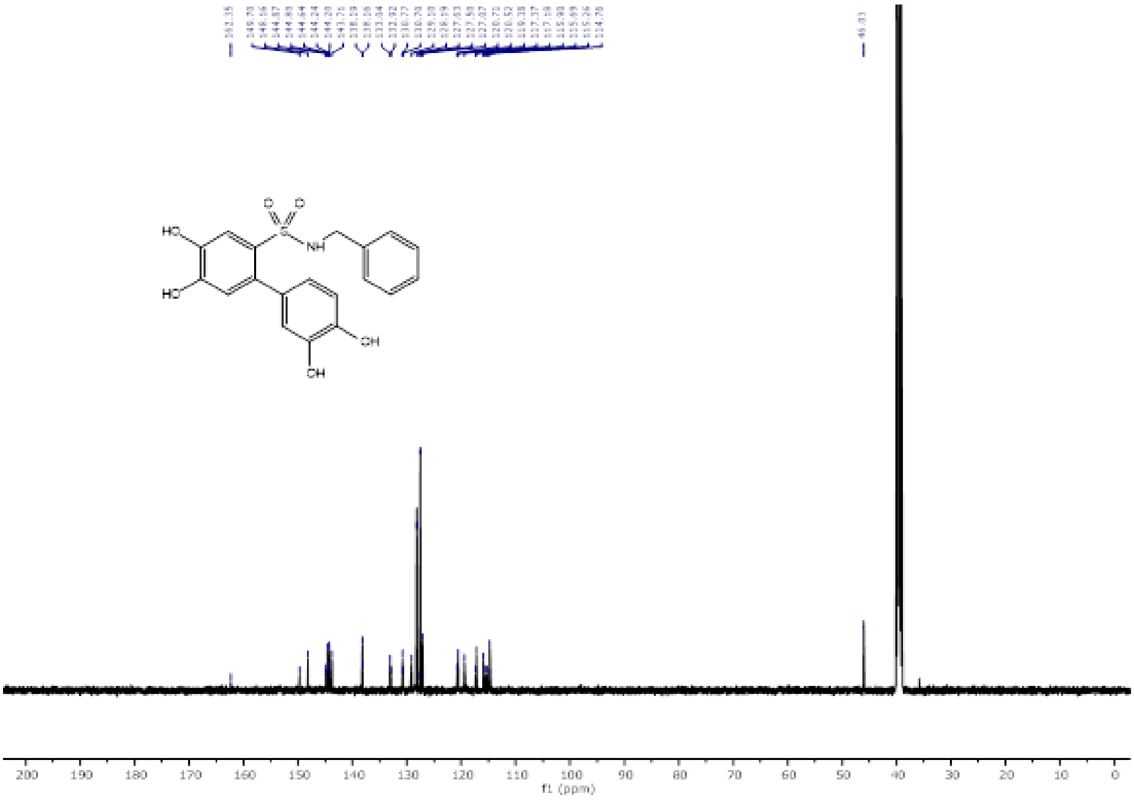

N-benzyl-4,5-dihydroxy-4'-(trifluoromethyl)-[1,1'-biphenyl]-2-sulfonamide ***(9e):***

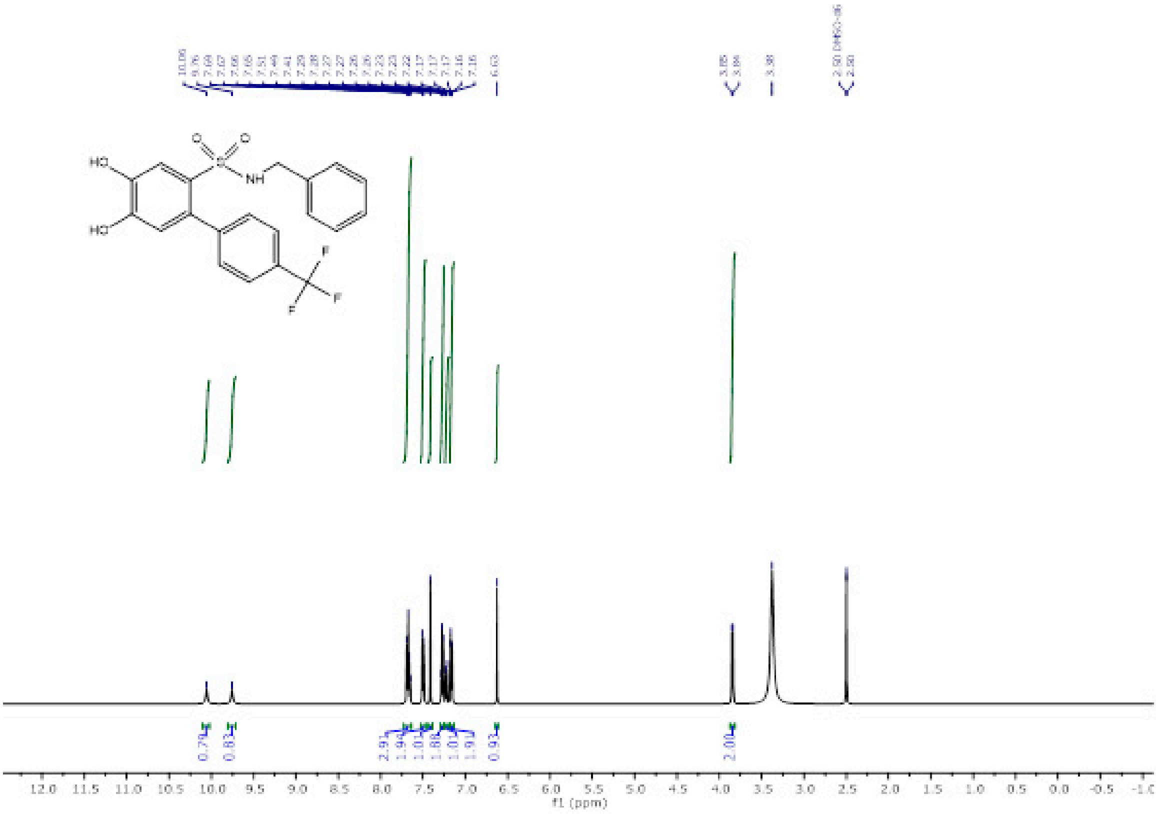

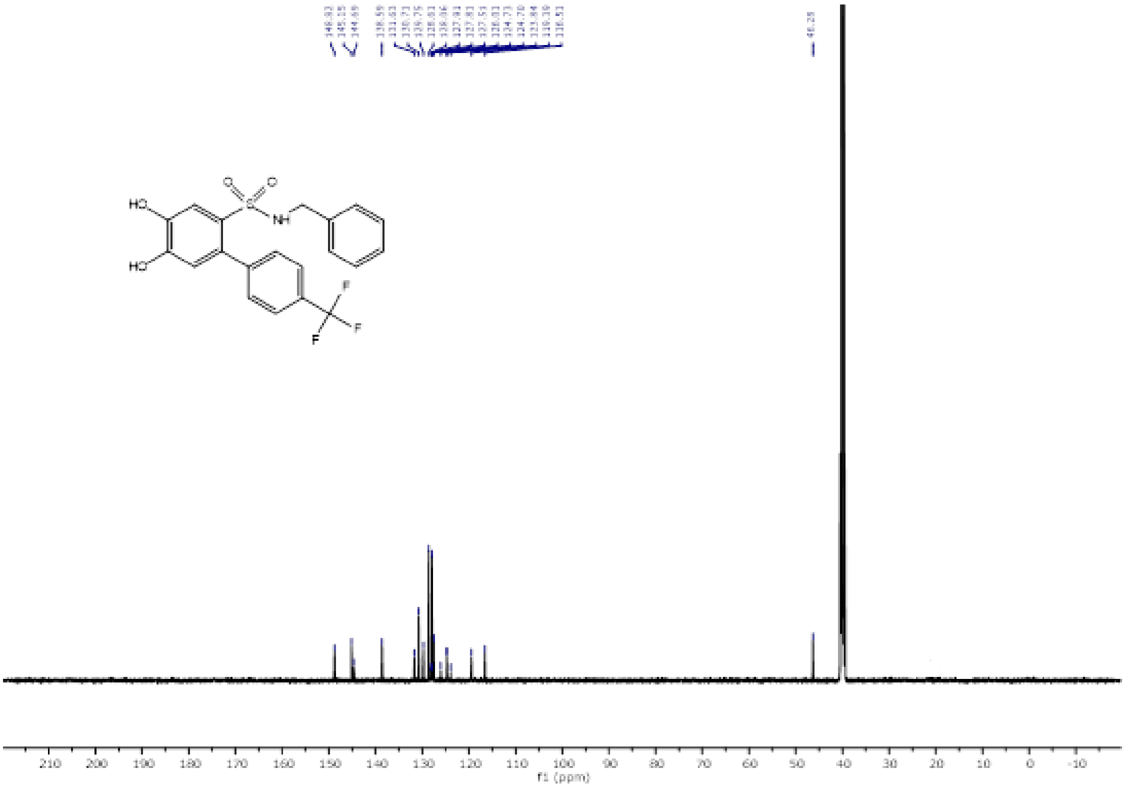

N-benzyl-4,5-dihydroxy-3',5'-bis(trifluoromethyl)-[1,1'-biphenyl]-2-sulfonamide (***9f***):

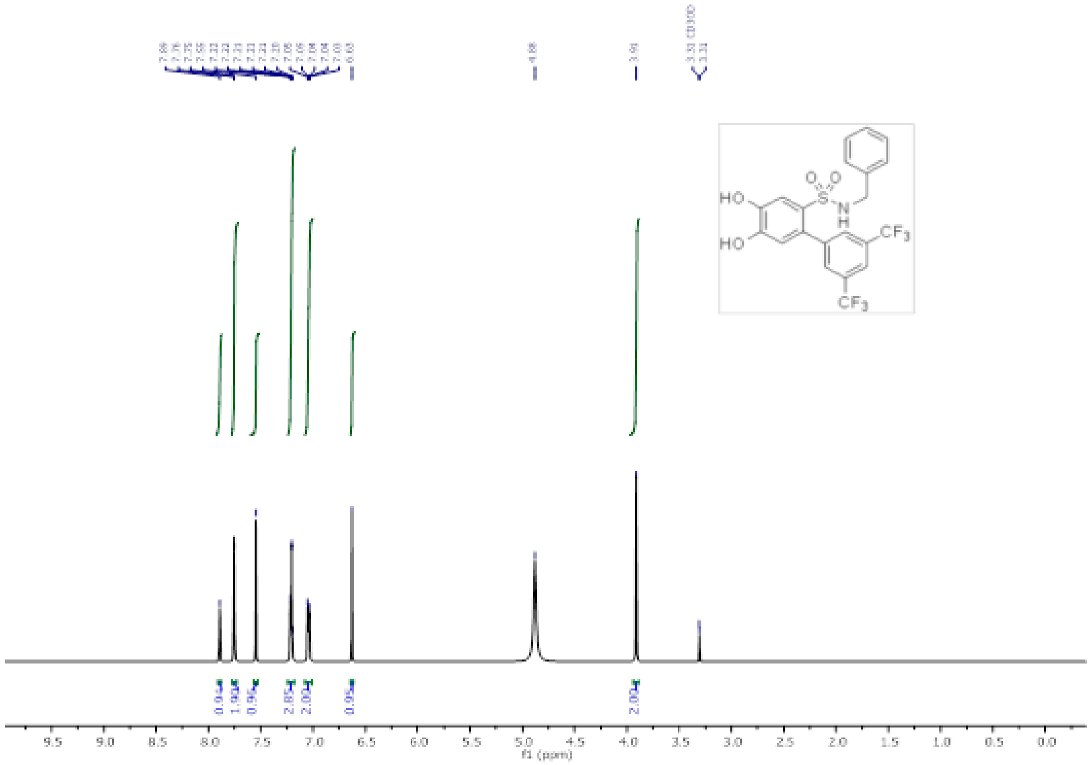

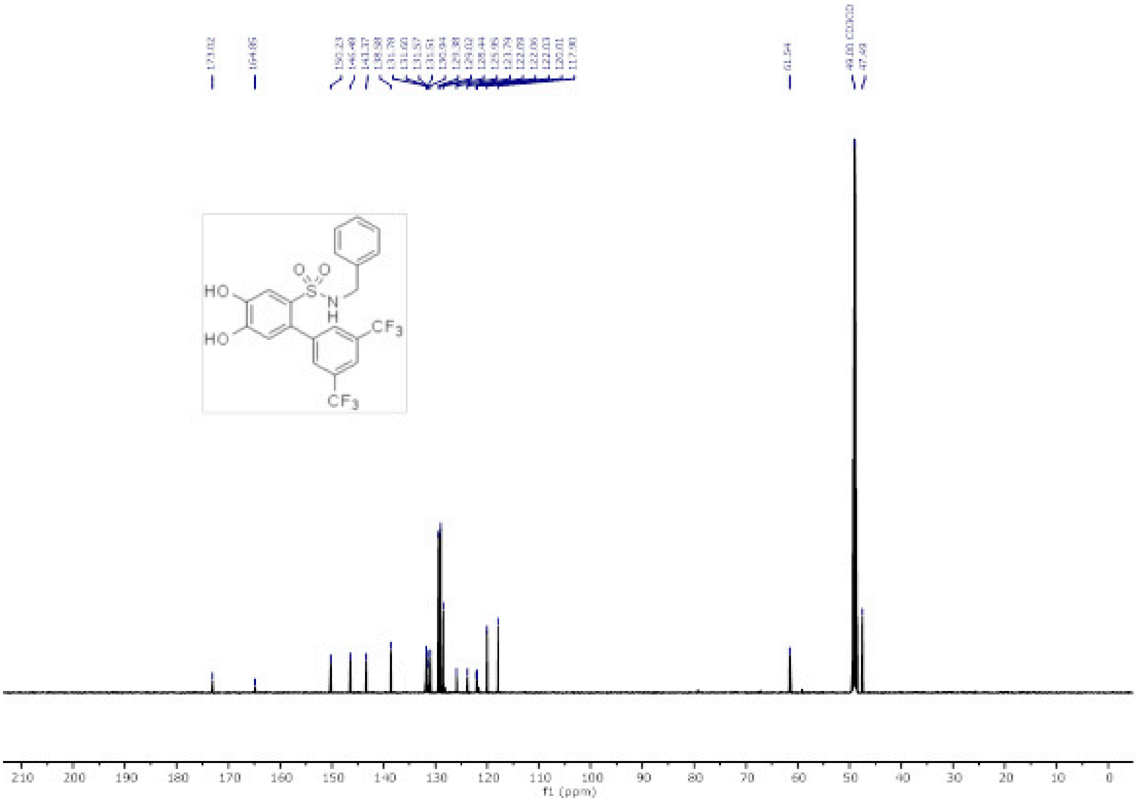

N-benzyl-3'-fluoro-4,4',5-trihydroxy-[1,1'-biphenyl]-2-sulfonamide ***(9g)****:*

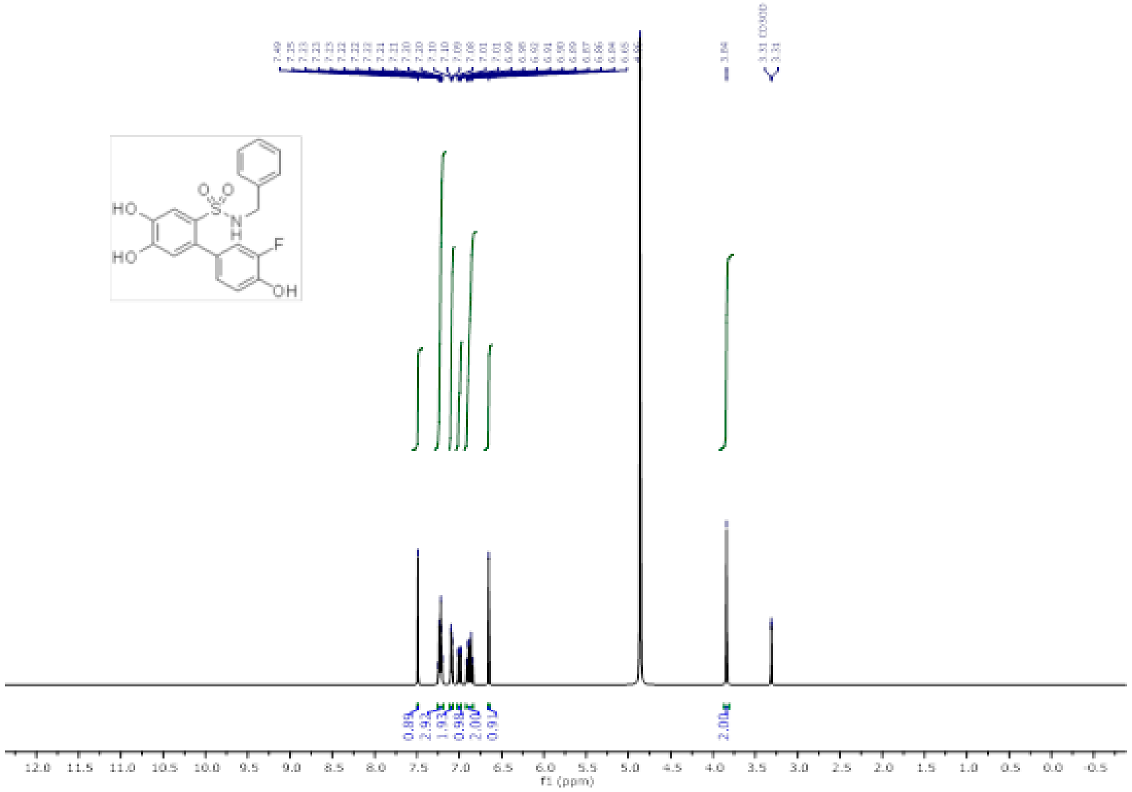

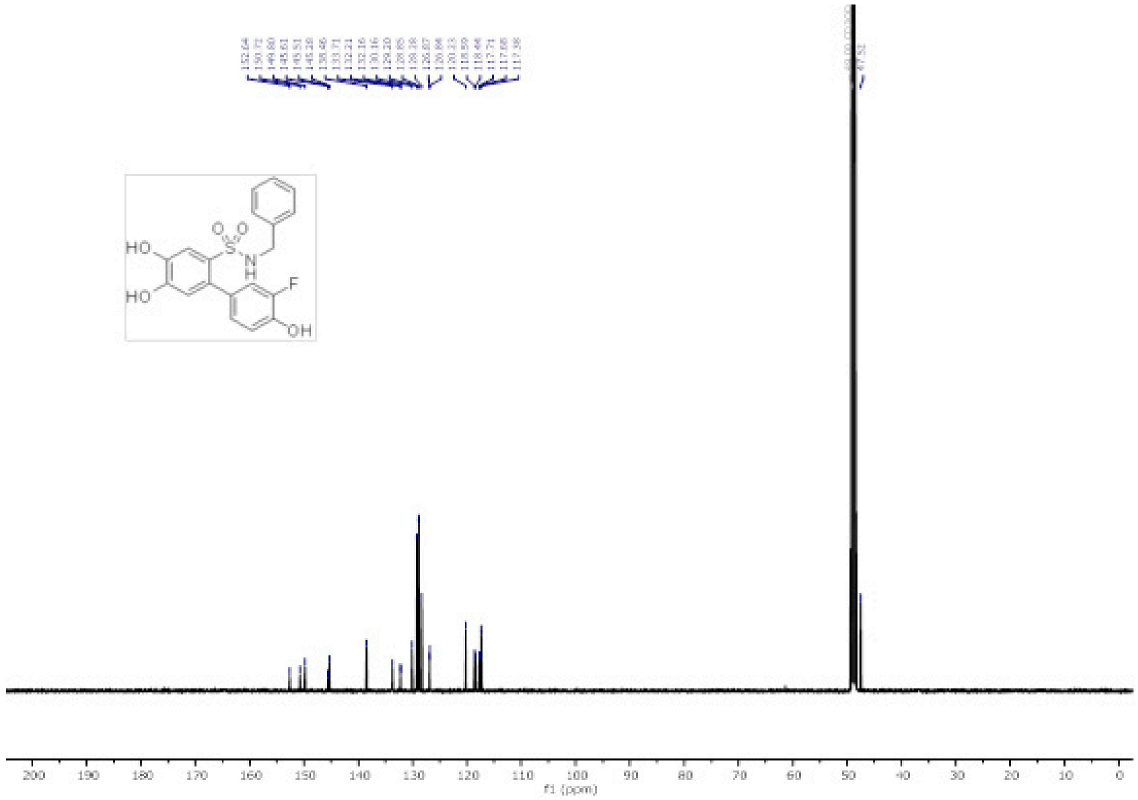

N-benzyl-4,4',5-trihydroxy-3'-(trifluoromethyl)-[1,1'-biphenyl]-2-sulfonamide ***(9h)****:*

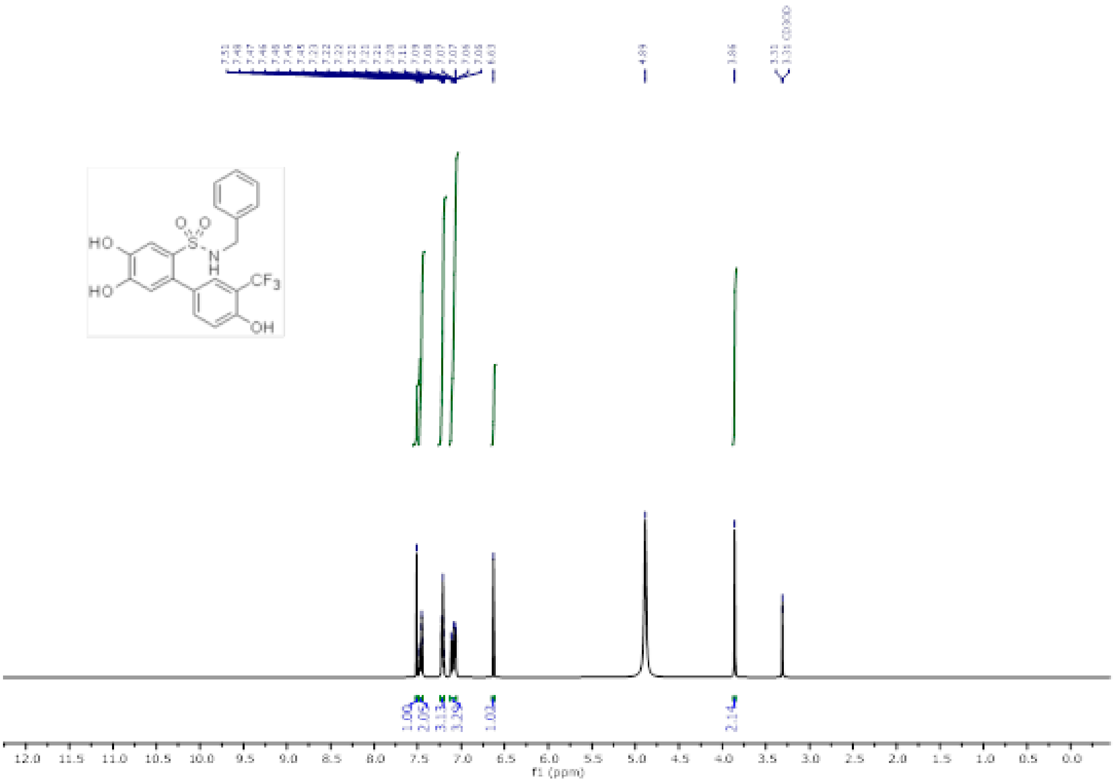

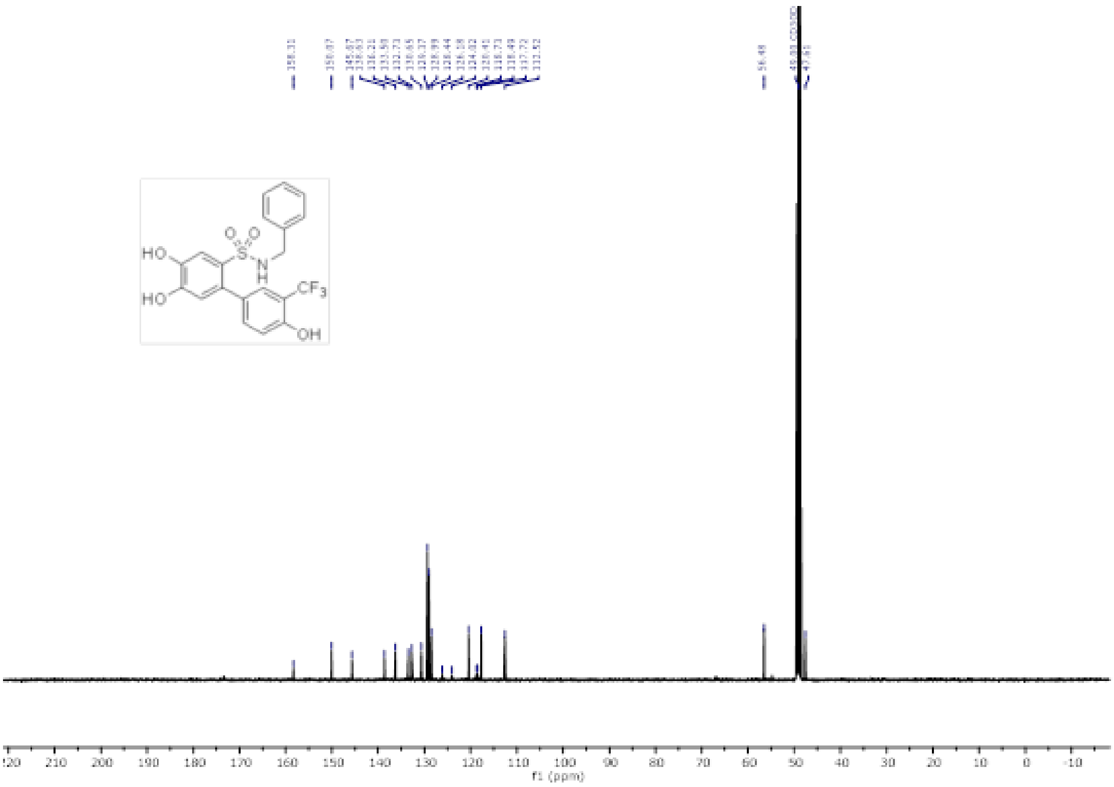

*N-benzyl-4,5-dihydroxy-4'-(trifluoromethoxy)-[1,1'-biphenyl]-2-sulfonamide **(9i)**:*

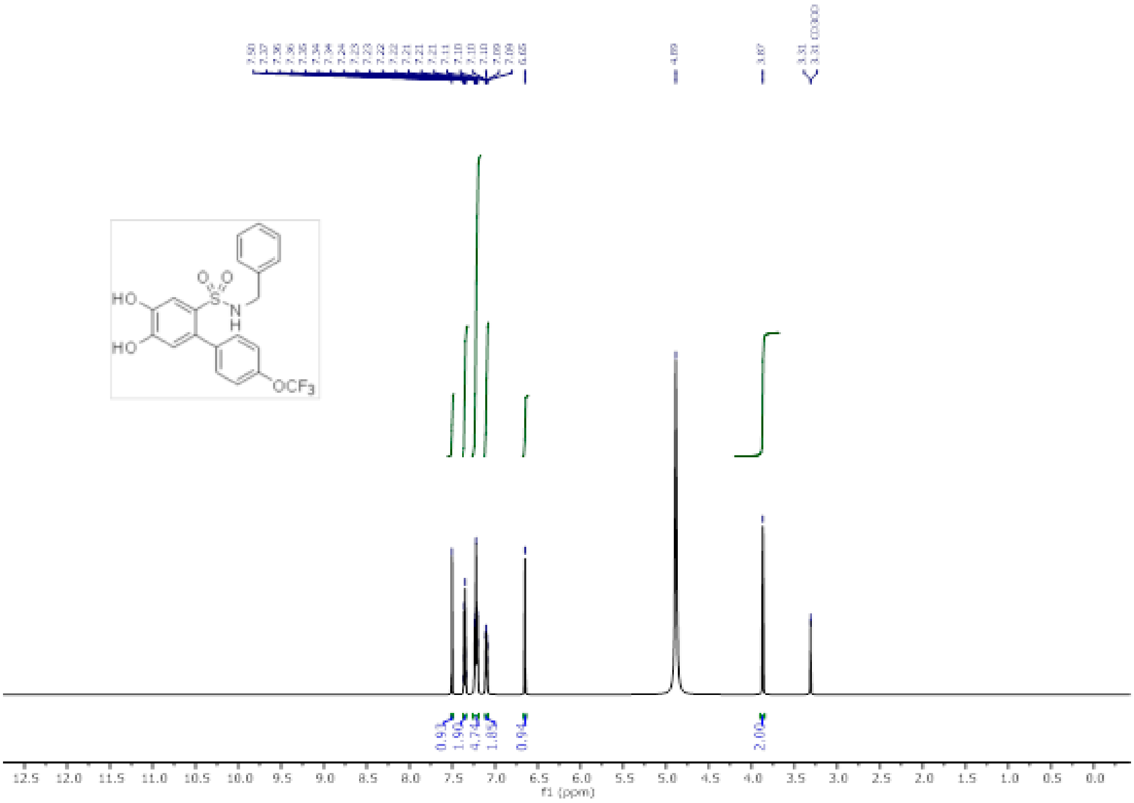

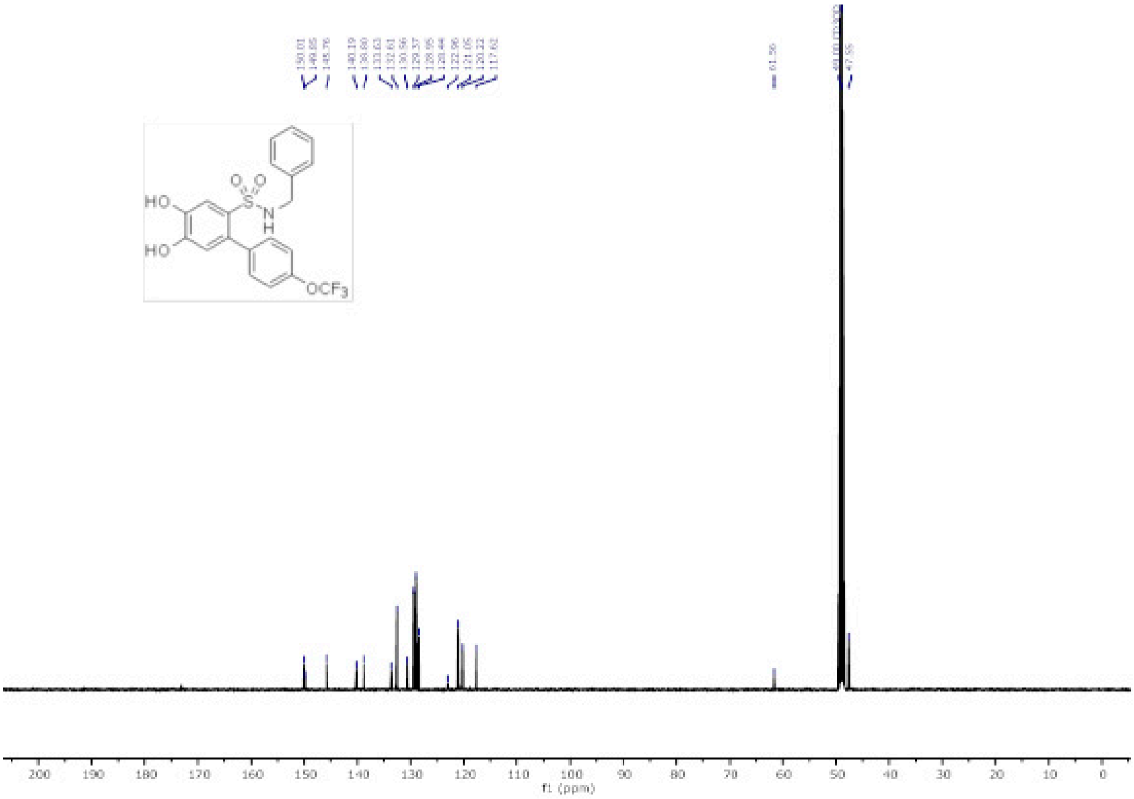

N-benzyl-2-(6-chloro-2-methoxypyridin-3-yl)-4,5-dihydroxybenzenesulfonamide ***(9j)***:

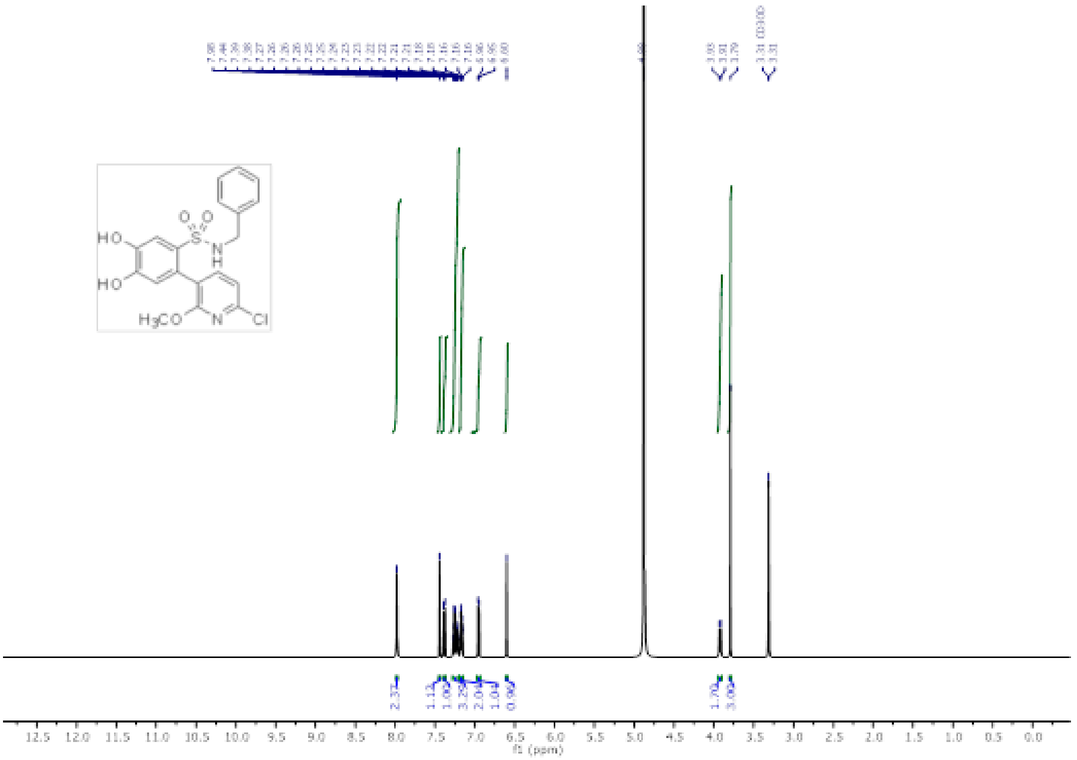

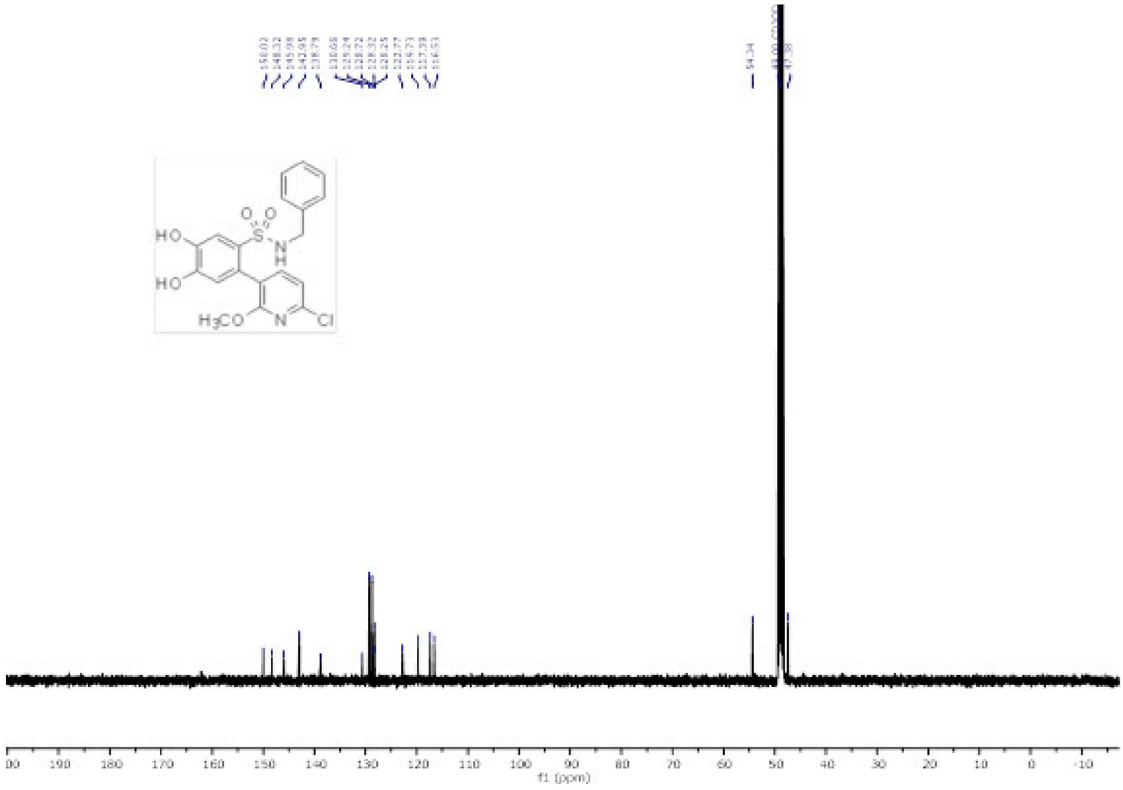

2-bromo-N-(4-fluorobenzyl)-4,5-dimethoxybenzenesulfonamide ***(10)***:

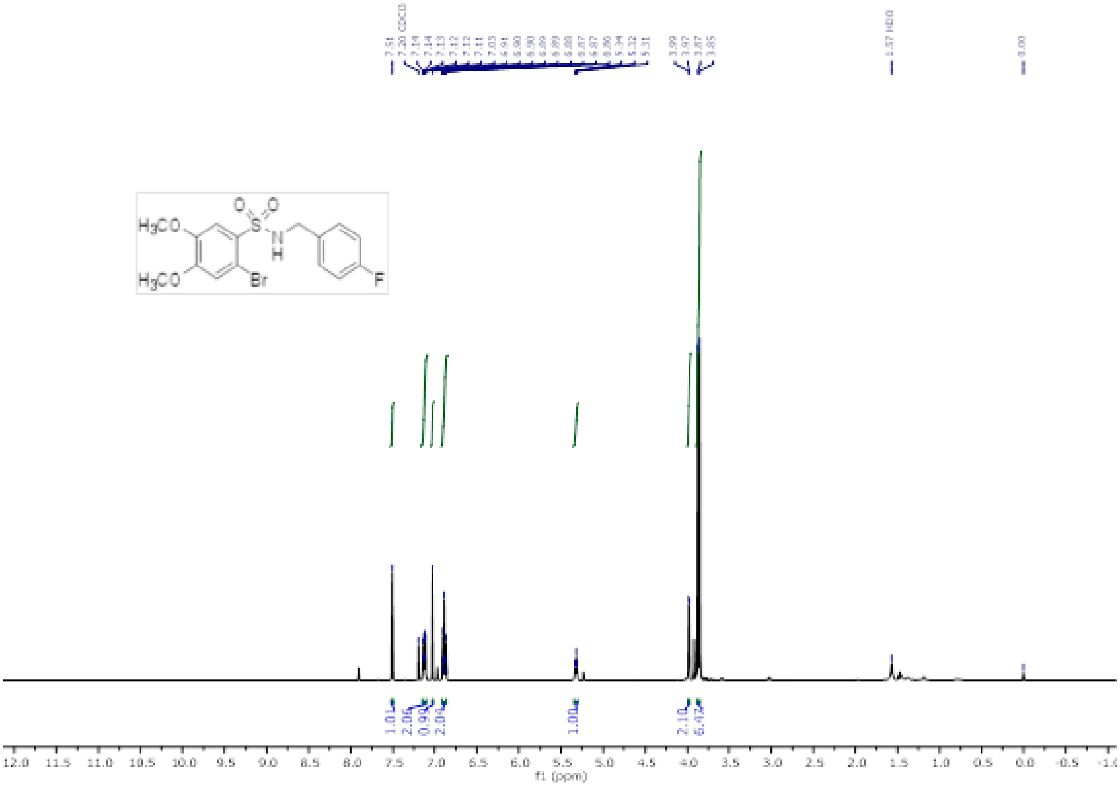

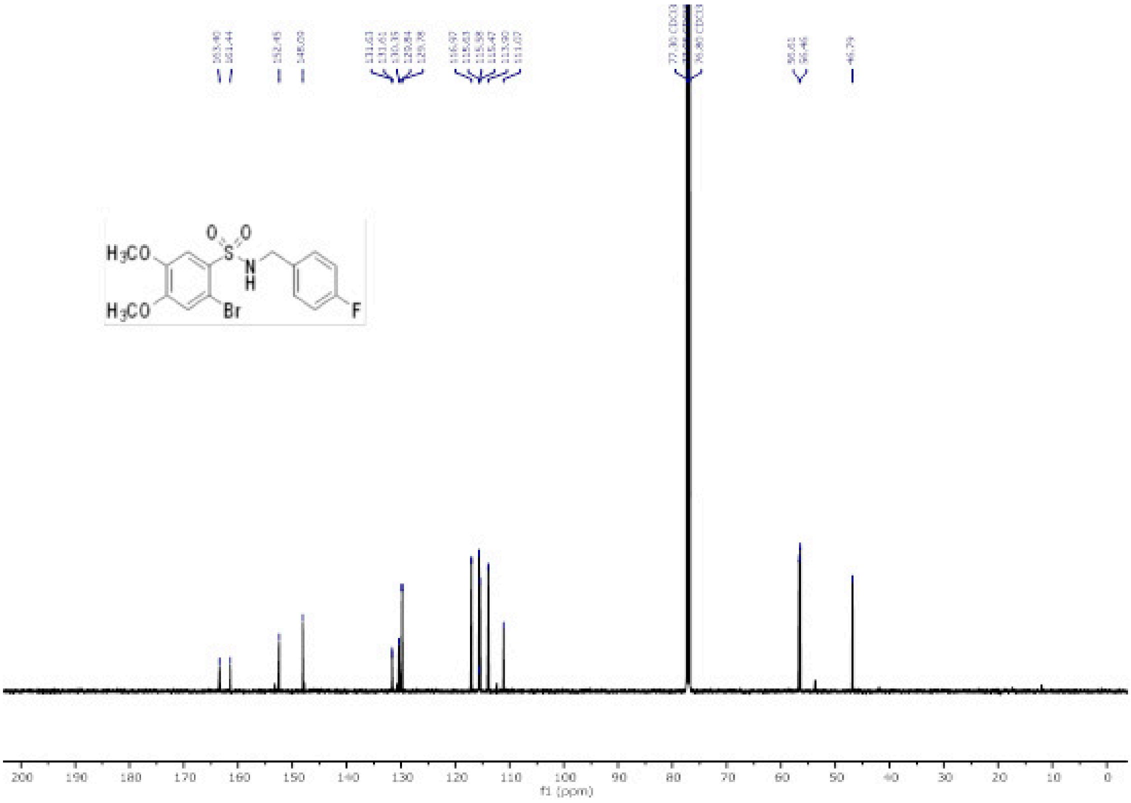

N-(4-fluorobenzyl)-3',4,5-trimethoxy-[1,1'-biphenyl]-2-sulfonamide ***(11a)***:

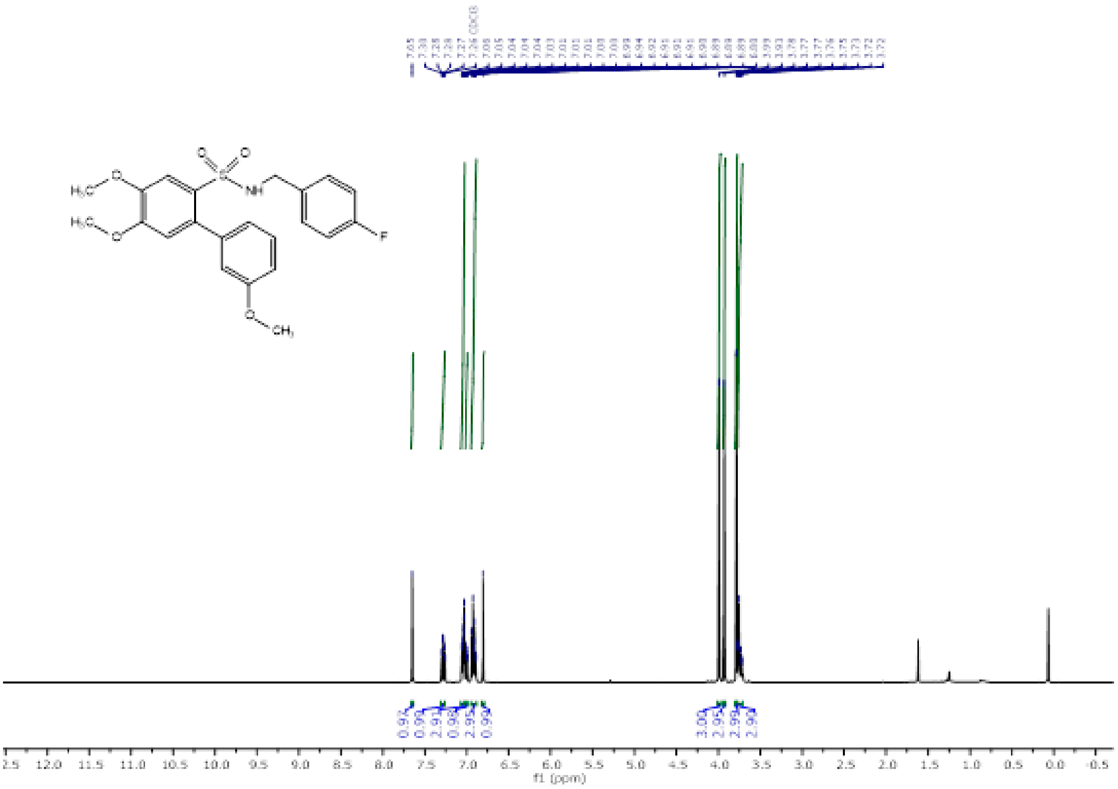

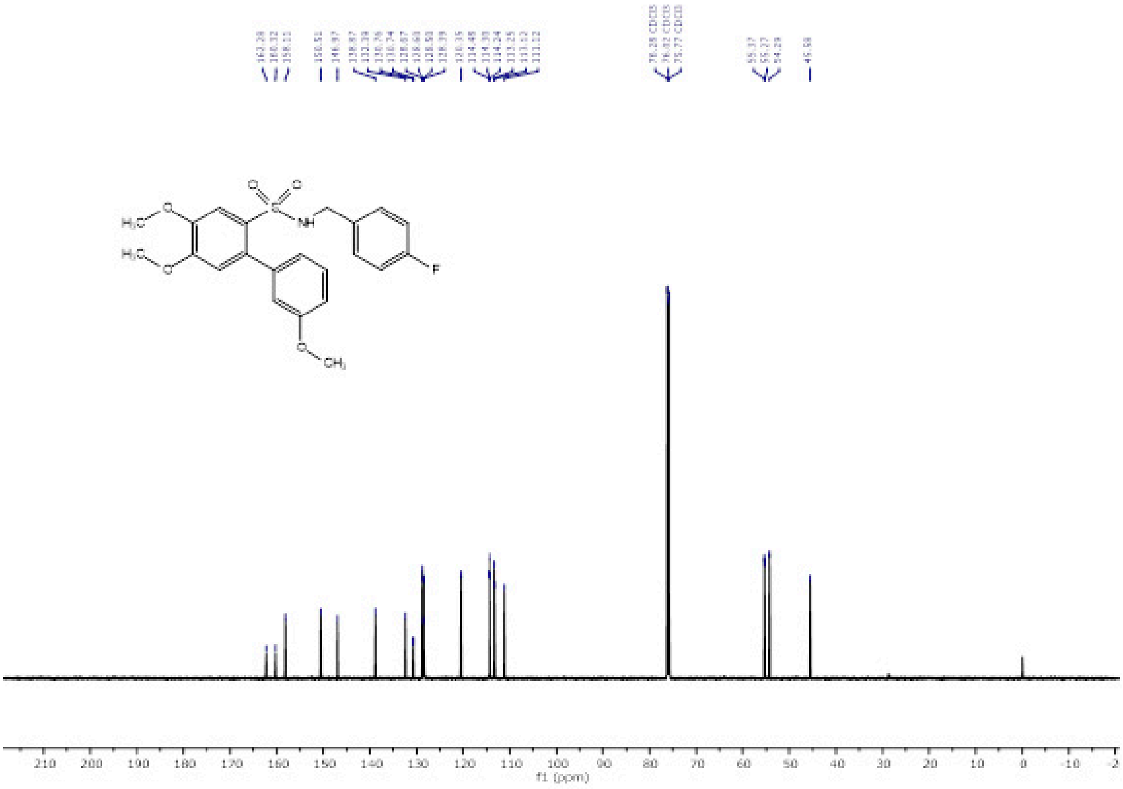

N-(4-fluorobenzyl)-4,4',5-trimethoxy-[1,1'-biphenyl]-2-sulfonamide ***(11b)***:

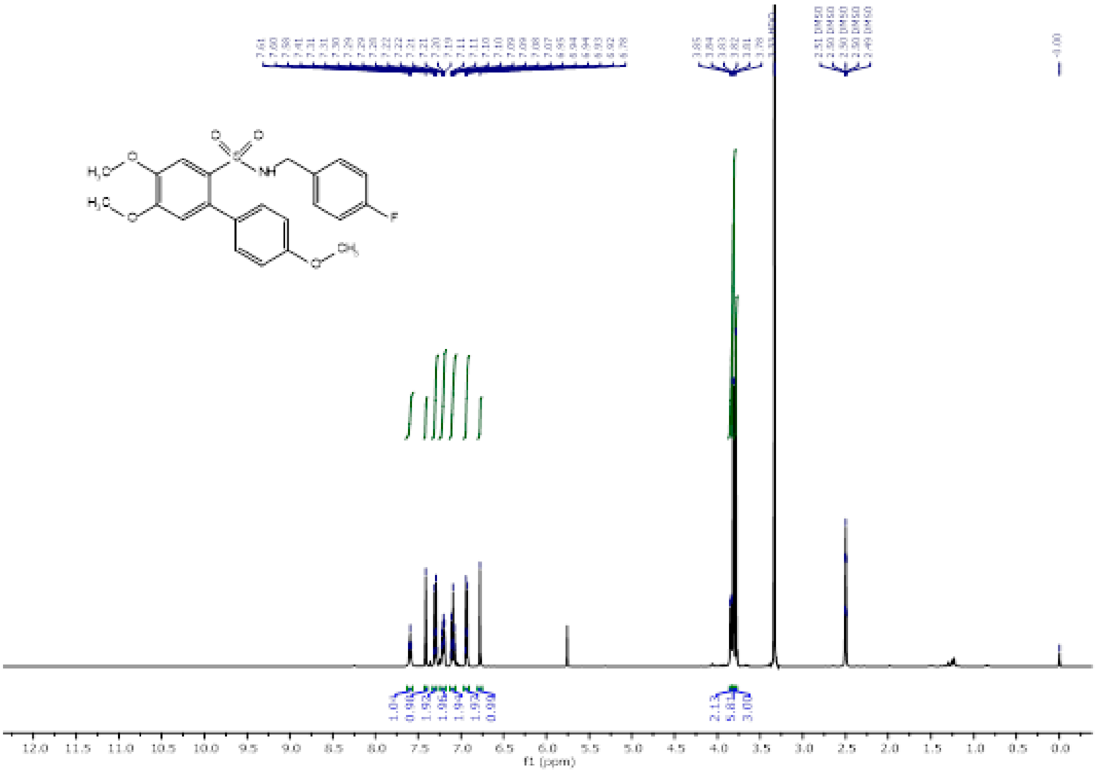

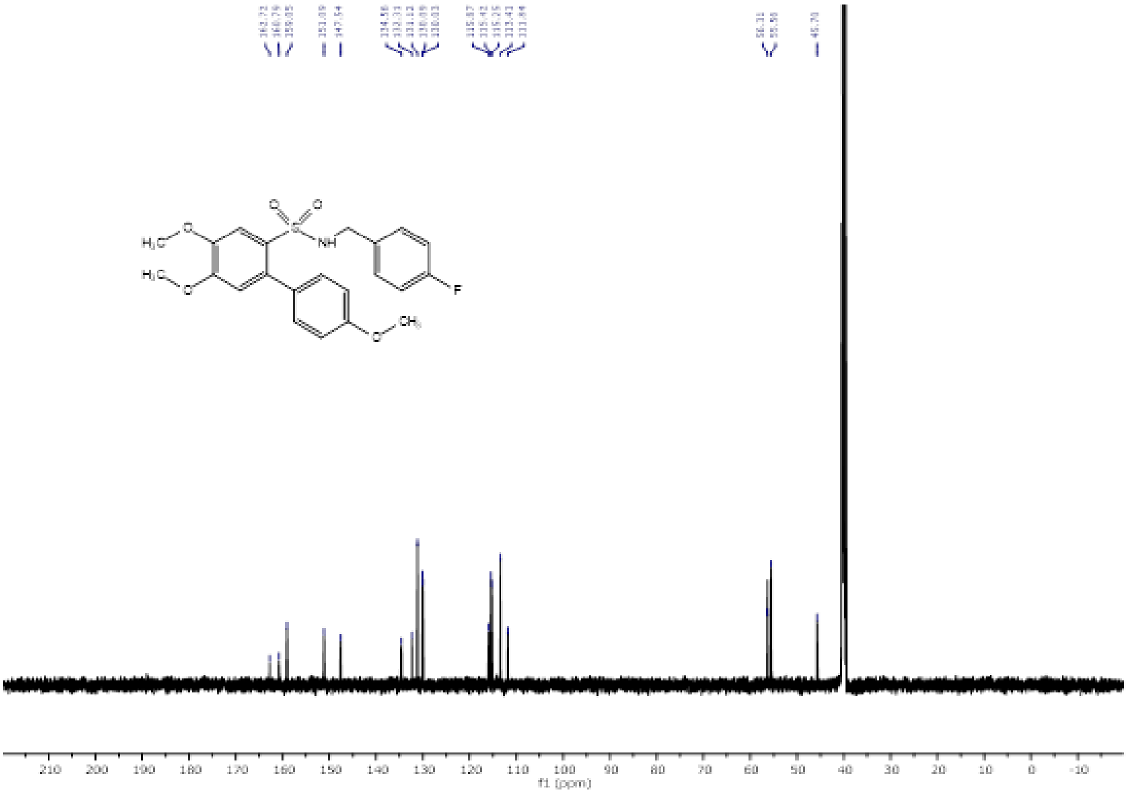

N-(4-fluorobenzyl)-3',4,5,5'-tetramethoxy-[1,1'-biphenyl]-2-sulfonamide ***(11c)***:

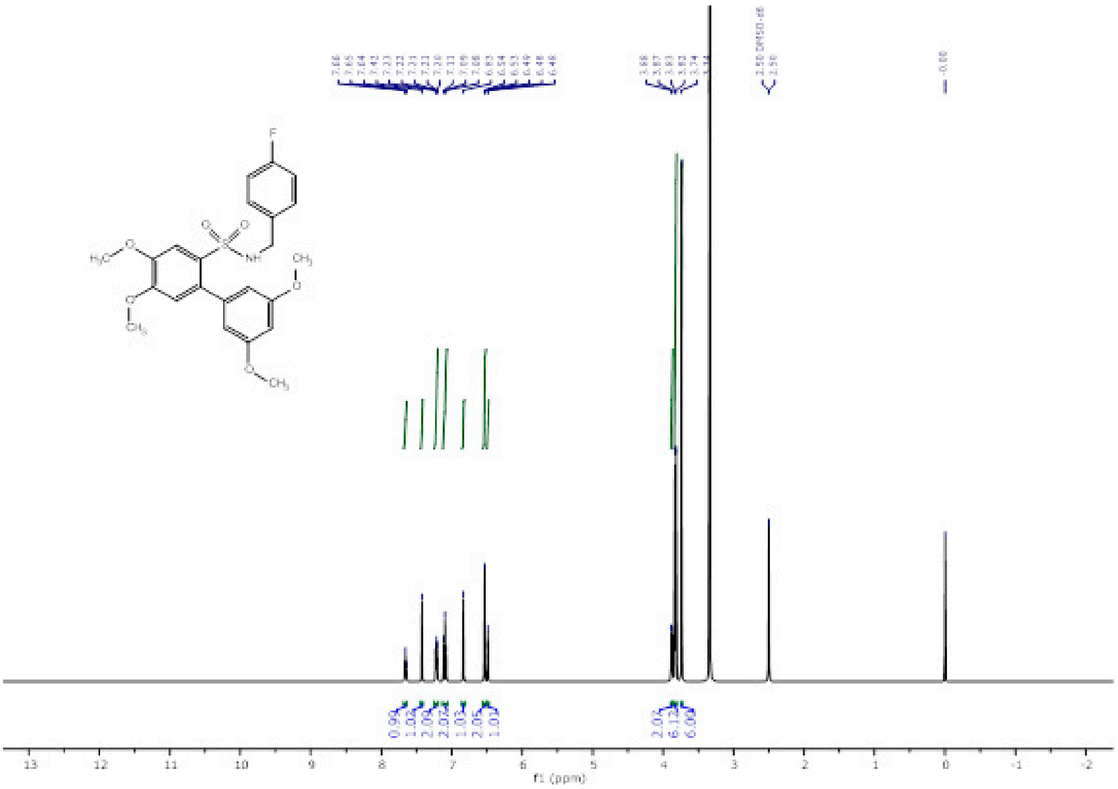

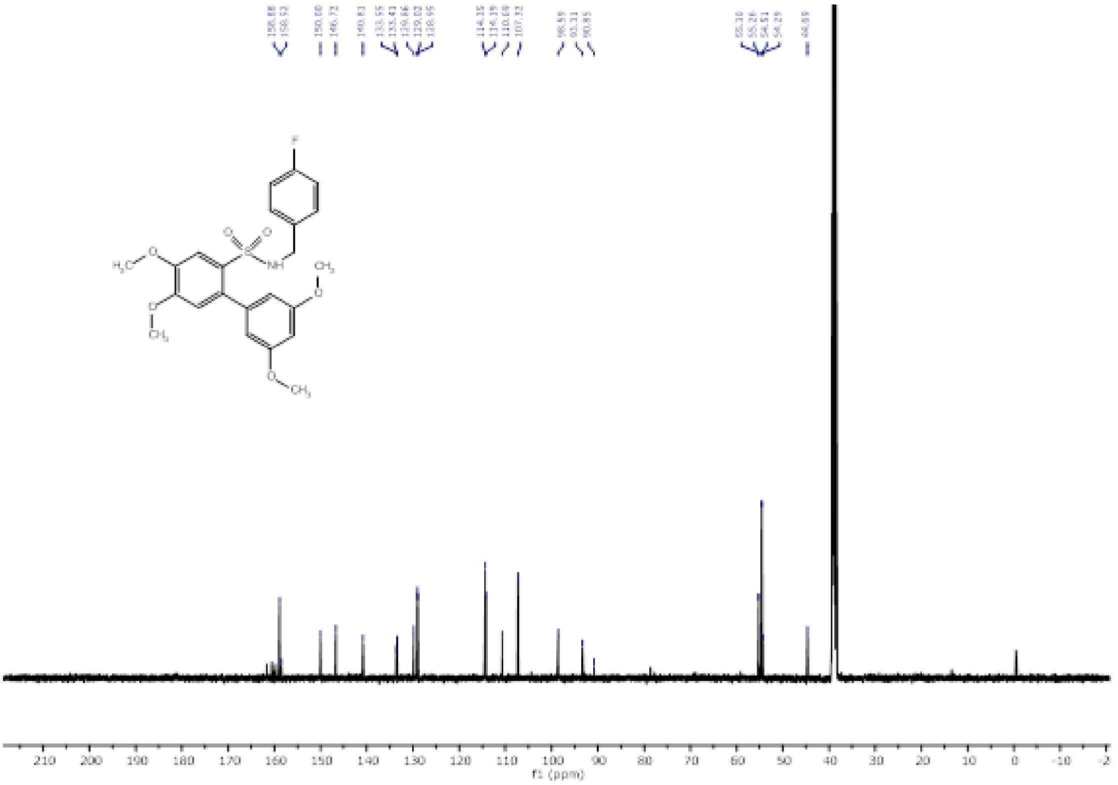

N-(4-fluorobenzyl)-4,5-dimethoxy-4'-(trifluoromethyl)-[1,1'-biphenyl]-2-sulfonamide ***(11e)***:

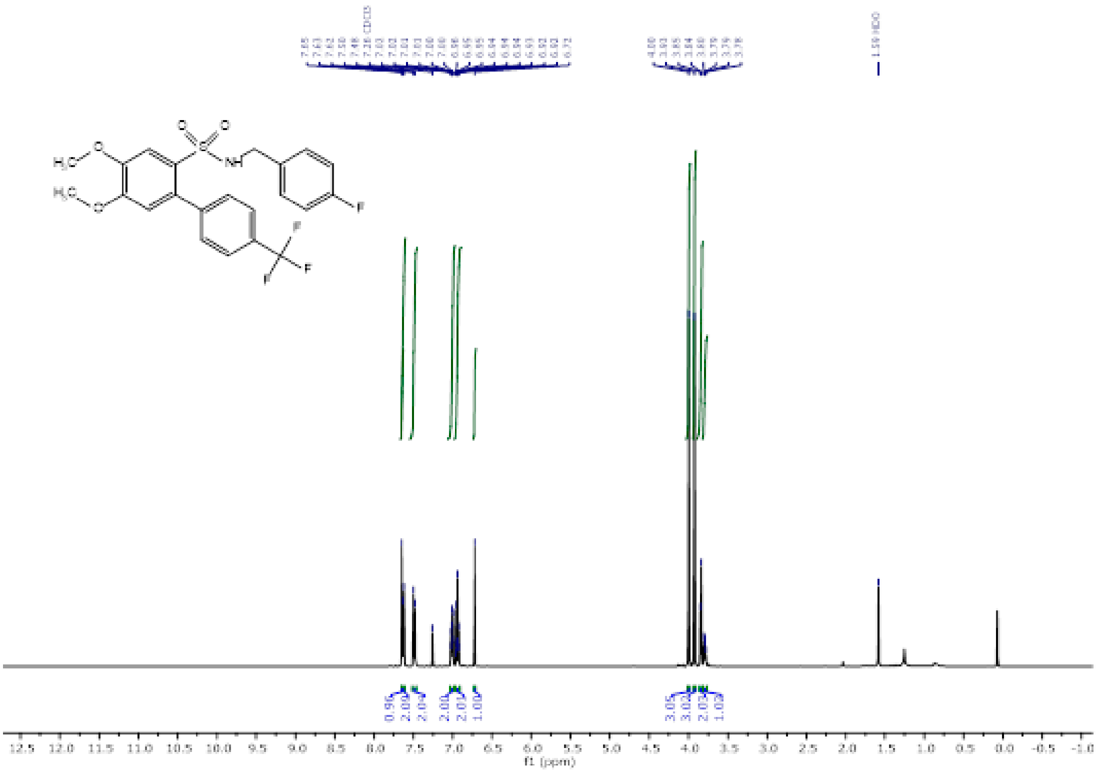

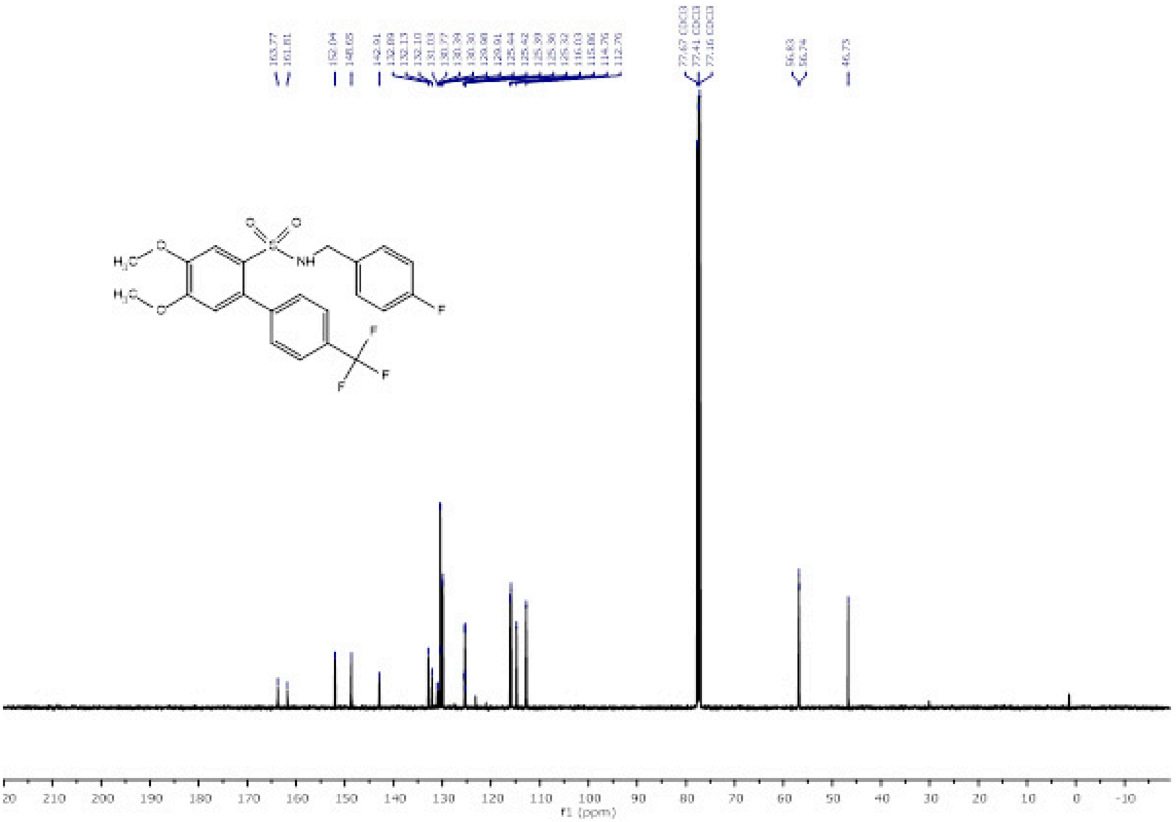

N-(4-fluorobenzyl)-4,5-dimethoxy-3',5'-bis(trifluoromethyl)-[1,1'-biphenyl]-2-sulfon-amide ***(11f)****:*

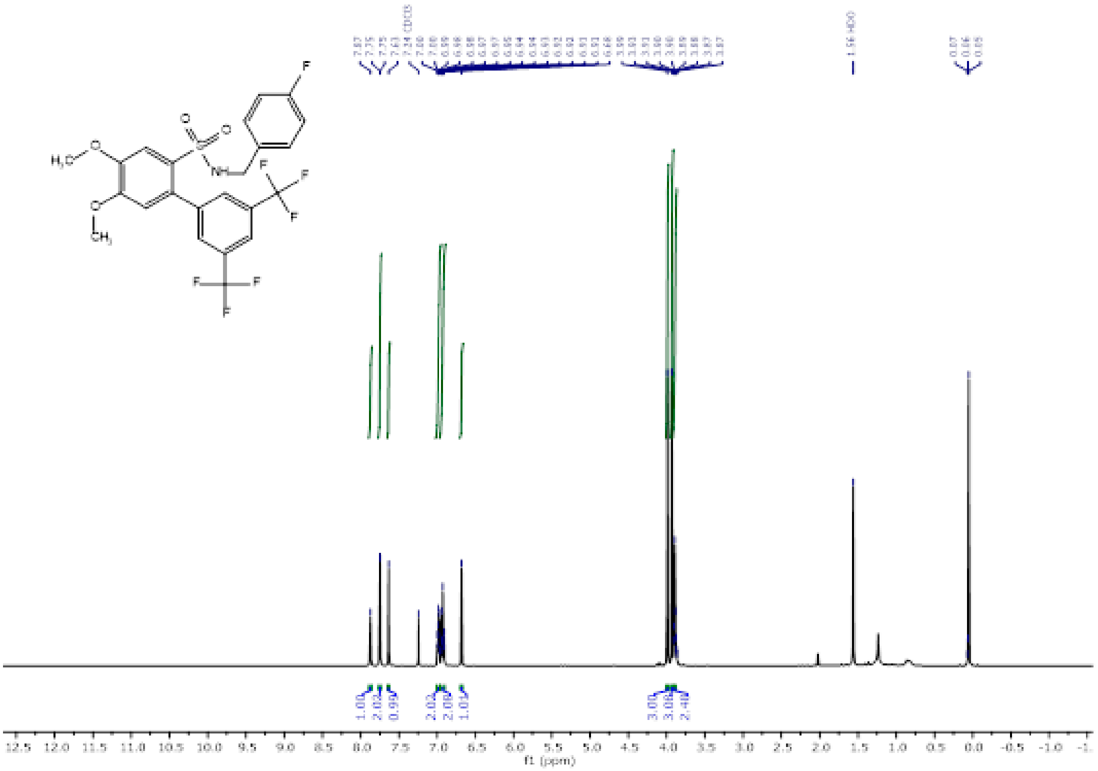

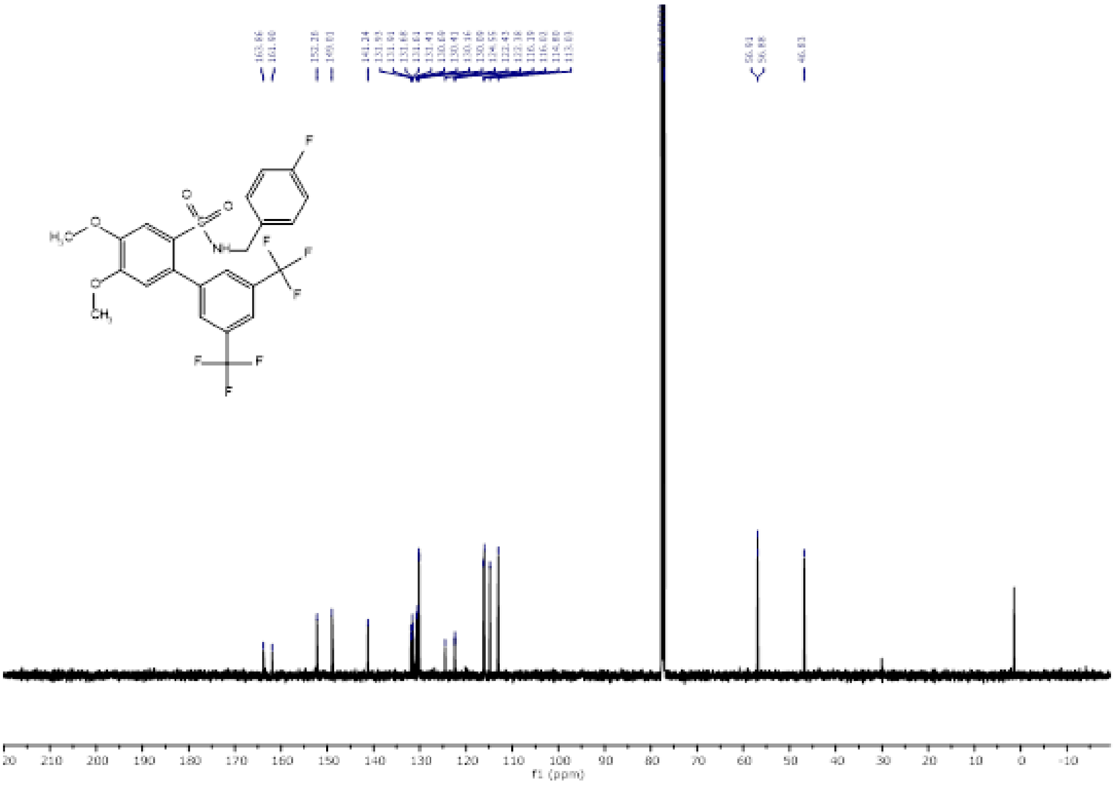

N-(4-fluorobenzyl)-3',4,5-trihydroxy-[1,1'-biphenyl]-2-sulfonamide ***(12a)****:*

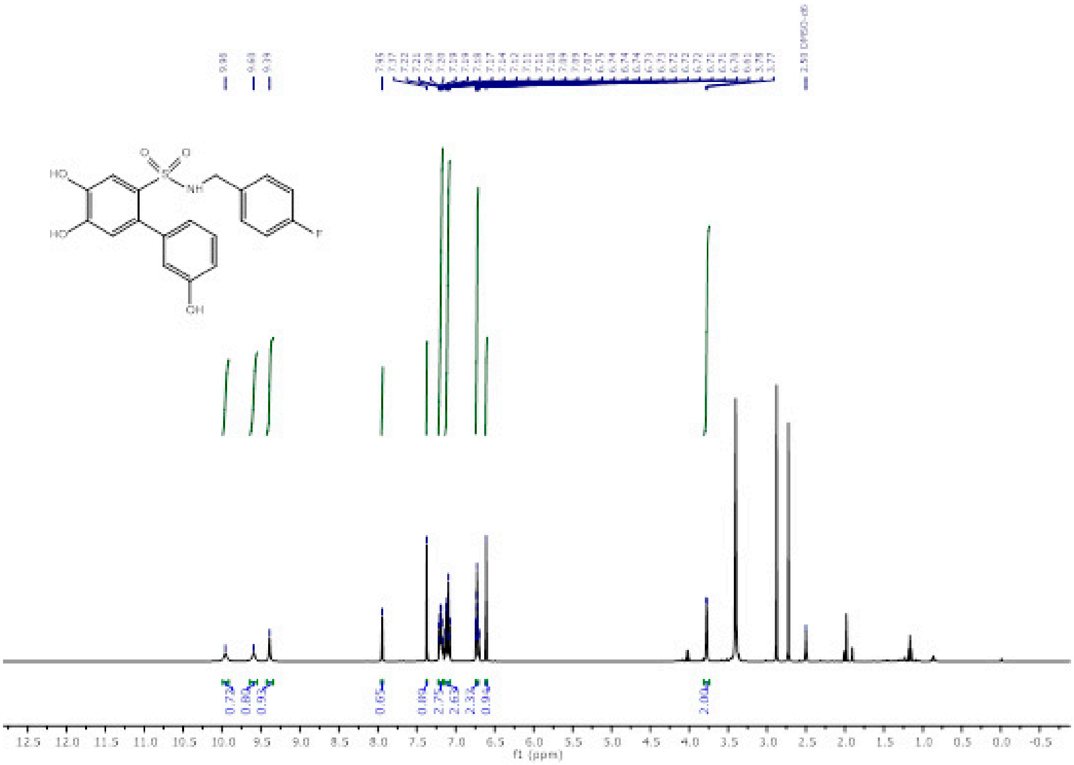

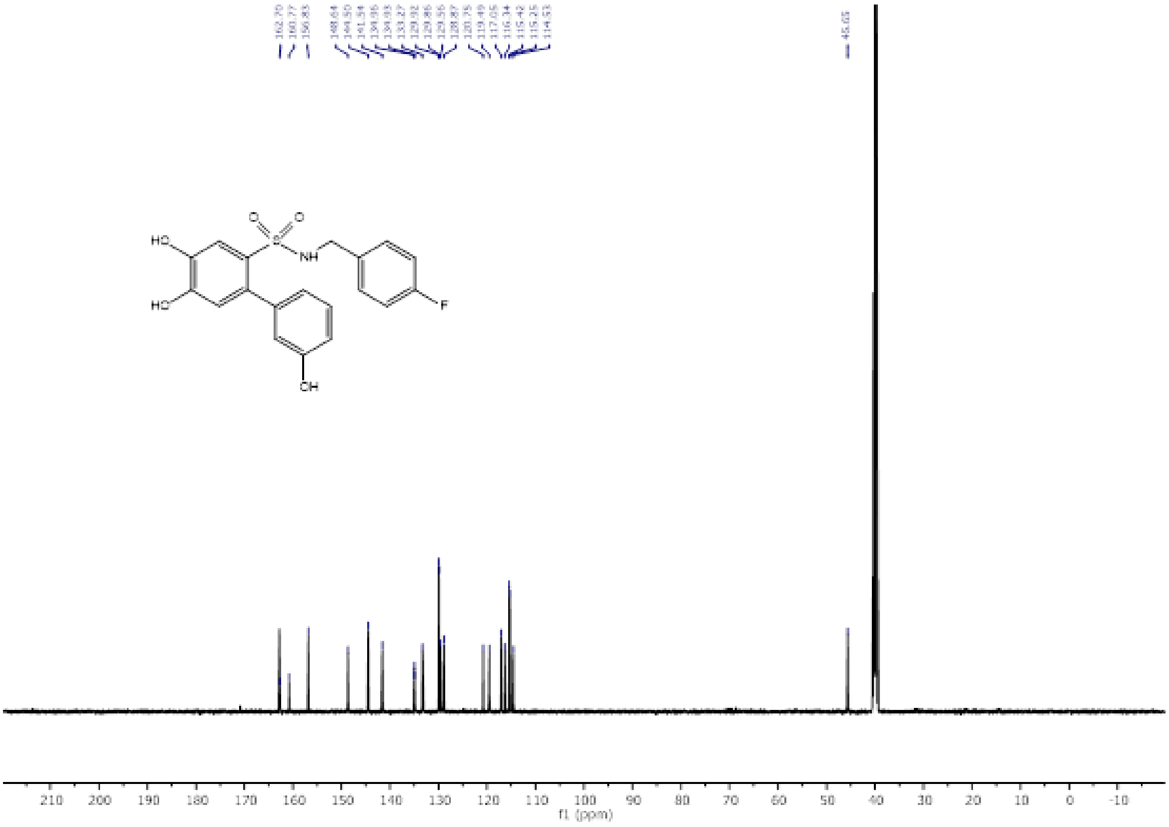

N-(4-fluorobenzyl)-4,4',5-trihydroxy-[1,1'-biphenyl]-2-sulfonamide ***(12b)***:

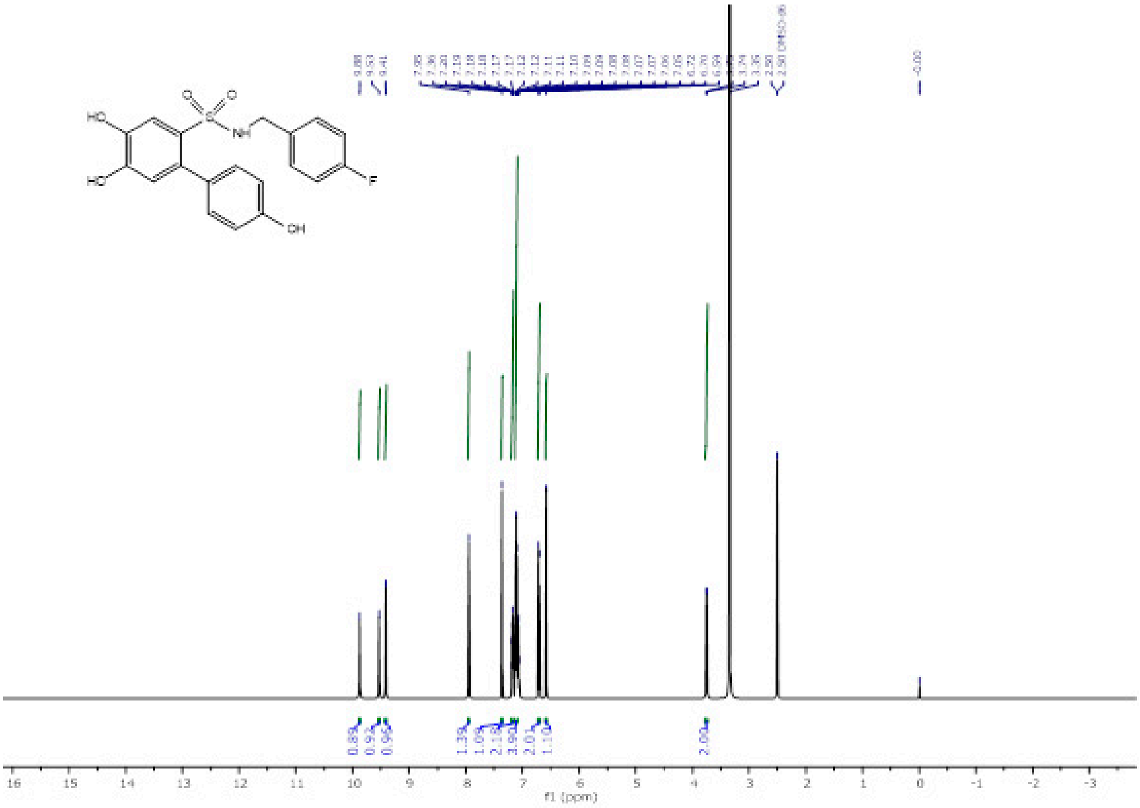

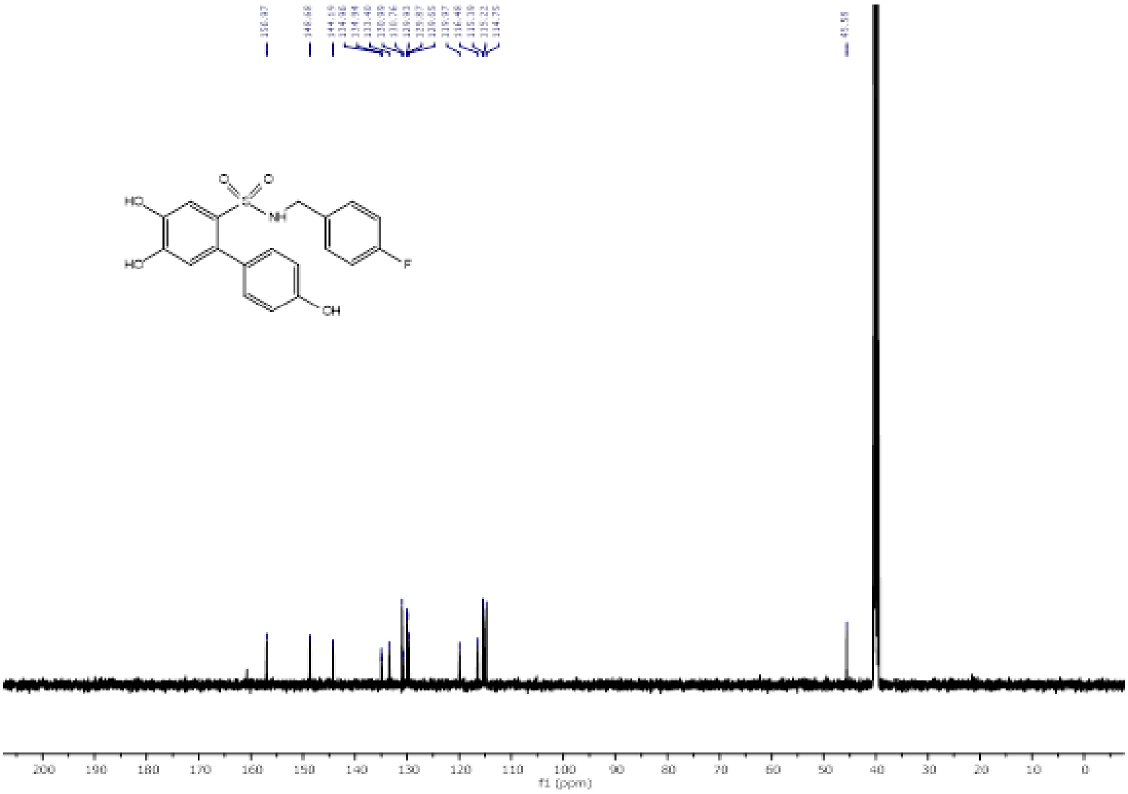

N-(4-fluorobenzyl)-3',4,5,5'-tetrahydroxy-[1,1'-biphenyl]-2-sulfonamide ***(12c)***:

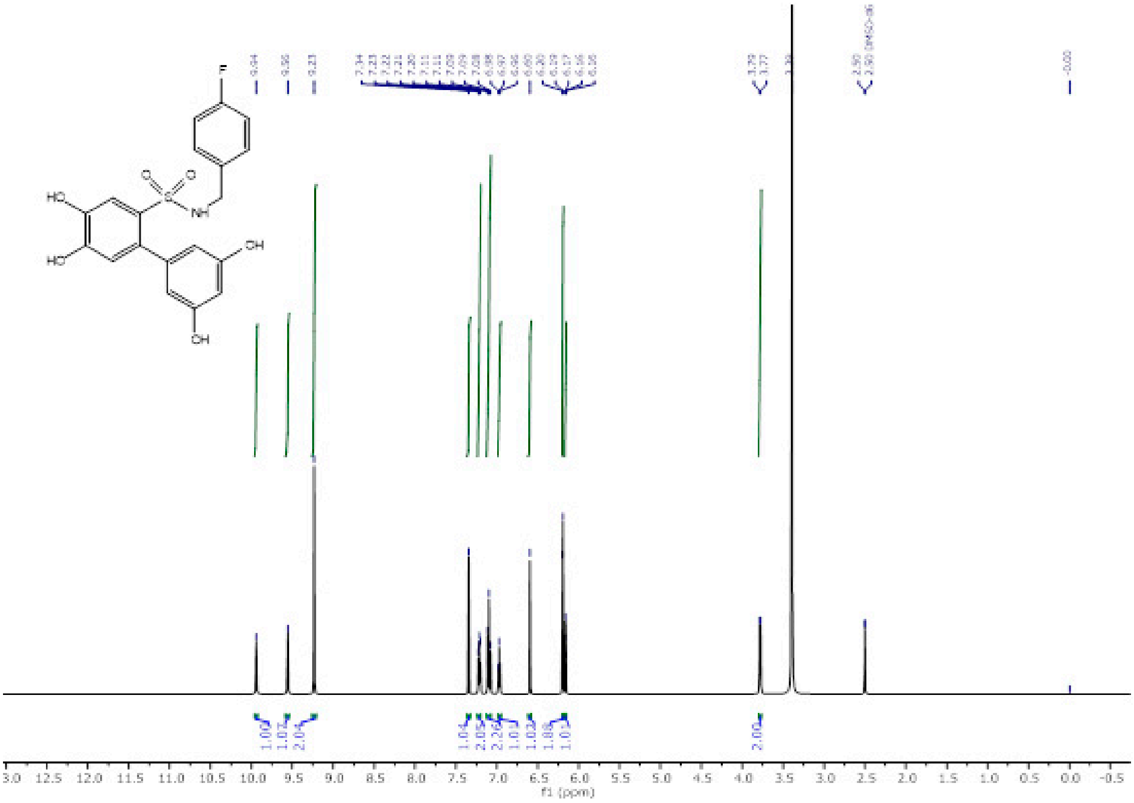

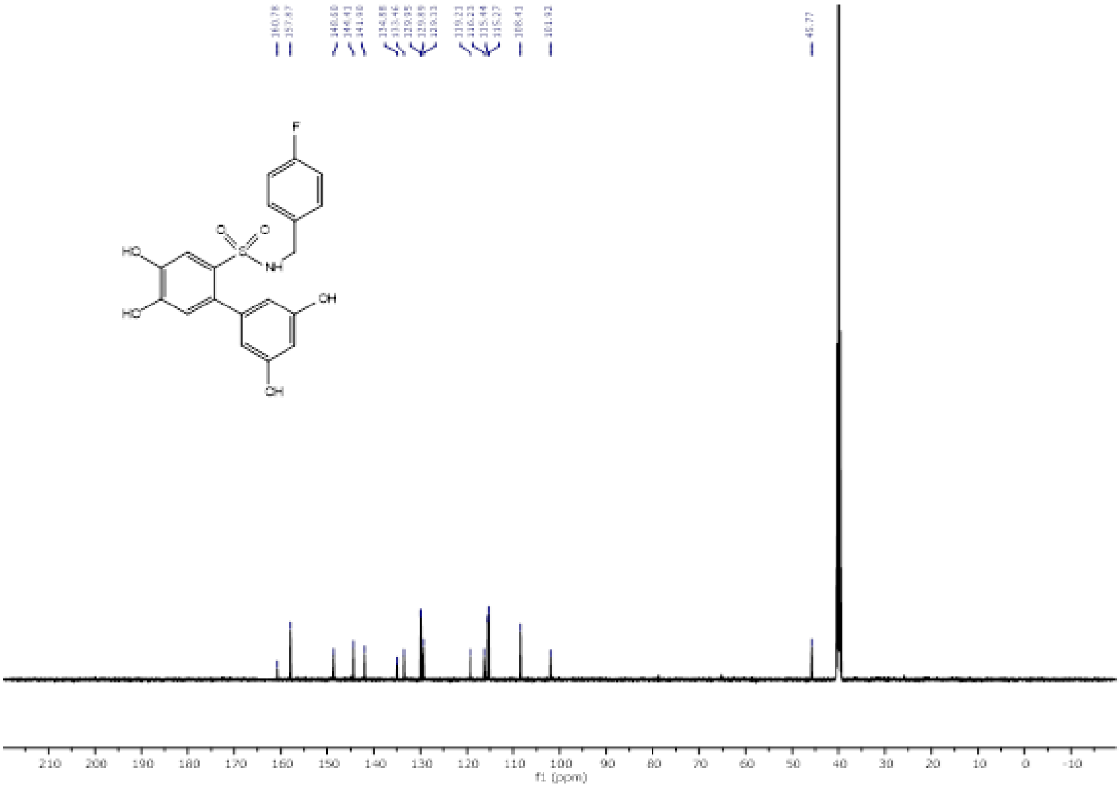

N-(4-fluorobenzyl)-3',4,4',5-tetrahydroxy-[1,1'-biphenyl]-2-sulfonamide ***(12d)***:

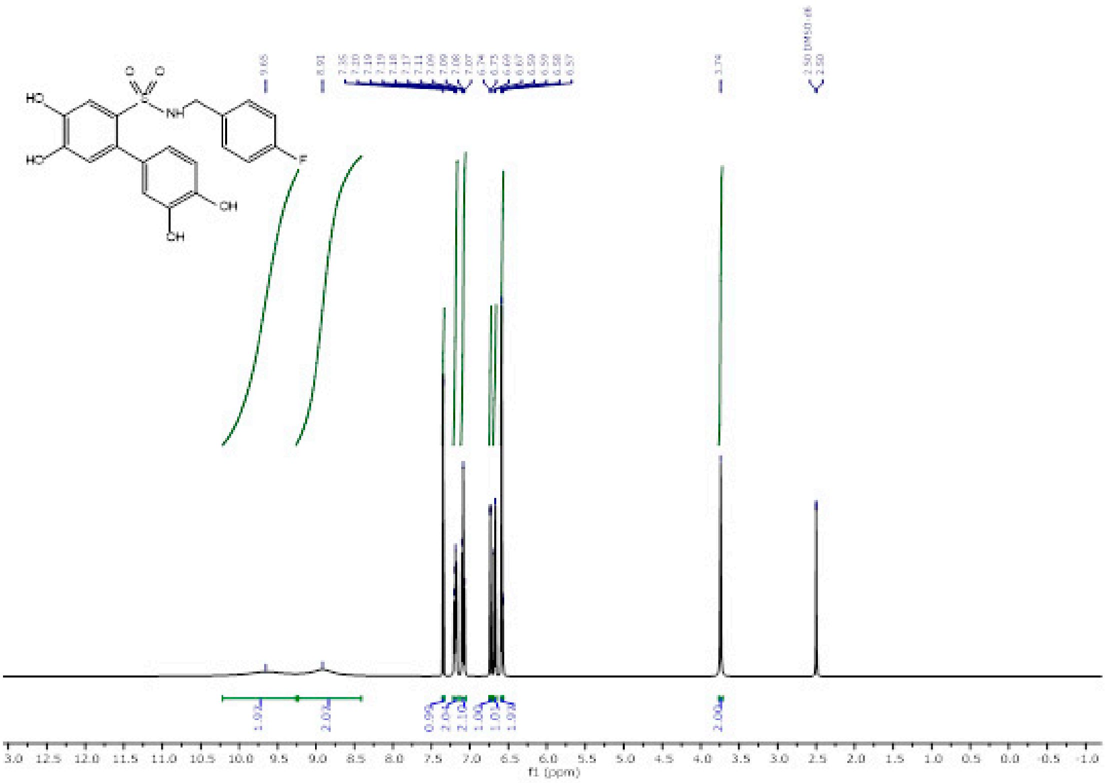

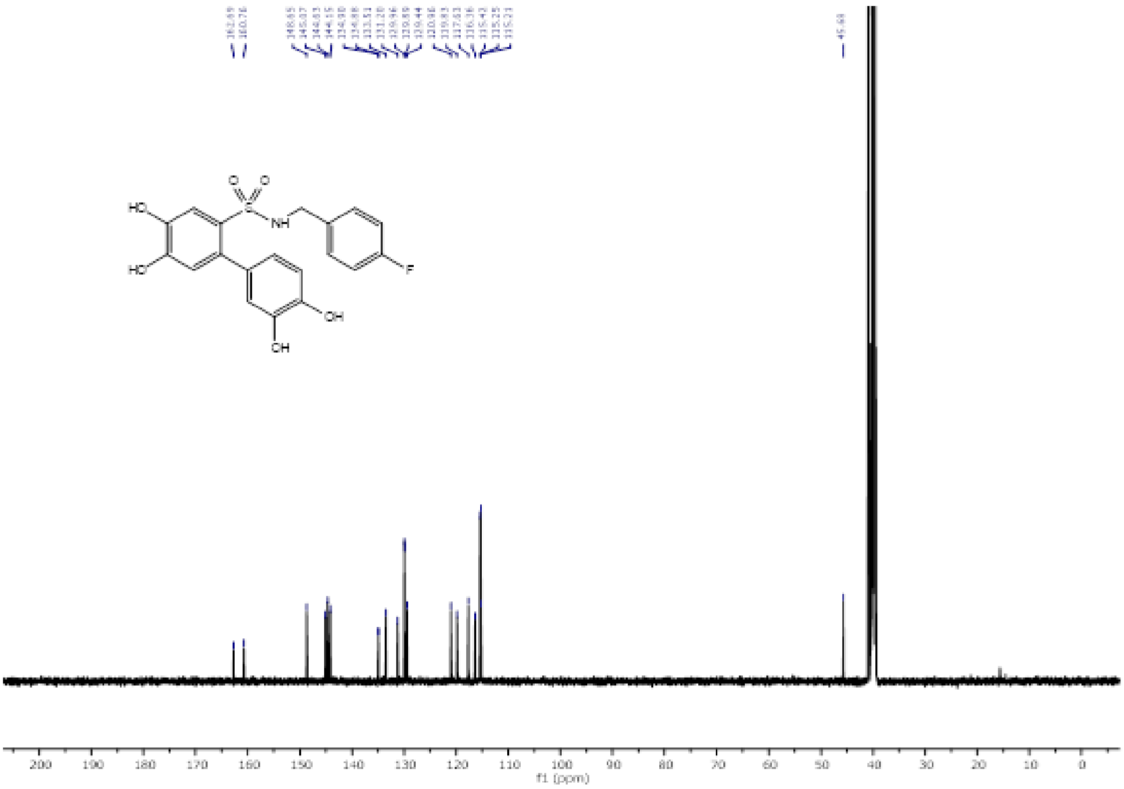

N-(4-fluorobenzyl)-4,5-dihydroxy-4'-(trifluoromethyl)-[1,1'-biphenyl]-2-sulfonamide ***(12e)****:*

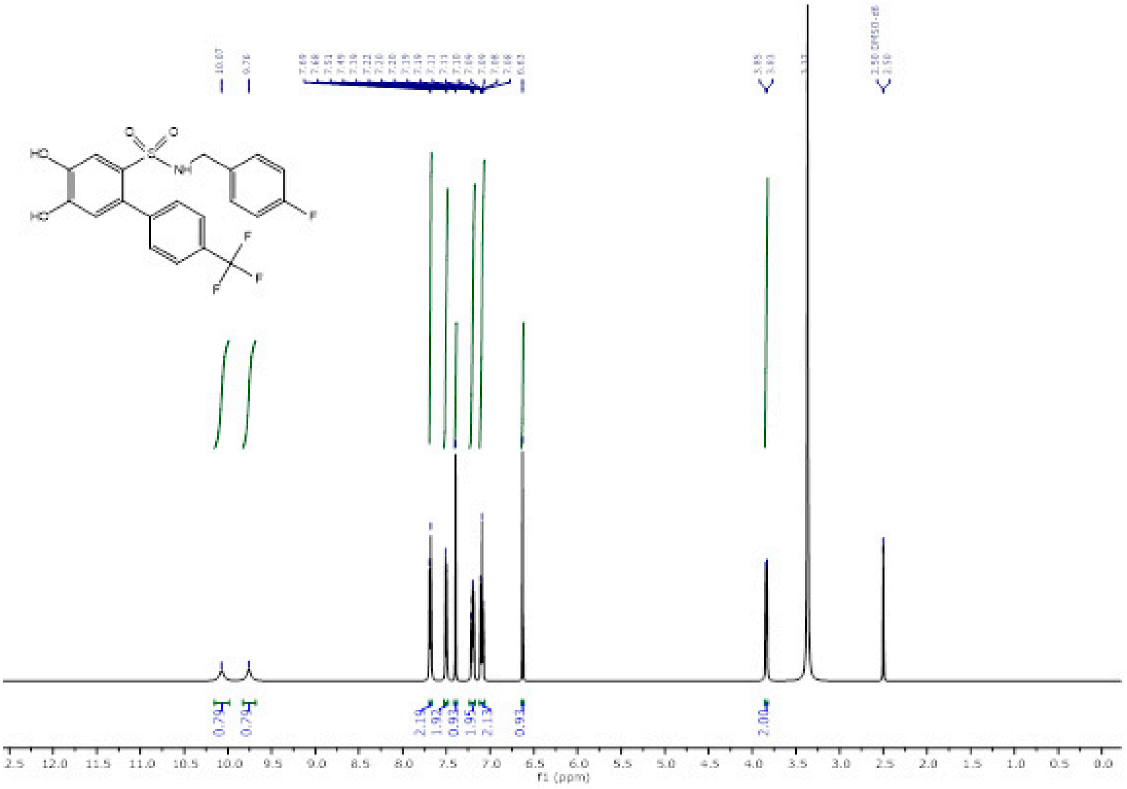

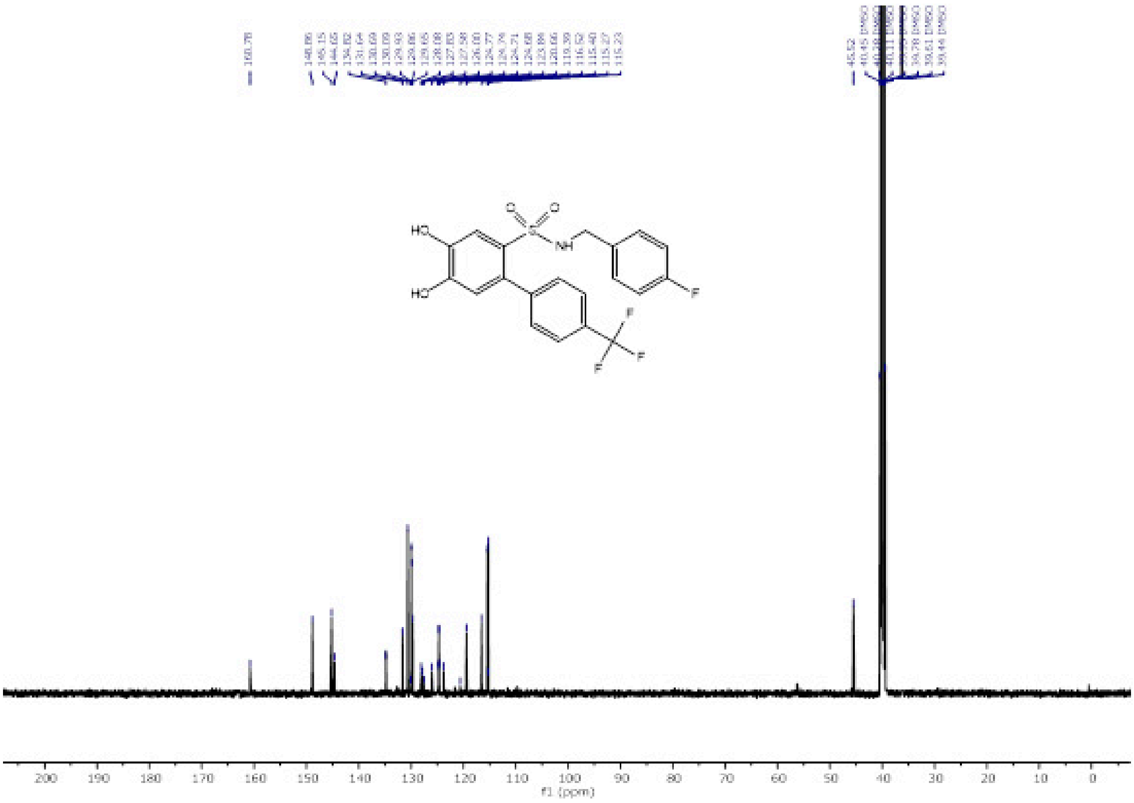

N-(4-fluorobenzyl)-4,5-dihydroxy-3',5'-bis(trifluoromethyl)-[1,1'-biphenyl]-2-sulfon-amide ***(12f)***:

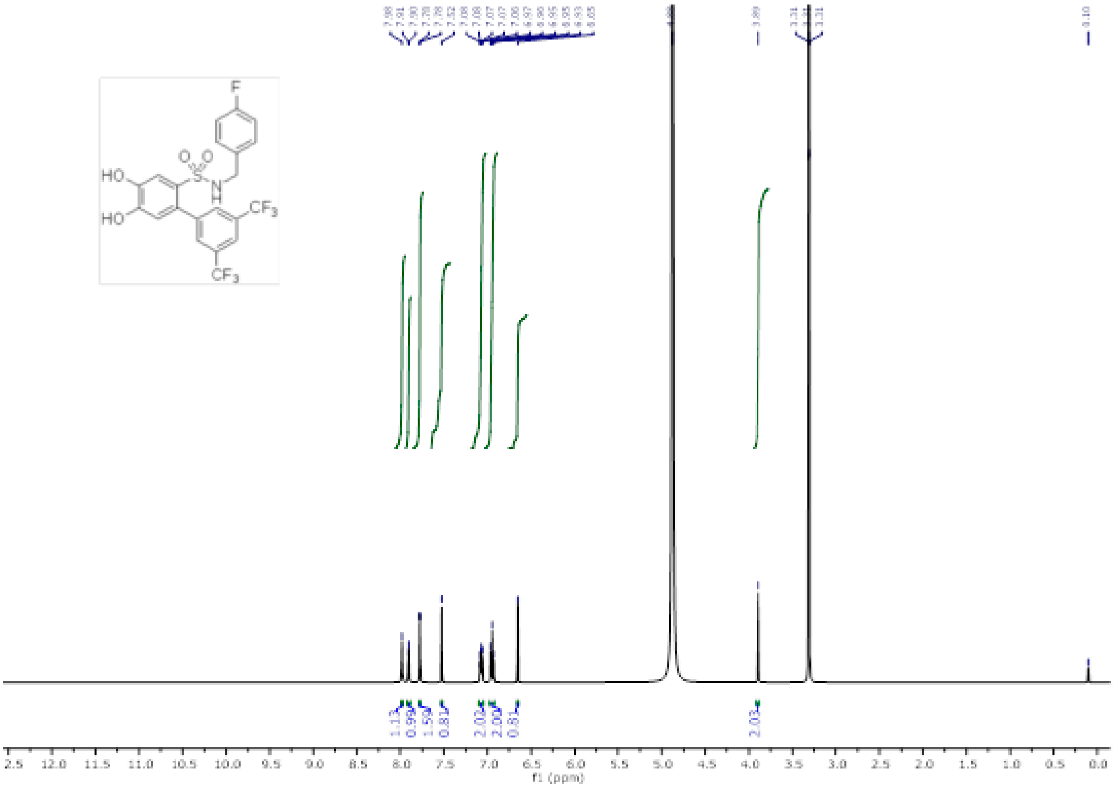

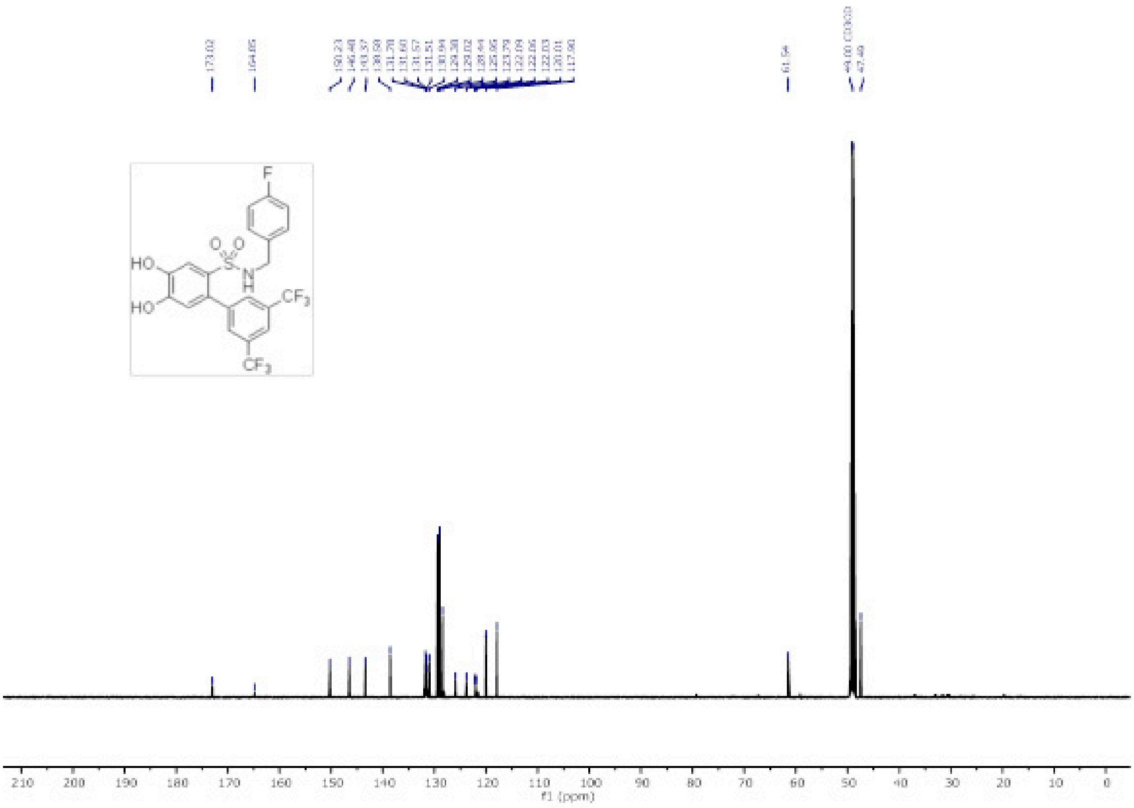

6-benzyl-2,3,9-trimethoxy-6H-dibenzo[c,e][1,2]thiazine 5,5-dioxide ***(14a)***:

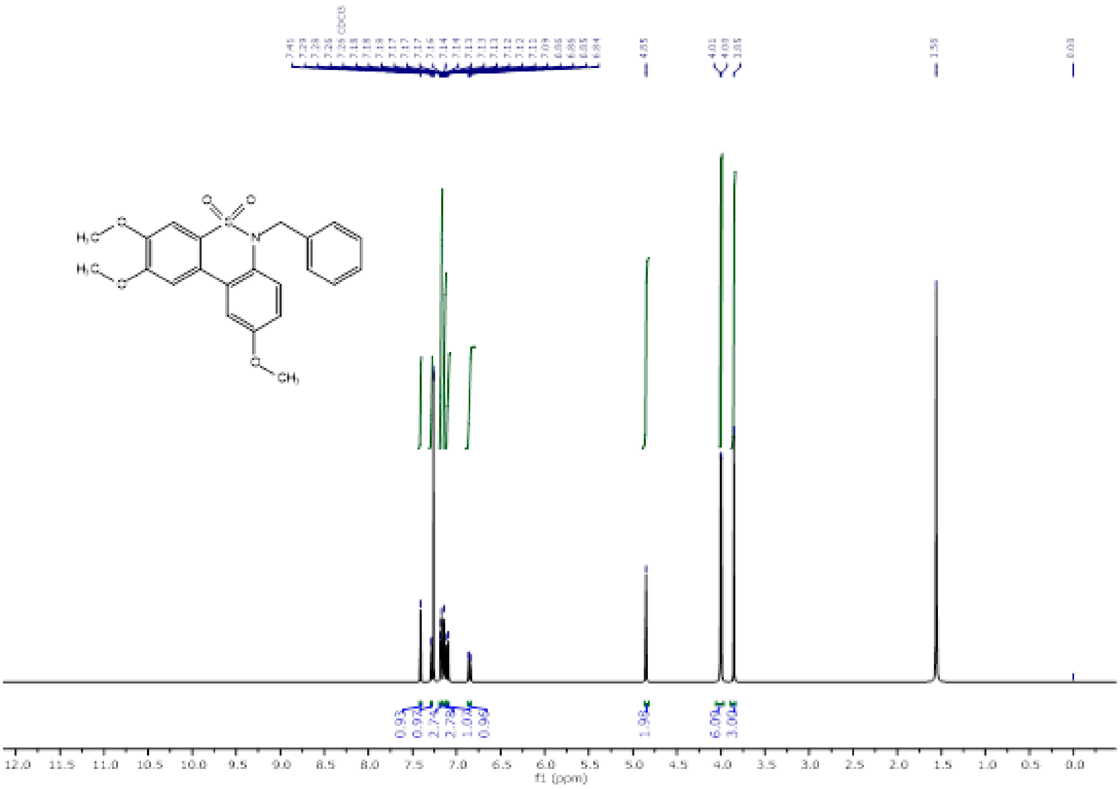

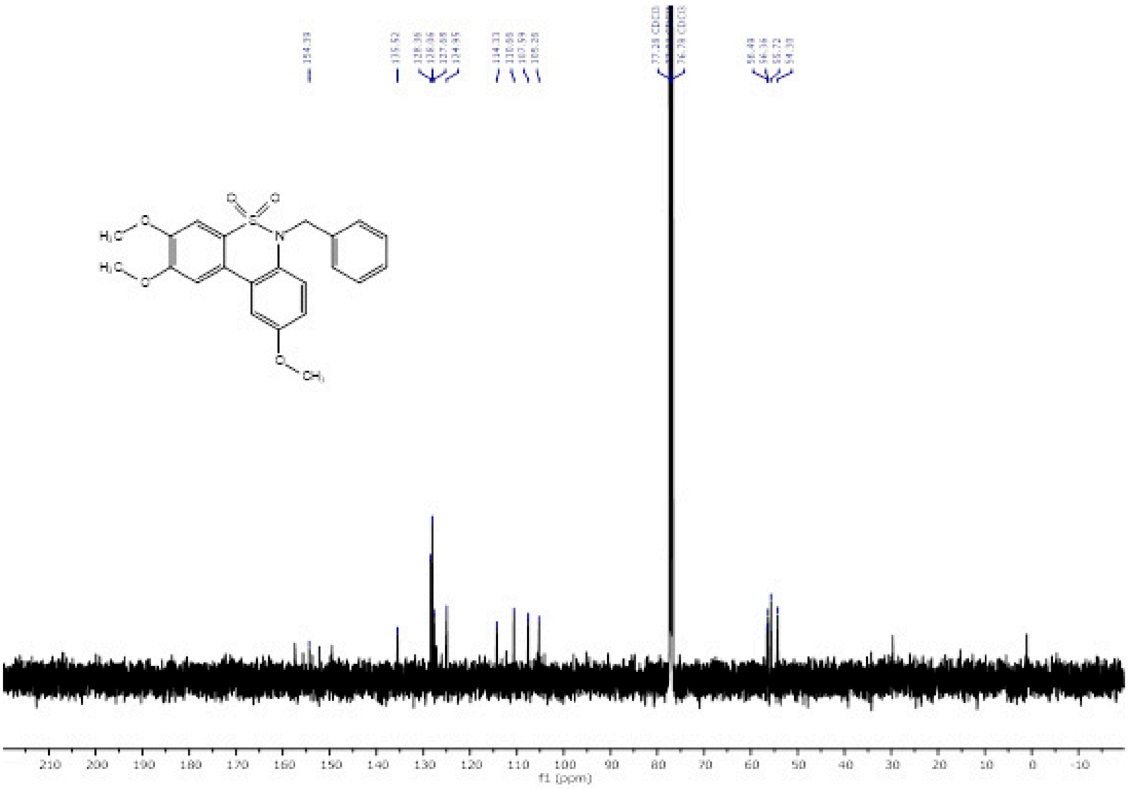

6-benzyl-2,3,8-trimethoxy-6H-dibenzo[c,e][1,2]thiazine 5,5-dioxide ***(14b)****:*

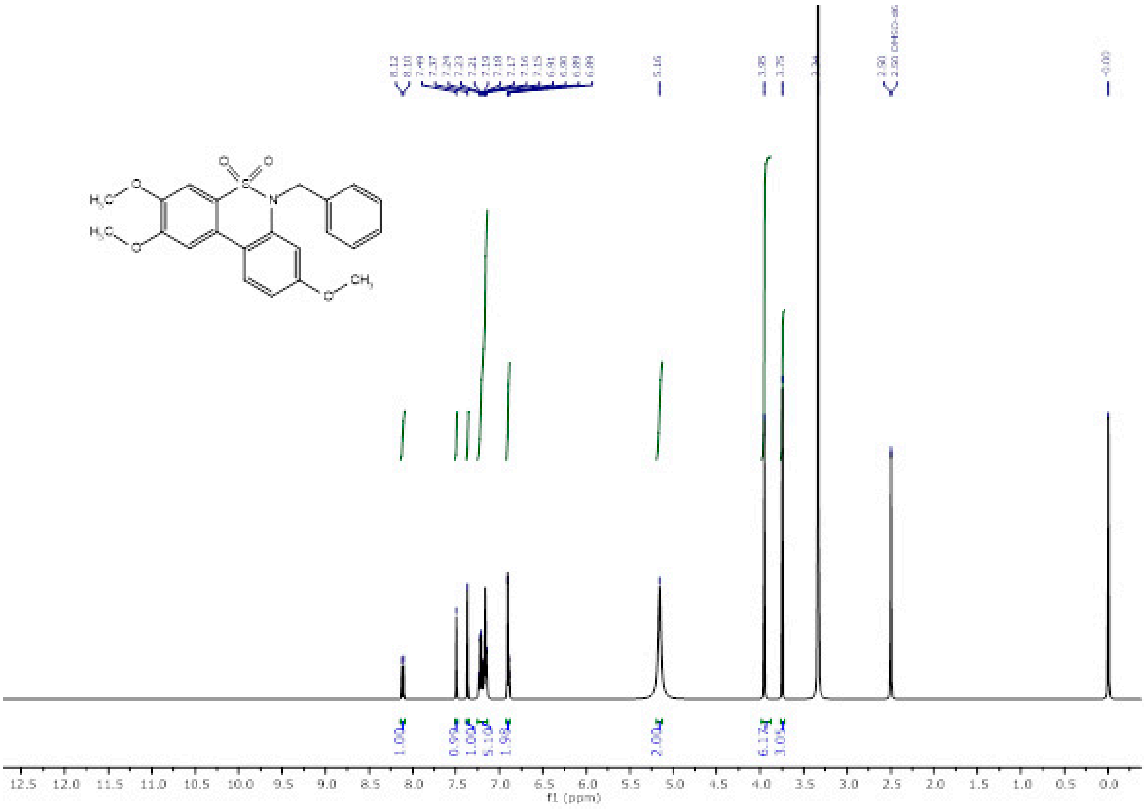

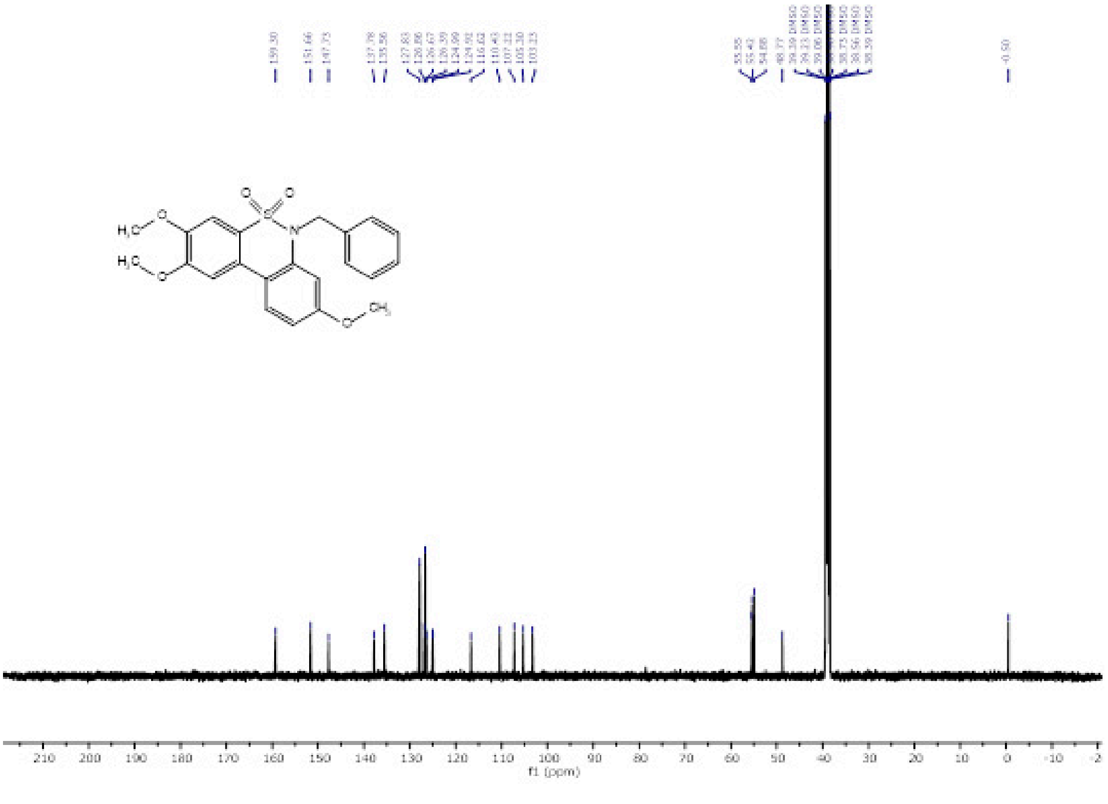

6-benzyl-2,3,7,9-tetramethoxy-6H-dibenzo[c,e][1,2]thiazine 5,5-dioxide ***(14c)***:

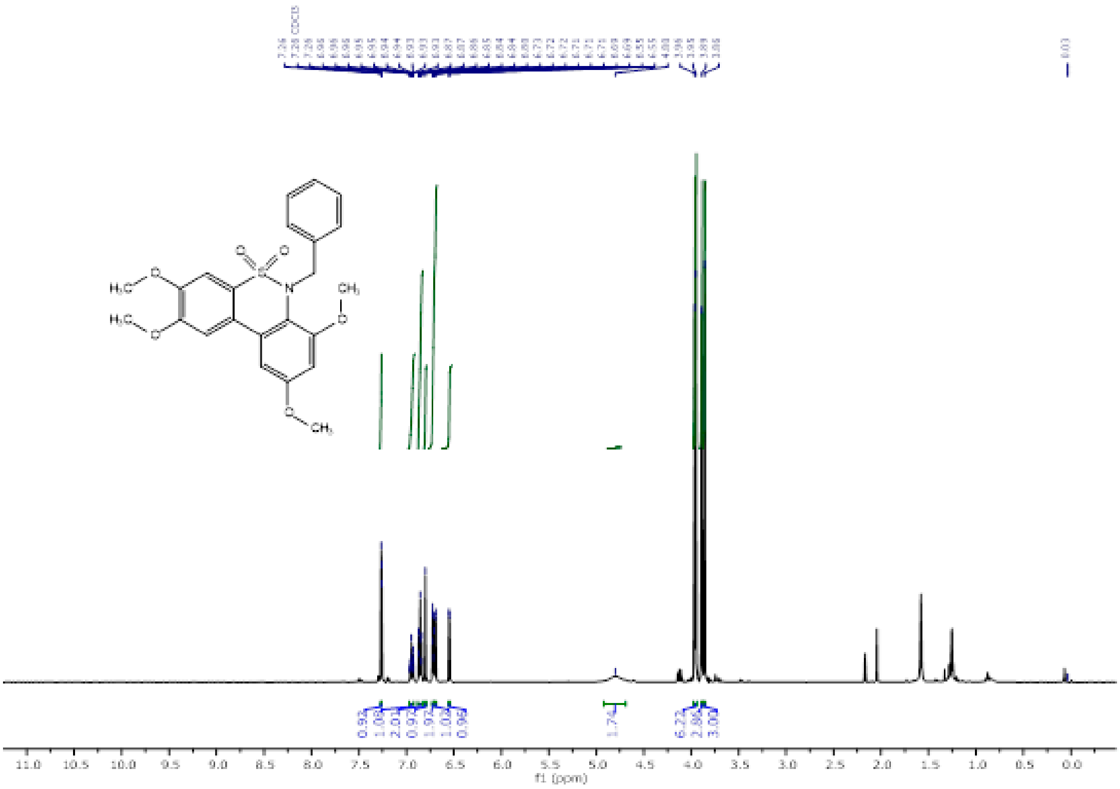

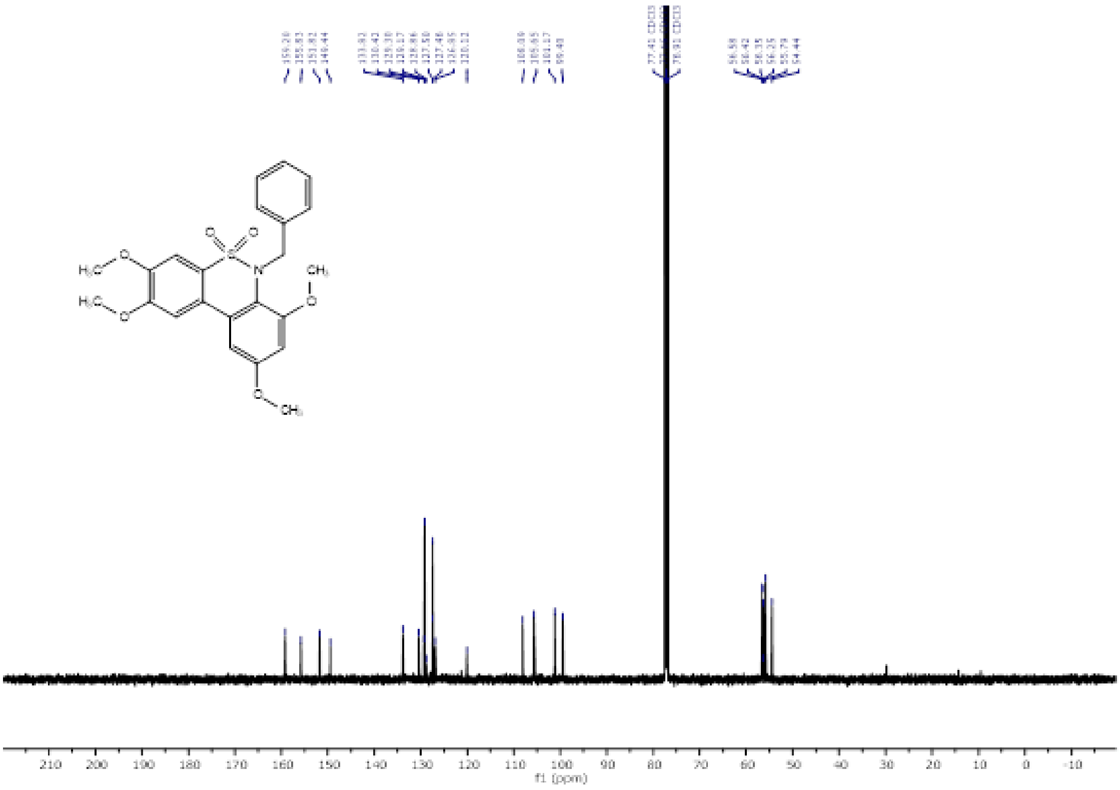

6-benzyl-2,3,8,9-tetramethoxy-6H-dibenzo[c,e][1,2]thiazine 5,5-dioxide ***(14d)***:

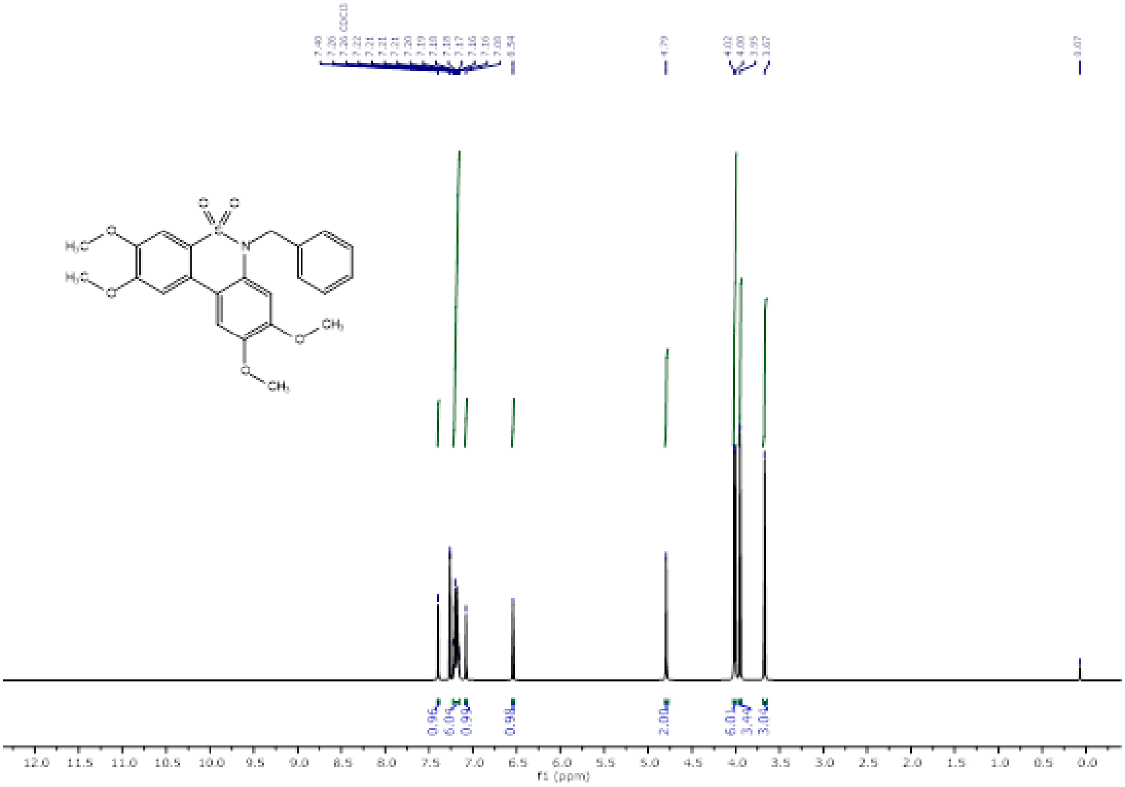

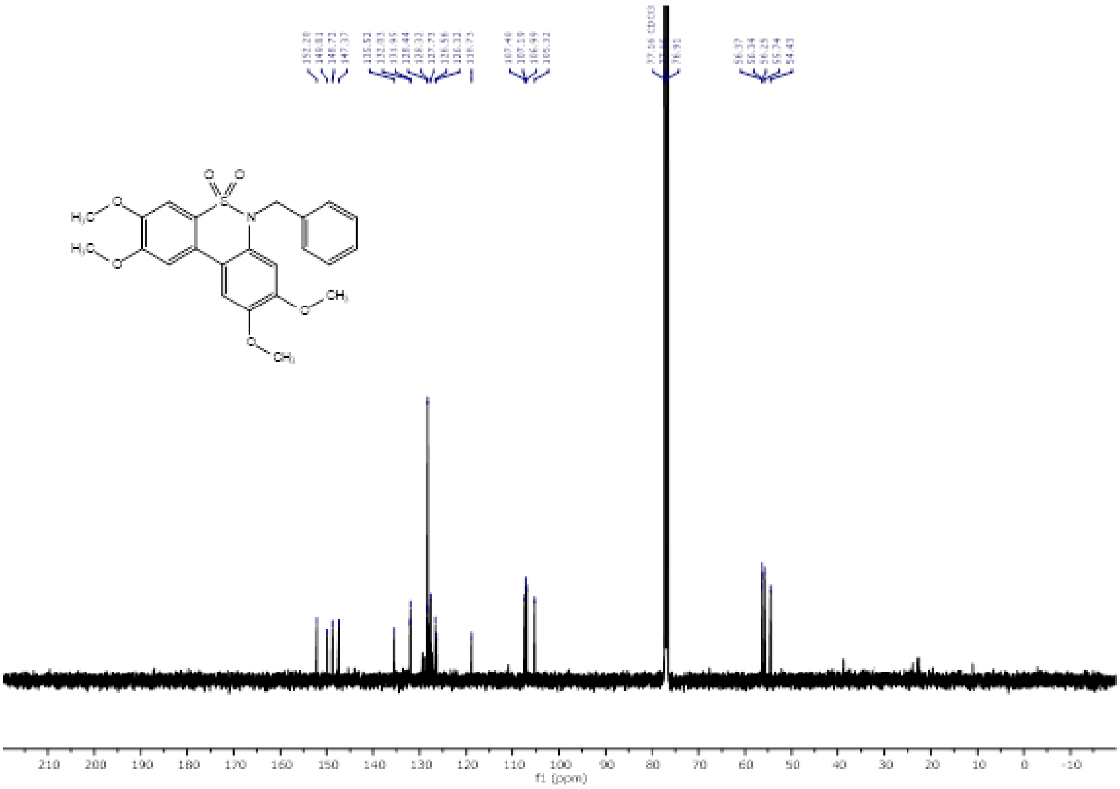

6-benzyl-2,3-dimethoxy-8-(trifluoromethyl)-6H-dibenzo[c,e][1,2]thiazine 5,5-dioxide ***(14e)****:*

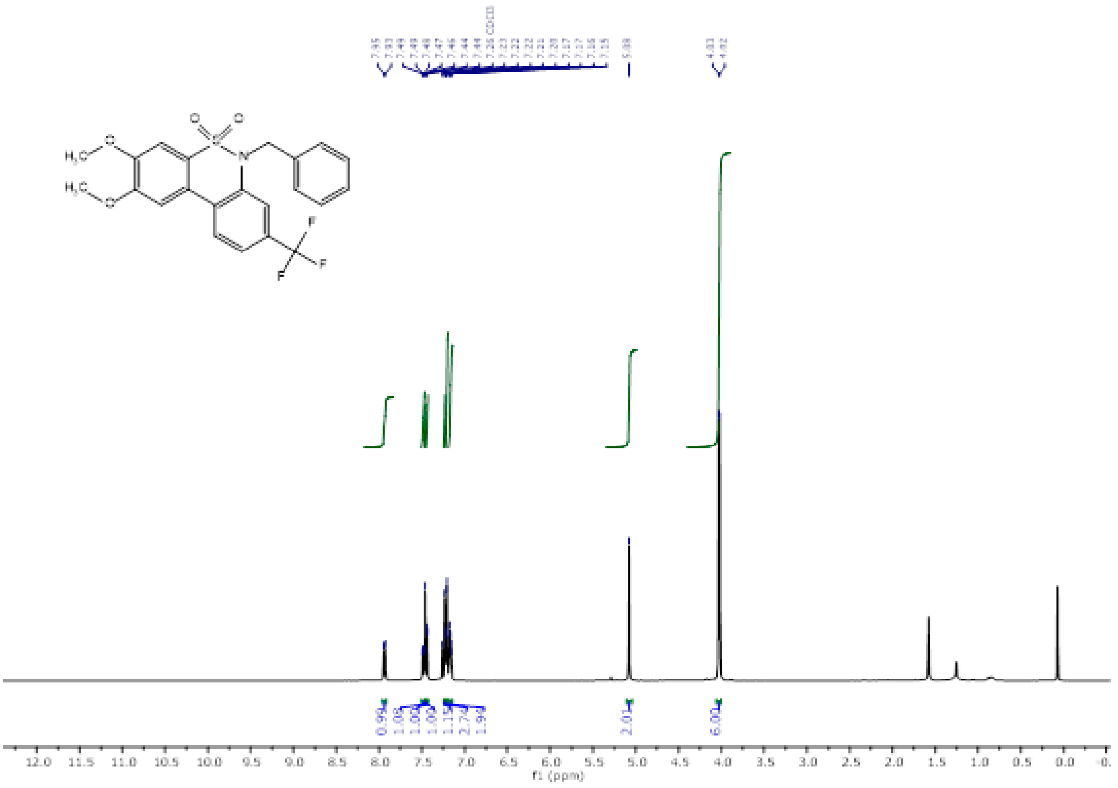

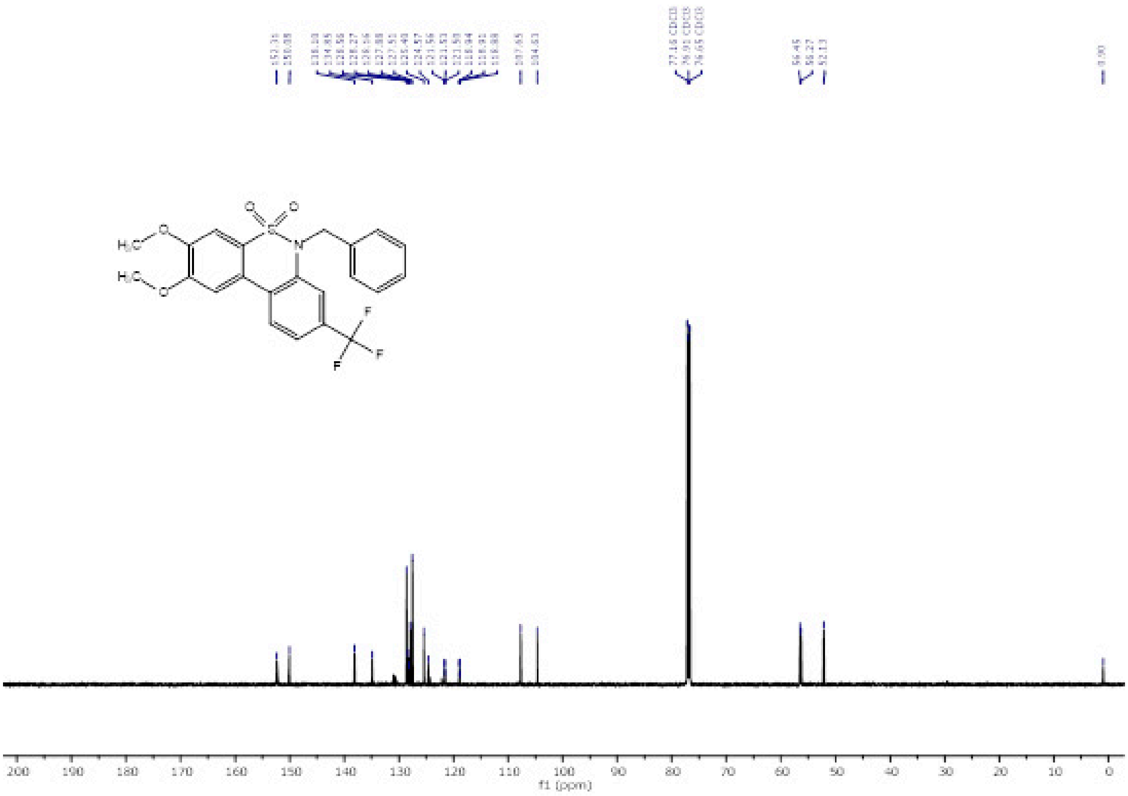

6-benzyl-9-fluoro-2,3,8-trimethoxy-6H-dibenzo[c,e][1,2]thiazine 5,5-dioxide ***(14f)****:*

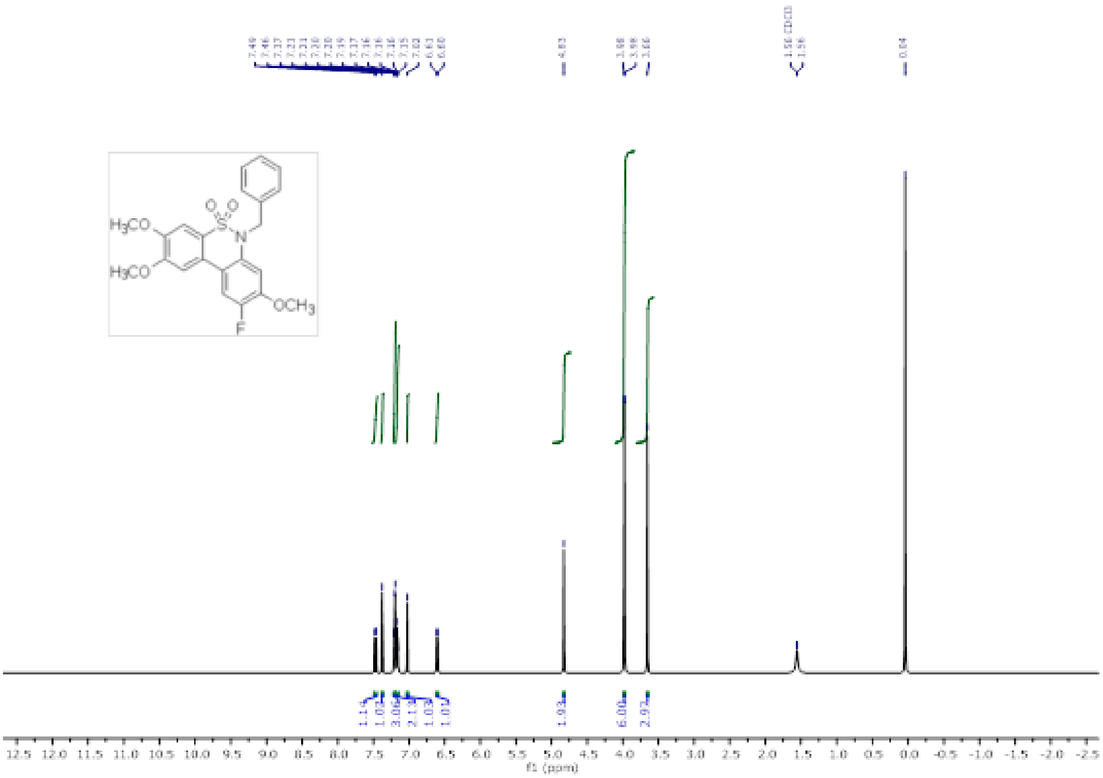

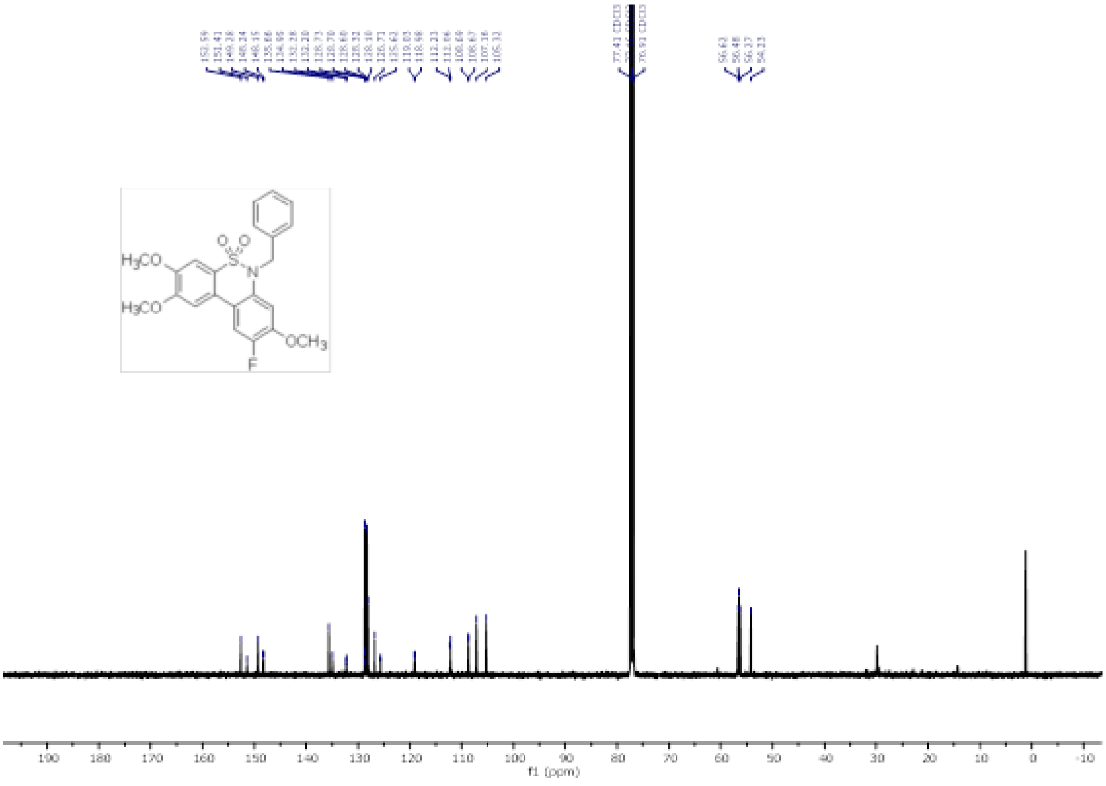

6-benzyl-2,3,8-trimethoxy-9-(trifluoromethyl)-6H-dibenzo[c,e][1,2]thiazine 5,5-dioxide ***(14g)***:

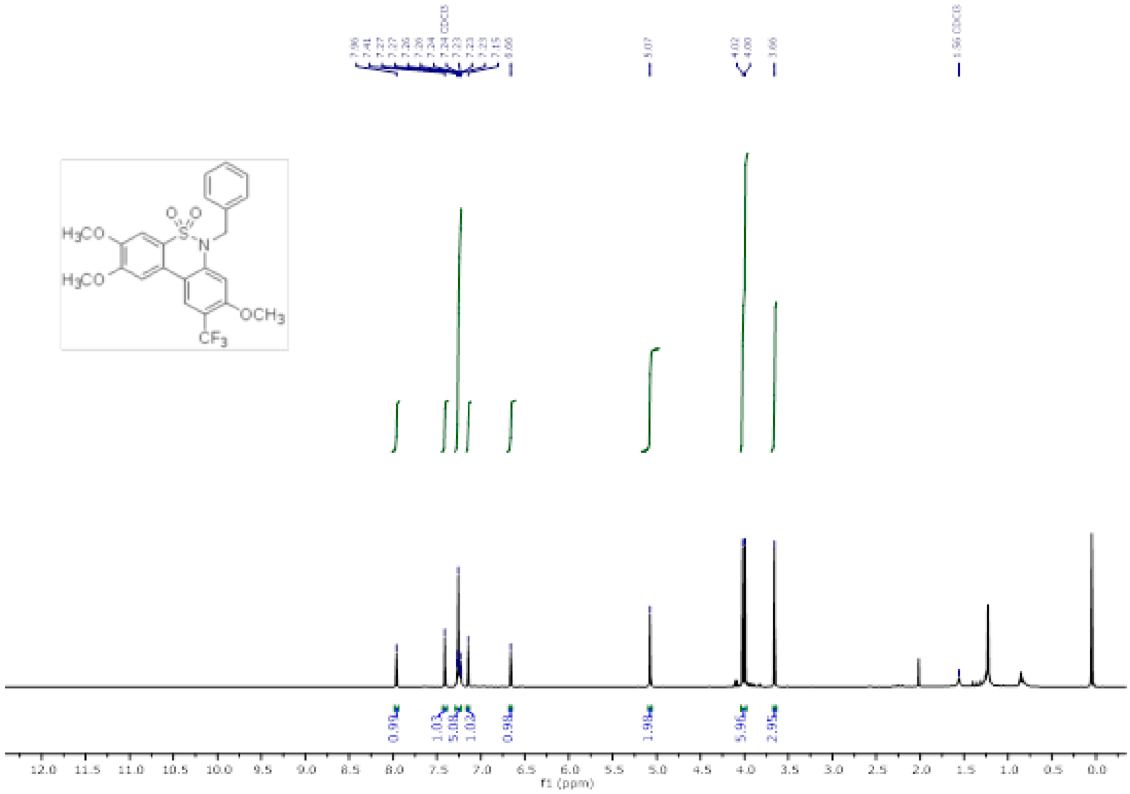

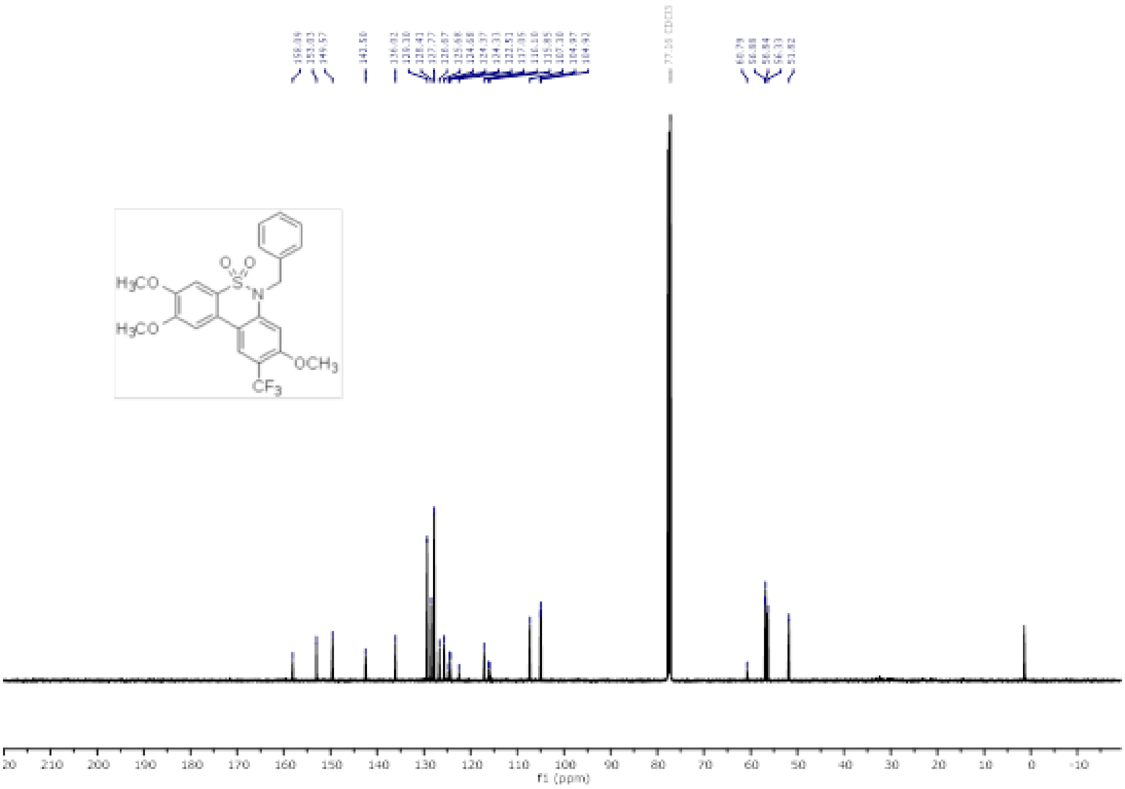

6-benzyl-2,3-dimethoxy-8-(trifluoromethoxy)-6H-dibenzo[c,e][1,2]thiazine 5,5-dioxide ***(14h)****:*

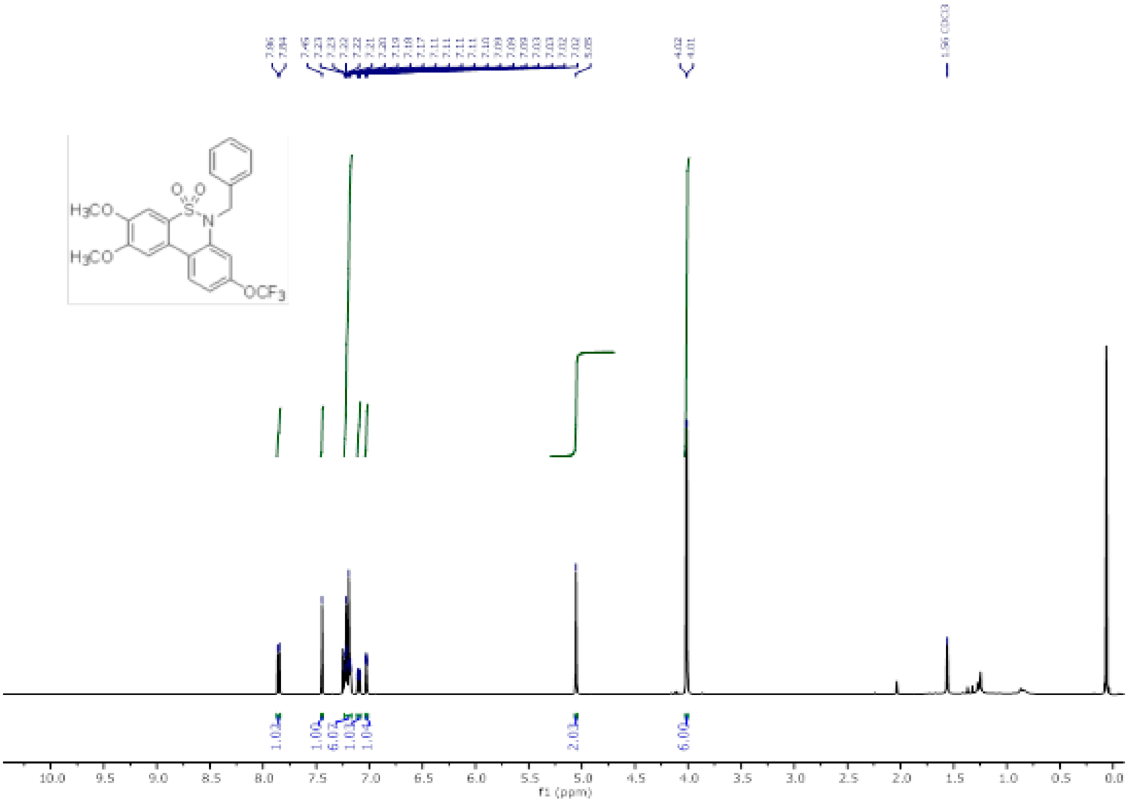

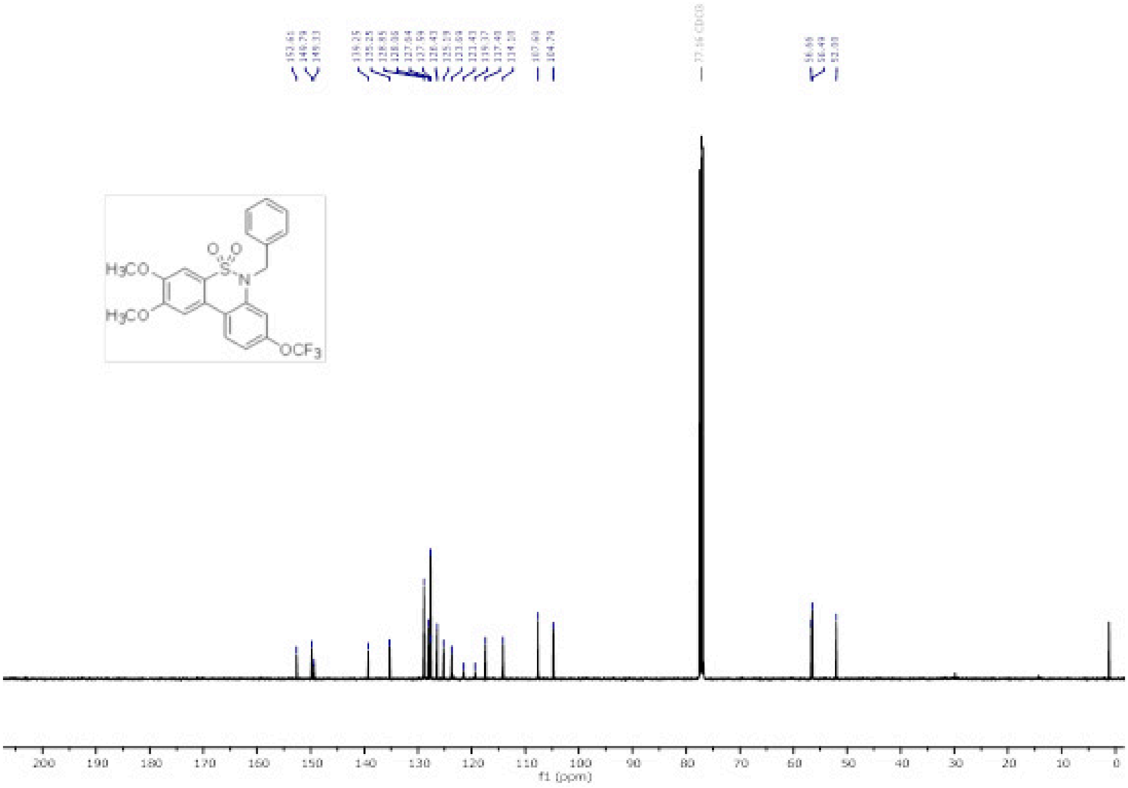

6-benzyl-2,3,9-trihydroxy-6H-dibenzo[c,e][1,2]thiazine 5,5-dioxide ***(15a)****:*

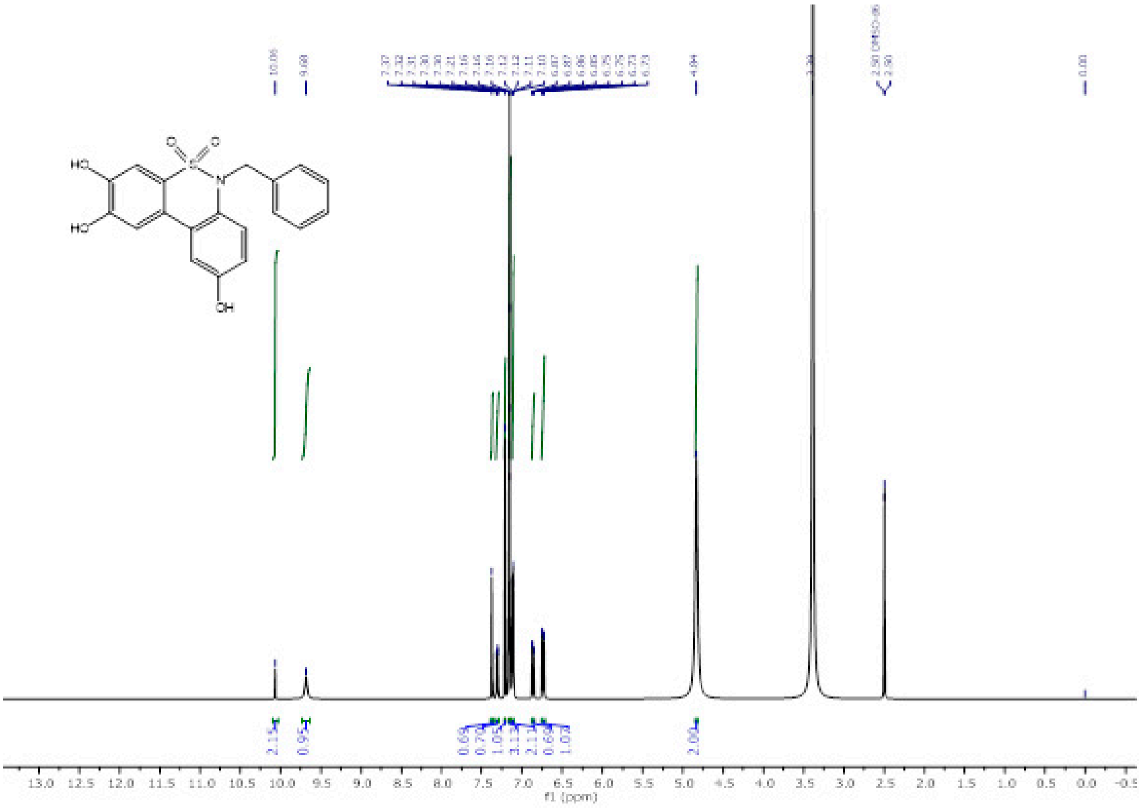

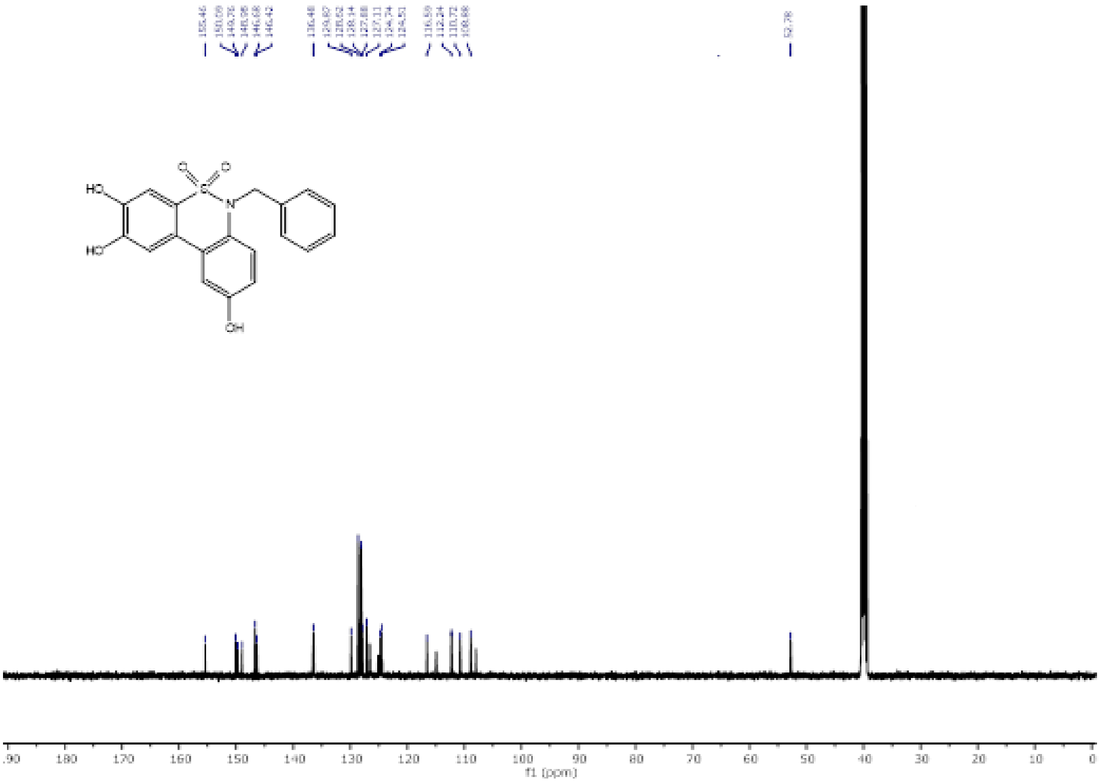

6-benzyl-2,3,8-trihydroxy-6H-dibenzo[c,e][1,2]thiazine 5,5-dioxide ***(15b)****:*

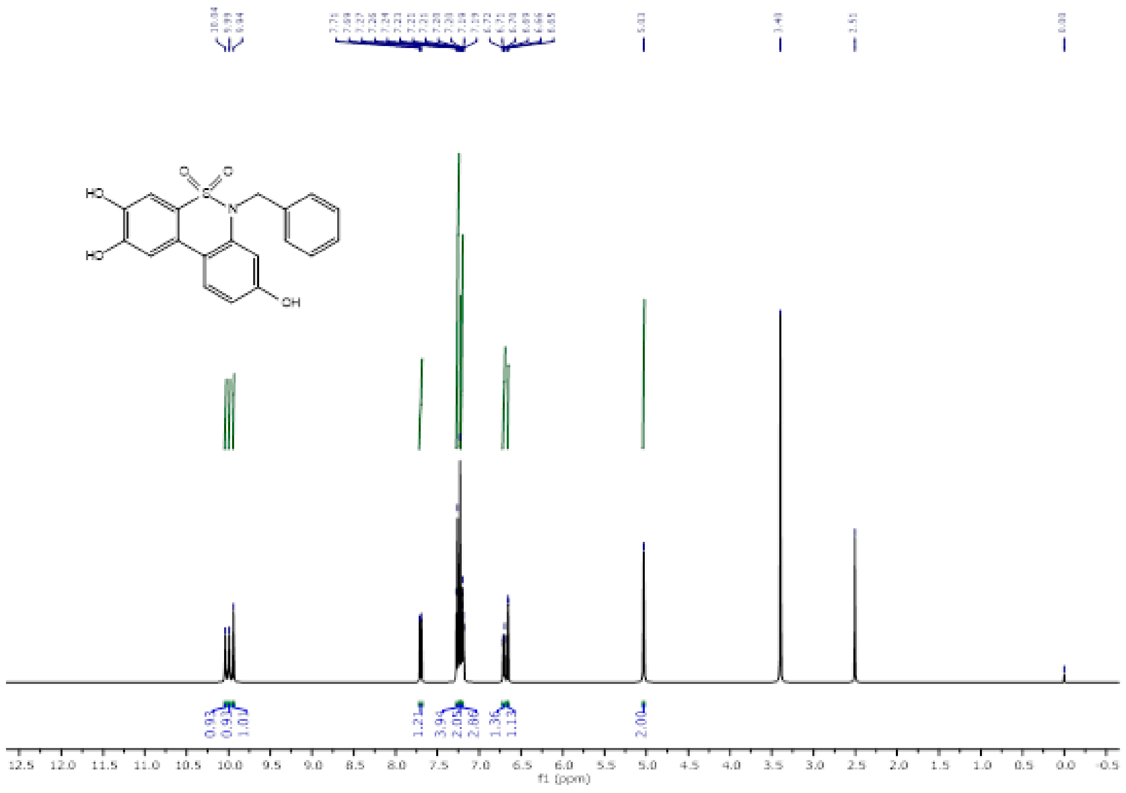

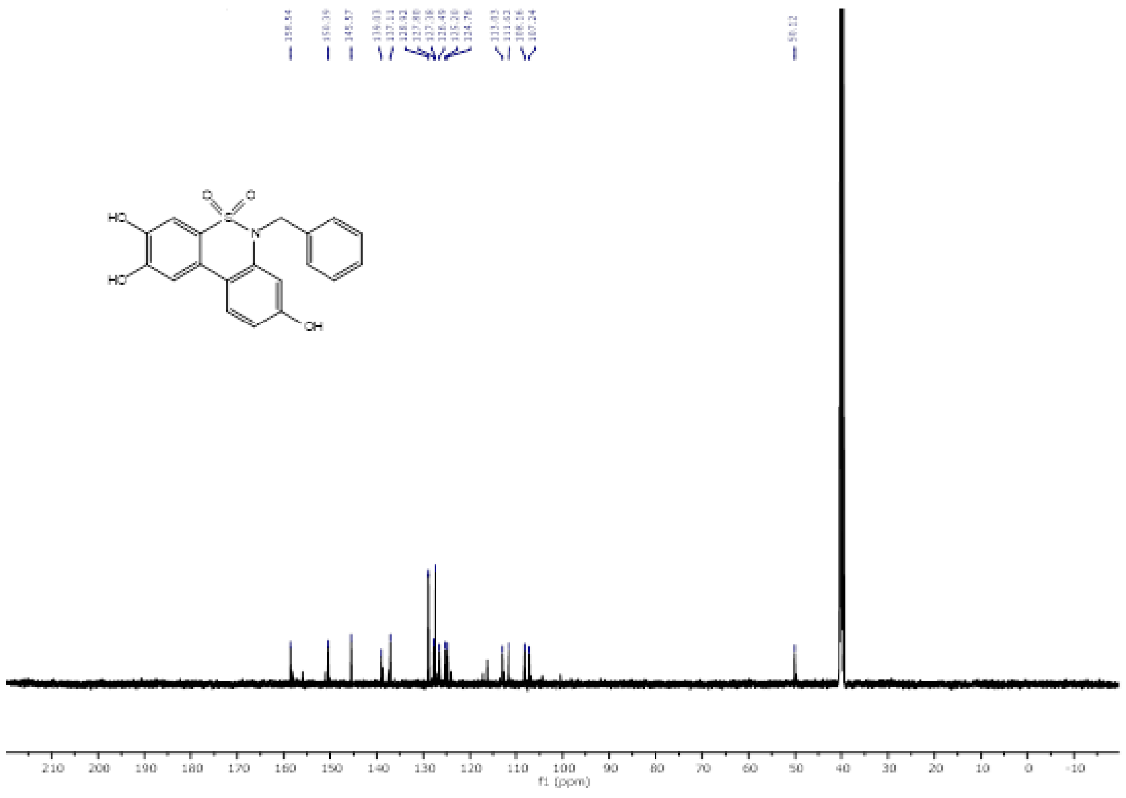

6-benzyl-2,3,7,9-tetrahydroxy-6H-dibenzo[c,e][1,2]thiazine 5,5-dioxide ***(15c)***:

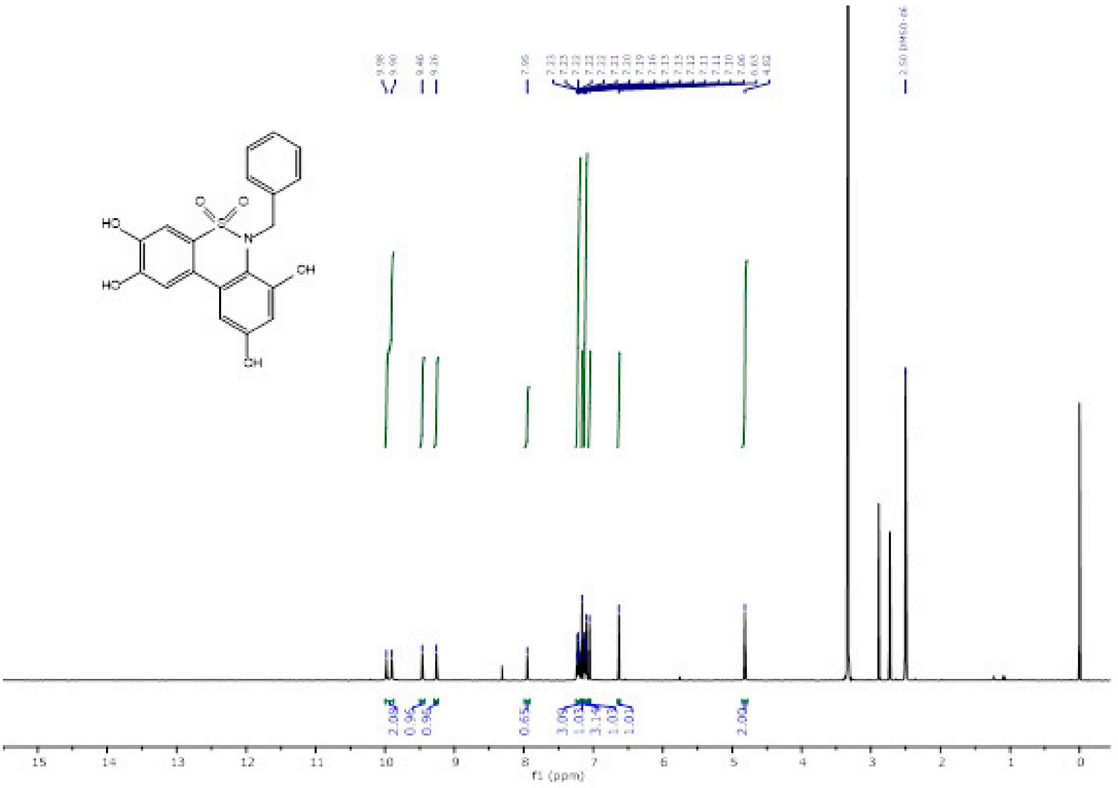

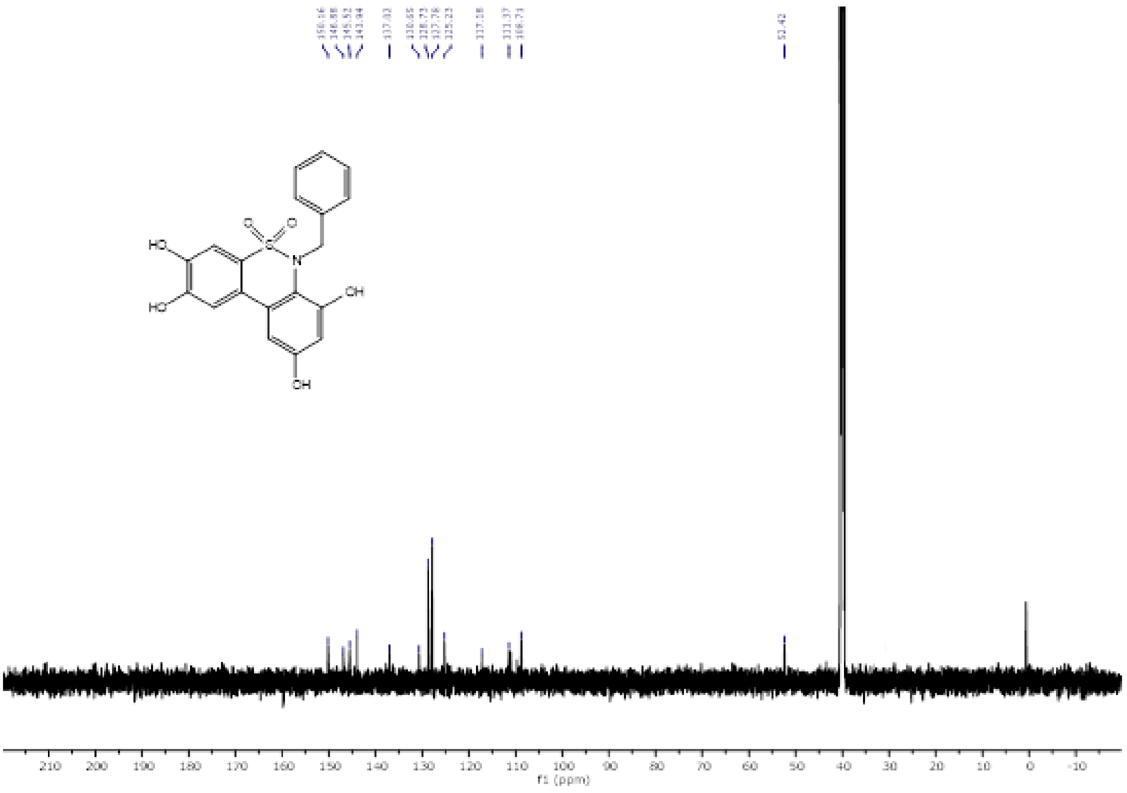

6-benzyl-2,3,8,9-tetrahydroxy-6H-dibenzo[c,e][1,2]thiazine 5,5-dioxide ***(15d)***:

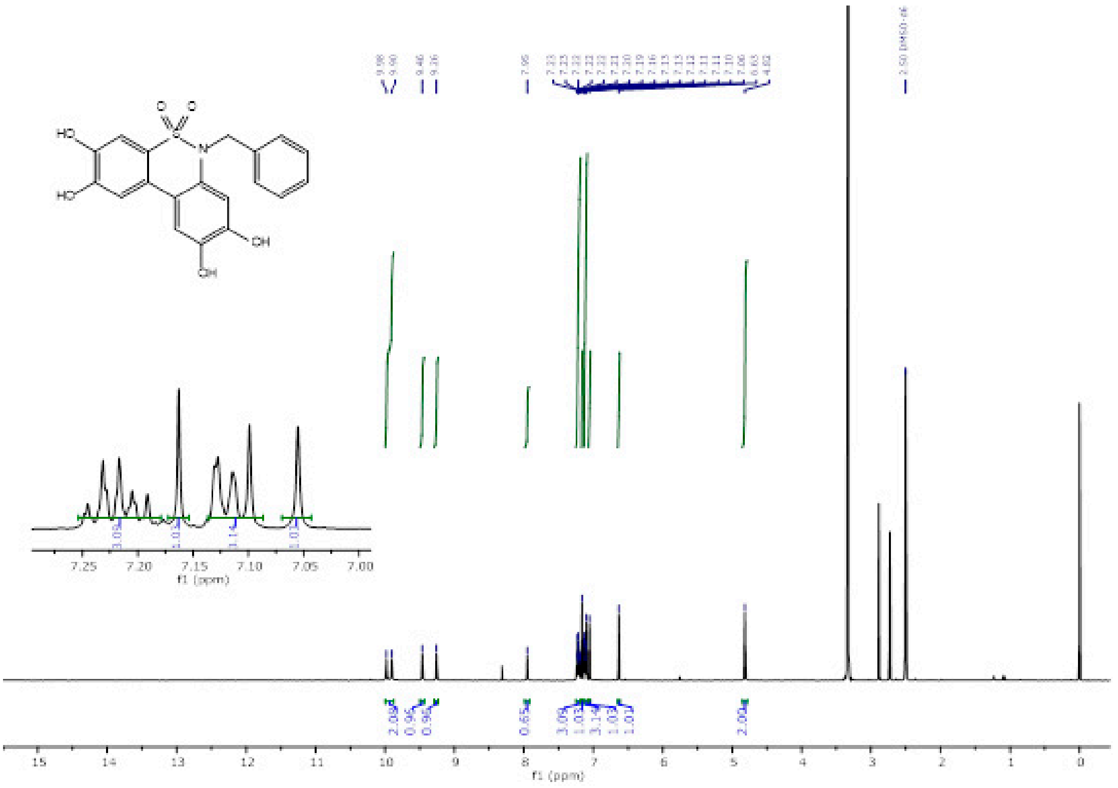

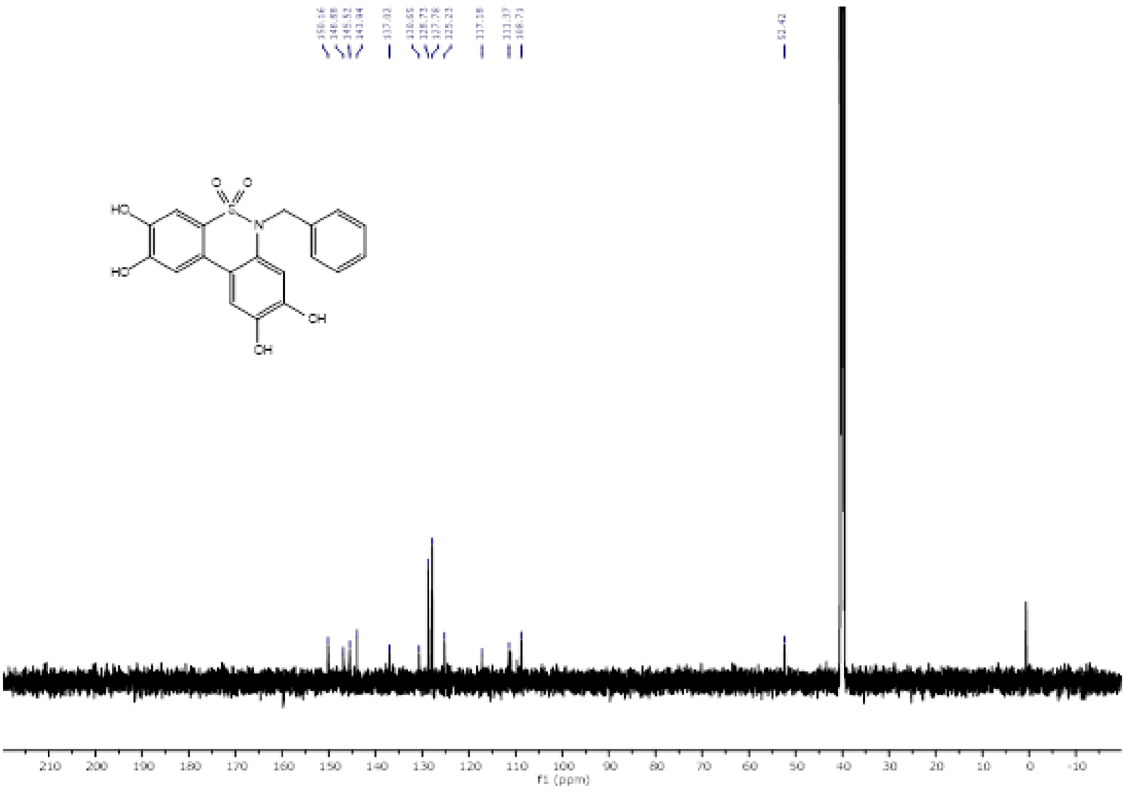

6-benzyl-2,3-dihydroxy-8-(trifluoromethyl)-6H-dibenzo[c,e][1,2]thiazine 5,5-dioxide ***(15e)****:*

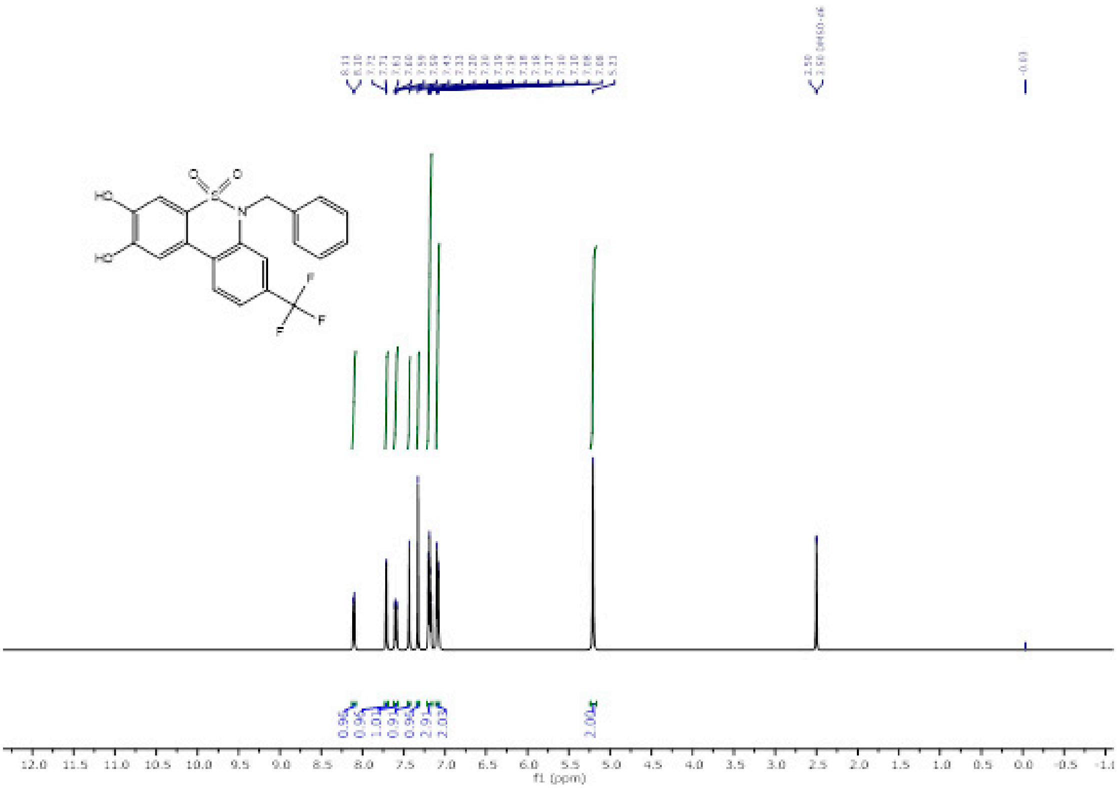

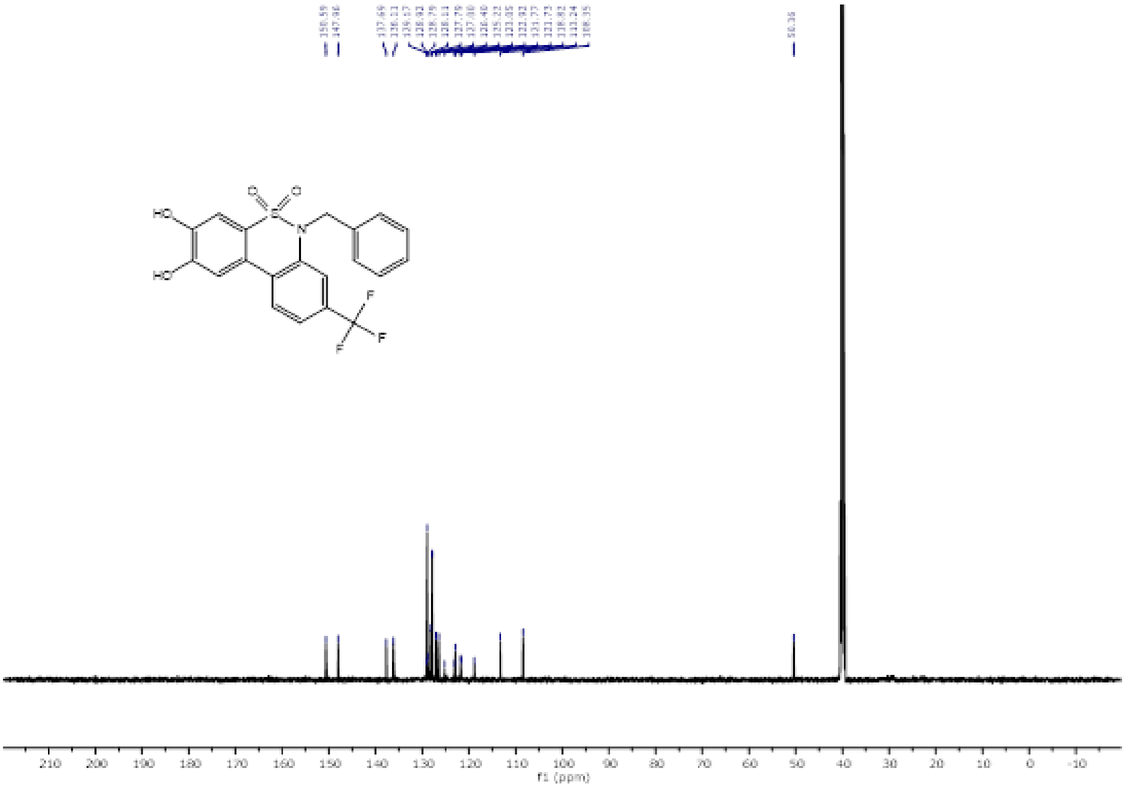

6-benzyl-9-fluoro-2,3,8-trihydroxy-6H-dibenzo[c,e][1,2]thiazine 5,5-dioxide ***(15f)****:*

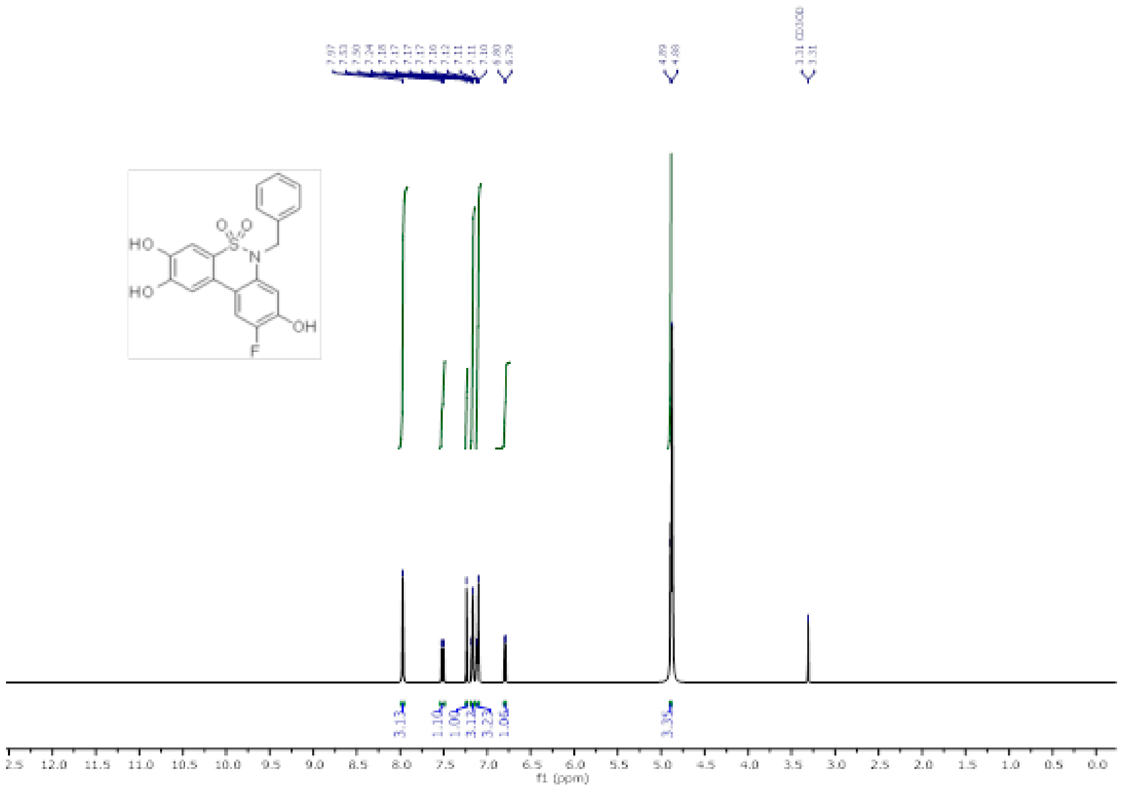

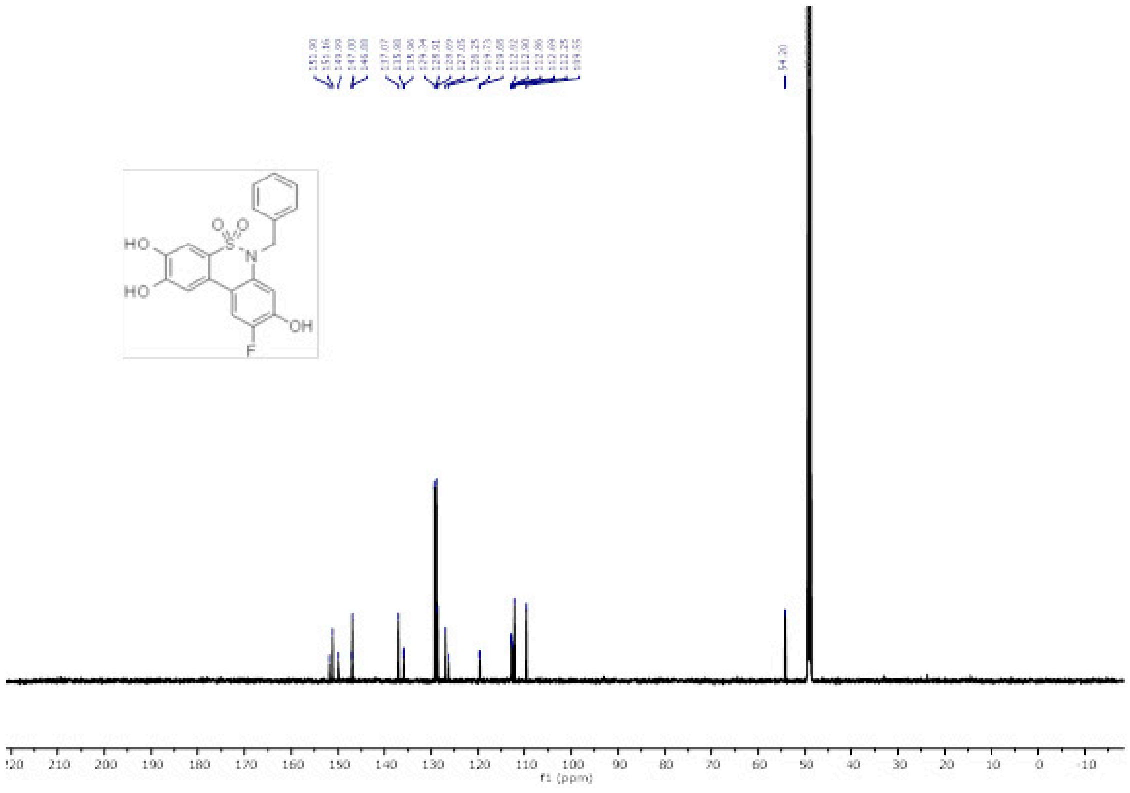

6-benzyl-2,3-dihydroxy-8-methoxy-9-(trifluoromethyl)-6H-dibenzo[c,e][1,2]thiazine 5,5-dioxide ***(15g)***:

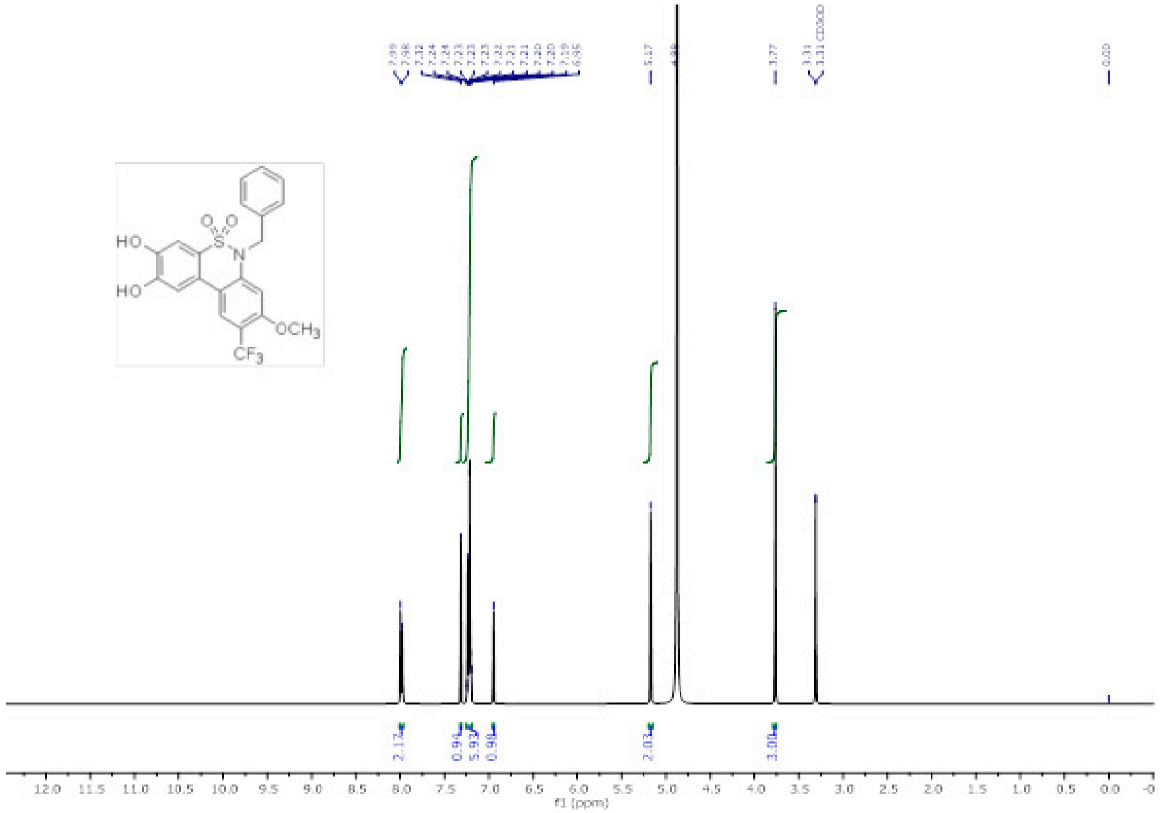

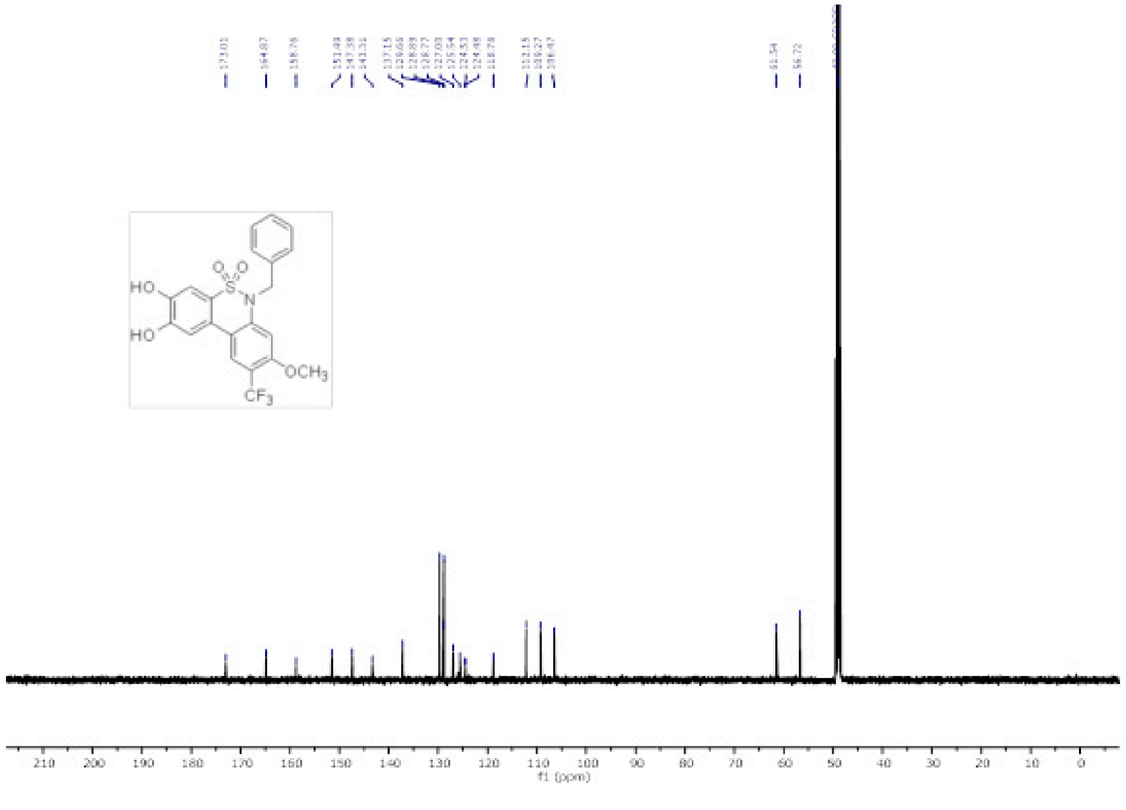

6-benzyl-2,3-dihydroxy-8-(trifluoromethoxy)-6H-dibenzo[c,e][1,2]thiazine 5,5-dioxide ***(15h)****:*

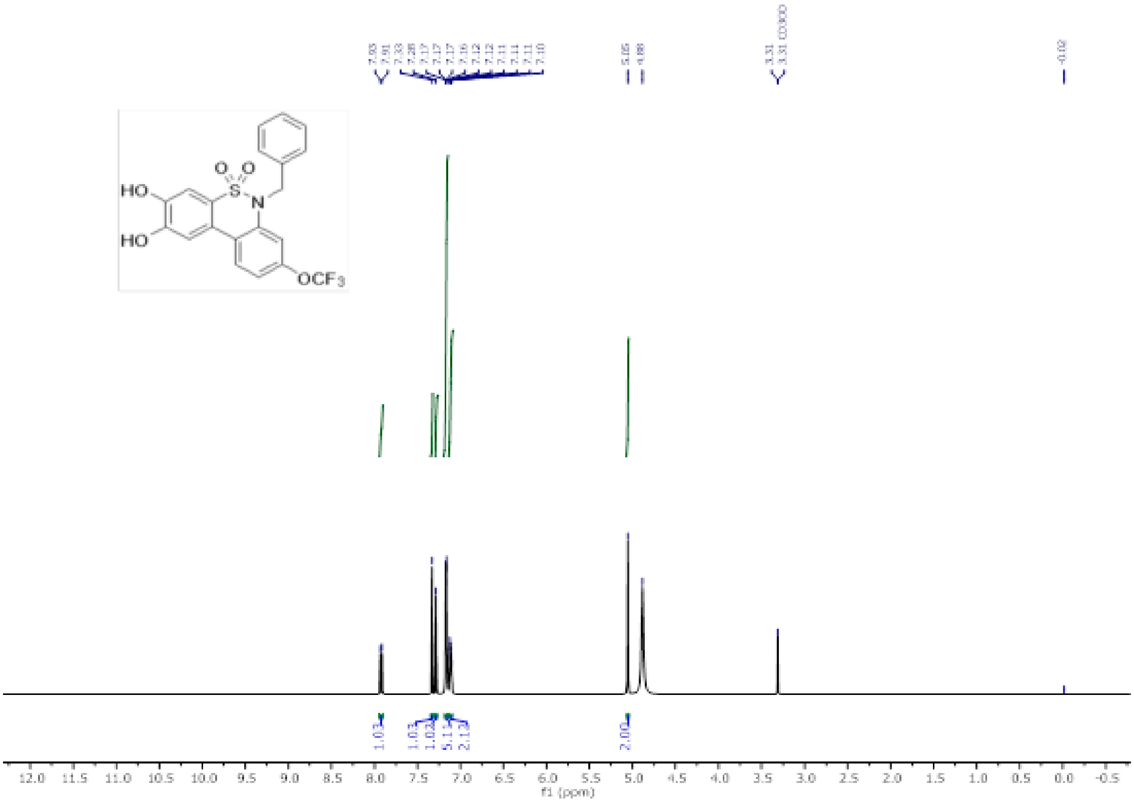

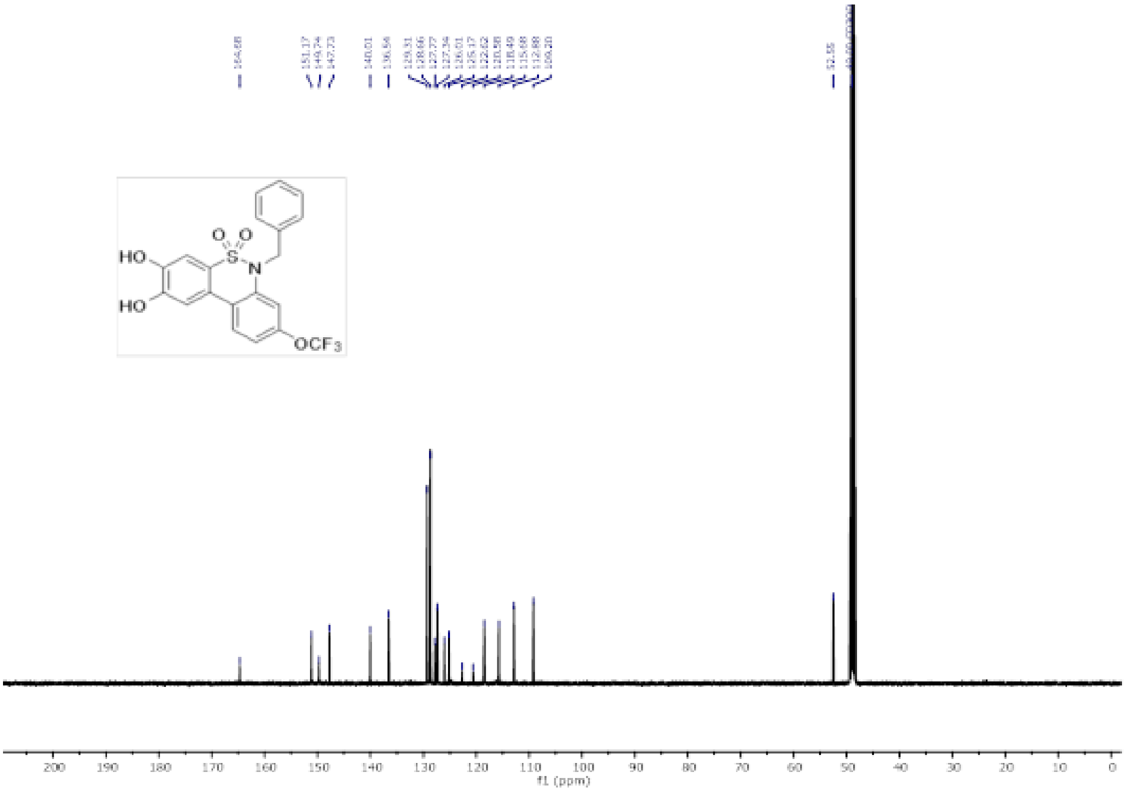

*6-(4-fluorobenzyl)-2,3,9-trimethoxy-6H-dibenzo[c,e][1,2]thiazine 5,5-dioxide **(17a)**:*

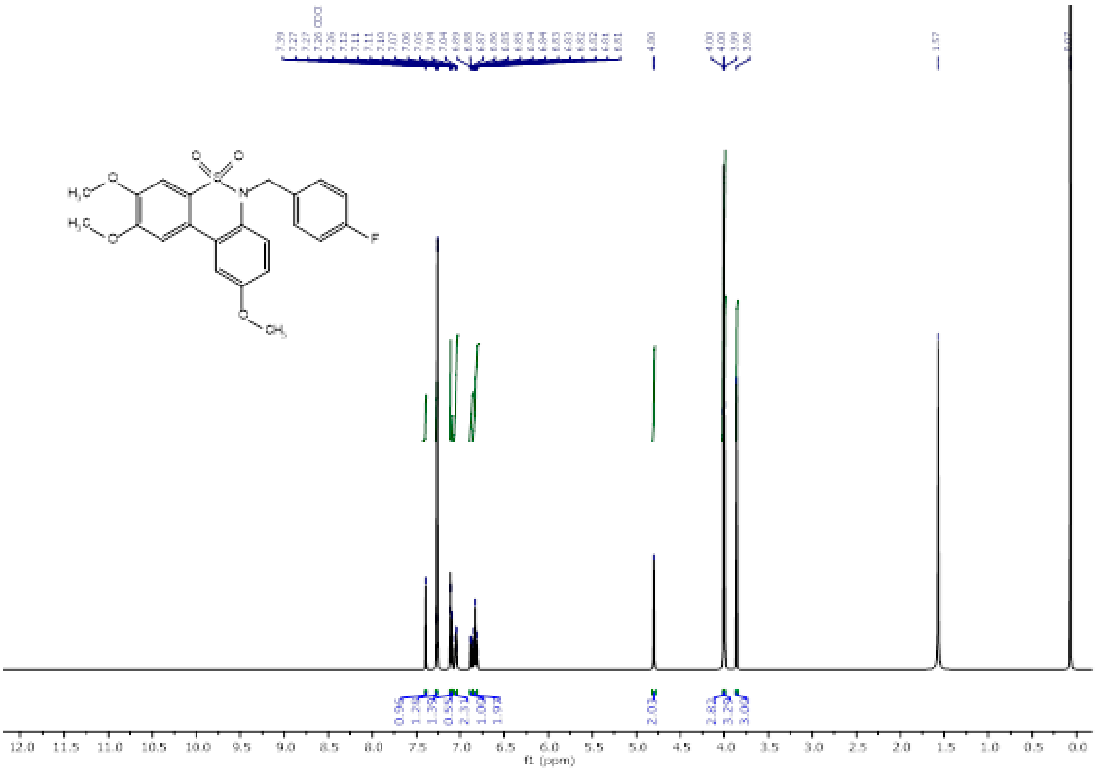

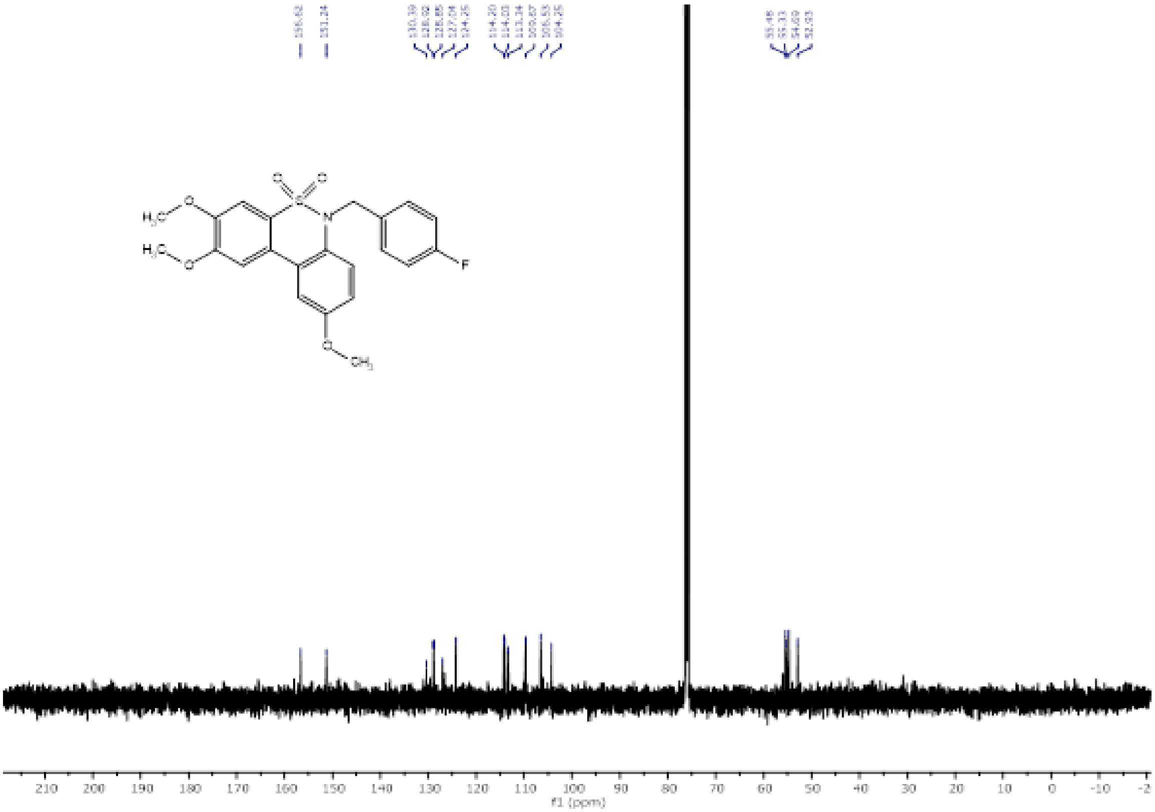

6-(4-fluorobenzyl)-2,3,8-trimethoxy-6H-dibenzo[c,e][1,2]thiazine 5,5-dioxide ***(17b)***:

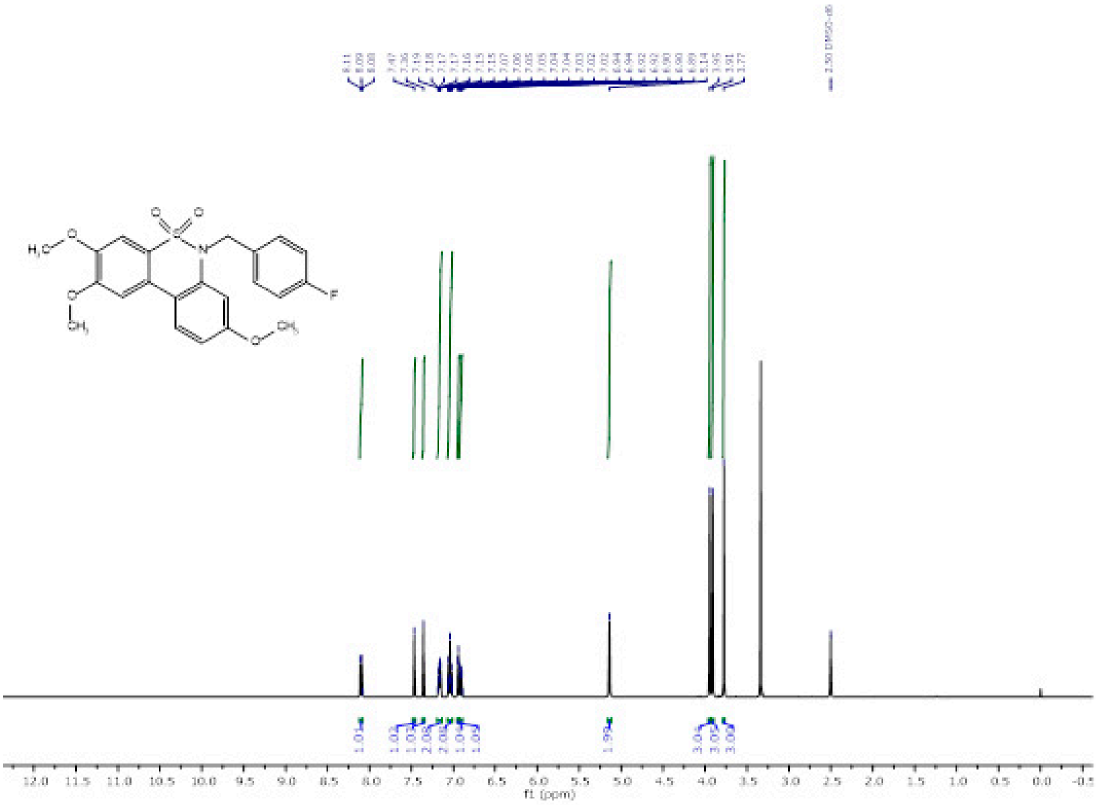

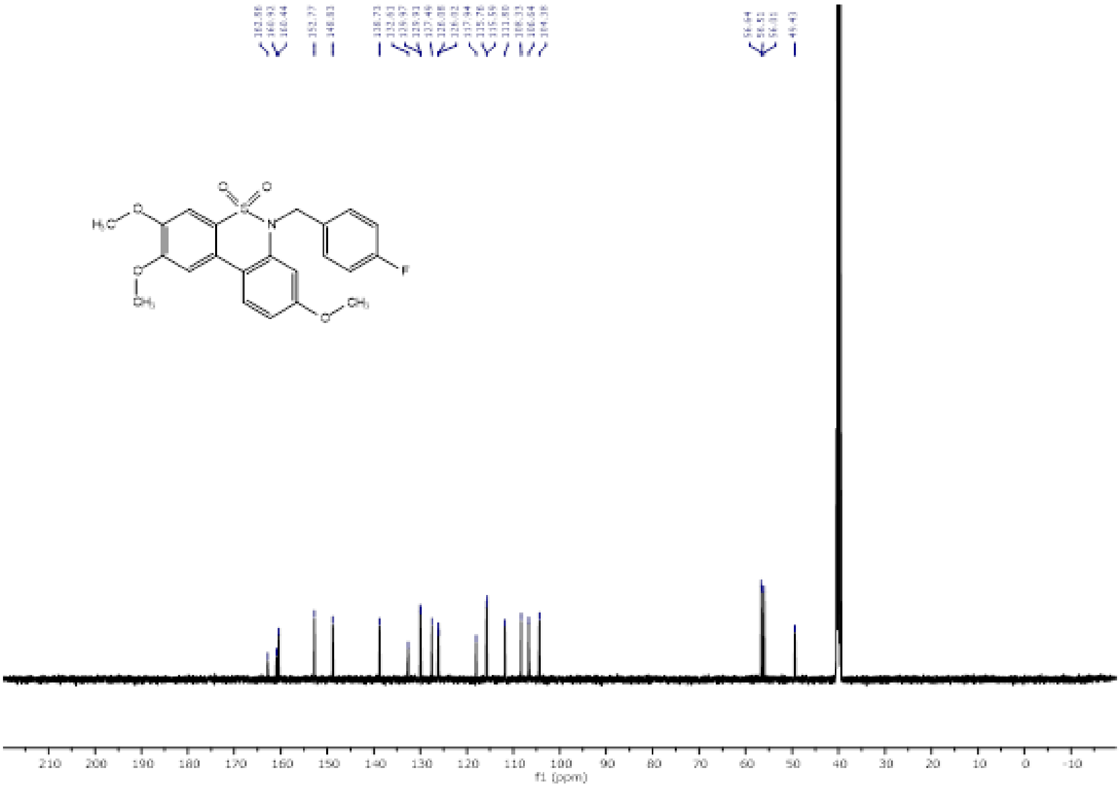

6-(4-fluorobenzyl)-2,3,7,9-tetramethoxy-6H-dibenzo[c,e][1,2]thiazine 5,5-dioxide ***(17c)***:

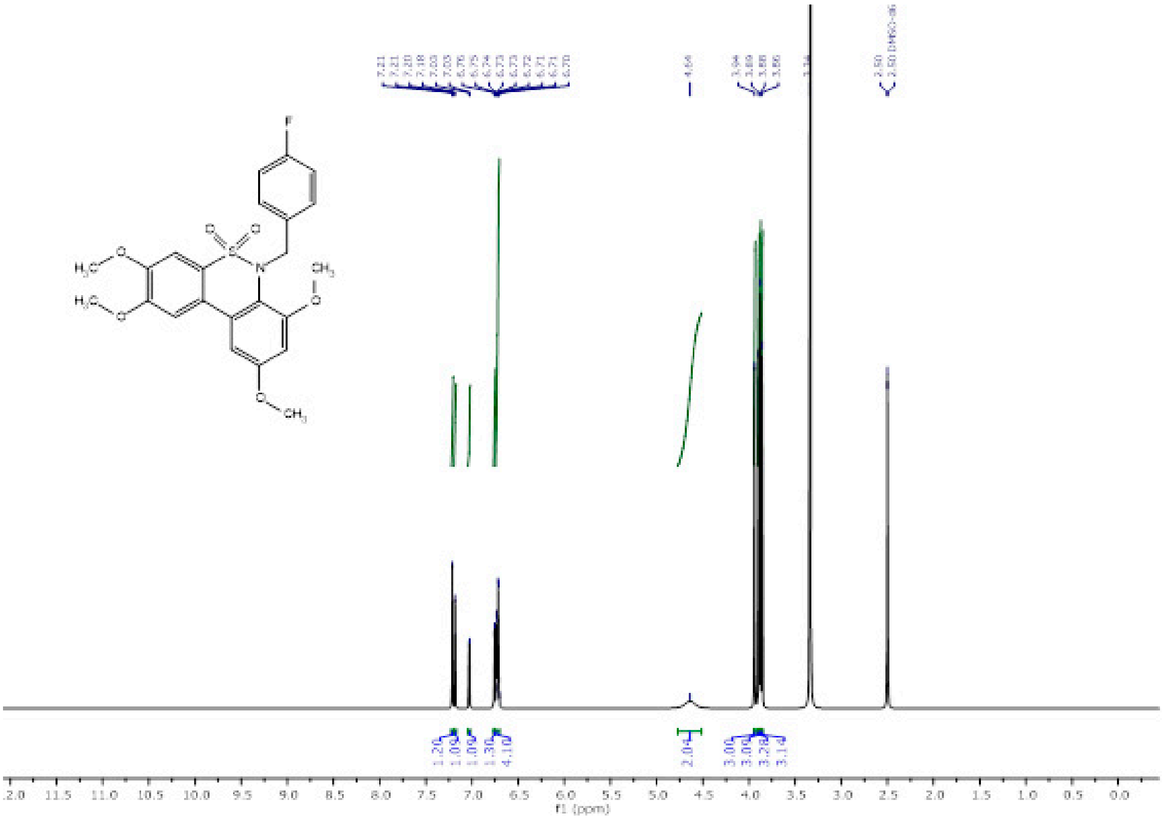

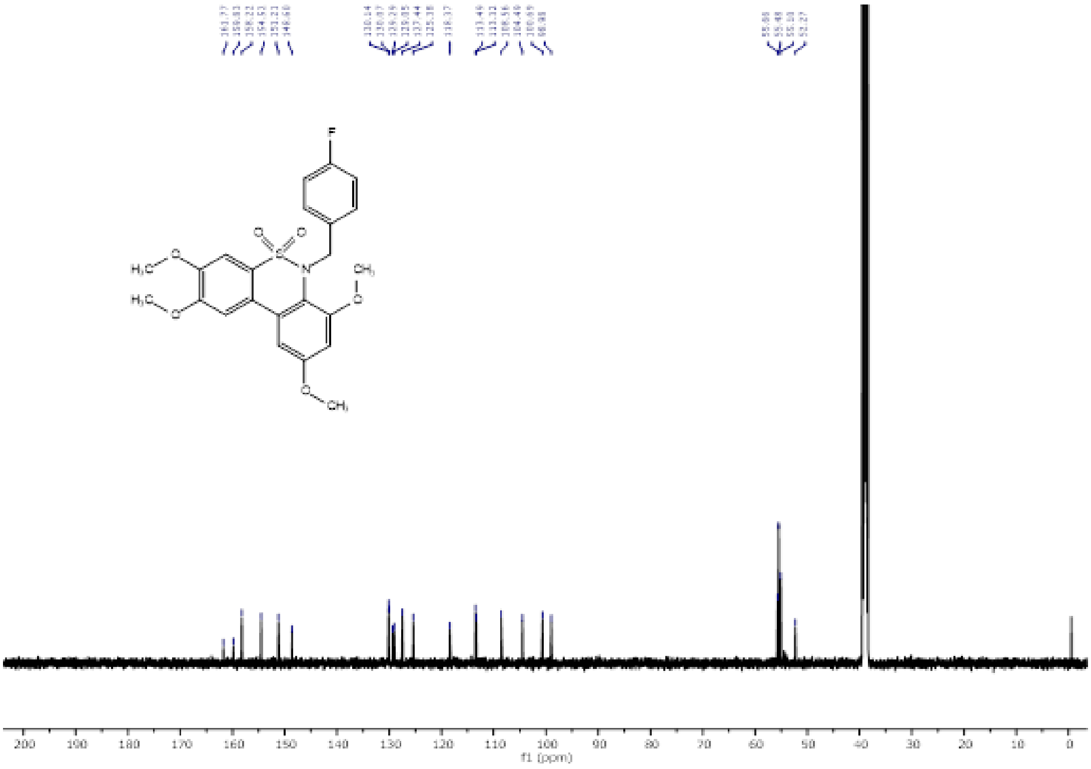

6-(4-fluorobenzyl)-2,3,8,9-tetramethoxy-6H-dibenzo[c,e][1,2]thiazine 5,5-dioxide ***(17d)****:*

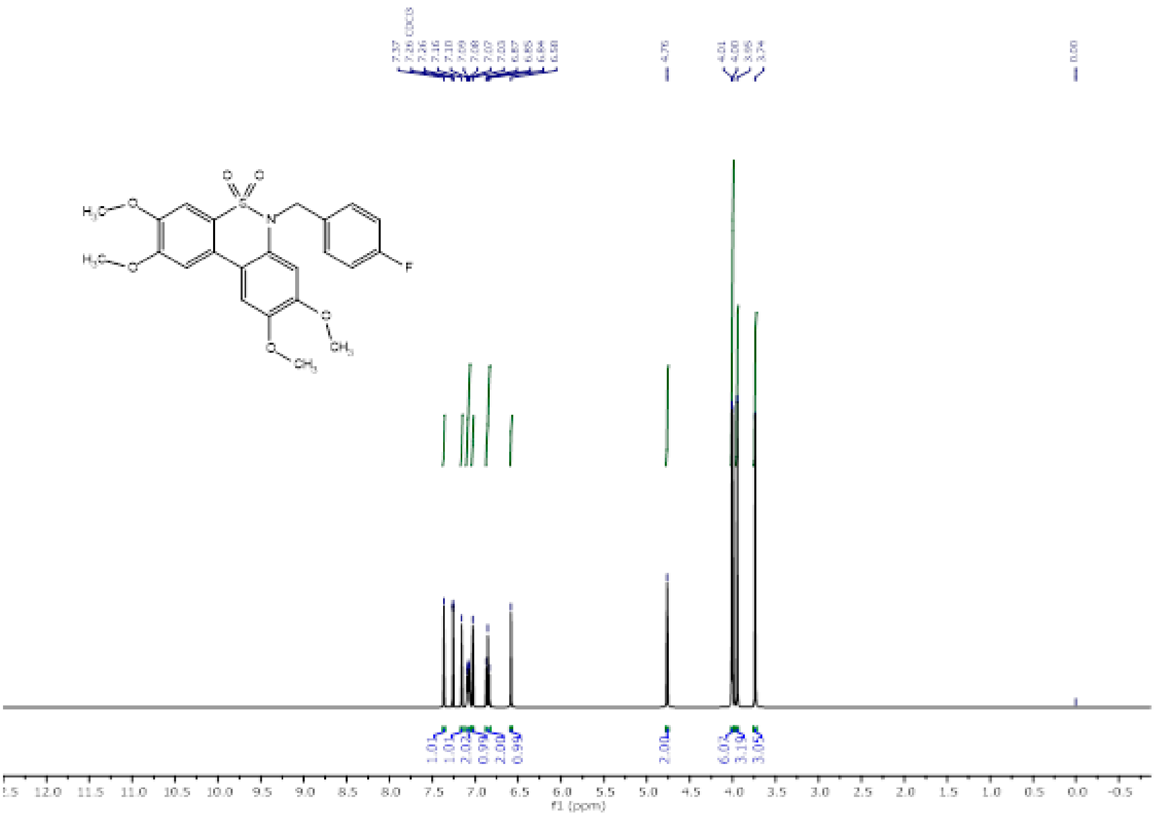

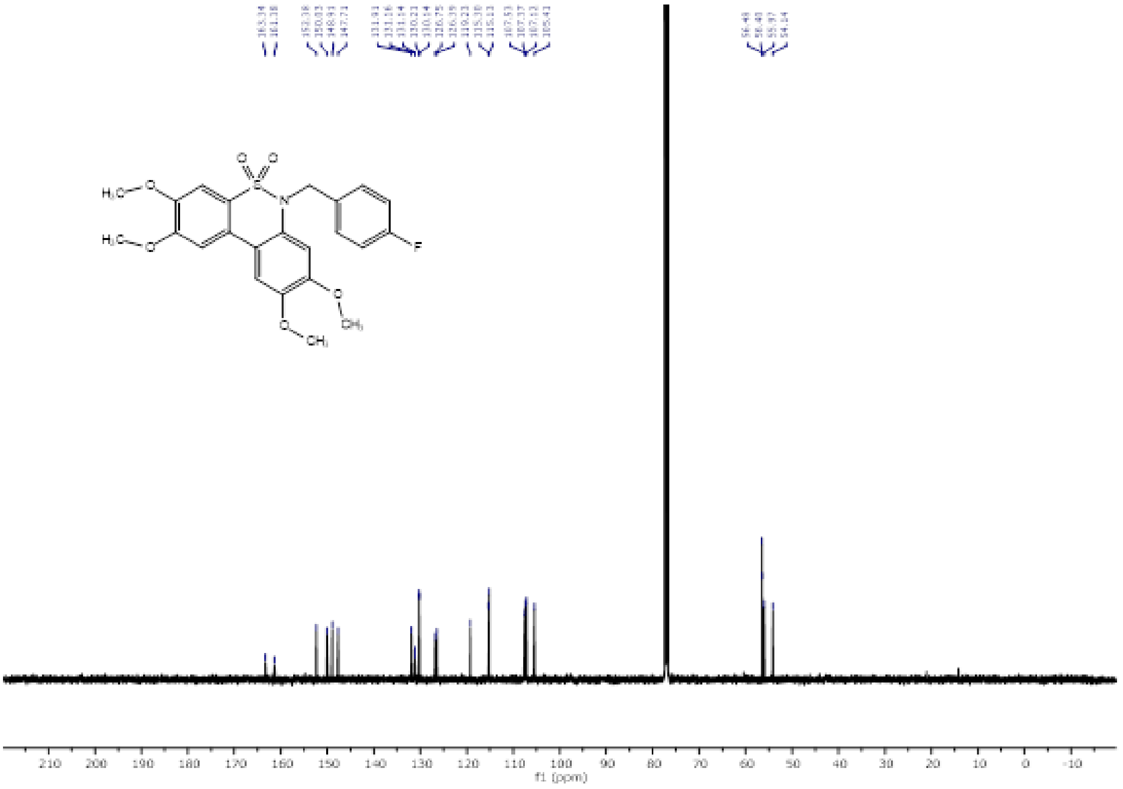

6-(4-fluorobenzyl)-2,3-dimethoxy-8-(trifluoromethyl)-6H-dibenzo[c,e][1,2]thiazine 5,5-dioxide ***(17e)****:*

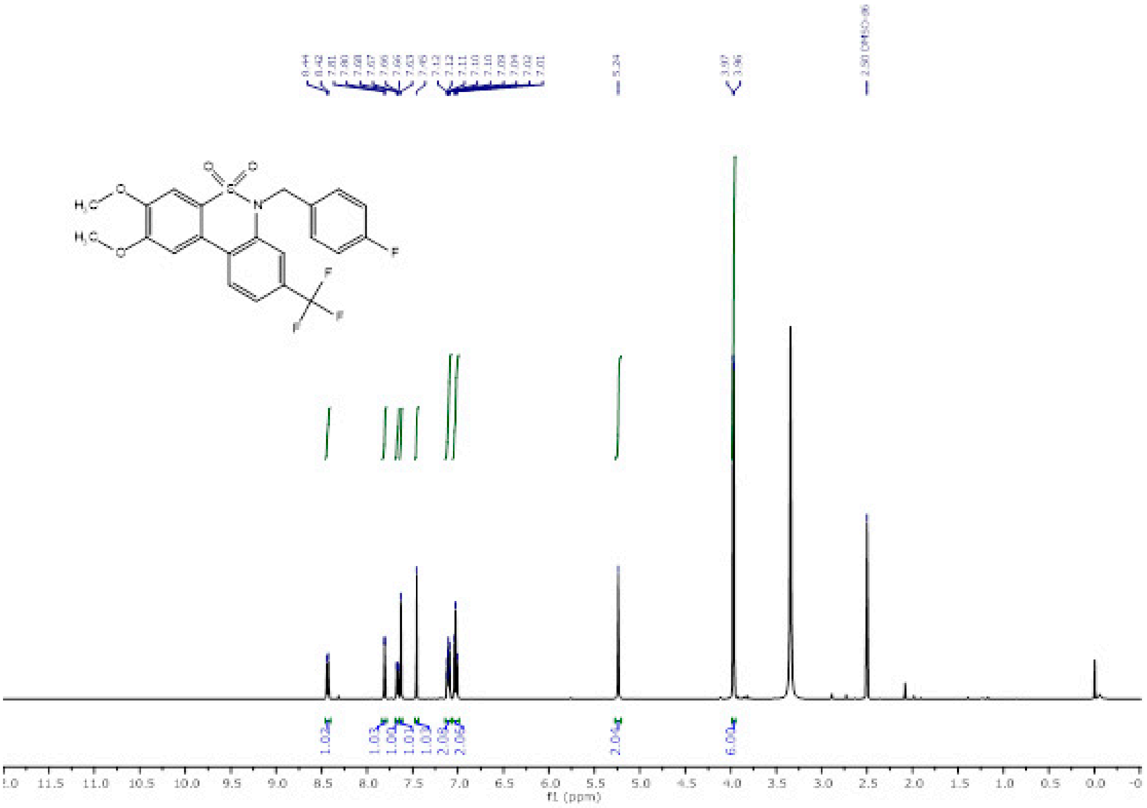

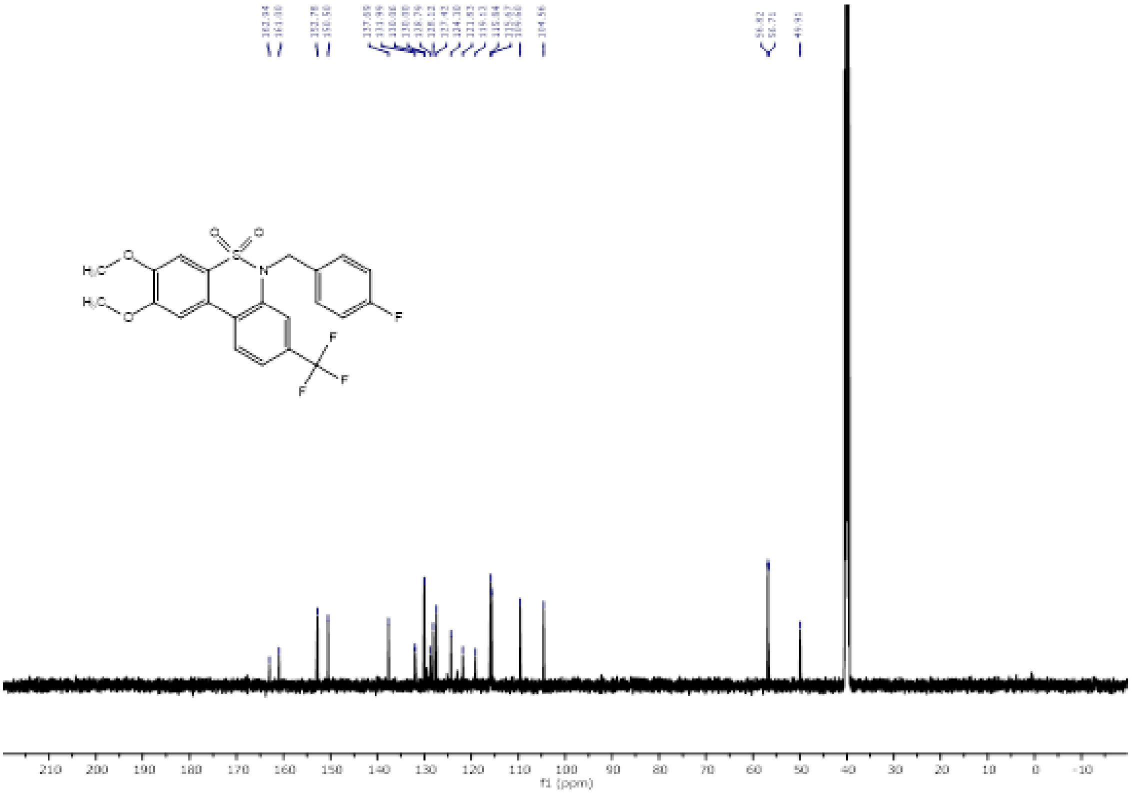

6-(4-fluorobenzyl)-2,3,9-trihydroxy-6H-dibenzo[c,e][1,2]thiazine 5,5-dioxide ***(18a)****:*

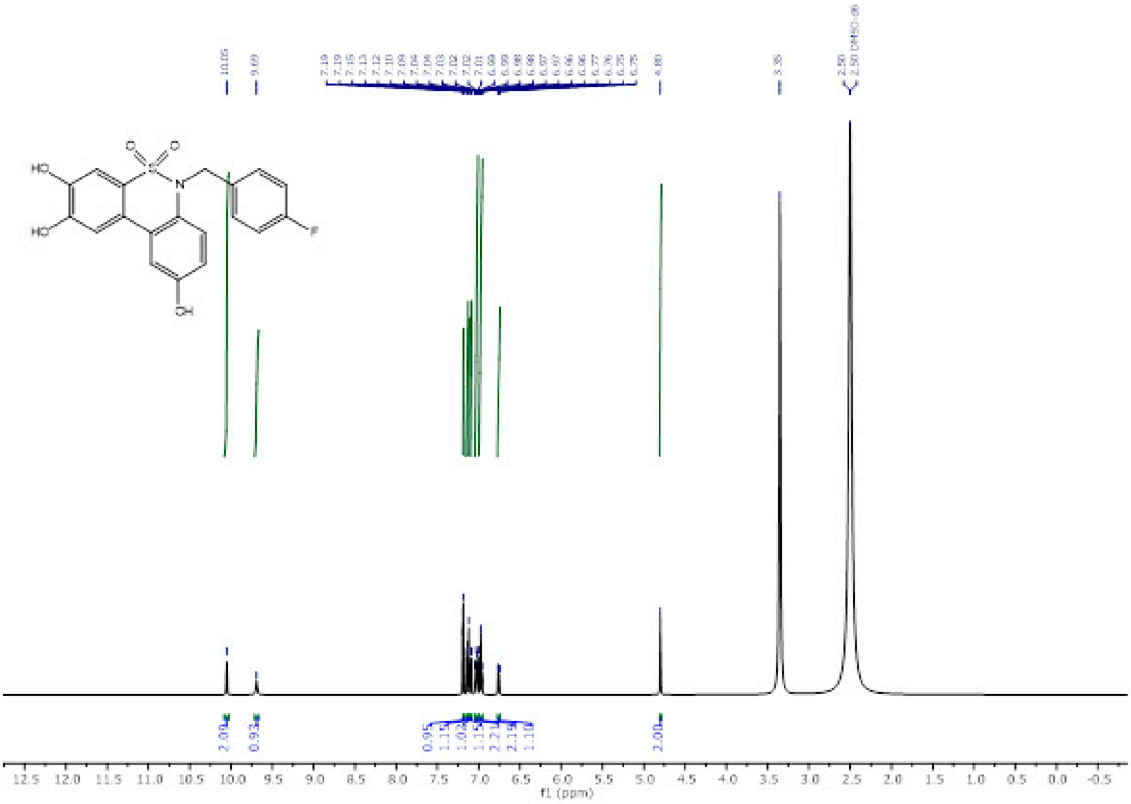

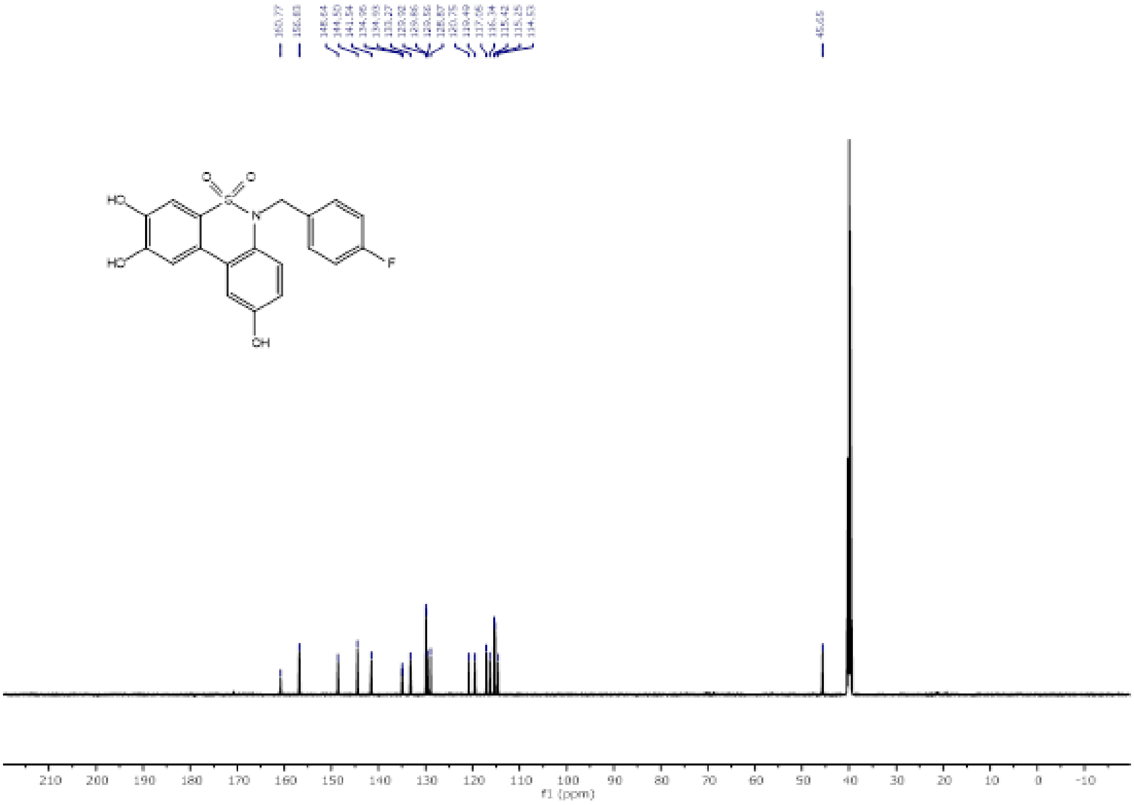

6-(4-fluorobenzyl)-2,3,8-trihydroxy-6H-dibenzo[c,e][1,2]thiazine 5,5-dioxidedioxide ***(18b)****:*

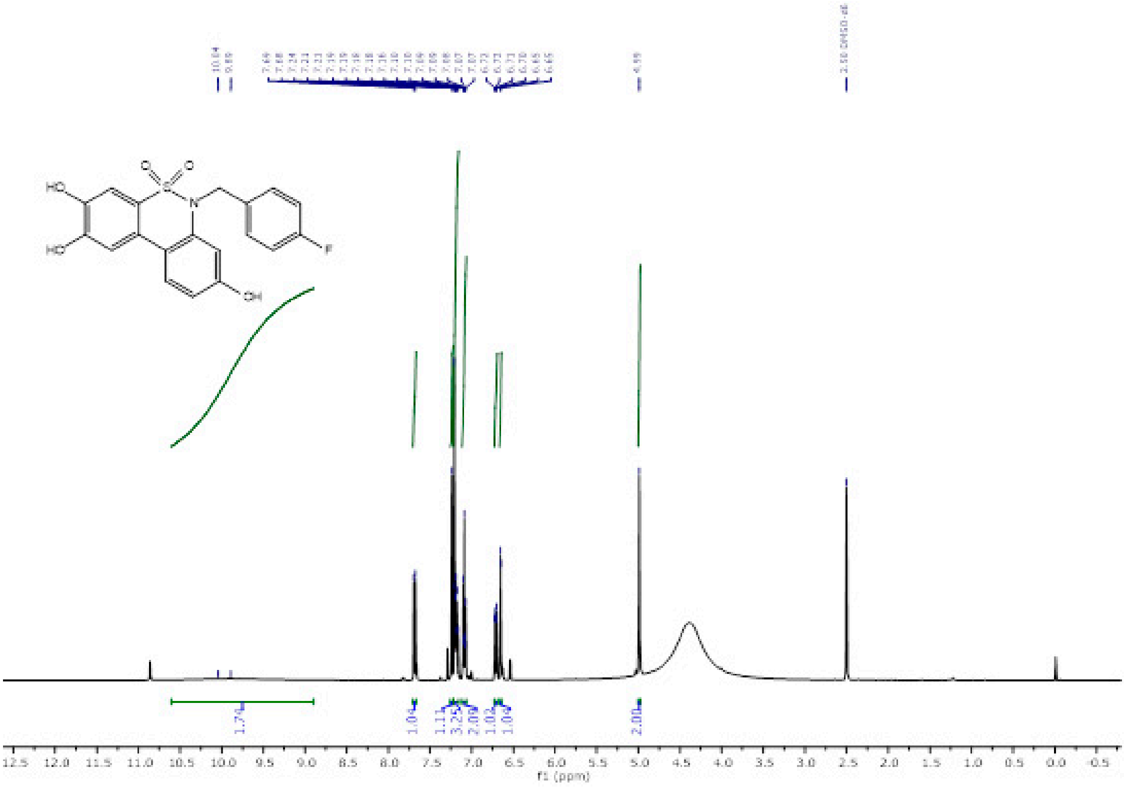

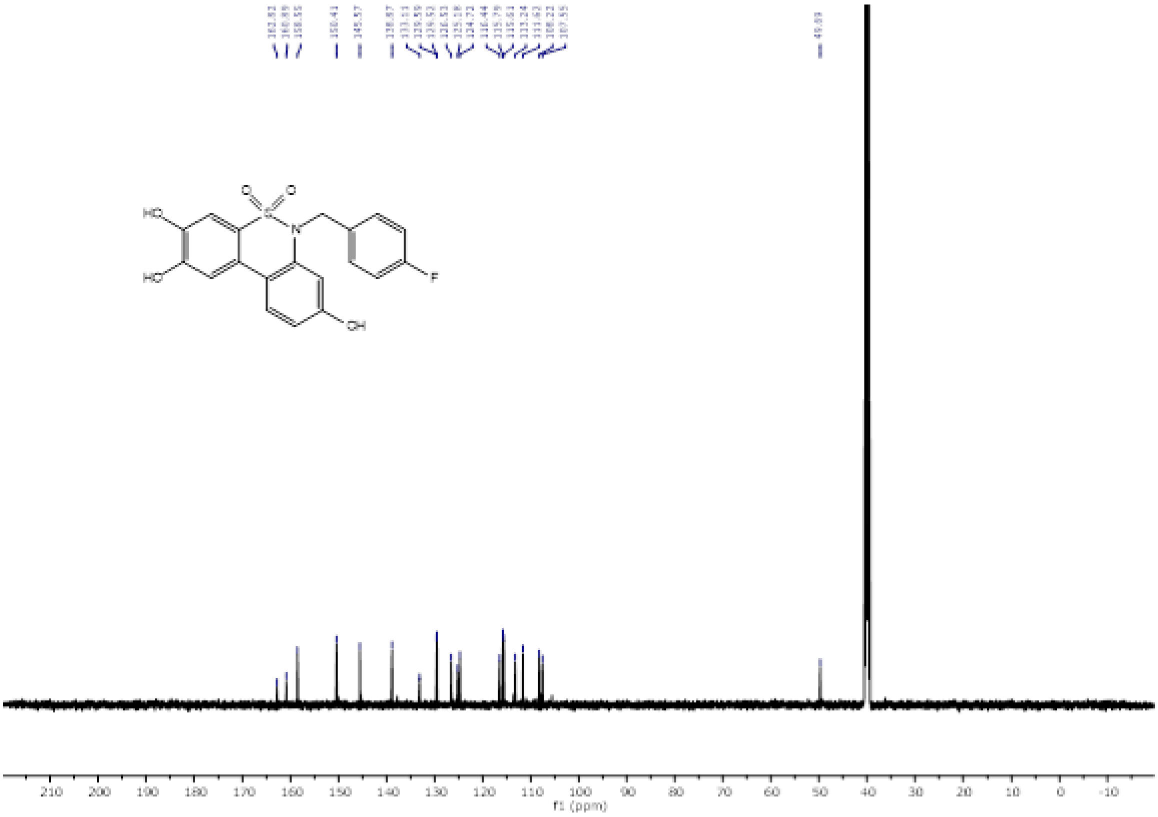

6-(4-fluorobenzyl)-2,3,7,9-tetrahydroxy-6H-dibenzo[c,e][1,2]thiazine 5,5-dioxide ***(18c)****:*

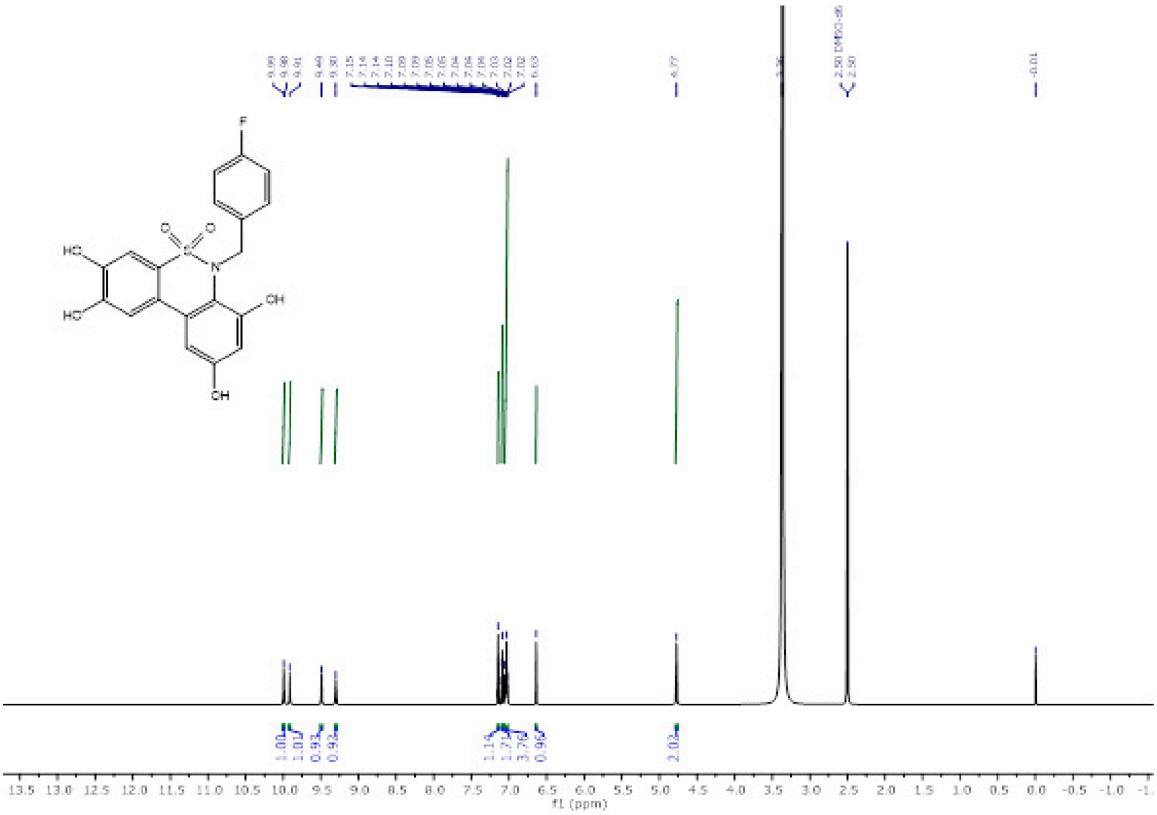

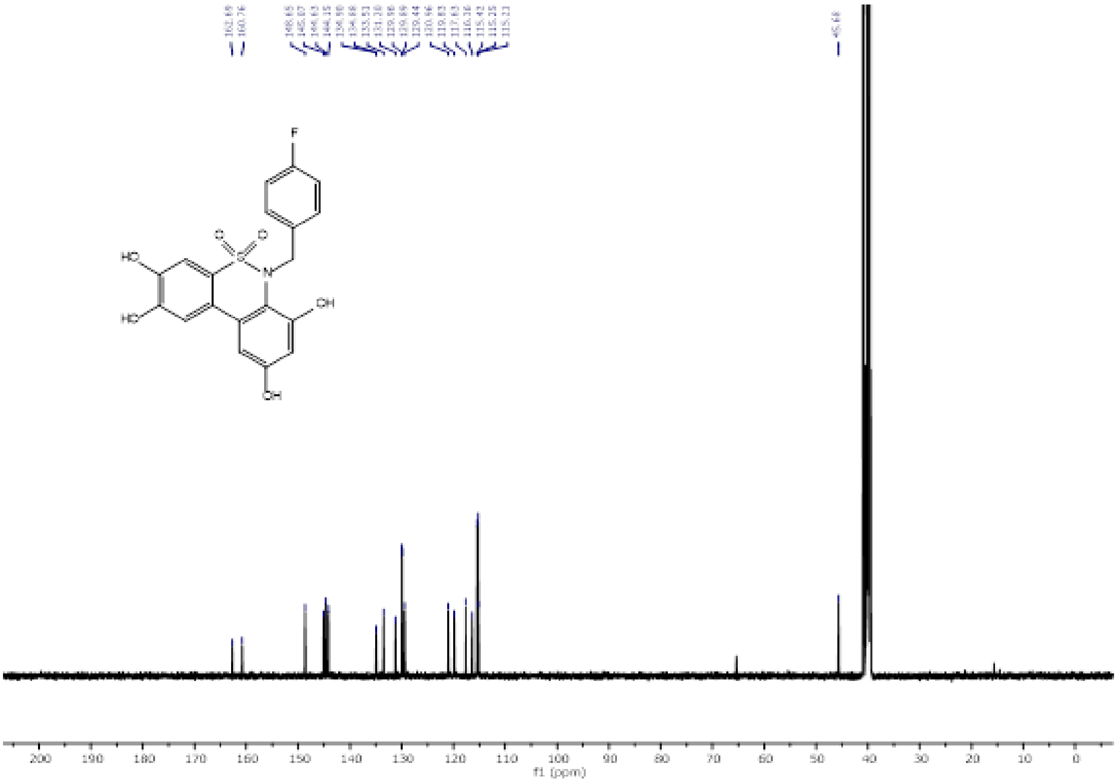

6-(4-fluorobenzyl)-2,3,8,9-tetrahydroxy-6H-dibenzo[c,e][1,2]thiazine 5,5-dioxide ***(18d)****:*

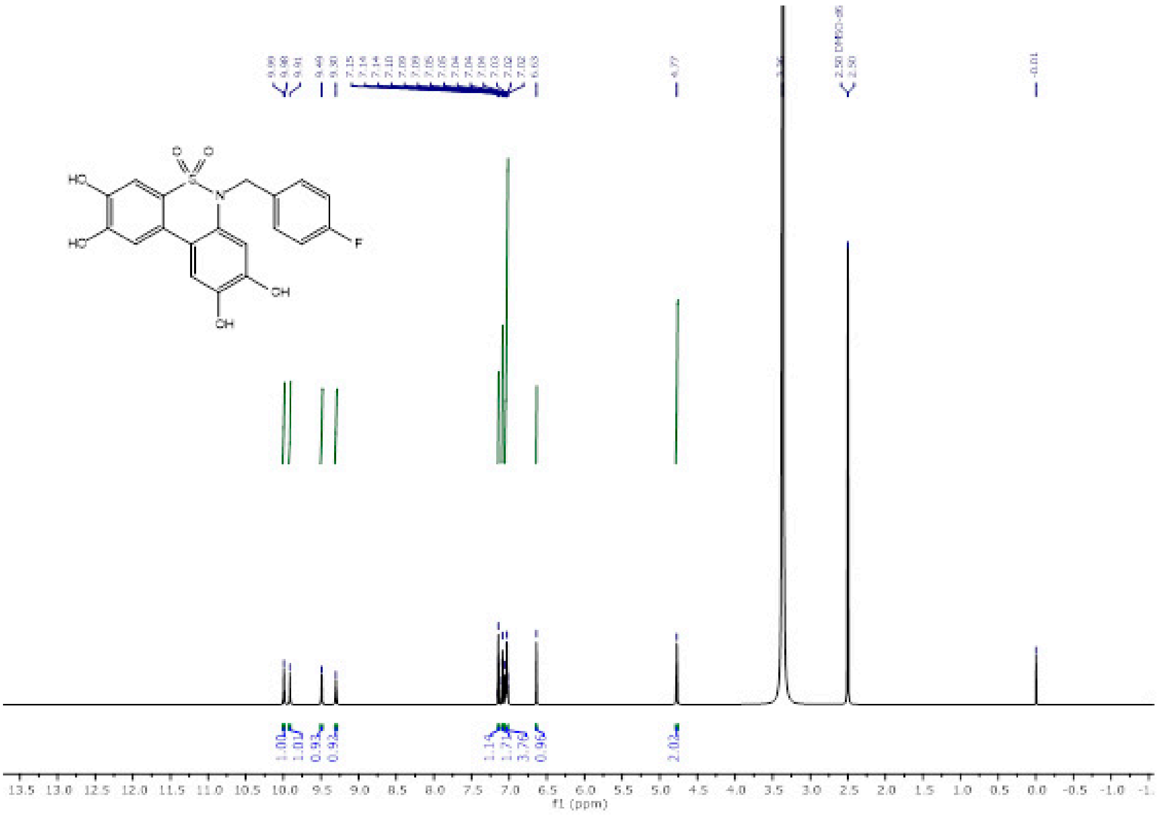

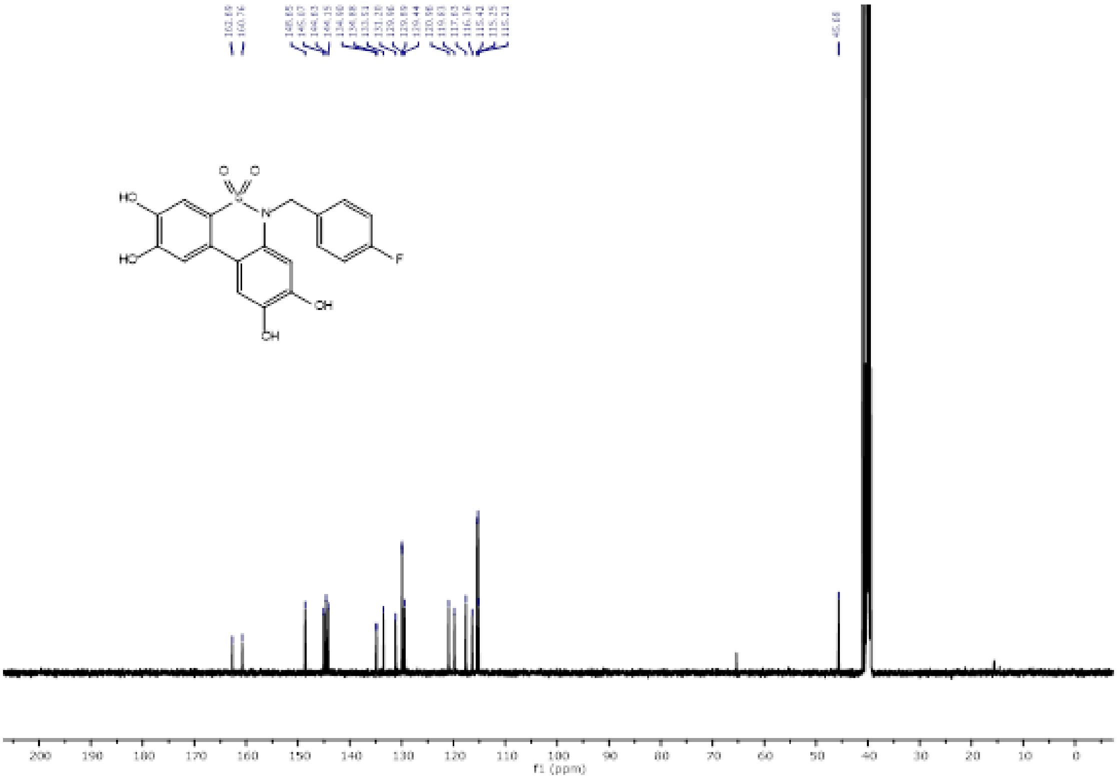

6-(4-fluorobenzyl)-2,3-dihydroxy-8-(trifluoromethyl)-6H-dibenzo[c,e][1,2]thiazine 5,5-dioxide ***(18e)****:*

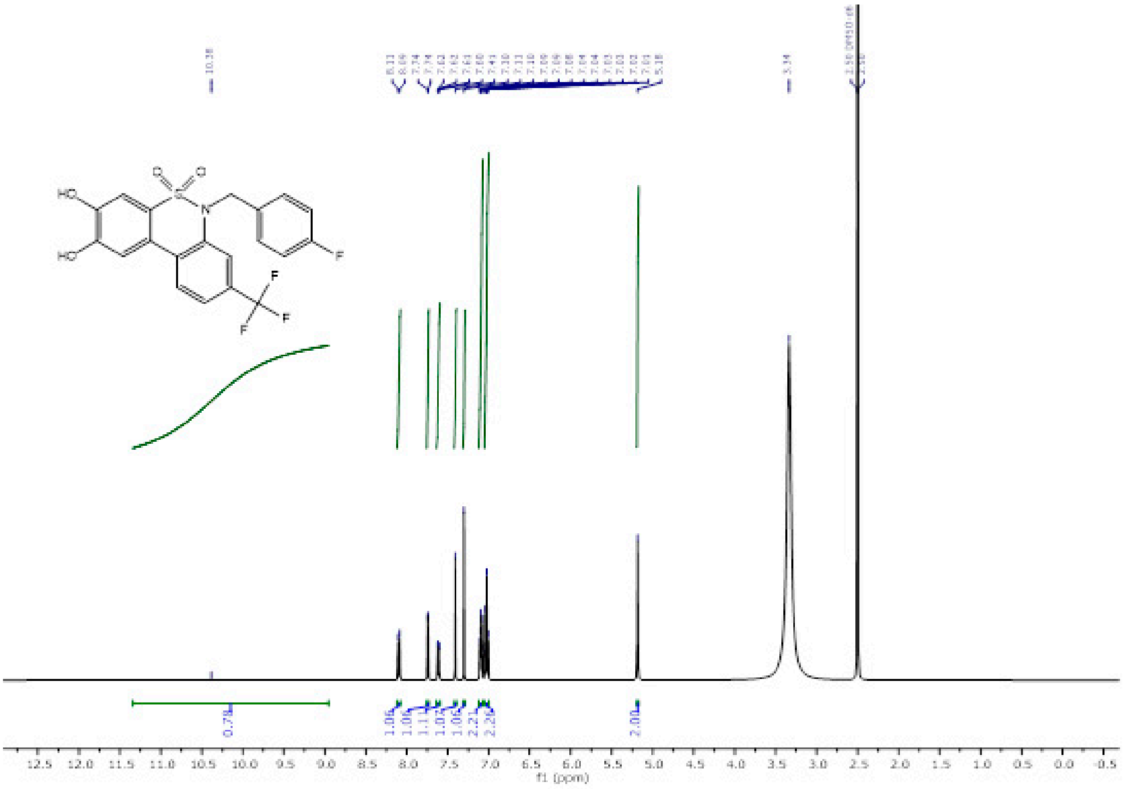

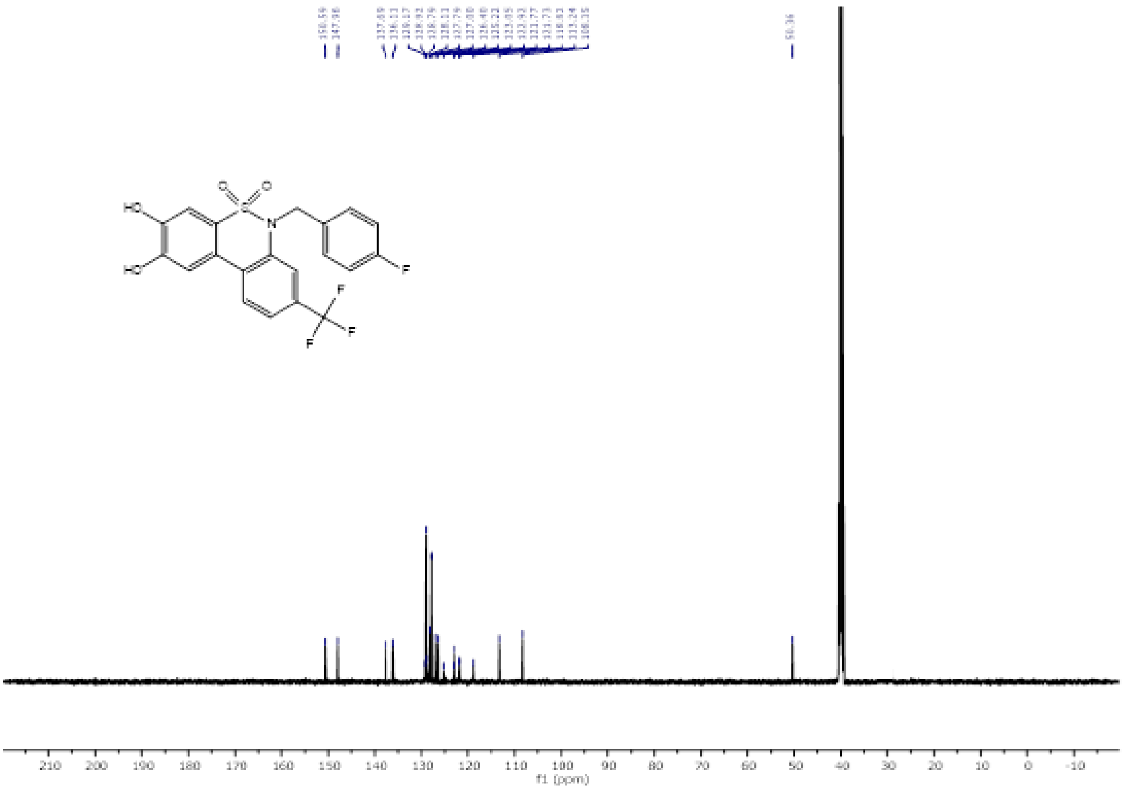

2,3,8,9-tetrahydroxy-6H-dibenzo[c,e][1,2]thiazine 5,5-dioxidee ***(21a)****:*

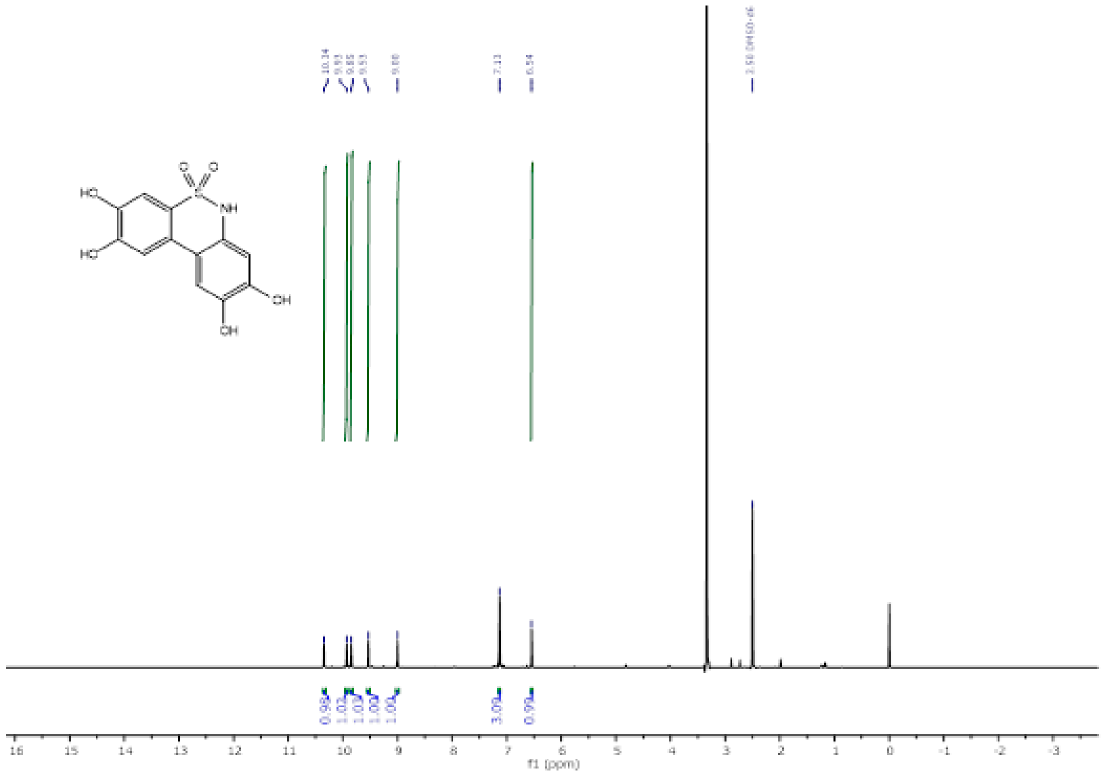

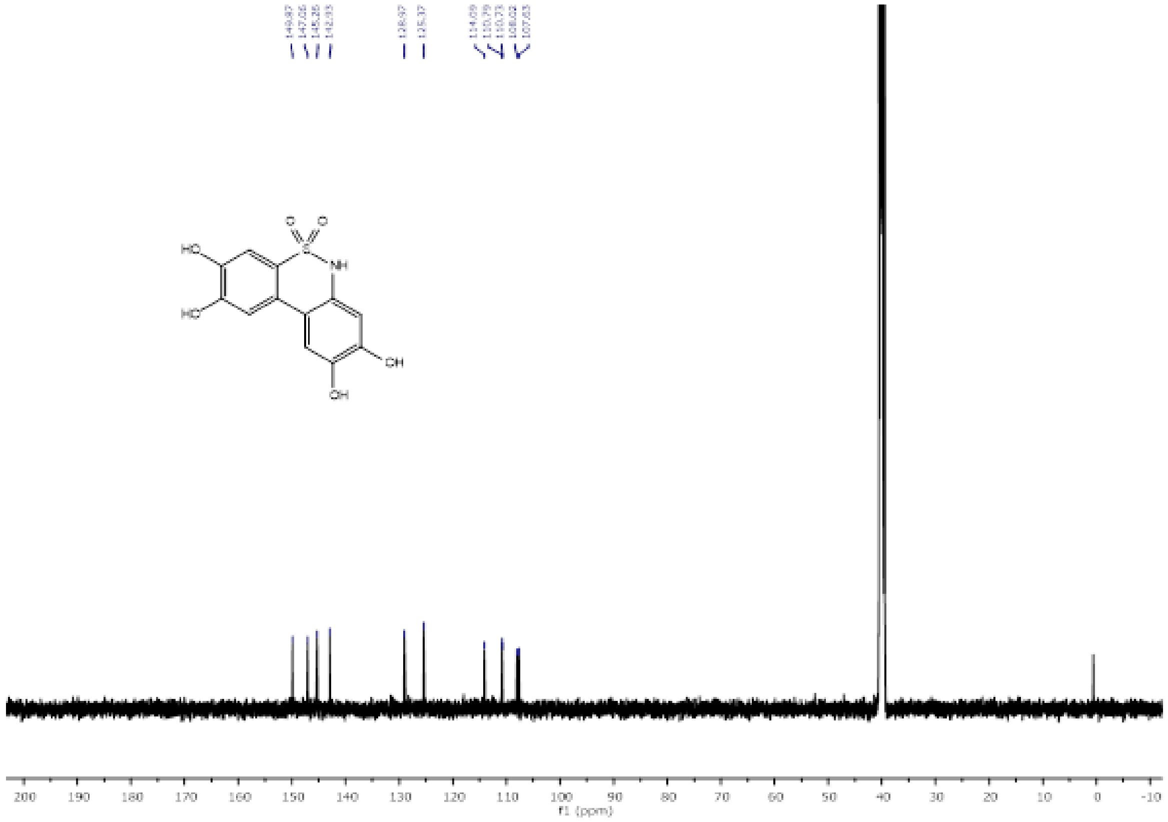

2,3,7,9-tetrahydroxy-6H-dibenzo[c,e][1,2]thiazine 5,5-dioxide ***(21b)****:*

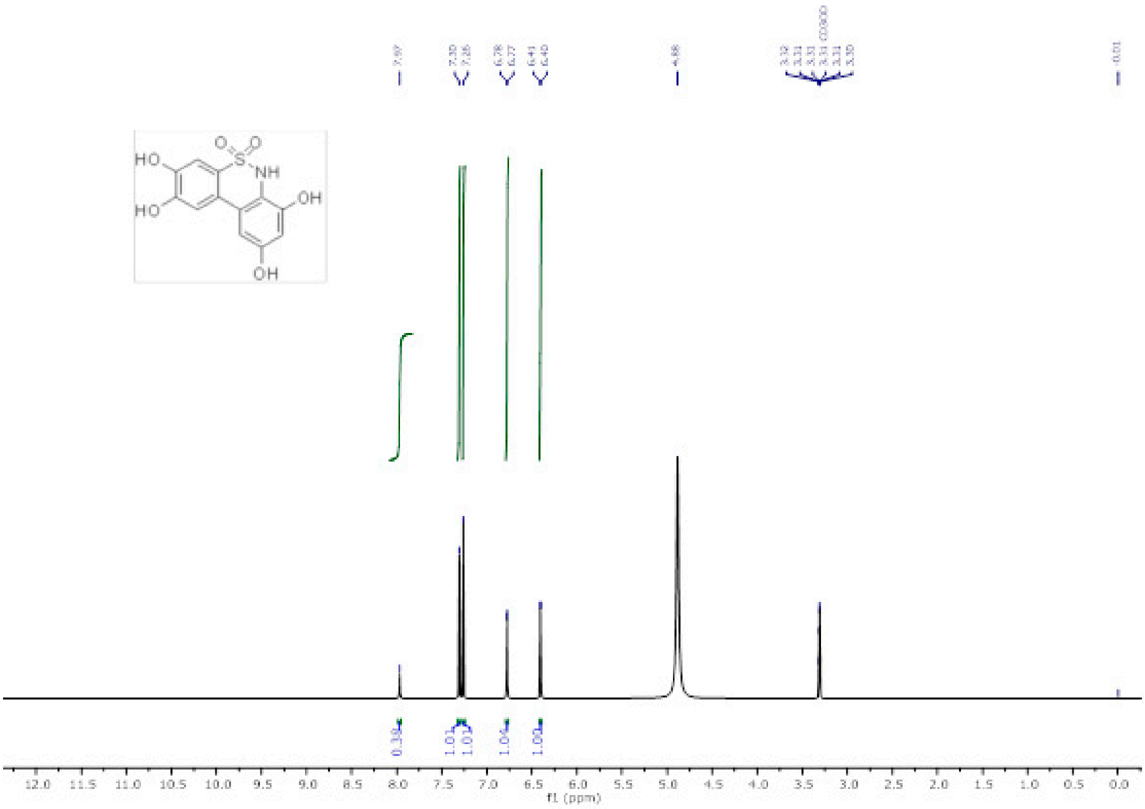

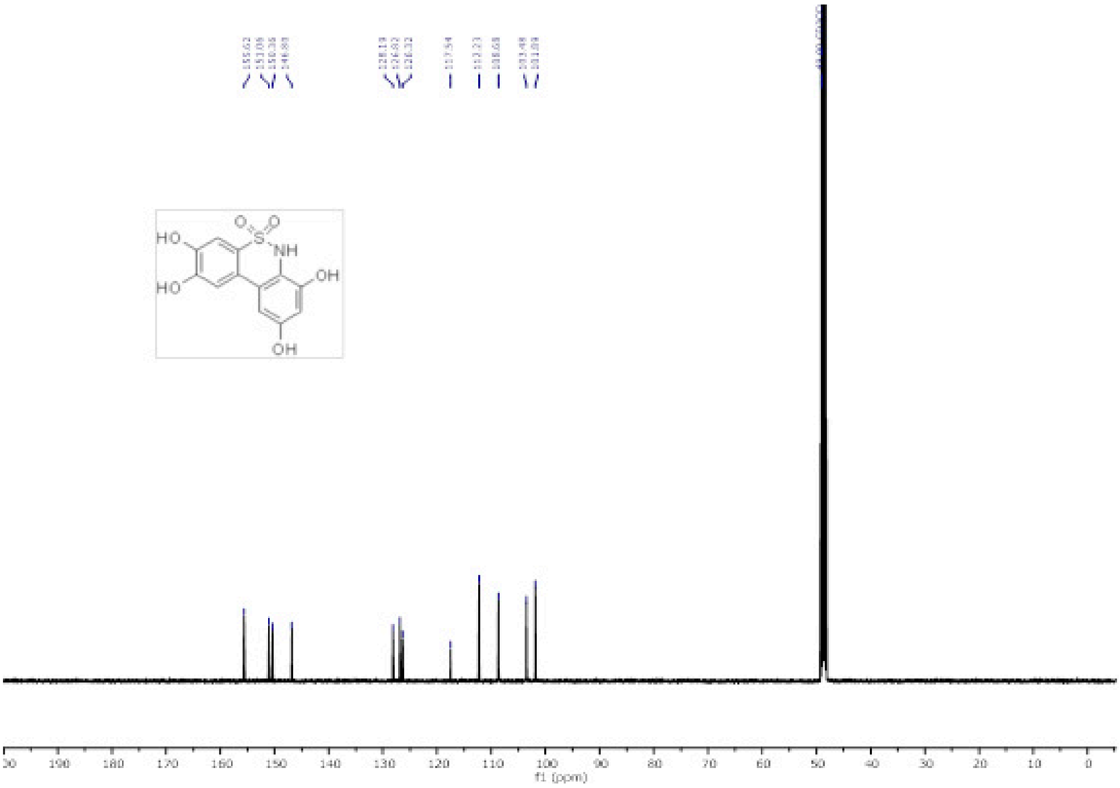

Supplement: Supplementary file 1 [file ijms-25-07986-s001.zip › ijms-3070304-supplementary.pdf]
